# Palladium-catalyzed regiodivergent hydrochlorocarbonylation of alkenes for formation of acid chlorides

**DOI:** 10.1038/s41467-023-38748-3

**Published:** 2023-05-31

**Authors:** Fei Wu, Bo Wang, Na-Qi Li, Hui-Yi Yang, Zhi-Hui Ren, Zheng-Hui Guan

**Affiliations:** Key Laboratory of Synthetic and Natural Functional Molecule of the Ministry of Education, Department of Chemistry & Materials Science, Northwest University, 710127 Xi’an, P. R. China

**Keywords:** Synthetic chemistry methodology, Catalyst synthesis, Homogeneous catalysis

## Abstract

Novel strategy for acid chlorides formation that do not use carboxylic acids is particularly attractive in chemical synthesis but remains challenging. Herein, we reported the development of a highly effective Pd-catalyzed hydrochlorocarbonylation of alkenes with CO for the formation of alkyl acid chlorides. Chlorosilane and AcOH were found as a mild HCl source for the reaction. The reaction shows broad substrate scope and produces both branched and linear alkyl acid chlorides in good to high yields upon different ligands and solvents. Cooperating with follow-up acylation reactions, the Pd-catalyzed hydrochlorocarbonylation offers a complementary platform for the synthesis of diverse carbonyl compounds from alkenes. Mechanistic investigations suggested that the reaction proceeded though a palladium hydride pathway, and CO prompted reductive elimination of the acyl-Pd-Cl intermediate.

## Abstract (web summary)

Acid chloride formation is generally limited to the chlorination of carboxylic acids. Here, the authors report a palladium-catalyzed regiodivergent hydrochlorocarbonylation of alkenes for the synthesis of various alkyl acid chlorides.

## Introduction

**Fig. 1:**
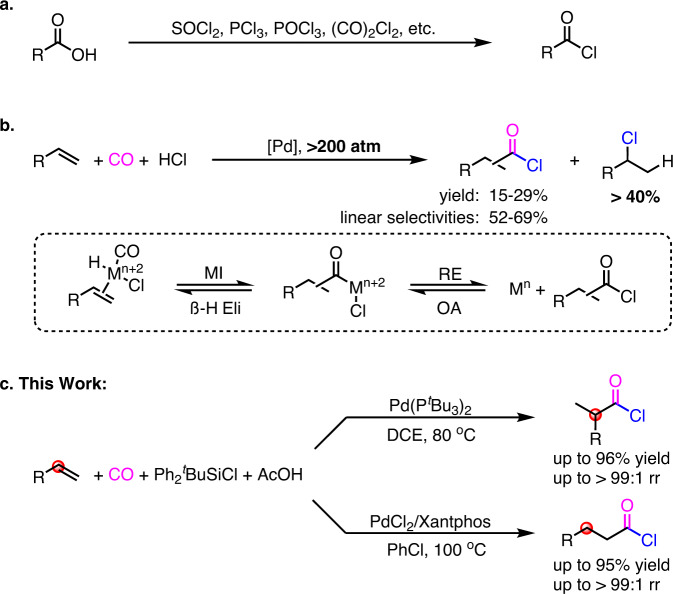
New strategies for acid chlorides’ formation. **a** Traditional methods for the synthesis of acid chlorides. **b** The original Pd-catalyzed hydrochlorocarbonylation of alkenes (1975). **c** Our work: Pd-catalyzed regiodivergent hydrochlorocarbonylation of alkenes with chlorosilane and AcOH. R, alkyl or aryl.

The acid chlorides are one of the most versatile and useful building blocks, which have been widely applied in esters or amides formation, Friedel-Craft acylation, cross-couplings, polymerizations and many so forth^1^. Conventionally, they are synthesized through chlorination of carboxylic acids with SOCl_2_, PCl_3_, POCl_3_, (CO)_2_Cl_2_ or other chlorinating reagents (Fig. 1a)^2,3^. Nevertheless, heavily dependent on carboxylic acids as the substrates often limits the availability of the desired acid chlorides in target-oriented synthesis, that is especially true in the case of carboxylic acids were inaccessible. As a result, the direct conversion of organic functional groups into the synthetically useful acid chlorides is of great significance in chemical synthesis^4,5^. Recently, palladium-catalyzed chlorocarbonylations of aryl halides using CO as a cheap and readily available C1 source for the formation of aryl acid chlorides have been developed by Arndtsen and coworkers^6–9^. Palladium-catalyzed isodesmic shuttle reaction of unsaturated hydrocarbons and butyryl chloride to afford acid chlorides has been developed by Morandi and coworkers^10–12^. Notwithstanding, the development of novel and general strategies to access various alkyl acid chlorides from readily available starting materials remain highly desirable.

Palladium-catalyzed hydrocarbonylations of alkenes represent a fundamental and powerful tool for the formation of carbonyl compounds^13–15^. With different nucleophiles in the reaction, the hydrocarbonylations create diverse carbonyl compounds such as amides^16–23^, esters^24–32^, and carboxylic acids^33–40^, with full atom economy. In this context, hydrochlorocarbonylation, a palladium-catalyzed hydrocarbonylation of alkenes with CO and HCl is supposed to be a possible approach to access acid chlorides in theory. However, distinct from the aminolysis/alcoholysis/hydrolysis of the key acyl-Pd-X intermediates in traditional hydrocarbonylations, the chlorolysis of the acyl-Pd-X species is considered be less likely due to the weak nucleophilicity of HCl. Whereas reductive elimination of the acyl-Pd-Cl species is assumed to form acid chlorides, the generated high reactive acid chlorides are readily undergo re-oxidative addition to palladium(0) catalyst^41,42^, thus leading to the hydrochlorocarbonylation proceeded in very low efficiency and with uncontrollable regioselectivity (Fig. 1b)^43,44^. Moreover, the inevitable side hydrochlorination of alkenes by HCl (even became predominant reaction) is also importantly burned off the yield of desired acid chloride^43,44^. As a result, the original hydrochlorocarbonylation reaction has less been used in organic synthesis and less been known in the past half-century. Aiming to gain new and flexible methods for the formation of synthetically important alkyl acid chlorides, and based on our interest on palladium-catalyzed hydrocarbonylations^16–18,33^, herein, we report the development of a highly effective palladium-catalyzed regiodivergent hydrochlorocarbonylation of alkenes with CO (Fig. 1c).

## Results and discussion

### Optimization of reaction conditions

We initiated the investigations of Pd-catalyzed hydrochlorocarbonylation with styrene **1a** as the substrate at 80 °C in DCE under CO (Table 1). With Pd(PPh_3_)_2_Cl_2_ as the catalyst, the mixture of regioisomers of acid chlorides **2a/3a** were indeed observed in 15% yield in the presence of HCl (entry 1). Unfortunately, due to the unavoidable hydrochlorination side-reaction initiated by HCl, the yield and regioselectivity of the reaction could not be improved after several attempts. We hypothesized that using in situ generated HCl might partially overcome the undesired hydrochlorination side reaction^45^. Therefore, a number of protocols for in situ formation of HCl have been examined in the reaction (entries 2–5)^46^. A 22% yield of acid chlorides **2a/3a** were obtained with Ph_2_^*t*^BuSiCl/EtOH as the HCl source (entry 5). Nevertheless, the drawback of this Ph_2_^*t*^BuSiCl/EtOH reagent is of the accompanied hydroethanoxycarbonylation side reaction caused by EtOH. To this end, the Ph_2_^*t*^BuSiCl/AcOH reagent was found to be the best choice for hydrochlorocarbonylation, afford acid chlorides **2a/3a** in 39% yields (entry 6).

**Table 1:** Optimization of the reaction conditions^a^


Entry	[Pd]	Ligand	[HCl]	Solvent	Yield (%) 2a + 3a	Ratio of 2a/3a
1	Pd(PPh_3_)_2_Cl_2_	–	HCl	DCE	15%	76:24
2	Pd(PPh_3_)_2_Cl_2_	–	CH_3_COCl + EtOH	DCE	3%	ND
3	Pd(PPh_3_)_2_Cl_2_	–	TMSCl+EtOH	DCE	5%	ND
4	Pd(PPh_3_)_2_Cl_2_	–	Ph_3_SiCl + EtOH	DCE	17%	75:25
5	Pd(PPh_3_)_2_Cl_2_	–	Ph_2_^*t*^BuSiCl + EtOH	DCE	22%	73:27
6	Pd(PPh_3_)_2_Cl_2_	–	Ph_2_^*t*^BuSiCl + AcOH	DCE	39%	73:27
7	PdCl_2_	P(2-MeOPh)_3_	Ph_2_^*t*^BuSiCl + AcOH	DCE	34%	88:12
8	PdCl_2_	PAd_2_^*n*^Bu	Ph_2_^*t*^BuSiCl + AcOH	DCE	30%	>99:1
9	PdCl_2_	P^*t*^BuPh_2_	Ph_2_^*t*^BuSiCl + AcOH	DCE	56%	90:10
10	PdCl_2_	P^*t*^Bu_3_	Ph_2_^*t*^BuSiCl + AcOH	DCE	90%	97:3
11	Pd(P^*t*^Bu_3_)_2_	–	Ph_2_^*t*^BuSiCl + AcOH	DCE	96%	>99:1
12	PdCl_2_	Dtbpx	Ph_2_^*t*^BuSiCl + AcOH	DCE	NR	ND
13	PdCl_2_	DPE-Phos	Ph_2_^*t*^BuSiCl + AcOH	DCE	73%	33:67
14	PdCl_2_	Xantphos	Ph_2_^*t*^BuSiCl + AcOH	DCE	67%	28:72
15	PdCl_2_	^*t*^Bu-Xantphos	Ph_2_^*t*^BuSiCl + AcOH	DCE	42%	49:51
16	PdCl_2_	Xantphos	Ph_2_^*t*^BuSiCl + AcOH	THF	86%	37:63
17	PdCl_2_	Xantphos	Ph_2_^*t*^BuSiCl + AcOH	Toluene	75%	10:90
18	PdCl_2_	Xantphos	Ph_2_^*t*^BuSiCl + AcOH	PhCl	64%	4:96
19^b^	PdCl_2_	Xantphos	Ph_2_^*t*^BuSiCl + AcOH	PhCl	95%	5:95

^*t*^*Bu* tertiary-butyl, ^*n*^*Bu* normal-butyl.

^a^Conditions: styrene **1a** (0.2 mmol), [Pd] (5 mol%), bisphosphine ligand (6 mol%) or monophosphine ligand (10 mol%), [SiCl] (0.3 mmol), EtOH or AcOH (0.3 mmol), solvent (1.5 mL), CO (30 atm), 80 °C for 24 h. The yields of **2a** + **3a** and ratio of regioisomers **2a/3a** were determined by GC-MS analysis of their derivatized methyl carboxylate using *n*-hexadecane as the internal standard (see SI).

^b^CO (40 atm), 100 °C for 24 h.

Since reductive elimination of the acyl-Pd(L)-Cl species might be a key factor of the reaction efficiency, various ligands, which might improve the reactivity and tune the regioselectivity of the reaction, were screened (entries 7–15). We were found that the use of monophosphines favors to access branched acid chloride **2a**, while the use of bisphosphine ligands favors to access linear product **3a**^47^. In this context, the sterically bulky P^*t*^Bu_3_ showed the highest efficiency for the formation of the branched acid chloride **2a**, thus offering the optimal conditions for branched selective hydrochlorocarbonylation of alkenes (entry 11). In contrast, although the linear acid chloride **3a** was observed as the main product in the presence of Xantphos ligand, the yield and regioselectivity remains to be improved at this stage (entry 14). Therefore, other parameters including solvents, temperatures and pressure of CO were extensively optimized. After many attempts, a 95% yield of acid chloride **3a** with l/b = 95:5 was finally obtained by using chlorobenzene as the solvent at 100 °C (entry 19).

### Hydrochlorocarbonylation of alkenes

**Fig. 2:**
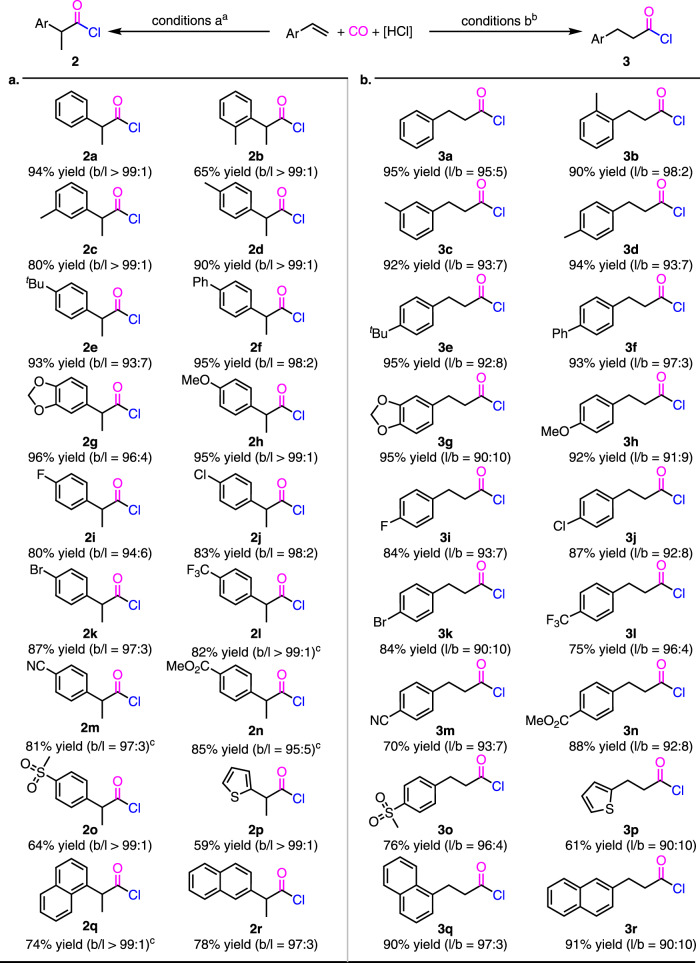
Pd-catalyzed regiodivergent hydrochlorocarbonylation of aryl alkenes. ^a^Conditions: alkene (0.2 mmol), Pd(P^*t*^Bu_3_)_2_ (5 mol%), Ph_2_^*t*^BuSiCl (0.3 mmol), AcOH (0.3 mmol), CO (30 atm), DCE (1.5 mL), 80 °C, 24 h. ^b^Conditions: alkene (0.2 mmol), PdCl_2_ (5 mol%), Xantphos (6 mol%), Ph_2_^*t*^BuSiCl (0.3 mmol), AcOH (0.3 mmol), CO (40 atm), PhCl (1.5 mL), 100 °C, 24 h. ^c^100 °C. Yields refer to isolated yields of the derivatized methyl carboxylates (see SI). The ratios of regioisomers within parentheses were determined by GC-MS analysis of their derivatized methyl carboxylates of crude acid chlorides.

With the optimized reaction conditions in hand, the scope of the hydrochlorocarbonylation have been explored (Fig. 2). Under the conditions for the synthesis of branched acid chlorides, Pd-catalyzed hydrochlorocarbonylation of aryl alkenes proceeded well to give rise to the corresponding products in high yields with excellent regioselectivities (Fig. 2a). The functional group tolerance of the reaction is of broad. Both electron-donating and electron-withdrawing groups, such as alkyl, alkoxyl, [1,3]-dioxol, F-, Cl-, Br-, CF_3_-, MeO_2_C-, sulfonyl, and even coordinative cyano-, were compatible under the conditions (**2b-2o**). In addition, 2-vinylthiophene and vinylnaphthalenes were tolerated in the reaction to afford the desired acid chlorides **2p–2r** in good to high yields. In contrast, under the conditions for the synthesis of linear acid chlorides, Pd-catalyzed hydrochlorocarbonylation of aryl alkenes also proceeded well to generate the corresponding linear acid chlorides in high yields with high regioselectivities (Fig. 2b). The substrate scope and functional group tolerance in these cases (**3b–3r**) were as good as the branched selective hydrochlorocarbonylation.

**Fig. 3:**
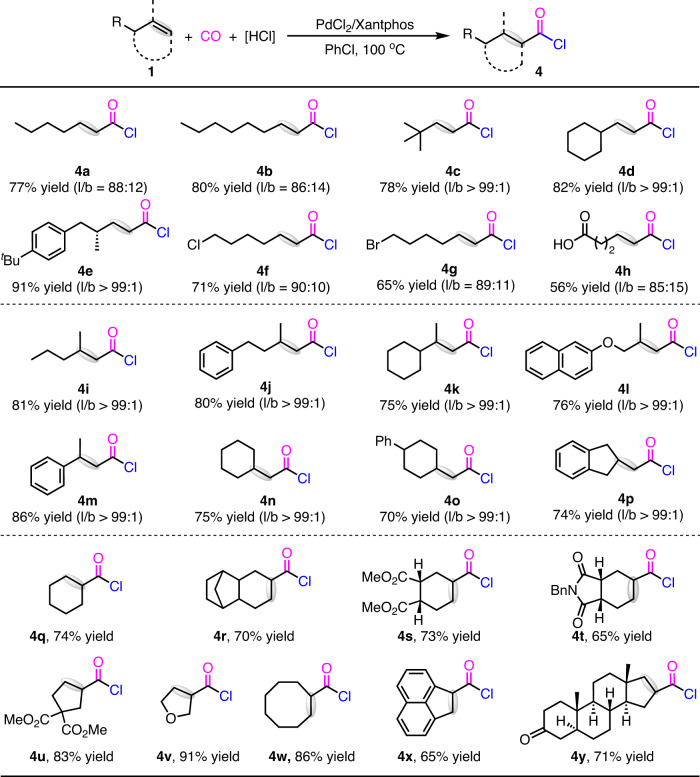
Pd-catalyzed hydrochlorocarbonylation of various alkenes. Conditions: alkene (0.2 mmol), PdCl_2_ (5 mol%), Xantphos (6 mol%), Ph_2_^*t*^BuSiCl (0.3 mmol), AcOH (0.3 mmol), CO (40 atm), PhCl (1.5 mL), 100 °C, 24 h. Isolated yields of the derivatized products (see SI). The ratio of isomers within parentheses was determined by GC-MS analysis of the derivatized products of the acid chlorides.

The substrate scope with respect to alkyl alkenes was investigated (Fig. 3). The linear selective hydrochlorocarbonylation of alkyl alkenes were proceeded well, while the branched selective reaction was unsuccessful under the above standard conditions. As such, both non-functionalized alkenes and functionalized alkenes including lilestralis-derived alkene, 6-chlorohex-1-ene, 6-bromohex-1-ene, pent-4-enoic acid were proceeded under the linear selective conditions to form the carbon chain extended acid chlorides **4a–4h** in high yields. With a bulky substituent adjacent to the alkene group, the regiomeric ratios (l:b) of products **4c–4e** could be as high as >99:1. Expectedly, with 1,1-dialkyl substituted alkenes, 1-aryl-1-alkyl substituted alkenes, or exocyclic alkenes as the substrate, the corresponding acid chlorides **4i–4p** were obtained in 70–86% yields.

Cyclic internal alkenes were feasible substrates in the reaction to form the desired acid chlorides **4q–4y** in good to high yields. Whereas the isomerization of the alkyl substituted alkenes is typical a low energy barrier pathway in traditional hydrofunctionalizations^48–50^, the desired acid chlorides **4r–4v** were obtained as the sole products in high yields. These results indicated that the insertion of [PdH] species into the double bond of the alkenes is irreversible in our hydrochlorocarbonylation. Notably, acenaphthylene and androstenone were tolerated in the reaction to afford the corresponding acid chloride **4x–4y** in 65% to 71% yield.

### Synthetic application of hydrochlorocarbonylation

**Fig. 4:**
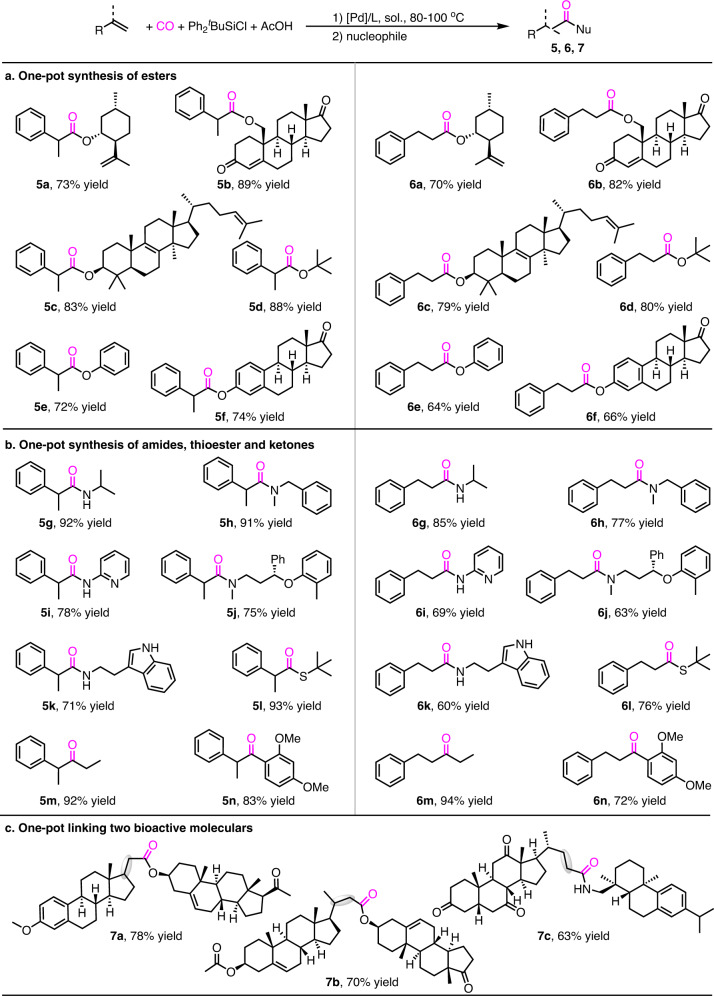
Synthetic application of hydrochlorocarbonylation. Conditions for **a**, **b**, left column: alkene (0.2 mmol), Pd(P^*t*^Bu_3_)_2_ (5 mol%), Ph_2_^*t*^BuSiCl (0.3 mmol), AcOH (0.3 mmol), CO (30 atm), DCE (1.5 mL), 80 °C, 24 h. Conditions for **a**, **b**, right column and **c**: alkene (0.2 mmol), PdCl_2_ (5 mol%), Xantphos (6 mol%), Ph_2_^*t*^BuSiCl (0.3 mmol), AcOH (0.3 mmol), CO (40 atm), PhCl (1.5 mL), 100 °C, 24 h. After completed the hydrochlorocarbonylation, the crude acid chloride was involved in follow-up acylation with the corresponding nucleophiles in one-pot. Isolated yields.

The efficiency of above Pd-catalyzed hydrochlorocarbonylation encouraged us to establish a complementary platform for the synthesis of diverse carbonyl compounds from alkenes in one-pot manner (Fig. 4). In this respect, although the transformation of alkenes to esters and amides have been developed as exemplified by Pd-catalyzed hydroalkoxycarbonylation and hydroaminocarbonylation, the substrate scope of these reactions is often limited in primary alcohols and anilines^13–40^. Tertiary alcohols, phenols and aliphatic amines are typically incompatible with the traditional hydrocarbonylations because of their steric encumbrance, weak nucleophilicity, and strong basicity. Therefore, one-pot hydrochlorocarbonylation of alkenes and esterification with sterically encumbered alcohols or weakly nucleophilic phenols for the synthesis of corresponding esters has been explored. As illustrated in Fig. 4a, sterically encumbered bioactive alcohols, such as (-)-isopulegol, 19-hydroxyandrostendione, and lanosterol, were well tolerated in one-pot reactions to afford the desired esters **5a–5c** and **6a–6c** in good to high yields. Tertiary butanol and weakly nucleophilic phenol and estrone were also tolerated in one-pot reactions to form the corresponding esters **5d–5f** and **6d–6f** in good yields.

One-pot hydrochlorocarbonylation and amidation with aliphatic amines have subsequently been studied (Fig. 4b). Aliphatic amines and 2-aminopyridine that inhibit the generation of active metal-hydride catalyst in hydroaminocarbonylation reactions, could be easily transformed into amides **5g–5k** and **6g–6k** in good to high yields in one-pot hydrochlorocarbonylation-amidation reactions. Additionally, as illustrated in **5l–5n** and **6l–6n**, one-pot hydrochlorocarbonylation-thioesterification, one-pot hydrochlorocarbonylation-C-C bond coupling reaction, and one-pot hydrochlorocarbonylation-Friedel-Crafts acylation were proceeded smoothly, thus demonstrating the usefulness of this Pd-catalyzed hydrochlorocarbonylation of alkenes.

In order to further demonstrate the utility of the hydrochlorocarbonylation, late-stage functionalization of complex bioactive molecules has been examined (Fig. 4c). Delightfully, the hydrochlorocarbonylation showed high efficiency in these cases, following by one-pot esterification/amidation, the ester/amide **7a–7c** that linked two bioactive molecules were obtained in 63–78% yield.

### Mechanistic studies

**Fig. 5:**
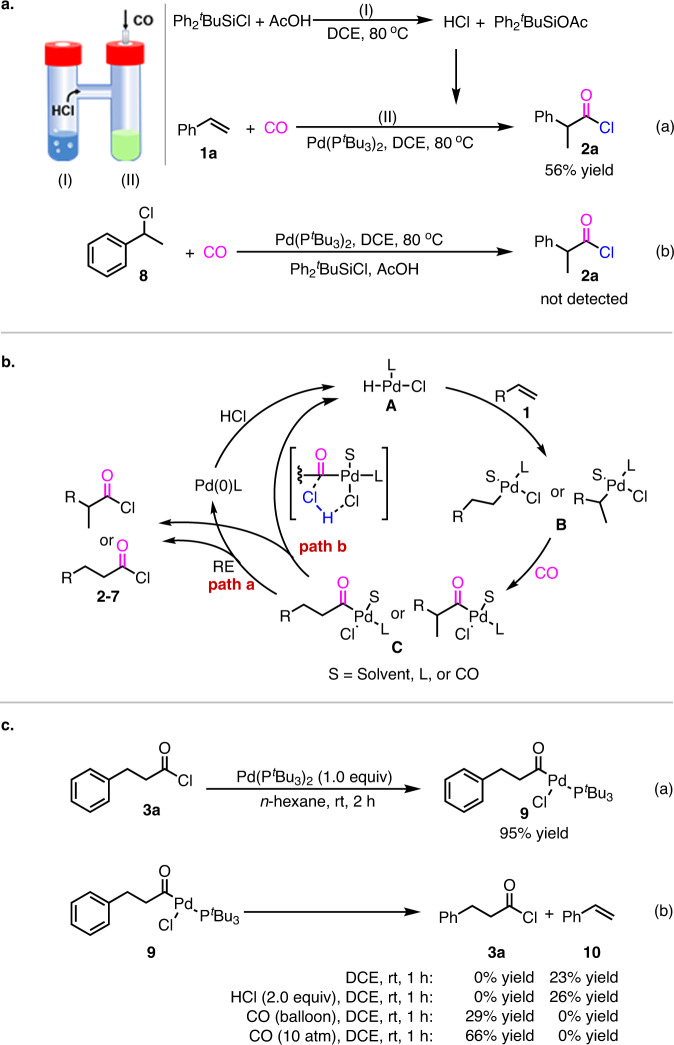
Mechanistic studies. **a** Two chamber experiments. **b** Tentative mechanisms of the reaction. **c** Control experiment.

To gain mechanistic insight into the reaction, control experiments were performed. When the reaction was operated in a two-chamber system with the ex situ generation of HCl from tert-butylchlorodiphenylsilane and AcOH, a 56% yield of acid chloride **2a** was obtained (Fig. 5a (a)). This result suggested that tert-butylchlorodiphenylsilane combined with AcOH play a role as mild HCl-releasing reagents. To check if the reaction started from a palladium-hydride insertion pathway or a hydrochlorination pathway, (1-chloroethyl)benzene **8** was used as the substrate in the reaction (Fig. 5a (b)). The result of no reaction in this case indicated that the hydrochlorination of alkene was excluded from our hydrochlorocarbonylation.

As a result, tentative mechanisms that proceeded through palladium hydride pathway were proposed in Fig. 5b. Initially, coordination and insertion of alkene **1** into palladium hydride **A** affords the alkyl-Pd-Cl intermediate **B**. The alkyl-Pd-Cl **B** undergoes CO coordination and insertion to generate the acyl-Pd-Cl intermediate **C**. Reductive elimination of acyl-Pd-Cl species **C** produces the desired acid chloride and palladium catalyst Pd(0)L. Finally, oxidative addition of Pd(0)L into HCl forms the palladium hydride catalyst **A** (path a). Alternatively, the catalytic cycle might be directly completed to form the acid chloride and palladium hydride **A** through chlorolysis of acyl-Pd-Cl intermediate **C** by HCl (path b). In both catalytic cycles, the regiocontrol were dependent on the ligand and solvent.

To identify above two catalytic cycles, further studies have been conducted. First, the acyl-palladium complex **9**, a stable and yellow precipitate^19^, was prepared by the reaction of **3a** and stoichiometric Pd(P^*t*^Bu_3_)_2_ in *n*-hexane (Fig. 5c (a)). Stirring the acyl-palladium **9** in DCE solvent at room temperature in 1 h, 23% yield of styrene **10** was formed (Fig. 5c (b)). Similar result was observed under 2.0 equiv of HCl. However, when the reaction was operated under balloon pressure of CO, the acid chloride **3a** was obtained in 29% yield. And the yield of acid chloride **3a** was enhanced to 66% under high pressure of CO^51^. These observations suggested that: (1) the pathway of chlorolysis of acyl-Pd-Cl intermediate **C** to form acid chloride (Fig. 5b, path b) is less likely; (2) CO prompted reductive elimination of acyl-palladium species **9** to afford acid chloride is reasonable (Fig. 5b, path a); and (3) both the step of insertion of palladium hydride **A** into alkenes and insertion of CO into alkyl-Pd intermediate **B** are irreversible under CO^42,52,53^.

In summary, we have developed a novel and effective palladium-catalyzed hydrochlorocarbonylation of alkenes for the synthesis of alkyl acid chlorides. Depended on the ligands and solvents, both branched and linear alkyl acid chlorides were formed selectively in good to high yields. The reaction employs tert-butylchlorodiphenylsilane and AcOH as mild HCl source and shows good functional group compatibility. Cooperating with follow-up acylation reactions, this Pd-catalyzed hydrochlorocarbonylation offers a complementary platform for the synthesis of diverse carbonyl compounds from readily available alkenes. Mechanistic investigations revealed that the reaction proceeded through a palladium hydride mechanism, and CO prompted reductive elimination of the acyl-Pd-Cl intermediate.

## Methods

### Synthesis of compound 2

In a glove box, to a dry and stirred glass vessel, alkene **1** (0.2 mmol, 1.0 equiv), Pd(P^*t*^Bu_3_)_2_ (0.01 mmol, 5 mol%), and DCE (1.5 mL) were added. To the above mixture, Ph_2_^*t*^BuSiCl (0.3 mmol, 1.5 equiv) and AcOH (0.3 mmol, 1.5 equiv) were added. The glass vessel was then put into an autoclave. The autoclave was evacuated and backfilled with CO for three times in a well-ventilated fume hood and then pressurized to 30 atm of CO. The reaction mixture in autoclave was stirred at 80 °C for 24 h. After the reaction completed, the autoclave was cooled down to room temperature. Then, the CO in autoclave was carefully released in a well-ventilated fume hood. Take out the glass vessel, and MeOH (1.0 mL) was added. The mixture in glass vessel was stirred for 30 mins at room temperature. The residue was purified by column chromatography on silica gel to afford the corresponding methyl carboxylate by flash column chromatography on silica gel.

### Synthesis of compound 3

In a glove box, to a dry and stirred glass vessel, alkene **1** (0.2 mmol, 1.0 equiv), PdCl_2_ (0.01 mmol, 5 mol%), Xantphos (0.012 mmol, 6 mol%) and PhCl (1.5 mL) were added. To the above mixture, Ph_2_^*t*^BuSiCl (0.3 mmol, 1.5 equiv) and AcOH (0.3 mmol, 1.5 equiv) were added. The glass vessel was then put into an autoclave. The autoclave was evacuated and backfilled with CO for three times in a well-ventilated fume hood and then pressurized to 40 atm of CO. The reaction mixture in autoclave was stirred at 100 °C for 24 h. After the reaction completed, the autoclave was cooled down to room temperature. Then, the CO in autoclave was carefully released in a well-ventilated fume hood. Take out the glass vessel, and MeOH (1.0 mL) was added. The mixture in glass vessel was stirred for 30 mins at room temperature. The residue was purified by column chromatography on silica gel to afford the corresponding methyl carboxylate by flash column chromatography on silica gel.

## Supplementary information

Supplementary Information

## Data Availability

The authors declare that all relevant data supporting the findings of this study are available either within the manuscript itself and/or in the Supplementary Information. Experimental details and characterization of products are provided in the Supplementary Information. All other data are available from the corresponding author upon request.
