# Supplementary material for: Palladium-catalyzed regiodivergent hydrochlorocarbonylation of alkenes for formation of acid chlorides
Source: Nat Commun. 2023 May 31;14:3167. doi: 10.1038/s41467-023-38748-3 (PMC10232548; doi:10.1038/s41467-023-38748-3)

Supplementary Information

Palladium-catalyzed regiodivergent hydrochlorocarbonylation of alkenes for formation of acid chlorides

Fei Wu[1,2], Bo Wang[1,2], Na-Qi Li[1], Hui-Yi Yang[1], Zhi-Hui Ren[1] and Zheng-Hui Guan[1,*]

[1] Key Laboratory of Synthetic and Nature Functional Molecule of the Ministry Education, Department of Chemistry & Materials Science, Northwest University, Xi'an 710127, P. R. China

[2] These authors contributed equally to this work.

* guanzhh@nwu.edu.cn

CONTENT

1. **General Information ---S2**

2. **Supplementary Methods and Discussions --S4**

   2.1. Preparation of Aromatic and Aliphatic Alkenes **-----------------------------------S4**

   2.2. Optimization of the Reaction Conditions **--S5**

   2.3. Typical Procedure for the Reaction **--S6**

   2.4. Mechanistic Studies **---S12**

   2.5. Characterization Data of Products **---S14**

   2.6. Copies of ^{1}H and ^{13}C NMR Spectra **---S47**

1. General Information.

Chemicals

Chemicals were commercially purchased from Adamas-beta, Energy Chemical, Aladdin, etc. and directly used without further purification unless otherwise stated. $PdCl_2$, $PdBr_2$, PdI_2, $Pd(P^tBu_3)_2$, Xantphos, and DPEphos, etc. were purchased from Adamas-beta; *tert*-butylchlorodiphenylsilane, aldehyde, alkenes, triphenylsilyl chloride, etc. were purchased from Energy Chemical.

Chromatography

Analytical thin-layer chromatography (TLC) was carried out with silica gel pre-coated glass plates (TLC-Silica gel GF254, coating thickness: 0.20-0.25 mm, particle size: 10-40 μm) purchased from Xinnuo Chemical (Yantai, China). The TLC was visualized with a UV lamp (254 or 365 nm).

Flash Column chromatography was carried out on silica gel (60 Å, 200-300 mesh) purchased from Xinnuo Chemicals (Yantai, China) with technical grade solvents as the eluent. All the yields referred to spectroscopically and chromatographically pure compounds.

Nuclear Magnetic Resonance (NMR) Spectroscopy

^1H NMR spectra were recorded on Bruker AVANCE III-400 instrument (400 MHz spectrometer). The analytical sample was dissolved in an appropriate deuterated solvent. The employed deuterated solvent and the measuring frequency are indicated in each ^1H NMR data. Chemical shifts are reported in parts per million (ppm) with the solvent resonance as the internal reference ($CDCl_3$ δ 7.26). The following abbreviations (or combinations thereof) were used to explain multiplicities: s = singlet, d = doublet, t = triplet, q = quartet, m = multiplet, b = broad. Coupling constants, *J* were reported in Hertz unit (Hz).

^{13}C NMR spectra were recorded on Bruker AVANCE III-400 instrument (101 MHz spectrometer). The employed deuterated solvent and the measuring frequency are both indicated in each ^{13}C NMR data. Chemical shifts are reported in ppm with the solvent resonance as the internal reference ($CDCl_3$ δ 77.16).

High Resolution Mass Spectrometry (HRMS)

HRMS were recorded on a liquid chromatography/quadrupole time-of-flight mass spectrometer (MicroTof-Q II mass spectrometer, Bruker Daltonics) using electrospray ionization-time of flight (ESI-TOF) at the Instrumental Analysis Center of Northwest University. The calculated values are based on the most abundant isotope.

2. Supplementary Methods and Discussions.

2.1 Preparation of Aromatic and Aliphatic Alkenes.

Method A:

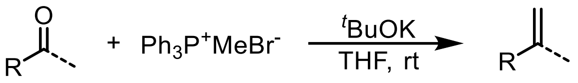

Supplementary Fig. 1. Preparation of Aromatic and Aliphatic Alkenes: Method A.

To a 25 ml round bottomed flask were added methyl triphenylphosphonium bromide (7.5 mmol, 1.5 equiv) and ^{t}BuOK (7.5 mmol, 1.5 equiv). Adding 10 mL of dry THF, the mixture was stirred at room temperature for 1.0 hour. After that diluted ketones or aldehydes (5.0 mmol, 1.0 equiv) in dry THF (2 mL) was added, then the reaction was stirred at room temperature for overnight. The mixture was diluted with CH_2Cl_2 (25 mL), washed with brine (3 x 15 mL), dried with Na_2SO_4, filtered and concentrated in vacuo. The crude material was purified by column chromatography (hexanes as the eluent) to afford alkenes.

Method B:

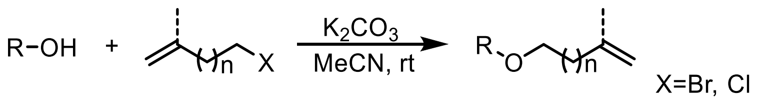

Supplementary Fig. 2. Preparation of Aromatic and Aliphatic Alkenes: Method B.

To a 25 ml round bottomed flask were added alcohol (5.0 mmol, 1.0 equiv) and K_2CO_3 (7.5 mmol, 1.5 equiv). Adding 10 mL of MeCN, the mixture was stirred at room temperature for 10 mins. After that diluted R-CH_2-X (5.0 mmol, 1.0 equiv) in MeCN (2 mL) was added, then the reaction was stirred at room temperature for overnight. The mixture was diluted with CH_2Cl_2 (25 mL), washed with brine (3 x 15 mL), dried with Na_2SO_4, filtered and concentrated in vacuo. The crude material was purified by column chromatography (hexanes as the eluent) to afford alkenes.

2.2.Optimization of the Reaction Condition.

Supplementary Table 1. Screening of Reaction Conditions[a]

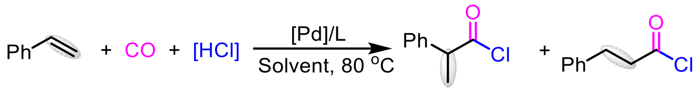

entry	[Pd]	ligand	[HCl]	solvent	yield (%) **2a+3a**	ratio of **2a/3a**
1	$Pd(PPh_3)_2Cl_2$	--	HCl	DCE	15%	76:24
2	$Pd(PPh_3)_2Cl_2$	--	CH_3COCl+EtOH	DCE	3%	ND
3	$Pd(PPh_3)_2Cl_2$	--	TMSCl+EtOH	DCE	5%	ND
4	$Pd(PPh_3)_2Cl_2$	--	Ph_3SiCl+EtOH	DCE	17%	75:25
5	$Pd(PPh_3)_2Cl_2$	--	$Ph_2{}^tBuSiCl$+EtOH	DCE	22%	73:27
6	$Pd(PPh_3)_2Cl_2$	--	$Ph_2{}^tBuSiCl$+AcOH	DCE	39%	73:27
7	$PdCl_2$	$P(2\text{-}MeOPh)_3$	$Ph_2{}^tBuSiCl$+AcOH	DCE	34%	88:12
8	$PdCl_2$	$PAd_2{}^nBu$	$Ph_2{}^tBuSiCl$+AcOH	DCE	30%	>99:1
9	$PdCl_2$	P^tBuPh_2	$Ph_2{}^tBuSiCl$+AcOH	DCE	56%	90:10
10	$PdCl_2$	P^tBu_3	$Ph_2{}^tBuSiCl$+AcOH	DCE	90%	97:3
11	$Pd(P^tBu_3)_2$	--	$Ph_2{}^tBuSiCl$+AcOH	DCE	96%	>99:1
12	$PdCl_2$	Dtbpx	$Ph_2{}^tBuSiCl$+AcOH	DCE	NR	ND
13	$PdCl_2$	DPE-Phos	$Ph_2{}^tBuSiCl$+AcOH	DCE	73%	33:67
14	$PdCl_2$	Xantphos	$Ph_2{}^tBuSiCl$+AcOH	DCE	67%	28:72
15	$PdCl_2$	^{t}Bu-Xantphos	$Ph_2{}^tBuSiCl$+AcOH	DCE	42%	49:51
16	$PdCl_2$	Xantphos	$Ph_2{}^tBuSiCl$+AcOH	THF	86%	37:63
17	$PdCl_2$	Xantphos	$Ph_2{}^tBuSiCl$+AcOH	Toluene	75%	10:90
18	$PdCl_2$	Xantphos	$Ph_2{}^tBuSiCl$+AcOH	PhCl	64%	4:96
19[b]	$PdCl_2$	Xantphos	$Ph_2{}^tBuSiCl$+AcOH	PhCl	95%	5:95

[a]Conditions: styrene **1a** (0.2 mmol), [Pd] (5 mol%), bisphosphine ligand (6 mol%) or monophosphine ligand (10 mol%), [SiCl] (0.3 mmol), EtOH or AcOH (0.3 mmol), solvent (1.5 mL), CO (30 atm), 80 °C for 24 h. The yields of **2a+3a** and ratio of regioisomers **2a/3a** were determined by GC-MS analysis of their derivatized methyl carboxylates using *n*-hexadecane as the internal standard (see SI). [b]CO (40 atm), 100 °C for 24 h. ^{t}Bu: tert-butyl.

2.3. Typical Procedure for the Reaction.

2.3.1. Reaction Conditions and Results for Figure 2a in maintext:

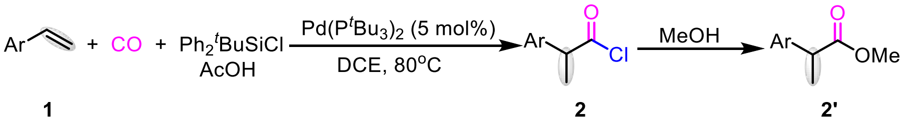

Supplementary Fig. 3. Reaction Conditions and Results for Figure 2a in maintext.

In a glove box, to a dry and stirred glass vessel, alkene **1** (0.2 mmol, 1.0 equiv), $Pd(P^tBu_3)_2$ (0.01 mmol, 5 mol%), and DCE (1.5 mL) were added. To the above mixture, $Ph_2{}^tBuSiCl$ (0.3 mmol, 1.5 equiv) and AcOH (0.3 mmol, 1.5 equiv) were added. The glass vessel was then put into an autoclave. The autoclave was evacuated and backfilled with CO for three times in a well-ventilated fume hood and then pressurized to 30 atm of CO. The reaction mixture in autoclave was stirred at 80 ^oC for 24 h. After the reaction completed, the autoclave was cooled down to room temperature. Then, the CO in autoclave was carefully released in a well-ventilated fume hood.

Take out the glass vessel, and MeOH (1.0 mL) was added. The mixture in glass vessel was stirred for 30 mins at room temperature. The regioselectivity listed in Figure 2a (within parentheses) in maintext was directly measured by GC-MS analysis of these crude products. The yields listed in Figure 2a in maintext were based on the isolated yields of methyl carboxylate of acid chlorides **2'** by flash column chromatography on silica gel.

2.3.2. Reaction Conditions and Results for Figure 2b in maintext:

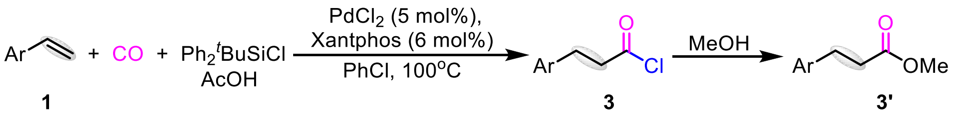

Supplementary Fig. 4. Reaction Conditions and Results for Figure 2b in maintext.

In a glove box, to a dry and stirred glass vessel, alkene **1** (0.2 mmol, 1.0 equiv), $PdCl_2$ (0.01 mmol, 5 mol%), Xantphos (0.012 mmol, 6 mol%) and PhCl (1.5 mL) were added. To the above mixture, $Ph_2{}^tBuSiCl$ (0.3 mmol, 1.5 equiv) and AcOH (0.3 mmol, 1.5 equiv) were added. The glass vessel was then put into an autoclave. The autoclave

was evacuated and backfilled with CO for three times in a well-ventilated fume hood and then pressurized to 40 atm of CO. The reaction mixture in autoclave was stirred at 100 °C for 24 h. After the reaction completed, the autoclave was cooled down to room temperature. Then, the CO in autoclave was carefully released in a well-ventilated fume hood.

Take out the glass vessel, and MeOH (1.0 mL) was added. The mixture in glass vessel was stirred for 30 mins at room temperature. The regioselectivity listed in Figure 2b (within parentheses) in maintext was directly measured by GC-MS analysis of these crude products. The yields listed in Figure 2b in maintext were based on the isolated yields of methyl carboxylate of acid chlorides **3'** by flash column chromatography on silica gel.

2.3.3. Reaction Conditions and Results for Figure 3 in maintext:

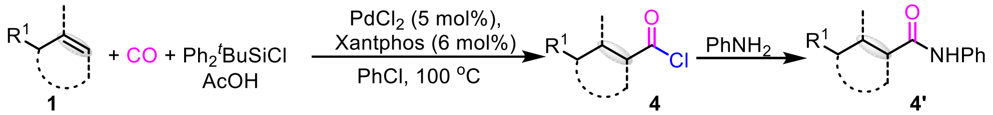

Supplementary Fig. 5. Reaction Conditions and Results for Figure 3 in maintext.

In a glove box, to a dry and stirred glass vessel, alkene **1** (0.2 mmol, 1.0 equiv), $PdCl_2$ (0.01 mmol, 5 mol%), Xantphos (0.012 mmol, 6 mol%) and PhCl (1.5 mL) were added. To the above mixture, $Ph_2{}^tBuSiCl$ (0.3 mmol, 1.5 equiv) and AcOH (0.3 mmol, 1.5 equiv) were added. The glass vessel was then put into an autoclave. The autoclave was evacuated and backfilled with CO for three times in a well-ventilated fume hood and then pressurized to 40 atm of CO. The reaction mixture in autoclave was stirred at 100 °C for 24 h. After the reaction completed, the autoclave was cooled down to room temperature. Then, the CO in autoclave was carefully released in a well-ventilated fume hood.

Take out the glass vessel, and MeOH (1.0 mL) was added. The mixture in glass vessel was stirred for 30 mins at room temperature. The regioselectivity listed in Figure 3 (within parentheses) in maintext was directly measured by GC-MS analysis of these crude products. For **4e**, **4m, 4x** listed in Figure 3 in maintext, their yields were based

on the isolated yields of methyl carboxylate of acid chlorides by flash column chromatography on silica gel.

For the alkyl alkenes, after hydrochlorocarbonylation, taking out the glass vessel, and then $PhNH_2$ (0.3 mmol, 1.5 equiv), DMAP (0.01mmol, 5 mol%) and NEt_3 (0.4 mmol, 2.0 equiv) were added. The mixture in glass vessel was stirred at 80 °C for 3 hours. The yields listed in Figure 3 in maintext were based on the isolated yields of amide of acid chlorides **4'** by flash column chromatography on silica gel.

2.3.4. Reaction Conditions and Results for One-pot Hydrochlorocarbonylation-Esterification, Amidation and Thioesterification in Figure 4 in maintext:

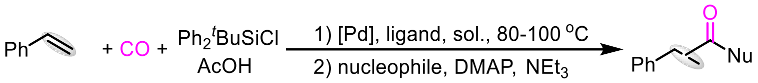

5, 6

Supplementary Fig. 6. Reaction Conditions and Results for One-pot Hydrochloro-carbonylation-Esterification, Amidation and Thioesterification in Figure 4 in maintext.

The hydrochlorocarbonylations of styrene for the formation of the corresponding branched or linear acid chlorides were carried out under the standard conditions as described above in **2.3.1** and **2.3.2**.

After completed the hydrochlorocarbonylation, the glass vessel was taken out from autoclave. To the mixture of glass vessel, the solution of NuH (3.0 M in DCM, 0.3 mmol, 1.5 equiv), DMAP (0.01mmol, 0.05 equiv) and NEt_3 (0.4 mmol, 2.0 equiv) was added and stirred overnight at 80 °C. The reaction mixture was cooled down to room temperature and concentrated under reduced pressure. The corresponding esters and amides **5a-5l, 6a-6l** were obtained by purified above residue by flash column chromatography on silica gel.

2.3.5. Reaction Conditions and Results for One-pot Hydrochlorocarbonylation and C-C Bond Coupling with Ethylmagnesium Chloride in Figure 4 in maintext:

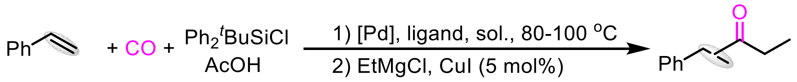

5m, 6m

Supplementary Fig. 7. Reaction Conditions and Results for One-pot Hydrochloro-carbonylation and C-C Bond Coupling with Ethylmagnesium Chloride in Figure 4 in maintext.

The hydrochlorocarbonylations of styrene for the formation of the corresponding branched or linear acid chlorides were carried out under the standard conditions as described above in **2.3.1** and **2.3.2**.

After completed the hydrochlorocarbonylation, the glass vessel was taken out from autoclave. To the mixture of glass vessel, CuI (0.01 mmol, 5 mol%) and ethyl magnesium chloride (2.0 M in THF, 100 μL, 0.2 mmol) was added at -78 ^{o}C under argon. After being stirred at -78 ^{o}C for 5 mins, the reaction was quenched with saturated aqueous NH_4Cl (2.5 mL) and extracted with EtOAc (3 x 5.0 mL). The combined organic layers were dried over anhydrous Na_2SO_4, and was concentrated under reduced pressure. The residue was purified by flash column chromatography on silica gel to give **5m** and **6m**.

2.3.6. Reaction Conditions and Results for One-pot Hydrochlorocarbonylation-Friedel-Crafts Acylation in Figure 4 in maintext:

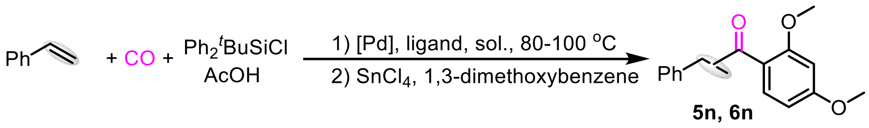

Supplementary Fig. 8. Reaction Conditions and Results for One-pot Hydrochloro-carbonylation-Friedel-Crafts Acylation in Figure 4 in maintext.

The hydrochlorocarbonylations of styrene for the formation of the corresponding branched or linear acid chlorides were carried out under the standard conditions as described above in **2.3.1** and **2.3.2**.

After completed the hydrochlorocarbonylation, the glass vessel was taken out from autoclave. To the mixture of glass vessel, $SnCl_4$ (0.3 mmol, 1.5 equiv) and the solution of 1,3-dimethoxybenzene (3.0 M in DCM, 0.3 mmol, 1.5 equiv) and was added and stirred overnight at room temperature. The reaction was quenched with H_2O (5 mL)

and extracted with EtOAc (3 x 5.0 mL). The combined organic layers were dried over anhydrous Na_2SO_4, and was concentrated under reduced pressure. The residue was purified by flash column chromatography on silica gel to give **5n** and **6n**.

2.3.7. Reaction Conditions and Results for One-pot Linking Two Biomolecules in Figure 4c in maintext:

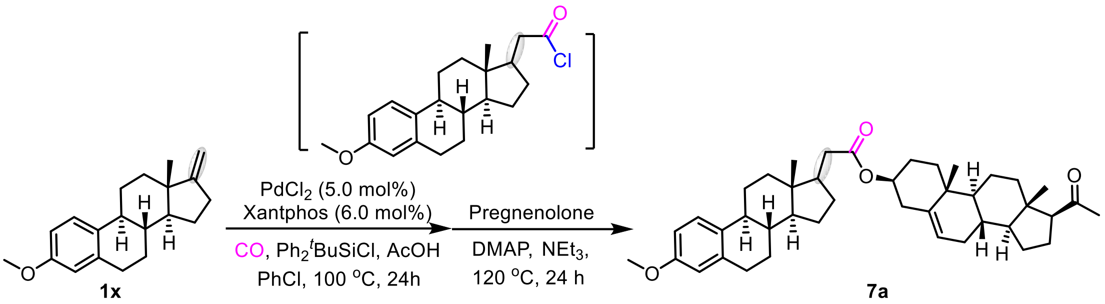

Supplementary Fig. 9. Reaction Conditions and Results for One-pot Linking Two Biomolecules **7a**.

The hydrochlorocarbonylations of **1x** for the formation of the corresponding acid chloride was carried out under the standard conditions as described above in **2.3.2**.

After completed the hydrochlorocarbonylation, the glass vessel was taken out from autoclave. To the mixture of glass vessel, the pregnenolone (3.0 M in DCM, 0.3 mmol, 1.5 equiv), DMAP (0.01mmol, 0.05 equiv) and NEt_3 (0.4 mmol, 2.0 equiv) was added and stirred at 120 °C for 24 h. The reaction mixture was cooled down to room temperature and concentrated under reduced pressure. The corresponding ester **7a** was obtained by purified above residue by flash column chromatography on silica gel.

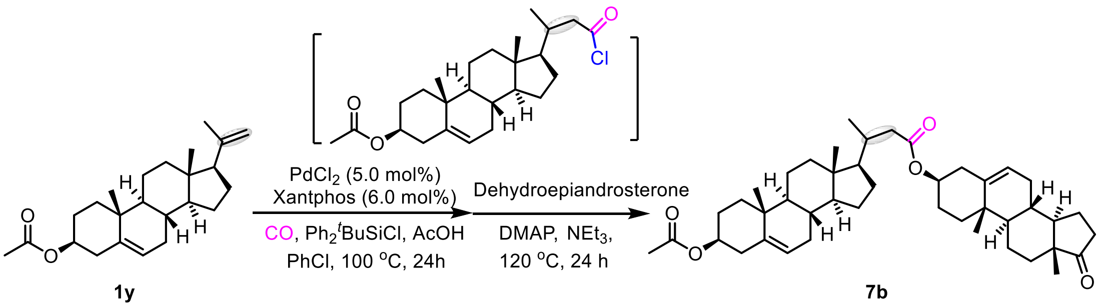

Supplementary Fig. 10. Reaction Conditions and Results for One-pot Linking Two Biomolecules **7b**.

The hydrochlorocarbonylations of **1y** for the formation of the corresponding acid chloride was carried out under the standard conditions as described above in **2.3.2**.

After completed the hydrochlorocarbonylation, the glass vessel was taken out from autoclave. To the mixture of glass vessel, the dehydroepiandrosterone (3.0 M in DCM, 0.3 mmol, 1.5 equiv), DMAP (0.01mmol, 0.05 equiv) and NEt_3 (0.4 mmol, 2.0 equiv) was added and stirred at 120 °C for 24 h. The reaction mixture was cooled down to room temperature and concentrated under reduced pressure. The ester **7b** was obtained by purified above residue by flash column chromatography on silica gel.

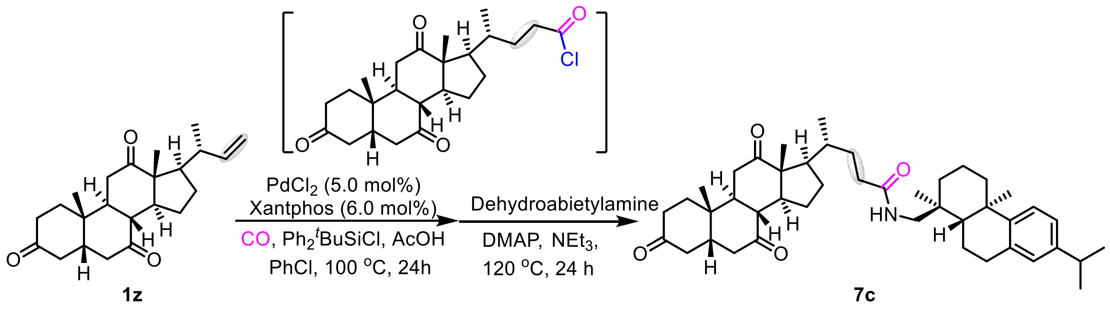

Supplementary Fig. 11. Reaction Conditions and Results for One-pot Linking Two Biomolecules **7c**.

The hydrochlorocarbonylations of **1z** for the formation of the corresponding acid chloride was carried out under the standard conditions as described above in **2.3.2**.

After completed the hydrochlorocarbonylation, the glass vessel was taken out from autoclave. To the mixture of glass vessel, the dehydroabietylamine (3.0 M in DCM, 0.3 mmol, 1.5 equiv), DMAP (0.01mmol, 0.05 equiv) and NEt_3 (0.4 mmol, 2.0 equiv) was added and stirred at 120 °C for 24 h. The reaction mixture was cooled down to room temperature and concentrated under reduced pressure. The amide **7c** was obtained by purified above residue by flash column chromatography on silica gel.

2.4.Mechanistic Studies.

2.4.1. The Experiments of Two Chamber Reaction.

Two Chamber Reaction:

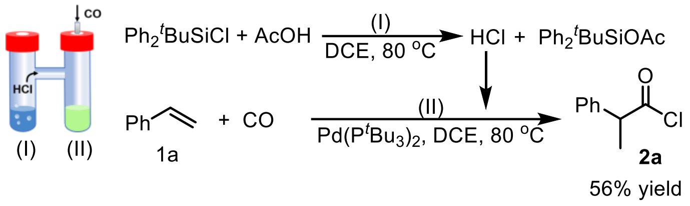

Supplementary Fig. 12. Two Chamber Reaction.

In a glove box, to chamber I of a two-chamber reactor was added $Ph_2{}^tBuSiCl$ (0.6 mmol, 3 equiv.), AcOH (0.6 mmol, 3 equiv.), and DCE (5 mL). To chamber II of a two-chamber reactor was added with styrene **1a** (0.2 mmol, 1.0 equiv), $Pd(P^tBu_3)_2$ (0.01 mmol, 5 mol%), DCE (1.5 mL). The two-chamber reactor was put into an autoclave. The autoclave was evacuated and backfilled with CO for three times in a well-ventilated fume hood and then pressurized to 30 atm of CO. The reaction mixture in autoclave was stirred at 80 °C for 24 h. After the reaction completed, the autoclave was cooled down to room temperature. Then, the CO in autoclave was carefully released in a well-ventilated fume hood.

Take out the glass vessel, and MeOH (1.0 mL) was added. The mixture in glass vessel was stirred for 30 mins at room temperature. The yield of methyl 2-phenylpropanoate **2a'** (56% yield) was determined by GC-MS using *n*-hexadecane as the internal standard.

2.4.2. The Control Experiments by (1-chloroethyl)benzene as the Substrate.

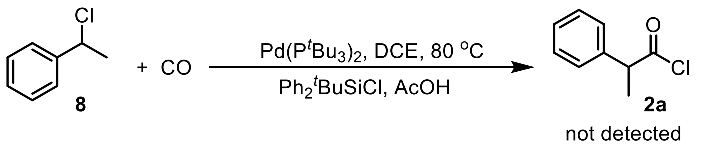

Supplementary Fig. 13. The Control Experiments by (1-chloroethyl)benzene as the Substrate.

In a glove box, to a dry and stirred glass vessel, (1-chloroethyl)benzene **8** (0.2 mmol, 1.0 equiv), Pd(P^tBu_3)$_2$ (0.01 mmol, 5 mol%), and DCE (1.5 mL) were added. To the above mixture, $Ph_2{}^tBuSiCl$ (0.3 mmol, 1.5 equiv) and AcOH (0.3 mmol, 1.5 equiv) were added. The glass vessel was then put into an autoclave. The autoclave was evacuated and backfilled with CO for three times in a well-ventilated fume hood and then pressurized to 30 atm of CO. The reaction mixture in autoclave was stirred at 80 ^oC for 24 h. After the reaction completed, the autoclave was cooled down to room temperature. Then, the CO in autoclave was carefully released in a well-ventilated fume hood.

Take out the glass vessel, and MeOH (1.0 mL) was added. The mixture in glass vessel was stirred for 30 mins at room temperature. The reaction mixture was directly measured by GC-MS analysis of the crude products. Only the starting material (1-chloroethyl)benzene **8** was observed, no any methyl 2-phenylpropanoate was observed.

2.5. Characterization Data of Products.

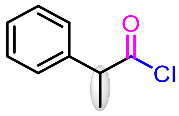

2a

2-phenylpropenyl chloride (2a)

Crude product was detected by NMR, ^{1}H NMR (400 MHz, $CDCl_3$) δ 7.39-7.31 (m, 3H), 7.29-7.24 (m, 2H), 4.14-4.08 (m, 1H), 1.59-1.57 (m, 3H); ^{13}C NMR (101 MHz, $CDCl_3$) δ 175.6, 129.2, 128.3, 128.0, 127.7, 57.5, 18.8.

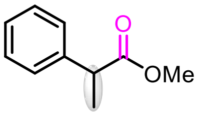

2a'

Methyl-2-phenylpropanoate (2a')

Yield = 94%, b/l > 99:1, ^{1}H NMR (400 MHz, $CDCl_3$) δ 7.34-7.28 (m, 4H), 7.26-7.22 (m, 1H), 3.72 (q, J = 7.2 Hz, 1H), 3.64 (s, 3H), 1.50 (d, J = 7.6 Hz, 3H); ^{13}C NMR (101 MHz, $CDCl_3$) δ 175.1, 140.6, 128.7, 127.6, 127.2, 52.1, 45.5, 18.7.

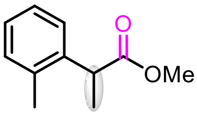

2b'

Methyl-2-(*o*-tolyl)propanoate (2b')

Yield = 65%, b/l > 99:1, ^{1}H NMR (400 MHz, $CDCl_3$) δ 7.26-7.24 (m, 1H), 7.21-7.13 (m, 3H), 3.96 (q, J = 7.2 Hz, 1H), 3.65 (s, 3H), 2.37 (s, 3H), 1.47 (d, J = 7.2 Hz, 3H); ^{13}C NMR (101 MHz, $CDCl_3$) δ 175.4, 139.2, 135.8, 130.6, 127.1, 126.6, 126.5, 52.1, 41.4, 19.7, 18.1.

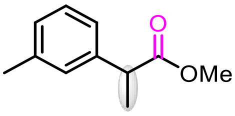

2c'

Methyl-2-(*m*-tolyl)propanoate (2c')

Yield = 80%, b/l > 99:1, ^{1}H NMR (400 MHz, $CDCl_3$) δ 7.22-7.19 (m, 1H), 7.10-7.05 (m, 3H), 3.68 (q, *J* = 7.2 Hz, 1H), 3.65 (s, 3H), 2.34 (s, 3H), 1.48 (d, *J* = 6.8 Hz, 3H); ^{13}C NMR (101 MHz, $CDCl_3$) δ 175.2, 140.6, 138.4, 128.6, 128.3, 128.0, 124.6, 52.1, 45.4, 21.5, 18.7.

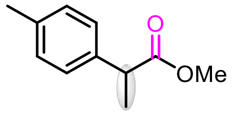

2d'

Methyl-2-(*p*-tolyl)propanoate (2d')

Yield = 90%, b/l > 99:1, ^{1}H NMR (400 MHz, $CDCl_3$) δ 7.18 (d, *J* = 8.4 Hz, 2H), 7.12 (d, *J* = 8.0 Hz, 2H), 3.68 (q, *J* = 7.2 Hz, 1H), 3.64 (s, 3H), 2.32 (s, 3H), 1.48 (d, *J* = 7.2 Hz, 3H); ^{13}C NMR (101 MHz, $CDCl_3$) δ 175.3, 137.7, 136.9, 129.4, 127.4, 52.1, 45.1, 22.1, 18.7.

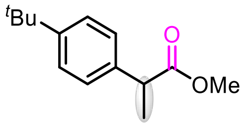

2e'

Methyl-2-(4-(*tert*-butyl)phenyl)propanoate (2e')

Yield = 93%, b/l = 93:7, ^{1}H NMR (400 MHz, $CDCl_3$) δ 7.33 (d, *J* = 8.8 Hz, 2H), 7.22 (d, *J* = 8.4 Hz, 2H), 3.70 (q, *J* = 7.2 Hz, 1H), 3.64 (s, 3H), 1.48 (d, *J* = 7.6 Hz, 3H), 1.30 (s, 9H); ^{13}C NMR (101 MHz, $CDCl_3$) δ 175.3, 150.0, 137.5, 127.2, 125.6, 52.0, 45.0, 34.5, 31.4, 18.7.

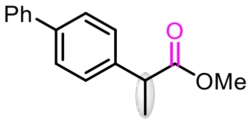

2f'

Methyl-2-([1,1'-biphenyl]-4-yl)propanoate (2f')

Yield = 95%, b/l = 98:2, ^{1}H NMR (400 MHz, $CDCl_3$) δ 7.58-7.54 (m, 4H), 7.44-7.40 (m, 2H), 7.38-7.31 (m, 3H), 3.77 (q, *J* = 7.2 Hz, 1H), 3.68 (s, 3H), 1.54 (q, *J* = 7.2 Hz, 3H); ^{13}C NMR (101 MHz, $CDCl_3$) δ 175.1, 140.9, 140.3, 139.7, 128.9, 128.0, 127.5, 127.4, 127.2, 52.2, 45.2, 18.7.

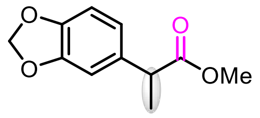

2g'

Methyl-2-(benzo[d][1,3]dioxol-5-yl)propanoate (2g')

Yield = 96%, b/l = 96:4, ^{1}H NMR (400 MHz, $CDCl_3$) δ 6.81 (s, 1H), 6.75 (s, 2H), 5.93 (s, 2H), 3.67-3.62 (m, 4H), 1.46 (d, *J* = 7.2 Hz, 3H); ^{13}C NMR (101 MHz, $CDCl_3$) δ 175.2, 147.9, 146.8, 134.4, 120.8, 108.4, 108.0, 101.2, 52.2, 45.1, 18.8.

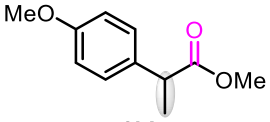

2h'

Methyl-2-(4-methoxyphenyl)propanoate (2h')

Yield = 95%, b/l > 99:1, ^{1}H NMR (400 MHz, $CDCl_3$) δ 7.21 (d, *J* = 8.4 Hz, 2H), 6.85 (d, *J* = 8.4 Hz, 2H), 3.77 (s, 3H), 3.70-3.64 (m, 4H), 1.47 (d, *J* = 7.2 Hz, 3H); ^{13}C NMR (101 MHz, $CDCl_3$) δ 175.4, 158.8, 132.7, 128.5, 114.1, 55.3, 52.0, 44.6, 18.7.

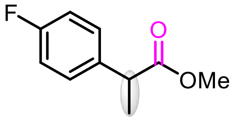

2i'

Methyl-2-(4-fluorophenyl)propanoate (2i')

Yield = 80%, b/l = 94:6, ^{1}H NMR (400 MHz, $CDCl_3$) δ 7.28-7.25 (m, 2H), 7.03-6.98 (m, 2H), 3.71 (q, *J* = 7.2 Hz, 1H), 3.66 (s, 3H), 1.48 (d, *J* = 8.0 Hz, 3H); ^{13}C NMR (101 MHz, $CDCl_3$) δ 175.0, 162.1 (d, *J* = 246.2 Hz), 136.4 (d, *J* = 3.1 Hz), 129.2 (d, *J* = 8.0 Hz), 115.5 (d, *J* = 21.3 Hz), 52.2, 44.7, 18.8.

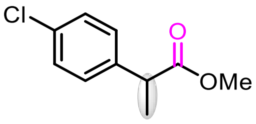

2j'

Methyl-2-(4-chlorophenyl)propanoate (2j')

Yield = 83%, b/l = 98:2, ^{1}H NMR (400 MHz, $CDCl_3$) δ 7.31-7.27 (m, 2H), 7.25-7.22 (m, 2H), 3.70 (q, J = 7.2 Hz, 1H), 3.66 (s, 3H), 1.48 (d, J = 8.0 Hz, 3H); ^{13}C NMR (101 MHz, $CDCl_3$) δ 174.7, 139.1, 133.1, 129.0, 128.9, 52.2, 44.9, 18.6.

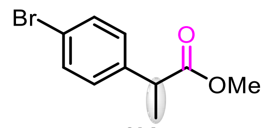

2k'

Methyl-2-(4-bromophenyl)propanoate (2k')

Yield = 87%, b/l = 97:3, ^{1}H NMR (400 MHz, $CDCl_3$) δ 7.44 (d, J = 8.8 Hz, 2H), 7.17 (d, J = 8.0 Hz, 2H), 3.71-3.65 (m, 4H), 1.48 (d, J = 7.2 Hz, 3H); ^{13}C NMR (101 MHz, $CDCl_3$) δ 174.6, 139.6, 131.8, 129.4, 121.2, 52.2, 45.0, 18.6.

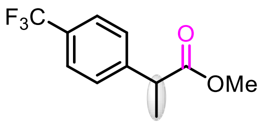

2l'

Methyl-2-(4-(trifluoromethyl)phenyl)propanoate (2l')

Yield = 82%, b/l > 99:1, ^{1}H NMR (400 MHz, $CDCl_3$) δ 7.59 (d, J = 8.0 Hz, 2H), 7.42 (d, J = 8.0 Hz, 2H), 3.79 (q, J = 7.2 Hz, 1H), 3.68 (s, 3H), 1.52 (d, J = 7.2 Hz, 3H); ^{13}C NMR (101 MHz, $CDCl_3$) δ 174.4, 144.5, 128.1, 125.7 (q, J = 3.8 Hz), 122.9, 52.4, 45.4, 18.6.

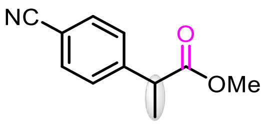

2m'

Methyl-2-(4-cyanophenyl)propanoate (2m')

Yield = 81%, b/l = 97:3, ^{1}H NMR (400 MHz, $CDCl_3$) δ 7.63 (d, J = 8.4 Hz, 2H), 7.42 (d, J = 7.2 Hz, 2H), 3.79 (q, J = 7.2 Hz, 1H), 3.68 (s, 3H), 1.52 (d, J = 7.2 Hz, 3H); ^{13}C NMR (101 MHz, $CDCl_3$) δ 173.9, 145.8, 132.6, 128.6, 118.8, 111.3, 52.5, 45.6, 18.5.

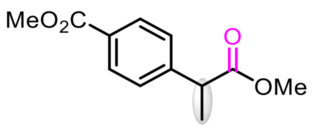

2n'

Methyl-4-(1-methoxy-1-oxopropan-2-yl)benzoate (2n')

Yield = 85%, b/l = 95:5, ^{1}H NMR (400 MHz, $CDCl_3$) δ 8.00 (d, *J* = 8.0 Hz, 2H), 7.37 (d, *J* = 8.0 Hz, 2H), 3.91 (s, 3H), 3.79 (q, *J* = 7.2 Hz, 1H), 3.67 (s, 3H), 1.52 (d, *J* = 6.8 Hz, 3H); ^{13}C NMR (101 MHz, $CDCl_3$) δ 174.4, 166.9, 145.7, 130.0, 129.1, 127.7, 52.3, 52.2, 45.5, 18.5.

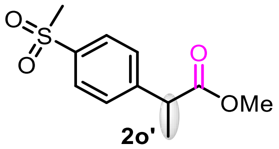

2o'

Methyl-2-(4-(methylsulfonyl)phenyl)propanoate (2o')

Yield = 64%, b/l > 99:1, ^{1}H NMR (400 MHz, $CDCl_3$) δ 7.92-7.90 (m, 2H), 7.53-7.49 (m, 2H), 3.83 (q, *J* = 7.2 Hz, 1H), 3.69 (s, 3H), 3.05 (s, 3H), 1.54 (d, *J* = 7.2 Hz, 3H); ^{13}C NMR (101 MHz, $CDCl_3$) δ 174.0, 146.8, 139.5, 128.8, 127.9, 52.5, 45.5, 44.6, 18.6.

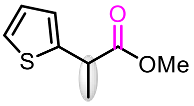

2p'

Methyl-2-(thiophen-2-yl)propanoate (2p')

Yield = 59%, b/l > 99:1, ^{1}H NMR (400 MHz, $CDCl_3$) δ 7.20 (t, *J* = 3.6 Hz, 1H), 6.95 (d, *J* = 3.2 Hz, 2H), 4.02 (q, *J* = 7.2 Hz, 1H), 3.70 (s, 3H), 1.58 (d, *J* = 6.8 Hz, 3H); ^{13}C NMR (101 MHz, $CDCl_3$) δ 174.2, 143.0, 126.8, 124.9, 124.5, 52.4, 40.9, 19.5.

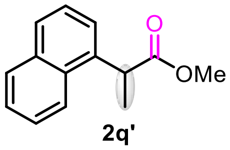

2q'

Methyl-2-(naphthalen-1-yl)propanoate (2q')

Yield = 74%, b/l > 99:1, ¹H NMR (400 MHz, $CDCl_3$) δ 7.15 (d, *J* = 8.8 Hz, 1H), 6.93 (d, *J* = 7.6 Hz, 1H), 6.85-6.82 (m, 1H), 6.63-6.51 (m, 4H), 3.58 (q, *J* = 7.2 Hz, 1H), 2.72 (s, 3H), 0.73 (d, *J* = 7.2 Hz, 3H); ¹³C NMR (101 MHz, $CDCl_3$) δ 175.6, 136.9, 134.1, 131.4, 129.1, 127.9, 126.5, 125.8, 125.7, 124.6, 123.2, 52.3, 41.4, 18.3.

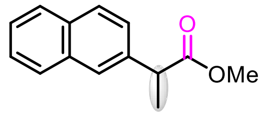

2r'

Methyl-2-(naphthalen-2-yl)propanoate (2r')

Yield = 78%, b/l = 97:3, ¹H NMR (400 MHz, $CDCl_3$) δ 7.79 (d, *J* = 8.4 Hz, 3H), 7.73 (s, 1H), 7.47-7.41 (m, 3H), 3.88 (q, *J* = 7.2 Hz, 1H), 3.85 (s, 3H), 1.58 (d, *J* = 6.8 Hz, 3H); ¹³C NMR (101 MHz, $CDCl_3$) δ 175.1, 138.1, 133.5, 132.7, 128.4, 127.9, 127.7, 126.3, 126.2, 125.9, 125.8, 52.2, 45.6, 18.7.

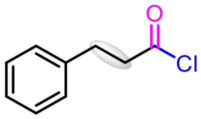

3a

3-phenylpropanoyl chloride (3a)

Crude product was detected by NMR, ¹H NMR (400 MHz, $CDCl_3$) δ 7.33-7.29 (m, 2H), 7.23-7.18 (m, 3H), 3.20 (t, *J* = 7.6 Hz, 2H), 3.00 (t, *J* = 7.6 Hz, 2H); ¹³C NMR (101 MHz, $CDCl_3$) δ 173.2, 138.7, 128.8, 128.4, 126.9, 48.6, 31.1.

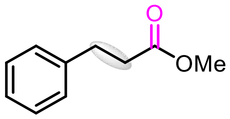

3a'

Methyl-3-phenylpropanoate (3a')

Yield = 95%, l/b = 95:5, ¹H NMR (400 MHz, $CDCl_3$) δ 7.30-7.26 (m, 2H), 7.21-7.18 (m, 3H), 3.66 (s, 3H), 2.95 (t, *J* = 8.0 Hz, 2H), 2.63 (t, *J* = 8.0 Hz, 2H); ¹³C NMR (101 MHz, $CDCl_3$) δ 173.4, 140.6, 128.6, 128.4, 126.4, 51.7, 35.8, 31.0.

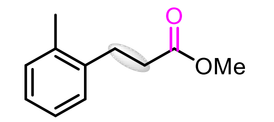

3b'

Methyl-3-(*o*-tolyl)propanoate (3b')

Yield = 90%, l/b = 98:2, ^{1}H NMR (400 MHz, $CDCl_3$) δ 7.13 (s, 4H), 3.68 (s, 3H), 2.94 (t, *J* = 8.0 Hz, 2H), 2.59 (t, *J* = 8.0 Hz, 2H), 2.32 (s, 3H); ^{13}C NMR (101 MHz, $CDCl_3$) δ 173.6, 138.7, 136.1, 130.4, 128.6, 126.5, 126.2, 51.7, 34.5, 28.4, 19.3.

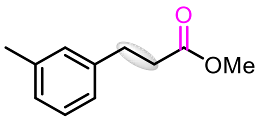

3c'

Methyl-3-(*m*-tolyl)propanoate (3c')

Yield = 92%, l/b = 93:7, ^{1}H NMR (400 MHz, $CDCl_3$) δ 7.18 (t, *J* = 8.0 Hz, 1H), 7.03-6.99 (m, 3H), 3.67 (s, 3H), 2.91 (t, *J* = 8.0 Hz, 2H), 2.62 (t, *J* = 8.0 Hz, 2H), 2.32 (s, 3H); ^{13}C NMR (101 MHz, $CDCl_3$) δ 173.6, 140.6, 138.2, 129.2, 128.5, 127.1, 125.4, 51.7, 35.9, 31.0, 21.5.

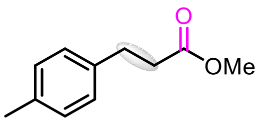

3d'

Methyl-3-(*p*-tolyl)propanoate (3d')

Yield = 94%, l/b = 93:7, ^{1}H NMR (400 MHz, $CDCl_3$) δ 7.09 (s, 4H), 3.67 (s, 3H), 2.91 (t, *J* = 8.0 Hz, 2H), 2.61 (t, *J* = 8.0 Hz, 2H), 2.31 (s, 3H); ^{13}C NMR (101 MHz, $CDCl_3$) δ 173.6, 137.6, 135.9, 129.3, 128.3, 51.7, 36.0, 30.6, 21.1.

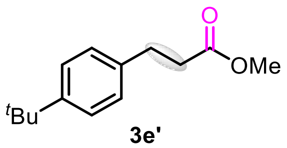

3e'

Methyl-3-(4-(*tert*-butyl)phenyl)propanoate (3e')

Yield = 95%, l/b = 92:8, ^{1}H NMR (400 MHz, $CDCl_3$) δ 7.31 (d, *J* = 8.4 Hz, 2H),

7.13 (d, J = 8.4 Hz, 2H), 3.67 (s, 3H), 2.92 (t, J = 8.0 Hz, 2H), 2.63 (t, J = 8.0 Hz, 2H), 1.30 (s, 9H); ^{13}C NMR (101 MHz, $CDCl_3$) δ 173.5, 149.0, 137.4, 127.9, 125.4, 51.6, 35.4, 31,3, 31,3, 30.3.

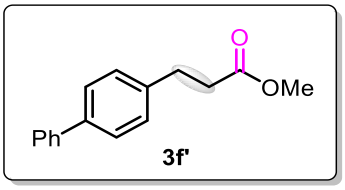

Methyl-3-([1,1'-biphenyl]-4-yl)propanoate (3f')

Yield = 93%, l/b = 97:3, ^{1}H NMR (400 MHz, $CDCl_3$) δ 7.58-7.51 (m, 4H), 7.44-7.40 (m, 2H), 7.34-7.26 (m, 3H), 3.68 (s, 3H), 2.99 (t, J = 8.0 Hz, 2H), 2.67 (t, J = 8.0 Hz, 2H); ^{13}C NMR (101 MHz, $CDCl_3$) δ 173.5, 141.1, 139.7, 139.4, 128.9, 128.8, 127.4, 127.3, 127.1, 51.8, 35.8, 30.7.

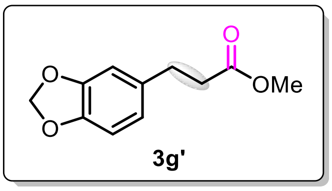

Methyl-3-(benzo[d][1,3]dioxol-5-yl)propanoate (3g')

Yield = 95%, l/b = 90:10, ^{1}H NMR (400 MHz, $CDCl_3$) δ 6.72 (d, J = 7.6 Hz, 1H), 6.68-6.68 (m, 1H), 6.65-6.63 (m, 1H), 5.91 (s, 2H), 3.66 (s, 3H), 2.86 (d, J = 8.0 Hz, 2H), 2.58 (d, J = 8.0 Hz, 2H); ^{13}C NMR (101 MHz, $CDCl_3$) δ 173.3, 147.7, 146.0, 134.4, 121.2, 108.8, 108.3, 100.9, 51.7, 36.1, 30.8.

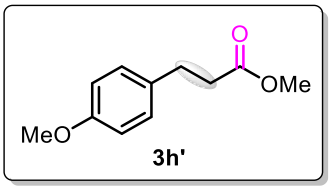

Methyl-3-(4-methoxyphenyl)propanoate (3h')

Yield = 92%, l/b = 91:9, ^{1}H NMR (400 MHz, $CDCl_3$) δ 7.13-7.10 (m, 2H), 6.85-6.80 (m, 2H), 3.78 (s, 3H), 3.66 (s, 3H), 2.89 (t, J = 8.0 Hz, 2H), 2.60 (t, J = 8.0 Hz, 2H); ^{13}C NMR (101 MHz, $CDCl_3$) δ 173.5, 158.2, 132.7, 129.3, 114.0, 55.3, 51.7, 31.6, 30.2.

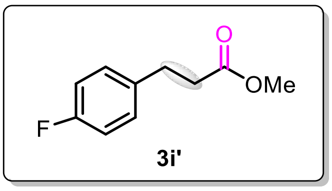

2-methyl-3-(4-fluorophenyl)propanoate (3i')

Yield = 84%, l/b = 93:7, ^{1}H NMR (400 MHz, $CDCl_3$) δ 7.17-7.14 (m, 2H), 6.99-6.95 (m, 2H), 3.66 (s, 3H), 2.92 (t, J = 7.6 Hz, 2H), 2.61 (t, J = 8.0 Hz, 2H); ^{13}C NMR (101 MHz, $CDCl_3$) δ 173.3, 162.2 (d, J = 329.5 Hz), 136.3, 129.9 (d, J = 8.1 Hz), 115.4 (d, J = 21.2 Hz), 51.8, 35.9, 30.3.

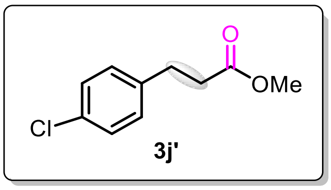

Methyl-3-(4-chlorophenyl)propanoate (3j')

Yield = 87%, l/b = 92:8, ^{1}H NMR (400 MHz, $CDCl_3$) δ 7.25 (d, J = 8.4 Hz, 2H), 7.13 (d, J = 8.8 Hz, 2H), 3.66 (s, 3H), 2.92 (t, J = 8.0 Hz, 2H), 2.61 (t, J = 7.6 Hz, 2H); ^{13}C NMR (101 MHz, $CDCl_3$) δ 173.2, 139.0, 132.1, 129.8, 128.7, 51.8, 35.6, 30.3.

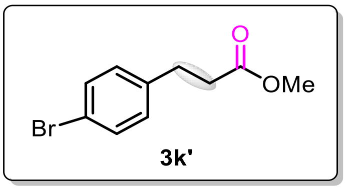

Methyl-3-(4-bromophenyl)propanoate (3k')

Yield = 84%, l/b = 90:10, ^{1}H NMR (400 MHz, $CDCl_3$) δ 7.40 (d, J = 7.2 Hz, 2H), 7.08 (d, J = 7.6 Hz, 2H), 3.66 (s, 3H), 2.92-2.88 (m, 2H), 2.63-2.59 (m, 2H); ^{13}C NMR (101 MHz, $CDCl_3$) δ 173.2, 139.6, 131.7, 130.2, 120.2, 51.8, 35.5, 30.4.

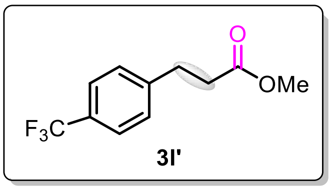

Methyl-3-(4-(trifluoromethyl)phenyl)propanoate (3l')

Yield = 75%, l/b = 96:4; ^{1}H NMR (400 MHz, $CDCl_3$) δ 7.54 (d, J = 8.0 Hz, 2H), 7.32 (d, J = 7.6 Hz, 2H)，3.67 (s, 3H), 3.01 (t, J = 8.0 Hz, 2H), 2.65 (t, J = 7.6 Hz, 2H); ^{13}C NMR (101 MHz, $CDCl_3$) δ 173.0, 144.7, 128.8, 125.6 (q, J = 3.9 Hz), 122.9, 51.9, 35.3, 30.8.

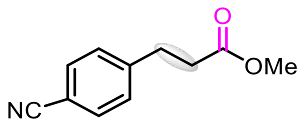

3m'

Methyl-3-(4-cyanophenyl)propanoate (3m')

Yield = 70%, l/b = 93:7; ^{1}H NMR (400 MHz, $CDCl_3$) δ 7.60-7.58 (m, 2H), 7.32 (d, J = 8.0 Hz, 2H), 3.67 (s, 3H), 3.02 (t, J = 7.6 Hz, 2H), 2.66 (t, J = 7.6 Hz, 2H); ^{13}C NMR (101 MHz, $CDCl_3$) δ 172.7, 146.2, 132.4, 129.3, 119.0, 110.3, 51.9, 34.9, 30.9.

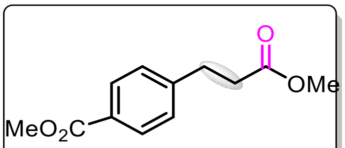

3n'

Methyl-4-(3-methoxy-3-oxopropyl)benzoate (3n')

Yield = 88%, l/b = 92:8, ^{1}H NMR (400 MHz, $CDCl_3$) δ 7.97-7.95 (m, 2H), 7.28-7.26 (m, 2H), 3.90 (s, 3H), 3.67 (s, 3H), 3.01 (t, J = 8.0 Hz, 2H), 2.65 (t, J = 7.6 Hz, 2H); ^{13}C NMR (101 MHz, $CDCl_3$) δ 173.1, 167.2, 146.0, 130.0, 128.5, 52.2, 51.8, 35.3, 31.0.

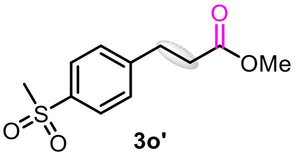

3o'

Methyl-3-(4-(methylsulfonyl)phenyl)propanoate (3o')

Yield = 76%, l/b = 96:4, ^{1}H NMR (400 MHz, $CDCl_3$) δ 7.87 (d, J = 8.4 Hz, 2H), 7.42 (d, J = 8.4 Hz, 2H), 3.68 (s, 3H), 3.07-3.03 (m, 5H), 2.68 (t, J = 7.6 Hz, 2H); ^{13}C NMR (101 MHz, $CDCl_3$) δ 172.8, 147.2, 138.6, 129.4, 127.7, 51.9, 44.6, 35.0, 30.7.

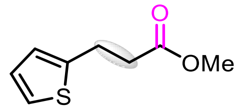

3p'

Methyl-3-(thiophen-2-yl)propanoate (3p')

Yield = 61%, l/b = 90:10, ^{1}H NMR (400 MHz, CDCl$_3$) δ 7.14-7.12 (m, 1H), 6.93-6.91 (m, 1H), 6.83-6.82 (m, 1H), 3.69 (s, 3H), 3.17 (t, *J* = 7.6 Hz, 2H), 2.70 (t, *J* = 7.6 Hz, 2H); ^{13}C NMR (101 MHz, CDCl$_3$) δ 173.0, 143.1, 127.0, 124.8, 123.7, 51.9, 36.1, 25.3.

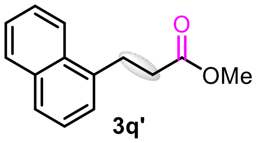

3q'

Methyl-3-(naphthalen-1-yl)propanoate (3q')

Yield = 90%, l/b = 97:3, ^{1}H NMR (400 MHz, CDCl$_3$) δ 8.02 (d, *J* = 8.4 Hz, 1H), 7.85 (d, *J* = 8.0 Hz, 1H), 7.72 (d, *J* = 8.0 Hz, 1H), 7.54-7.45 (m, 2H), 7.40-7.33 (m, 2H), 3.68 (s, 3H), 3.41 (t, *J* = 8.4 Hz, 2H), 2.76 (d, *J* = 8.4 Hz, 2H); ^{13}C NMR (101 MHz, CDCl$_3$) δ 173.6, 136.6, 134.0, 131.7, 129.0, 127.3, 126.2, 126.0, 125.7, 125.7, 123.5, 51,8, 35.1, 28.2.

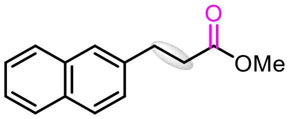

3r'

Methyl-3-(naphthalen-2-yl)propanoate (3r')

Yield = 91%, l/b = 90:10, ^{1}H NMR (400 MHz, CDCl$_3$) δ 7.80-7.75 (m, 3H), 7.63 (s, 1H), 7.46-7.39 (m, 2H), 7.33-7.31 (m, 1H), 3.66 (s, 3H), 3.11 (t, *J* = 8.0 Hz, 2H), 2.71 (t, *J* = 7.6 Hz, 2H); ^{13}C NMR (101 MHz, CDCl$_3$) δ 173.4, 138.1, 133.7, 132.3, 128.2, 127.7, 127.6, 127.1, 126.6, 126.1, 125.5, 51,8, 35.7, 31.2.

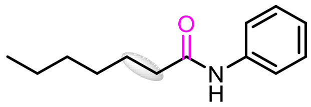

4a'

***N*-phenylheptanamide (4a')**

Yield = 77%, l/b = 88:12, ^{1}H NMR (400 MHz, $CDCl_3$) δ 7.52 (d, *J* = 8.0 Hz, 2H), 7.47 (s, 1H), 7.32-7.26 (m, 2H), 7.09 (t, *J* = 7.6 Hz, 1H), 2.34 (t, *J* = 7.6 Hz, 2H), 1.75-1.67 (m, 2H), 1.38-1.33 (m, 2H), 1.31-1.28 (m, 4H), 0.88 (t, *J* = 6.8 Hz, 3H); ^{13}C NMR (101 MHz, $CDCl_3$) δ 171.8, 138.1, 129.1, 124.3, 120.0, 37.9, 31.7, 29.1, 25.7, 22.6, 14.2.

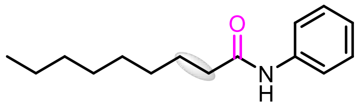

4b'

***N*-phenylnonanamide (4b')**

Yield = 80%, l/b = 86:14, ^{1}H NMR (400 MHz, $CDCl_3$) δ 7.52 (d, *J* = 8.4 Hz, 2H), 7.37 (s, 1H), 7.30 (t, *J* = 8.0 Hz, 2H), 7.09 (t, *J* = 7.6 Hz, 1H), 2.34 (t, *J* = 7.6 Hz, 2H), 1.75-1.68 (m, 2H), 1.38-1.23 (m, 10H), 0.89-0.86 (m, 3H); ^{13}C NMR (101 MHz, $CDCl_3$) δ 171.8, 138.1, 129.1, 124.3, 120.0, 37.9, 31.9, 29.5, 29.4, 29.3, 25.8, 22.8, 14.2.

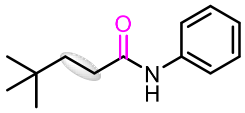

4c'

4,4-dimethyl-*N*-phenylpentanamide (4c')

Yield = 78%, l/b > 99:1, ^{1}H NMR (400 MHz, $CDCl_3$) δ 8.03 (s, 1H), 7.52 (d, *J* = 8.0 Hz, 2H), 7.26 (t, *J* = 8.0 Hz, 2H), 7.06 (t, *J* = 7.6 Hz, 1H), 2.33-2.29 (m, 2H), 1.64-1.60 (m, 2H), 0.88 (s, 9H); ^{13}C NMR (101 MHz, $CDCl_3$) δ 172.6, 138.3, 128.9, 124.2, 120.2, 39.4, 33.4, 30.2, 29.2.

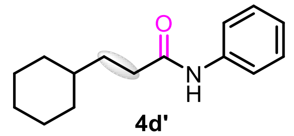

4d'

3-cyclohexyl-*N*-phenylpropanamide (4d')

Yield = 82%, l/b > 99:1, ^{1}H NMR (400 MHz, $CDCl_3$) δ 7.78 (s, 1H), 7.52 (d, *J* = 8.0 Hz, 2H), 7.28 (t, *J* = 8.0 Hz, 2H), 7.07 (t, *J* = 7.6 Hz, 1H), 2.35 (t, *J* = 8.0 Hz, 2H), 1.72-1.65 (m, 4H), 1.63-1.57 (m, 2H), 1.28-1.11 (m, 5H), 0.94-0.85 (m, 2H); ^{13}C NMR (101 MHz, $CDCl_3$) δ 172.3, 138.2, 129.0, 124.2, 120.1, 37.4, 35.3, 33.1, 26.6, 26.3.

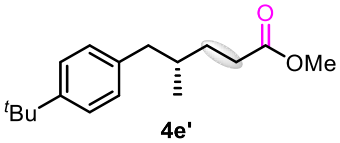

4e'

Methyl-(*S*)-5-(4-(*tert*-butyl)phenyl)-4-methylpentanoate (4e')

Yield = 91%, l/b > 99:1; ^{1}H NMR (400 MHz, $CDCl_3$) δ 7.30-7.28 (m, 2H), 7.06 (d, *J* = 8.8 Hz, 2H), 3.65 (s, 3H), 2.63-2.58 (m, 1H), 2.39-2.30 (m, 3H), 1.77-1.71 (m, 2H), 1.53-1.44 (m, 1H), 1.31 (s, 9H), 0.87 (d, *J* = 6.8 Hz, 3H); ^{13}C NMR (101 MHz, $CDCl_3$) δ 174.5, 148.6, 137.9, 128.9, 125.1, 51.6, 42.9, 34.6, 34.4, 32.1, 31.7, 31.5, 19.2.

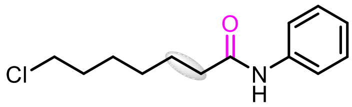

4f'

7-chloro-*N*-phenylheptanamide (4f')

Yield = 71%, l/b = 90:10, ^{1}H NMR (400 MHz, $CDCl_3$) δ 7.58 (s, 1H), 7.52 (d, *J* = 7.6 Hz, 2H), 7.30 (t, *J* = 8.0 Hz, 2H), 7.09 (t, *J* = 7.6 Hz, 1H), 3.52 (t, *J* = 6.8 Hz, 2H), 2.35 (t, *J* = 7.6 Hz, 2H), 1.80-1.71 (m, 4H), 1.50-1.43 (m, 2H), 1.41-1.35 (m, 2H); ^{13}C NMR (101 MHz, $CDCl_3$) δ 171.6, 138.1, 129.1, 124.3, 120.0, 45.1, 37.6, 32.4, 28.5, 26.6, 25.5.

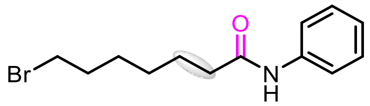

4g'

7-bromo-*N*-phenylheptanamide (4g')

Yield =65%, l/b = 89:11, ^{1}H NMR (400 MHz, $CDCl_3$) δ 7.52 (d, *J* = 8.0 Hz, 2H), 7.40 (s, 1H), 7.31 (t, *J* = 8.0 Hz, 2H), 7.10 (t, *J* = 7.6 Hz, 1H), 3.40 (t, *J* = 6.8 Hz, 2H), 2.36 (t, *J* = 7.6 Hz, 2H), 1.89-1.82 (m, 2H), 1.78-1.72 (m, 2H), 1.51-1.44 (m, 2H) , 1.43-1.36 (m, 2H); ^{13}C NMR (101 MHz, $CDCl_3$) δ 171.4, 138.0, 129.1, 124.4, 119.9, 37.6, 34.0, 32.6, 28.4, 27.9, 25.4.

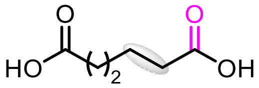

4h'

Adipic acid (4h')

Yield = 56%, l/b = 85:15, ^{1}H NMR (400 MHz, $CDCl_3$) δ 10.09 (s, 2H), 2.60-2.51 (m, 1H), 2.46 (t, *J* = 7.2 Hz, 2H), 2.06-1.97 (m, 1H), 1.87-1.79 (m, 1H), 1.22 (d, *J* = 7.2 Hz, 3H); ^{13}C NMR (101 MHz, $CDCl_3$) δ 182.7, 179.8, 38.7, 31.8, 28.2, 17.0.

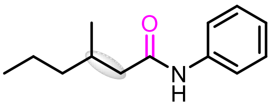

4i'

3-methyl-*N*-phenylhexanamide (4i')

Yield = 81%, l/b > 99:1; ^{1}H NMR (400 MHz, $CDCl_3$) δ 8.18 (s, 1H), 7.54 (d, *J* = 8.4 Hz, 2H), 7.26 (t, *J* = 8.0 Hz, 2H), 7.07 (t, *J* = 7.6 Hz, 1H), 2.35-2.31 (m, 1H), 2.14-2.01 (m, 2H), 1.38-1.15 (m, 4H), 0.94 (d, *J* = 6.4 Hz, 3H), 0.86 (t, *J* = 6.8 Hz, 3H); ^{13}C NMR (101 MHz, $CDCl_3$) δ 171.8, 138.2, 128.9, 124.2, 120.3, 45.4, 39.1, 30.7, 20.1, 19.6, 14.3.

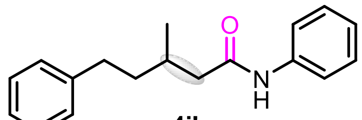

3-methyl-*N*,5-diphenylpentanamide (4j')

Yield = 80%, l/b > 99:1, ^{1}H NMR (400 MHz, $CDCl_3$) δ 7.99-7.97 (m, 1H), 7.50 (d, *J* = 8.0 Hz, 2H), 7.26-7.22 (m, 4H), 7.16-7.11 (m, 3H), 7.05 (t, *J* = 7.6 Hz, 1H), 2.67-2.60 (m, 1H), 2.57-2.49 (m, 1H), 2.38-2.31 (m, 1H), 2.15-2.05 (m, 2H), 1.71-1.62 (m, 1H), 1.52-1.43 (m, 1H), 1.00 (d, *J* = 6.4 Hz, 3H); ^{13}C NMR (101 MHz, $CDCl_3$) δ 171.4, 142.4, 138.1, 128.9, 128.4, 128.4, 125.8, 124.3, 120.2, 45.2, 38.6, 33.4, 30.7, 19.6.

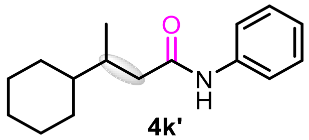

3-cyclohexyl-*N*-phenylbutanamide (4k')

Yield = 75%, l/b > 99:1; ^{1}H NMR (400 MHz, $CDCl_3$) δ 8.06 (s, 1H), 7.54 (d, *J* = 8.0 Hz, 2H), 7.26 (t, *J* = 8.0 Hz, 2H), 7.07 (t, *J* = 7.6 Hz, 1H), 2.45-2.40 (m, 1H), 2.10-2.04 (m, 1H), 2.01-1.91 (m, 1H), 1.73-1.69 (m, 2H), 1.65-1.58 (m, 3H), 1.24-1.06 (m, 4H), 1.03-0.96 (m, 2H), 0.90 (d, *J* = 6.8 Hz, 3H); ^{13}C NMR (101 MHz, $CDCl_3$) δ 172.1, 138.2, 128.9, 124.2, 120.2, 42.7, 42.6, 35.9, 30.5, 28.9, 26.8, 26.7, 26.7, 16.3.

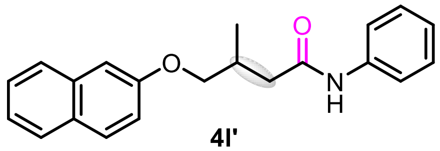

3-methyl-4-(naphthalen-2-yloxy)-*N*-phenylbutanamide (4l')

Yield = 76%, l/b > 99:1; ^{1}H NMR (400 MHz, $CDCl_3$) δ 7.77-7.68 (m, 3H), 7.47-7.41 (m, 4H), 7.35-7.31 (m, 1H), 7.30-7.25 (m, 2H), 7.15-7.12 (m, 2H), 7.08 (t, *J* = 7.6 Hz, 1H), 4.08-4.05 (m, 1H), 3.97-3.93 (m, 1H), 2.70-2.61 (m, 2H), 2.41-2.34 (m, 1H), 1.19 (d, *J* = 6.0 Hz, 3H); ^{13}C NMR (101 MHz, $CDCl_3$) δ 170.5, 156.8, 137.9, 134.6, 129.6, 129.1, 127.8, 126.9, 126.6, 124.4, 123.8, 120.0, 118.8, 106.9, 72.1, 41.9, 31.2,

17.3.

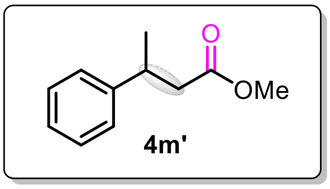

Methyl-3-phenylbutanoate (4m')

Yield = 86%, l/b = 99:1, ^{1}H NMR (400 MHz, $CDCl_3$) δ 7.32-7.28 (m, 2H), 7.25-7.17 (m, 3H), 3.62 (s, 3H), 3.32-3.23 (m, 1H), 2.66-2.52 (m, 2H), 1.30 (d, *J* = 7.2 Hz, 3H); ^{13}C NMR (101 MHz, $CDCl_3$) δ 173.0, 145.8, 128.6, 126.8, 126.5, 51.6, 42.9, 36.6, 21.9.

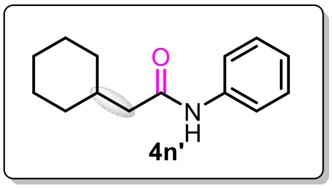

2-cyclohexyl-*N*-phenylacetamide (4n')

Yield = 75%, l/b > 99:1; ^{1}H NMR (400 MHz, $CDCl_3$) δ 7.54-7.50 (m, 3H), 7.30 (t, *J* = 8.0 Hz, 2H), 7.09 (t, *J* = 7.6 Hz, 1H), 2.21 (d, *J* = 7.6 Hz, 2H), 1.93-1.83 (m, 1H), 1.81-1.77 (m, 2H), 1.72-1.63 (m, 3H), 1.33-1.22 (m, 2H), 1.19-1.08 (m, 1H), 1.03-0.93 (m, 2H); ^{13}C NMR (101 MHz, $CDCl_3$) δ 171.2, 138.1, 129.1, 124.3, 120.0, 46.0, 35.6, 33.2, 26.3, 26.2.

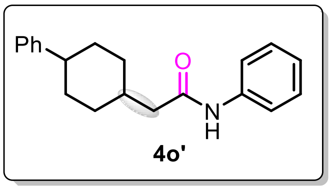

***N*-phenyl-2-(4-phenylcyclohexyl)acetamide (4o')**

Yield = 70%, l/b > 99:1; ^{1}H NMR (400 MHz, $CDCl_3$) δ 7.55 (d, *J* = 8.0 Hz, 2H), 7.40 (s, 1H), 7.34-7.25 (m, 4H), 7.20-7.16 (m, 3H), 7.11 (t, *J* = 7.6 Hz, 1H), 2.47 (t, *J* = 12.4 Hz, 1H), 2.28 (d, *J* = 7.6 Hz, 2H), 1.98-1.90 (m, 4H), 1.57-1.48 (m, 2H), 1.26-1.15 (m, 3H); ^{13}C NMR (101 MHz, $CDCl_3$) δ 170.9, 147.3, 138.0, 129.1, 128.5, 126.9, 126.1, 124.4, 120.0, 45.8, 44.2, 35.2, 34.0, 33.4.

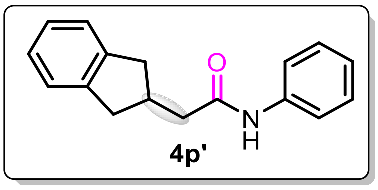

2-(2,3-dihydro-1H-inden-2-yl)-*N*-phenylacetamide (4p')

Yield = 74%, l/b > 99:1, ^{1}H NMR (400 MHz, CDCl$_3$) δ 7.53-7.50 (m, 3H), 7.29 (t, *J* = 8.4 Hz, 2H), 7.19-7.16 (m, 2H), 7.15-7.12 (m, 2H), 7.09 (t, *J* = 7.6 Hz, 1H), 3.17-3.12 (m, 2H), 3.02-2.95 (m, 1H), 2.68-2.63 (m, 2H), 2.47 (d, *J* = 7.6 Hz, 2H); ^{13}C NMR (101 MHz, CDCl$_3$) δ 170.8, 142.7, 138.0, 129.1, 126.4, 124.7, 124.4, 120.1, 43.3, 39.0, 36.6.

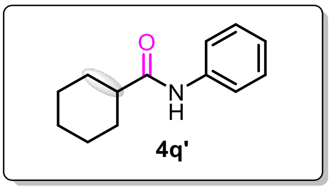

***N*-phenylcyclohexanecarboxamide (4q')**

Yield = 74%, ^{1}H NMR (400 MHz, CDCl$_3$) δ 7.86 (s, 1H), 7.55 (d, *J* = 8.0 Hz, 2H), 7.27 (t, *J* = 8.0 Hz, 2H), 7.06 (t, *J* = 7.6 Hz, 1H), 2.28-2.21 (m, 1H), 1.93-1.89 (m, 2H), 1.81-1.76 (m, 2H), 1.68-1.64 (m, 1H), 1.58-1.48 (m, 2H), 1.30-1.18 (m, 3H); ^{13}C NMR (101 MHz, CDCl$_3$) δ 175.0, 138.3, 128.9, 124.1, 120.0, 46.4, 29.7, 25.7, 25.7.

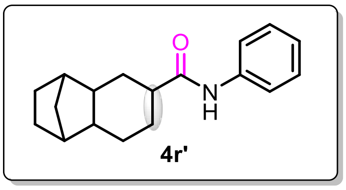

***N*-phenyldecahydro-1,4-methanonaphthalene-6-carboxamide (4r')**

Yield = 70%, ^{1}H NMR (400 MHz, CDCl$_3$) δ 7.70-7.59 (m, 1H), 7.53 (d, *J* = 8.0 Hz, 2H), 7.29-7.25 (m, 2H), 7.06 (t, *J* = 7.2 Hz, 1H), 2.70-2.35 (m, 1H), 1.96-1.89 (m, 1H), 1.86 (s, 2H), 1.81-1.75 (m, 1H), 1.73-1.68 (m, 1H), 1.60-1.57 (m, 1H), 1.55-1.49 (m, 3H), 1.47-1.37 (m, 4H), 1.20-1.15 (m, 2H), 1.00 (d, *J* = 10.4 Hz, 1H); ^{13}C NMR (101 MHz, CDCl$_3$) δ 176.2, 175.9, 138.5, 138.4, 129.0, 124.1, 124.0, 120.0, 119.9, 43.2, 42.5, 42.3, 42.2, 40.4, 39.1, 38.6, 33.0, 32.9, 29.8, 29.7, 29.4, 27.7, 27.1, 23.8, 23.4,

23.0, 22.9.

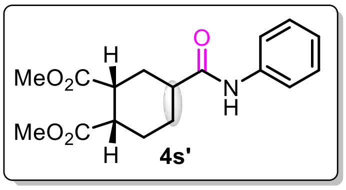

Dimethyl-(1*S*,2*R*)-4-(phenylcarbamoyl)cyclohexane-1,2-dicarboxylate (4s')

Yield = 73%, ^{1}H NMR (400 MHz, $CDCl_3$) δ 7.51 (d, *J* = 8.0 Hz, 2H), 7.34-7.30 (m, 3H), 7.10 (t, *J* = 7.6 Hz, 1H), 3.70 (s, 6H), 3.38-3.35 (m, 1H), 2.62-2.57 (m, 1H), 2.47-2.40 (m, 1H), 2.38-2.32 (m, 1H), 2.16-2.11 (m, 1H), 2.05-2.00 (m, 1H), 1.95-1.91 (m, 1H), 1.71-1.63 (m, 2H); ^{13}C NMR (101 MHz, $CDCl_3$) δ 174.0, 173.9, 173.3, 138.0, 129.2, 124.5, 119.9, 52.1, 52.0, 43.1, 41.6, 40.8, 30.3, 28.5, 23.5.

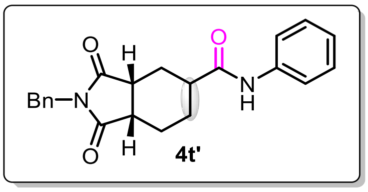

(3a*R*,7a*S*)-2-benzyl-1,3-dioxo-*N*-phenyloctahydro-1H-isoindole-5-carboxamide (4t')

Yield = 65%, ^{1}H NMR (400 MHz, $CDCl_3$) δ 7.58 (s, 1H), 7.47 (d, *J* = 8.0 Hz, 2H), 7.37-7.34 (m, 2H), 7.33-7.26 (m, 5H), 7.08 (t, *J* = 7.6 Hz, 1H), 4.68-4.58 (m, 2H), 3.09-3.05 (m, 1H), 2.94-2.88 (m, 1H), 2.36-2.31 (m, 1H), 2.19-2.13 (m, 1H), 2.11-2.04 (m, 1H), 1.99-1.91 (m, 1H), 1.74-1.62 (m, 2H), 1.41-1.31 (m, 1H); ^{13}C NMR (101 MHz, $CDCl_3$) δ 179.3, 179.0, 173.0, 137.9, 136.0, 129.1, 128.8, 128.7, 128.1, 124.5, 119.9, 42.4, 40.7, 39.2, 24.5, 24.4.

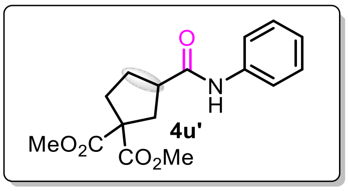

Dimethyl-3-(phenylcarbamoyl)cyclopentane-1,1-dicarboxylate (4u')

Yield = 83%, ^{1}H NMR (400 MHz, $CDCl_3$) δ 8.00 (s, 1H), 7.54 (d, *J* = 8.0 Hz, 2H), 7.30 (t, *J* = 8.0 Hz, 2H), 7.09 (t, *J* = 7.6 Hz, 1H), 3.75 (d, *J* = 5.6 Hz, 6H), 2.96-2.88 (m,

1H), 2.61-2.51 (m, 2H), 2.49-2.43 (m, 1H), 2.24-2.17 (m, 1H), 2.13-2.06 (m, 1H), 2.04-1.98 (m, 1H); ^{13}C NMR (101 MHz, CDCl$_3$) δ 172.7, 172.6, 138.2, 129.0, 124.3, 119.8, 60.4, 53.1, 53.0, 46.5, 37.7, 34.2, 30.2.

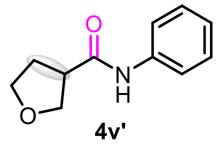

4v'

***N*-phenyltetrahydrofuran-3-carboxamide (4v')**

Yield = 91%, ^{1}H NMR (400 MHz, CDCl$_3$) δ 7.75 (s, 1H), 7.51 (d, *J* = 8.0 Hz, 2H), 7.31 (t, *J* =8.0 Hz, 2H), 7.11 (t, *J* = 7.6 Hz, 1H), 4.06-4.00 (m, 2H), 3.98-3.93 (m, 1H), 3.87-3.81 (m, 1H), 3.08-3.02 (m, 1H), 2.32-2.18 (m, 2H); ^{13}C NMR (101 MHz, CDCl$_3$) δ 172.2, 137.9, 129.1, 124.5, 120.1, 71.1, 68.3, 46.6, 30.7.

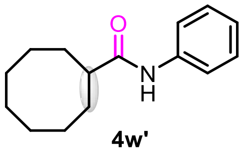

4w'

***N*-phenylcyclooctanecarboxamide (4w')**

Yield = 86%, ^{1}H NMR (400 MHz, CDCl$_3$) δ 7.53 (d, *J* = 8.0 Hz, 3H), 7.28 (t, *J* = 8.0 Hz, 2H), 7.07 (t, *J* = 7.6 Hz, 1H), 2.47-2.40 (m, 1H), 1.95-1.88 (m, 2H), 1.84-1.73 (m, 4H), 1.64-1.45 (m, 8H); ^{13}C NMR (101 MHz, CDCl$_3$) δ 176.3, 138.4, 129.0, 124.1, 120.0, 46.8, 29.9, 26.7, 26.3, 25.5.

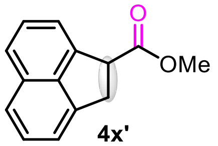

4x'

Methyl-1,2-dihydroacenaphthylene-1-carboxylate (4x')

Yield = 65%, ^{1}H NMR (400 MHz, CDCl$_3$) δ 7.69-7.65 (m, 1H), 7.62 (d, *J* = 8.0 Hz, 1H), 7.51-7.44 (m, 3H), 7.32 (d, *J* = 7.2 Hz, 1H), 4.60-4.57 (m, 1H), 3.90-3.84 (m, 1H), 3.79 (s, 3H), 3.65-3.59 (m, 1H); ^{13}C NMR (101 MHz, CDCl$_3$) δ 173.5, 143.4, 142.2, 138.3, 131.7, 128.2, 127.9, 124.1, 122.8, 120.5, 119.7, 52.5, 48.5, 34.3.

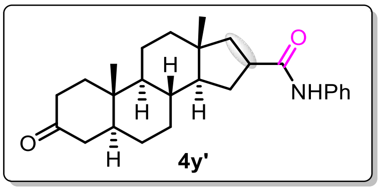

(5*S*,8*S*,9*S*,10*S*,13*R*,14*S*)-10,13-dimethyl-3-oxo-*N*-phenylhexadecahydro-1H-cyclopenta[a]phenanthrene-16-carboxamide (4y')

Yield = 71%, ^{1}H NMR (400 MHz, $CDCl_3$) δ 7.72-7.70 (m, 1H), 7.54 (d, *J* = 8.0 Hz, 2H), 7.31-7.27 (m, 2H), 7.07 (t, *J* = 7.6 Hz, 1H), 2.94- 2.86 (m, 1H), 2.41-2.22 (m, 3H), 2.11-1.98 (m, 3H), 1.83-1.78 (m, 1H), 1.76-1.65 (m, 2H), 1.60-1.47 (m, 4H), 1.39-1.29 (m, 5H), 1.27-1.23 (m, 3H), 1.02-0.96 (m, 4H), 0.76 (s, 3H); ^{13}C NMR (101 MHz, $CDCl_3$) δ 212.4, 175.7, 138.4, 129.0, 124.0, 119.8, 53.7, 53.6, 46.6, 45.6, 44.7, 42.8, 42.1, 38.6, 38.2, 38.1, 35.7, 35.6, 31.7, 30.9, 28.9, 21.3, 18.0, 11.5.

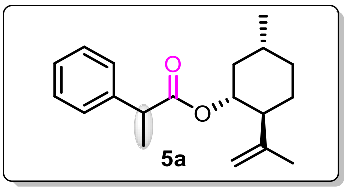

(*1R*,*2S*,*5R*)-5-methyl-2-(prop-1-en-2-yl)cyclohexyl-2-phenylpropanoate (5a)

Yield = 73%, ^{1}H NMR (400 MHz, $CDCl_3$) δ 7.30-7.20 (m, 5H), 4.80-4.74 (m, 1H), 4.72-4.71 (m, 1H), 4.52-4.45 (m, 1H), 3.66-3.60 (m, 1H), 2.13-1.87 (m, 2H), 1.68-1.65 (m, 1H), 1.64-1.62 (m, 2H), 1.55-1.49 (m, 1H), 1.46-1.43 (m, 5H), 1.38-1.31 (m, 1H), 1.04-1.00 (m, 1H), 0.93-0.87 (m, 4H); ^{13}C NMR (101 MHz, $CDCl_3$) δ 174.1, 174.0, 146.3, 145.8, 140.9, 140.8, 128.5, 128.4, 127.7, 127.6, 127.0, 127.0, 111.9, 111.8, 74.0, 73.8, 50.8, 50.7, 45.9, 45.8, 40.5, 40.1, 34.2, 34.2, 31.5, 31.4, 30.6, 30.5, 22.1, 22.1, 19.6, 19.4, 18.8, 18.4. HRMS calcd. (ESI) m/z for $C_{19}H_{26}O_2Na$ $[M+Na]^+$: 309.1825, found: 309.1823.

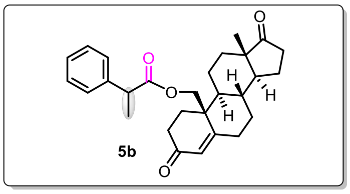

((8*R*,9*S*,10*S*,13*S*,14*S*)-13-methyl-3,17-dioxo-1,2,3,6,7,8,9,11,12,13,14,15,16,17-tetradecahydro-10H-cyclopenta[a]phenanthren-10-yl)methyl-2-phenylpropanoate (5b)

Yield = 89%, ^{1}H NMR (400 MHz, $CDCl_3$) δ 7.23-7.17 (m, 3H), 7.14-7.09 (m, 2H), 5.77-5.56 (m, 1H), 4.60-4.41 (m, 1H), 4.14-3.99 (m, 1H), 3.58-3.57 (m, 1H), 2.36-2.17 (m, 1H), 2.16-2.01 (m, 4H), 2.08-1.99 (m, 2H), 1.88-1.81 (m, 2H), 1.70-1.59 (m, 4H), 1.50-1.43 (m, 1H), 1.40-1.38 (m, 3H), 1.33-1.21 (m, 1H), 1.15-1.09 (m, 2H), 1.00-0.95 (m, 2H), 0.75 (s, 3H); ^{13}C NMR (101 MHz, $CDCl_3$) δ 219.8, 198.9, 198.8, 174.1, 174.0, 164.9, 164.3, 139.8, 139.7, 128.7, 128.7, 127.3, 127.2, 126.6, 126.5, 66.6, 66.4, 53.9, 53.8, 50.9, 47.3, 45.5, 45.5, 41.8, 41.6, 35.5, 35.4, 35.4, 34.2, 34.1, 33.3, 33.1, 32.8, 32.7, 31.4, 30.6, 21.5, 20.6, 17.8, 17.7, 13.7. HRMS calcd. (ESI) m/z for $C_{28}H_{34}O_4Na$ [M+Na]+: 457.2349, found: 457.2351.

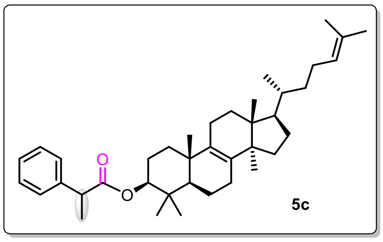

(3*S*,5*R*,10*S*,13*R*,14*R*,17*R*)-4,4,10,13,14-pentamethyl-17-((*R*)-6-methylhept-5-en-2-yl)-2,3,4,5,6,7,10,11,12,13,14,15,16,17-tetradecahydro-1H-cyclopenta[a]phenanthren-3-yl-2-phenylpropanoate (5c)

Yield = 83%, ^{1}H NMR (400 MHz, $CDCl_3$) δ 7.32-7.30 (m, 4H), 7.25-7.22 (m, 1H), 4.50-4.46 (m, 1H), 3.73-3.70 (m, 1H), 2.05-1.94 (m, 5H), 1.92-1.82 (m, 1H), 1.73-1.64 (m, 6H), 1.61-1.60 (m, 3H), 1.57-1.48 (m, 6H), 1.46-1.43 (m, 2H), 1.38-1.26 (m, 5H), 1.16-1.10 (m, 3H), 0.98-0.96 (m, 3H), 0.93-0.86 (m, 8H), 0.83-0.82 (m, 4H), 0.74-0.73 (m, 1H), 0.69-0.68 (m, 3H), 0.60-0.59 (m, 1H); ^{13}C NMR (101 MHz, $CDCl_3$) δ 174.3, 174.2, 141.0, 140.8, 134.5, 134.3, 130.9, 128.5, 127.7, 127.6, 127.1, 127.0, 125.4, 81.1, 81.0, 50.6, 50.5, 50.5, 49.9, 46.2, 46.0, 44.5, 39.6, 38.1, 37.9, 37.0, 36.6, 36.4, 36.4, 35.3, 35.2, 31.0, 30.9, 29.8, 28.3, 28.1, 28.0, 27.6, 26.4, 25.9, 25.0, 24.3, 24.3, 24.2, 23.9, 23.0, 22.7, 21.1, 19.2, 18.8, 18.7, 18.4, 18.2, 18.1, 17.7, 16.6, 16.5, 15.8. HRMS calcd. (ESI) m/z for $C_{39}H_{58}O_2Na$ $[M+Na]^+$: 581.4329, found: 581.4339.

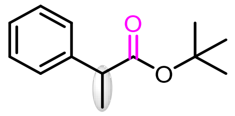

5d

***tert*-butyl-2-phenylpropanoate (5d)**

Yield = 88%, ^{1}H NMR (400 MHz, $CDCl_3$) δ 7.33-7.27 (m, 4H), 7.25-7.21 (m, 1H), 3.63-3.58 (m, 1H), 1.45 (d, J = 7.2 Hz, 3H), 1.39 (s, 9H); ^{13}C NMR (101 MHz, $CDCl_3$) δ 174.0, 141.3, 128.6, 127.5, 126.9, 80.6, 46.6, 28.1, 18.6. HRMS calcd. (ESI) m/z for $C_{13}H_{18}O_2Na$ [M+Na]+: 229.1199, found: 229.1194.

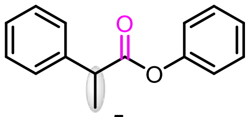

5e

Phenyl-2-phenylpropanoate (5e)

Yield = 72%, ^{1}H NMR (400 MHz, $CDCl_3$) δ 7.42-7.24 (m, 7H), 7.20-7.16 (m, 1H), 7.00-6.97 (m, 2H), 3.99-3.94 (m, 1H), 1.61 (d, J = 7.2 Hz, 3H); ^{13}C NMR (101 MHz, $CDCl_3$) δ 173.2, 150.9, 140.2, 129.5, 128.9, 127.7, 127.5, 125.9, 121.5, 45.8, 18.7. HRMS calcd. (ESI) m/z for $C_{15}H_{14}O_2Na$ [M+Na]+: 249.0886, found: 249.0889.

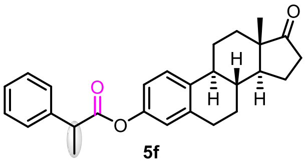

5f

(8*R*,9*S*,13*S*,14*S*)-13-methyl-17-oxo-7,8,9,11,12,13,14,15,16,17-decahydro-6H-cyclopenta[a]phenanthren-3-yl-2-phenylpropanoate (5f)

Yield = 74%, ^{1}H NMR (400 MHz, $CDCl_3$) δ 7.41-7.34 (m, 4H), 7.31-7.27 (m, 1H), 7.23 (d, J = 8.8 Hz, 1H), 6.78-6.75 (m, 1H), 6.72 (s, 1H), 3.97-3.92 (m, 1H), 2.87-2.84 (m, 2H), 2.52-2.46 (m, 1H), 2.40-2.35 (m, 1H), 2.28-2.22 (m, 1H), 2.17-2.08 (m, 1H), 2.06-1.92 (m, 3H), 1.61-1.57 (m, 4H), 1.56-1.38 (m, 5H), 0.89 (s, 3H); ^{13}C NMR (101 MHz, $CDCl_3$) δ 220.9, 173.3, 148.7, 140.2, 138.0, 137.4, 128.9, 127.6, 127.4, 126.4,

121.4, 118.6, 50.4, 48.0, 45.7, 44.2, 38.0, 35.9, 31.6, 29.4, 26.4, 25.8, 21.6, 18.7, 13.9. HRMS calcd. (ESI) m/z for $C_{27}H_{30}O_3Na$ [M+Na]+: 425.2087, found: 425.2091.

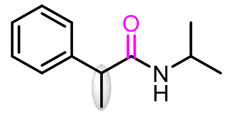

5g

N-isopropyl-2-phenylpropanamide (5g)

Yield = 92%, ^{1}H NMR (400 MHz, $CDCl_3$) δ 7.36-7.31 (m, 2H), 7.30-7.25 (m, 3H), 5.19 (s, 1H), 4.10-3.98 (m, 1H), 3.53-3.48 (m, 1H), 1.50 (d, *J* = 7.2 Hz, 3H), 1.07 (d, *J* = 6.4 Hz, 3H), 1.02 (d, *J* = 6.8 Hz, 3H); ^{13}C NMR (101 MHz, $CDCl_3$) δ 173.3, 141.7, 129.0, 127.7, 127.2, 47.3, 41.5, 22.7, 22.6, 18.8. HRMS calcd. (ESI) m/z for $C_{12}H_{17}NONa$ [M+Na]+: 214.1202, found: 214.1217.

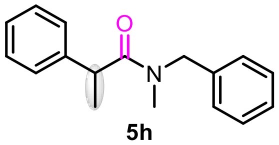

5h

***N*-benzyl-*N*-methyl-2-phenylpropanamide (5h)**

Yield = 91%, ^{1}H NMR (400 MHz, $CDCl_3$) δ 7.33-7.22 (m, 8H), 7.17-7.00 (m, 2H), 4.68-4.63 (m, 1H), 4.56-4.22 (m, 1H), 3.95-3.84 (m, 1H), 2.93-2.79 (m, 3H), 1.49-1.45 (m, 3H); ^{13}C NMR (101 MHz, $CDCl_3$) δ 174.3, 173.9, 142.1, 141.8, 137.6, 136.8, 129.0, 128.9, 128.6, 128.0, 127.6, 127.5, 127.4, 127.3, 127.0, 126.9, 126.4, 53.0, 51.3, 43.6, 43.2, 34.8, 34.3, 21.1, 20.9. HRMS calcd. (ESI) m/z for $C_{17}H_{19}NONa$ [M+Na]+: 276.1359, found: 276.1370.

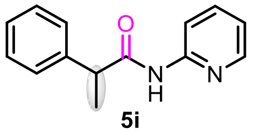

5i

2-phenyl-*N*-(pyridin-2-yl)propanamide (5i)

Yield = 78%, ^{1}H NMR (400 MHz, $CDCl_3$) δ 8.23 (d, *J* = 8.4 Hz, 1H), 8.19-8.17 (m, 1H), 7.95 (s, 1H), 7.70-7.66 (m, 1H), 7.39-7.35 (m, 4H), 7.33-7.28 (m, 1H), 7.01-

6.98 (m, 1H), 3.77-3.71 (m, 1H), 1.60 (d, J = 6.8 Hz, 3H); ^{13}C NMR (101 MHz, $CDCl_3$) δ 172.9, 151.5, 147.7, 140.6, 138.6, 129.3, 127.8, 119.9, 114.0, 48.4, 18.6. HRMS calcd. (ESI) m/z for $C_{14}H_{14}N_2ONa$ [M+Na]+: 249.0998, found: 249.1011.

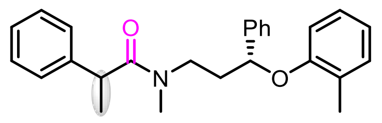

5j

N-methyl-2-phenyl-N-((R)-3-phenyl-3-(o-tolyloxy)propyl)propanamide (5j)

Yield = 75%, ^{1}H NMR (400 MHz, $CDCl_3$) δ 7.34-7.18 (m, 9H), 7.15-7.06 (m, 2H), 6.97-6.84 (m, 1H), 6.80-6.71 (m, 1H), 6.57-6.34 (m, 1H), 5.15-4.95 (m, 1H), 3.84-3.18 (m, 3H), 2.93-2.80 (m, 3H), 2.39-2.30 (m, 3H), 2.16-1.76 (m, 2H), 1.43-1.39 (m, 2H), 1.26-1.21 (m, 1H); ^{13}C NMR (101 MHz, $CDCl_3$) δ 173.9, 173.6, 155.9, 155.7, 155.5, 142.3, 142.2, 142.0, 141.9, 141.7, 141.0, 130.9, 130.7, 130.6, 129.0, 128.9, 128.9, 128.8, 128.7, 128.7, 127.9, 127.8, 127.6, 127.5, 127.4, 127.3, 127.0, 126.9, 126.8, 126.7, 126.7, 125.7, 125.6, 120.7, 120.6, 120.4, 120.3, 112.8, 112.5, 112.5, 77.6, 77.04, 76.11, 46.5, 46.2, 46.1, 45.9, 43.7, 43.2, 42.7, 37.5, 37.0, 36.4, 35.7, 34.0, 33.7, 21.1, 20.8, 20.8, 20.7, 16.8, 16.6. HRMS calcd. (ESI) m/z for $C_{26}H_{29}NO_2Na$ [M+Na]+: 410.2091, found: 410.2094.

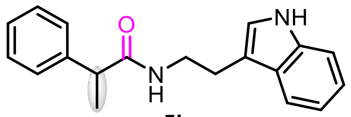

5k

N-(2-(1H-indol-3-yl)ethyl)-2-phenylpropanamide (5k)

Yield = 71%, ^{1}H NMR (400 MHz, $CDCl_3$) δ 8.50 (s, 1H), 7.49 (d, J = 7.6 Hz, 1H), 7.31-7.14 (m, 7H), 7.06 (t, J = 7.6 Hz, 1H), 6.64 (d, J = 2.4 Hz, 1H), 5.60-5.57 (m, 1H), 3.56-3.40 (m, 3H), 2.89-2.77 (m, 2H), 1.47 (d, J = 6.8 Hz, 3H); ^{13}C NMR (101 MHz, $CDCl_3$) δ 174.4, 141.4, 136.4, 128.9, 127.7, 127.2, 127.2, 122.3, 122.0, 119.3, 118.6, 112.4, 111.4, 47.1, 39.9, 25.1, 18.5. HRMS calcd. (ESI) m/z for $C_{19}H_{20}N_2ONa$ [M+Na]+: 315.1468, found: 315.1483.

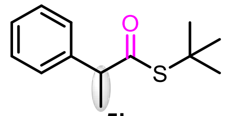

5l

***S*-(*tert*-butyl)-2-phenylpropanethioate (5l)**

Yield = 93%, ^{1}H NMR (400 MHz, $CDCl_3$) δ 7.35-7.23 (m, 5H), 3.83-3.77 (m, 1H), 1.48 (d, *J* = 7.2 Hz, 3H), 1.42 (s, 9H); ^{13}C NMR (101 MHz, $CDCl_3$) δ 201.8, 140.4, 128.7, 127.9, 127.3, 54.7, 48.1, 29.9, 18.7. HRMS calcd. (ESI) m/z for $C_{13}H_{18}OSNa$ [M+Na]+: 245.0971, found: 245.0976.

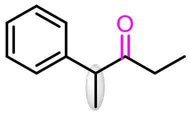

5m

2-phenylpentan-3-one (5m)

Yield = 92%, ^{1}H NMR (400 MHz, $CDCl_3$) δ 7.35-7.31 (m, 2H), 7.27-7.25 (m, 1H), 7.24-7.20 (m, 2H), 3.79-3.74 (m, 1H), 2.46-2.30 (m, 2H), 1.39 (d, *J* = 7.2 Hz, 3H), 0.96 (t, *J* = 7.2 Hz, 3H); ^{13}C NMR (101 MHz, $CDCl_3$) δ 211.8, 141.0, 129.0, 128.0, 127.2, 52.8, 34.4, 17.7, 8.1. HRMS calcd. (ESI) m/z for $C_{11}H_{14}ONa$ [M+Na]+: 185.0937, found: 185.0948.

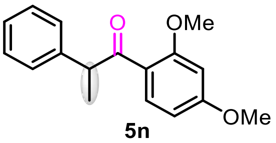

5n

1-(2,4-dimethoxyphenyl)-2-phenylpropan-1-one (5n)

Yield = 83%, ^{1}H NMR (400 MHz, $CDCl_3$) δ 7.66 (d, *J* = 9.2 Hz, 1H), 7.26-7.21 (m, 4H), 7.19-7.13 (m, 1H), 6.46-6.43 (m, 1H), 6.36 (d, *J* = 2.4 Hz, 1H), 4.79-4.74 (m, 1H), 3.80 (d, *J* = 6.4 Hz, 6H), 1.47 (d, *J* = 6.4 Hz, 3H); ^{13}C NMR (101 MHz, $CDCl_3$) δ 201.7, 164.1, 160.1, 142.3, 133.1, 128.5, 128.2, 126.5, 121.4, 105.1, 98.5, 55.6, 55.4, 51.3, 19.4. HRMS calcd. (ESI) m/z for $C_{17}H_{18}O_3Na$ [M+Na]+: 293.1148, found: 293.1163.

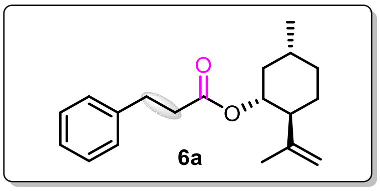

(1*R*,2*S*,5*R*)-5-methyl-2-(prop-1-en-2-yl)cyclohexyl-3-phenylpropanoate (6a)

Yield = 70%, ^{1}H NMR (400 MHz, $CDCl_3$) δ 7.29-7.25 (m, 2H), 7.20-7.17 (m, 3H), 4.84-4.77 (m, 1H), 4.70-4.69 (m, 2H), 2.93-2.88 (m, 2H), 2.57-2.53 (m, 2H), 2.13-2.06 (m, 1H), 1.98-1.93 (m, 1H), 1.72-1.67 (m, 2H), 1.62 (s, 3H), 1.58-1.49 (m, 1H), 1.42-1.32 (m, 1H), 1.03-0.91 (m, 5H); ^{13}C NMR (101 MHz, $CDCl_3$) δ 172.4, 146.2, 140.8, 128.5, 128.4, 126.2, 111.9, 73.6, 50.8, 40.5, 36.1, 34.2, 31.5, 31.1, 30.5, 22.1, 19.5. HRMS calcd. (ESI) m/z for $C_{19}H_{26}O_2Na$ $[M+Na]^+$: 309.1825, found: 309.1827.

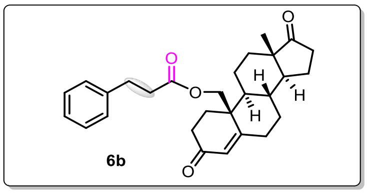

((8*R*,9*S*,10*S*,13*S*,14*S*)-13-methyl-3,17-dioxo-1,2,3,6,7,8,9,11,12,13,14,15,16,17-tetradecahydro-10H-cyclopenta[a]phenanthren-10-yl)methyl-3-phenylpropanoate (6b)

Yield = 82%, ^{1}H NMR (400 MHz, $CDCl_3$) δ 7.23-7.21 (m, 2H), 7.17-7.15 (m, 1H), 7.12-7.09 (m, 2H), 5.81 (s, 1H), 4.61 (d, *J* = 11.6 Hz, 1H), 4.09 (d, *J* = 10.8 Hz, 1H), 2.87-2.83 (m, 2H), 2.58-2.53 (m, 2H), 2.50-2.39 (m, 2H), 2.30-2.21 (m, 4H), 2.09-1.99 (m, 1H), 1.97-1.88 (m, 2H), 1.82-1.71 (m, 4H), 1.55-1.47 (m, 1H), 1.44-1.34 (m, 1H), 1.23-1.17 (m, 2H), 1.10-1.03 (m, 2H), 0.85-0.84 (m, 3H); ^{13}C NMR (101 MHz, $CDCl_3$) δ 219.9, 199.1, 172.5, 164.9, 139.9, 128.5, 128.1, 126.9, 126.3, 66.5, 53.9, 51.0, 47.4, 41.8, 35.7, 35.6, 34.5, 33.4, 32.8, 31.5, 30.8, 30.7, 21.6, 20.7, 13.7. HRMS calcd. (ESI) m/z for $C_{28}H_{34}O_4Na$ [M+Na]+: 457.2349, found: 457.2357.

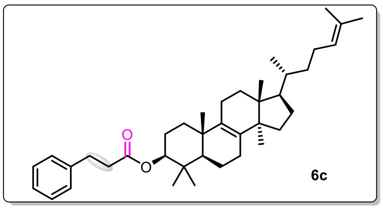

(3*S*,5*R*,10*S*,13*R*,14*R*,17*R*)-4,4,10,13,14-pentamethyl-17-((*R*)-6-methylhept-5-en-2-yl)-2,3,4,5,6,7,10,11,12,13,14,15,16,17-tetradecahydro-1H-cyclopenta[a]phenanthren-3-yl-3-phenylpropanoate (6c)

Yield = 79%, ^{1}H NMR (400 MHz, $CDCl_3$) δ 7.30-7.26 (m, 2H), 7.21-7.17 (m, 3H), 4.52-4.48 (m, 1H), 2.96 (t, *J* = 8.0 Hz, 2H), 2.66-2.62 (m, 2H), 2.05-1.97 (m, 4H), 1.95-1.86 (m, 2H), 1.74-1.57 (m, 10H), 1.56-1.52 (m, 2H), 1.50-1.45 (m, 2H), 1.38-1.25 (m, 5H), 1.16-1.12 (m, 3H), 0.99 (s, 3H), 0.92-0.90 (m, 2H), 0.86 (d, *J* = 7.6 Hz, 9H), 0.81 (s, 3H), 0.68 (s, 3H); ^{13}C NMR (101 MHz, $CDCl_3$) δ 172.9, 140.7, 134.6, 134.3, 131.1, 128.6, 128.4, 126.3, 125.4, 81.1, 50.6, 49.9, 44.6, 37.9, 37.0, 36.4, 35.4, 31.2, 31.1, 30.9, 29.8, 28.3, 28.0, 26.5, 25.9, 25.0, 24.4, 24.3, 21.1, 19.3, 18.8, 18.2, 16.7, 15.9. HRMS calcd. (ESI) m/z for $C_{39}H_{58}O_2Na$ $[M+Na]^+$: 581.4329, found: 581.4366.

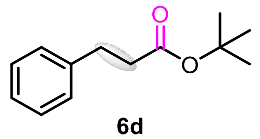

6d

***tert*-butyl-3-phenylpropanoate (6d)**

Yield = 80%, ^{1}H NMR (400 MHz, $CDCl_3$) δ 7.30-7.25 (m, 2H), 7.21-7.17 (m, 3H), 2.93-3.89 (m, 2H), 2.56-2.52 (m, 2H), 1.42 (s, 9H); ^{13}C NMR (101 MHz, $CDCl_3$) δ 172.4, 140.9, 128.5, 128.5, 126.2, 80.5, 37.2, 31.3, 28.2. HRMS calcd. (ESI) m/z for $C_{13}H_{18}O_2Na$ [M+Na]+: 229.1199, found: 229.1217.

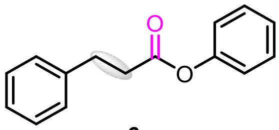

6e

Phenyl-3-phenylpropanoate (6e)

Yield = 64%, ^{1}H NMR (400 MHz, $CDCl_3$) δ 7.37-7.30 (m, 4H), 7.27-7.19 (m, 4H), 7.00 (d, *J* = 8.4 Hz, 2H), 3.07 (t, *J* = 7.6 Hz, 2H), 2.88 (t, *J* = 8.0 Hz, 2H); ^{13}C NMR (101 MHz, $CDCl_3$) δ 171.6, 150.7, 140.2, 129.5, 128.7, 128.5, 126.6, 125.9, 121.6, 36.1, 31.1. HRMS calcd. (ESI) m/z for $C_{15}H_{14}O_2Na$ [M+Na]+: 249.0886, found: 249.0889.

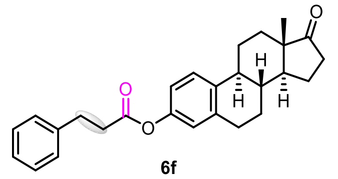

6f

(8*R*,9*S*,13*S*,14*S*)-13-methyl-17-oxo-7,8,9,11,12,13,14,15,16,17-decahydro-6H-cyclopenta[a]phenanthren-3-yl-3-phenylpropanoate (6f)

Yield = 66%, ^{1}H NMR (400 MHz, $CDCl_3$) δ 7.34-7.30 (m, 2H), 7.28-7.22 (m, 4H), 6.79-6.77 (m, 1H), 6.74-6.73 (m, 1H), 3.09-3.05 (m, 2H), 2.91-2.85 (m, 4H), 2.54-2.47 (m, 1H), 2.41-2.37 (m, 1H), 2.31-2.24 (m, 1H), 2.19-2.09 (m, 1H), 2.08-1.94 (m, 3H), 1.67-1.58 (m, 2H), 1.57-1.43 (m, 4H), 0.90 (s, 3H); ^{13}C NMR (101 MHz, $CDCl_3$) δ 221.0, 171.8, 148.6, 140.3, 138.1, 137.5, 128.7, 128.5, 126.5, 126.5, 121.7, 118.8, 50.5, 48.0, 44.2, 38.1, 36.1, 36.0, 31.6, 31.1, 29.5, 26.4, 25.8, 21.7, 13.9. HRMS calcd. (ESI) m/z for $C_{27}H_{30}O_3Na$ [M+Na]+: 425.2087, found: 425.2082.

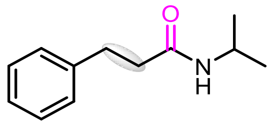

6g

***N*-isopropyl-3-phenylpropanamide (6g)**

Yield = 85%, ^{1}H NMR (400 MHz, $CDCl_3$) δ 7.30-7.26 (m, 2H), 7.21-7.19 (m, 3H), 5.13 (s, 1H), 4.07-4.02 (m, 1H), 2.96 (t, *J* = 7.6 Hz, 2H), 2.42 (t, *J* = 8.0 Hz, 2H), 1.07 (d, *J* = 6.8 Hz, 6H); ^{13}C NMR (101 MHz, $CDCl_3$) δ 171.3, 141.1, 128.6, 128.5, 126.3, 41.4, 38.9, 32.0, 22.9. HRMS calcd. (ESI) m/z for $C_{12}H_{17}NONa$ [M+Na]+: 214.1202, found: 214.1207.

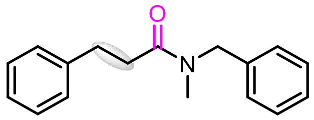

6h

***N*-benzyl-*N*-methyl-3-phenylpropanamide-*N*-phenylnonanamide (6h)**

Yield = 77%, ^{1}H NMR (400 MHz, $CDCl_3$) δ 7.35-7.07 (m, 10H), 4.59-4.45 (m, 2H), 3.05-2.97 (m, 2H), 2.95- 2.84 (m, 3H), 2.70-2.64 (m, 2H); ^{13}C NMR (101 MHz,

CDCl$_3$) δ 172.8, 172.5, 141.4, 141.3, 137.4, 136.6, 129.0, 128.7, 128.6, 128.4, 128.1, 127.7, 127.4, 126.3, 126.2, 53.4, 51.0, 35.5, 35.1, 34.9, 34.1, 31.7, 31.5, 30.9. HRMS calcd. (ESI) m/z for $C_{17}H_{19}NONa$ [M+Na]+: 276.1359, found: 276.1353.

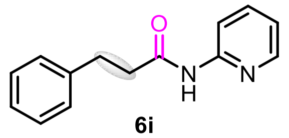

6i

3-phenyl-*N*-(pyridin-2-yl)propanamide (6i)

Yield = 69%, ^{1}H NMR (400 MHz, CDCl$_3$) δ 9.20 (s, 1H), 8.26 (d, *J* = 8.0 Hz, 1H), 8.19-8.17 (m, 1H), 7.72-7.68 (m, 1H), 7.29-7.19 (m, 5H), 7.04-6.99 (m, 1H), 3.06-3.02 (m, 2H), 2.71-2.67 (m, 2H); ^{13}C NMR (101 MHz, CDCl$_3$) δ 171.2, 151.8, 147.5, 140.6, 138.7, 128.6, 128.5, 128.4, 126.4, 119.8, 114.6, 39.2, 31.3. HRMS calcd. (ESI) m/z for $C_{14}H_{14}N_2ONa$ [M+Na]+: 249.0998, found: 249.0995.

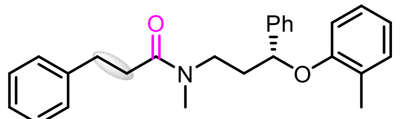

6j

(*R*)-*N*-methyl-3-phenyl-*N*-(3-phenyl-3-(*o*-tolyloxy)propyl)propanamide (6j)

Yield = 63%, ^{1}H NMR (400 MHz, CDCl$_3$) δ 7.34-7.16 (m, 9H), 7.11-7.01 (m, 1H), 7.04 (d, *J* = 7.6 Hz, 1H), 6.97-6.92 (m, 1H), 6.79-6.75 (m, 1H), 6.58-6.51 (m, 1H), 5.18-5.10 (m, 1H), 3.67-3.35 (m, 2H), 2.95-2.86 (m, 5H), 2.59-2.49 (m, 2H), 2.34-2.22 (m, 3H), 2.17-2.06 (m, 2H); ^{13}C NMR (101 MHz, CDCl$_3$) δ 172.3, 172.1, 155.8, 155.4, 141.7, 141.5, 141.4, 141.0, 130.9, 130.7, 128.9, 128.7, 128.6, 128.5, 128.5, 128.4, 127.9, 127.6, 127.0, 126.8, 126.7, 126.7, 126.2, 126.1, 125.8, 125.6, 120.7, 120.4, 112.8, 112.5, 77.6, 76.2, 46.3, 45.6, 37.6, 36.6, 35.9, 35.6, 34.8, 33.4, 31.6, 31.4, 29.8, 16.6, 16.5. HRMS calcd. (ESI) m/z for $C_{26}H_{29}NO_2Na$ [M+Na]+: 410.2091, found: 410.2106.

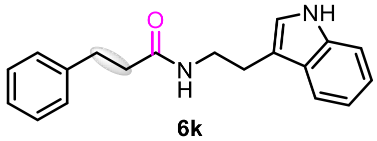

6k

N-(2-(1H-indol-3-yl)ethyl)-3-phenylpropanamide (6k)

Yield = 60%, ^{1}H NMR (400 MHz, $CDCl_3$) δ 8.37 (s, 1H), 7.53 (d, J = 8.0 Hz, 1H), 7.33 (d, J = 8.0 Hz, 1H), 7.26-7.23 (m, 2H), 7.19-7.01 (m, 5H), 6.81 (d, J = 2.0 Hz, 1H), 5.53 (t, J = 6.0 Hz, 1H), 3.55-3.50 (m, 2H), 2.93-2.85 (m, 4H), 2.38-2.35 (m, 2H); ^{13}C NMR (101 MHz, $CDCl_3$) δ 172.3, 140.9, 136.5, 128.6, 128.5, 127.3, 126.3, 122.2, 122.1, 119.4, 118.7, 112.7, 111.4, 39.8, 38.5, 31.8, 25.3. HRMS calcd. (ESI) m/z for $C_{19}H_{20}N_2ONa$ [M+Na]+: 315.1468, found: 315.1465.

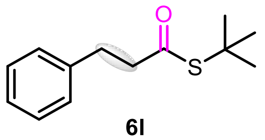

6l

S-(*tert*-butyl)-3-phenylpropanethioate (6l)

Yield = 76%, ^{1}H NMR (400 MHz, $CDCl_3$) δ 7.29-7.25 (m, 2H), 7.21-7.16 (m, 3H), 2.96-2.92 (m, 2H), 2.77-2.73 (m, 2H), 1.45 (s, 9H); ^{13}C NMR (101 MHz, $CDCl_3$) δ 199.6, 140.4, 128.6, 128.5, 126.4, 48.1, 46.1, 31.6, 29.9. HRMS calcd. (ESI) m/z for $C_{13}H_{18}OSNa$ [M+Na]+: 245.0971, found: 249.0994.

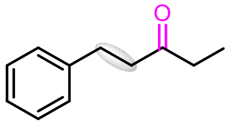

6m

1-phenylpentan-3-one (6m)

Yield = 94%, ^{1}H NMR (400 MHz, $CDCl_3$) δ 7.29-7.25 (m, 2H), 7.18-7.17 (m, 3H), 2.91-2.88 (m, 2H), 2.74-2.70 (m, 2H), 2.43-2.37 (m, 2H), 1.03 (t, J = 7.2 Hz, 3H); ^{13}C NMR (101 MHz, $CDCl_3$) δ 210.8, 141.3, 128.6, 128.4, 126.2, 44.0, 36.2, 29.9, 7.8. HRMS calcd. (ESI) m/z for $C_{11}H_{14}ONa$ [M+Na]+: 185.0937, found: 185.0943.

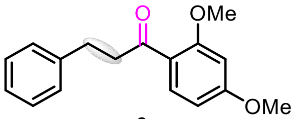

6n

1-(2,4-dimethoxyphenyl)-3-phenylpropan-1-one (6n)

Yield = 72%, ^{1}H NMR (400 MHz, $CDCl_3$) δ 7.82 (t, J = 8.8 Hz, 1H), 7.31-7.19 (m, 5H), 6.54-6.52 (m, 1H), 6.45 (d, J = 2.0 Hz, 1H), 3.86 (d, J = 5.2 Hz, 6H), 3.29-3.25 (m, 2H), 3.02-2.98 (m, 2H); ^{13}C NMR (101 MHz, $CDCl_3$) δ 199.6, 164.5, 160.9, 142.2, 132.9, 128.6, 128.5, 126.0, 121.2, 105.2, 98.5, 55.7, 55.6, 45.5, 30.8. HRMS calcd. (ESI) m/z for $C_{17}H_{18}O_3Na$ [M+Na]+: 293.1148, found: 293.1163.

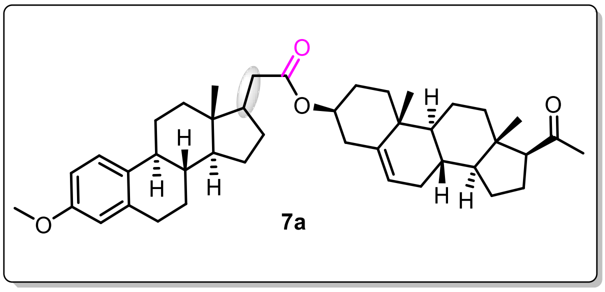

(3*S*,8*S*,9*S*,10*R*,13*S*,14*S*,17*S*)-17-acetyl-10,13-dimethyl-2,3,4,7,8,9,10,11,12,13,14,15,16,17-tetradecahydro-1H-cyclopenta[a]phenanthren-3-yl-2-((8*S*,9*S*,13*R*,14*S*)-3-methoxy-13-methyl-7,8,9,11,12,13,14,15,16,17-decahydro-6H-cyclopenta[a]phenanthren-17-yl)acetate (7a)

Yield = 78%, ^{1}H NMR (400 MHz, $CDCl_3$) δ 7.20 (d, J = 8.0 Hz, 1H), 6.71-6.84 (m, 1H), 6.63-6.62 (m, 1H), 5.39-5.38 (m, 1H), 4.65-4.61 (m, 1H), 3.77 (s, 3H), 2.86-2.84 (m, 2H), 2.56-2.51 (m, 1H), 2.36-2.29 (m, 3H), 2.27-2.17 (m, 2H), 2.12 (s, 3H), 2.06-1.97 (m, 3H), 1.92-1.85 (m, 4H), 1.82-1.79 (m, 1H), 1.69-1.58 (m, 5H), 1.50-1.47 (m, 4H), 1.41-1.37 (m, 3H), 1.32-1.29 (m, 1H), 1.27-1.22 (m, 3H), 1.21-1.15 (m, 3H), 1.05-0.97 (m, 6H), 0.64-0.63 (m, 6H); ^{13}C NMR (101 MHz, $CDCl_3$) δ 209.7, 176.9, 173.4, 157.5, 139.8, 138.1, 132.8, 126.3, 122.4, 122.3, 113.8, 111.5, 73.6, 63.7, 56.9, 55.2, 54.4, 53.7, 50.5, 49.9, 48.7, 47.1, 44.0, 44.0, 43.8, 42.5, 38.9, 38.8, 38.8, 38.2, 37.5, 37.2, 37.1, 36.7, 35.8, 32.1, 31.9, 31.8, 31.6, 29.9, 29.9, 29.6, 28.3, 28.0, 27.9, 27.8, 26.5, 26.4, 24.5, 24.4, 24.3, 22.9, 21.1, 19.4, 13.3, 13.2, 12.7, 12.7. HRMS calcd. (ESI) m/z for $C_{42}H_{58}O_4Na$ [M+Na]+: 649.4227, found: 649.4216.

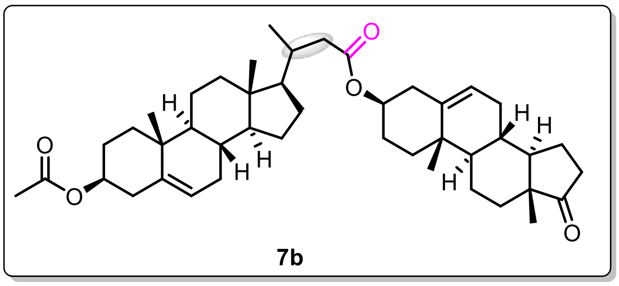

(3*R*,8*S*,9*R*,10*S*,13*R*,14*R*)-10,13-dimethyl-17-oxo-2,3,4,7,8,9,10,11,12,13,14,15,16,17-tetradecahydro-1H-cyclopenta[a]phenanthren-3-yl-3-((3*S*,8*S*,9*S*,10*R*,13*R*,14*S*,17*R*)-3-acetoxy-10,13-dimethyl-2,3,4,7,8,9,10,11,12,13,14,15,16,17-tetradecahydro-1H-cyclopenta[a]phenanthren-17-yl)butanoate (7b)

Yield = 70%, ^{1}H NMR (400 MHz, $CDCl_3$) δ 5.42-5.41 (m, 1H), 5.38-5.37 (m, 1H), 4.64-4.59 (m, 1H), 2.50-2.42 (m, 1H), 2.35-2.30 (m, 3H), 2.26- 2.18 (m, 1H), 2.14-2.07 (m, 3H), 2.04 (s, 3H), 1.99-1.93 (m, 4H), 1.89-1.83 (m, 5H), 1.70-1.64 (m, 4H), 1.62-1.53 (m, 5H), 1.51-1.43 (m, 4H), 1.25 (s, 9H), 1.05 (s, 3H), 1.02 (s, 3H), 0.99 (s, 3H), 0.90-0.88 (m, 4H), 0.85-0.83 (m, 3H), 0.72-0.71 (m, 3H); ^{13}C NMR (101 MHz, $CDCl_3$) δ 221.3, 173.2, 170.7, 140.1, 139.8, 122.7, 121.9, 74.1, 73.5, 56.8, 56.1, 51.8, 50.2, 50.0, 47.6, 42.5, 42.4, 42.1, 39.7, 38.3, 38.2, 37.1, 36.9, 36.7, 36.0, 34.0, 31.9, 31.7, 31.6, 31.5, 30.9, 28.3, 27.8, 24.3, 22.0, 21.6, 21.1, 20.4, 19.6, 19.5, 19.4, 13.7, 12.2, 12.0. HRMS calcd. (ESI) m/z for $C_{44}H_{64}O_5Na$ [M+Na]+: 695.4646, found: 695.4639.

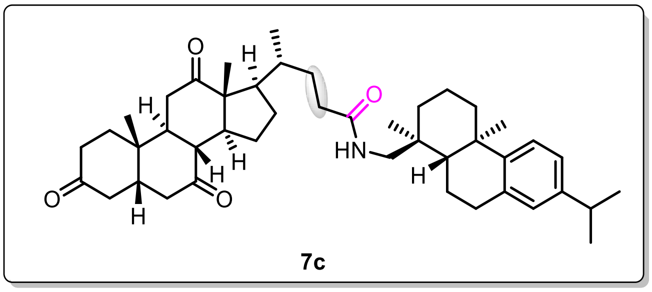

(*R*)-4-((5*S*,8*R*,9*S*,10*S*,13*R*,14*S*,17*R*)-10,13-dimethyl-3,7,12-trioxohexadecahydro-1H-cyclopenta[a]phenanthren-17-yl)-*N*-(((1*S*,4a*R*,10a*S*)-7-isopropyl-1,4a-dimethyl-1,2,3,4,4a,9,10,10a-octahydrophenanthren-1-yl)methyl)pentanamide (7c)

Yield = 63%, ^{1}H NMR (400 MHz, $CDCl_3$) δ 7.16 (d, J = 8.4 Hz, 1H), 7.00-6.98

(m, 1H), 6.89 (s, 1H), 5.59-5.55 (m, 1H), 3.24-3.19 (m, 1H), 3.14-3.09 (m, 1H), 2.94-2.85 (m, 3H), 2.84-2.75 (m, 3H), 2.37-2.34 (m, 1H), 2.31-2.24 (m, 5H), 2.20-2.18 (m, 2H), 2.15-2.07 (m, 3H), 2.03-1.96 (m, 4H), 1.90-1.77 (m, 5H), 1.72-1.60 (m, 3H), 1.39 (s, 5H), 1.36-1.35 (m, 1H), 1.32-1.31 (m, 1H), 1.25 (s, 2H), 1.23 (s, 3H), 1.21-1.21 (m, 6H), 1.01 (s, 3H), 0.93 (s, 3H), 0.89-0.84 (m, 1H), 0.81 (d, J = 6.4 Hz, 3H); ^{13}C NMR (101 MHz, $CDCl_3$) δ 212.2, 209.3, 209.0, 173.5, 147.2, 145.7, 134.9, 127.0, 124.3, 123.9, 57.0, 51.8, 49.8, 49.0, 46.9, 45.6, 45.5, 45.4, 45.1, 42.9, 38.7, 38.4, 37.5, 37.4, 36.6, 36.3, 36.1, 35.5, 35.3, 33.9, 33.5, 31.3, 30.3, 27.7, 25.4, 25.2, 24.1, 22.0, 19.0, 18.8, 18.8, 18.7, 11.9. HRMS calcd. (ESI) m/z for $C_{44}H_{63}O_4Na$ [M+K]+: 694.4358, found: 694.4515.

2.6. Copies of ^{1}H and ^{13}C NMR Spectra.

Supplementary Fig. 14. ^{1}H NMR Spectra (400 MHz, $CDCl_3$) of **crude 2-phenylpropanoyl chloride 2a**

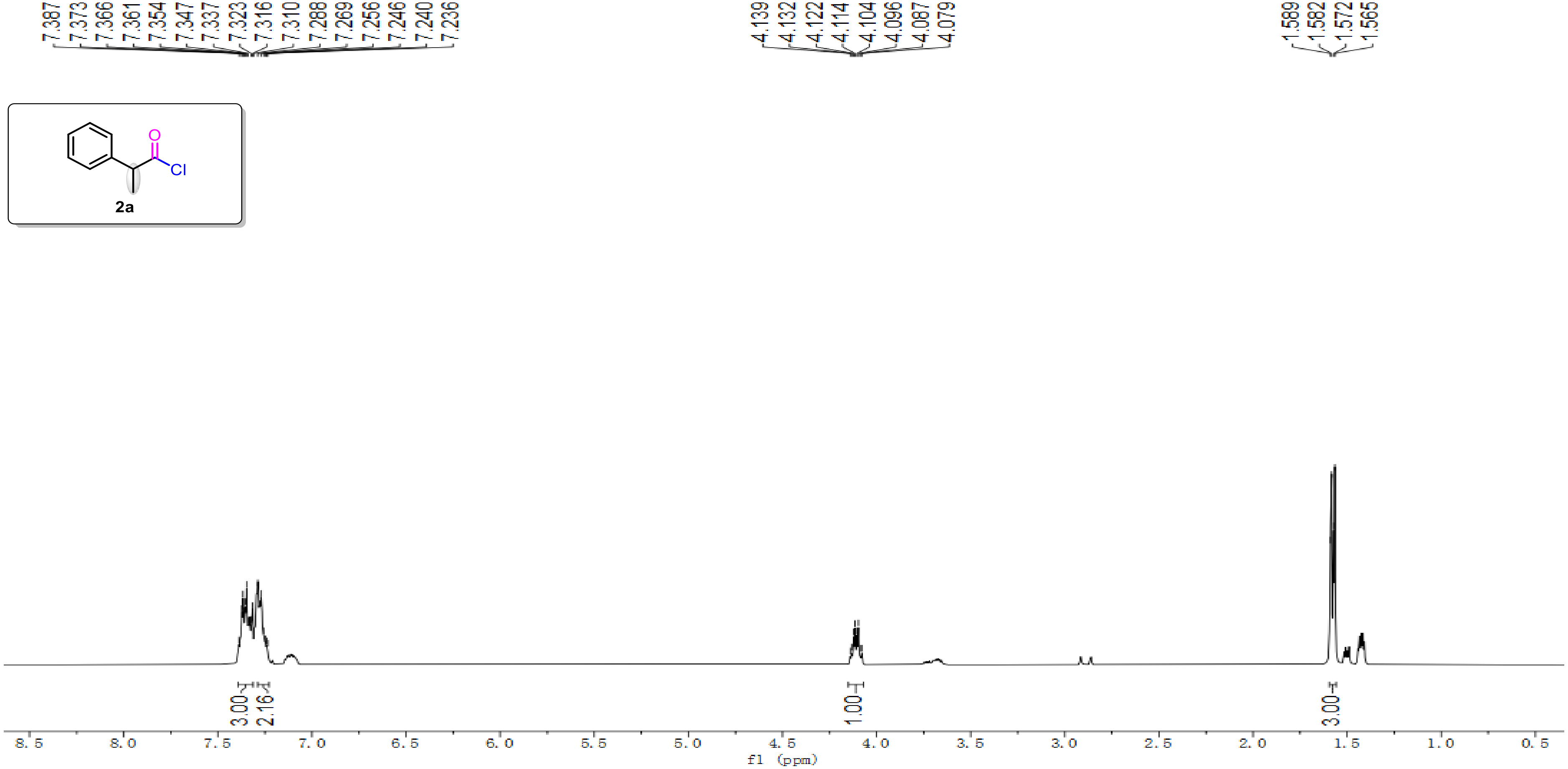

Supplementary Fig. 15. ^{13}C NMR Spectra (101 MHz, $CDCl_3$) of **crude 2-phenylpropanoyl chloride 2a**

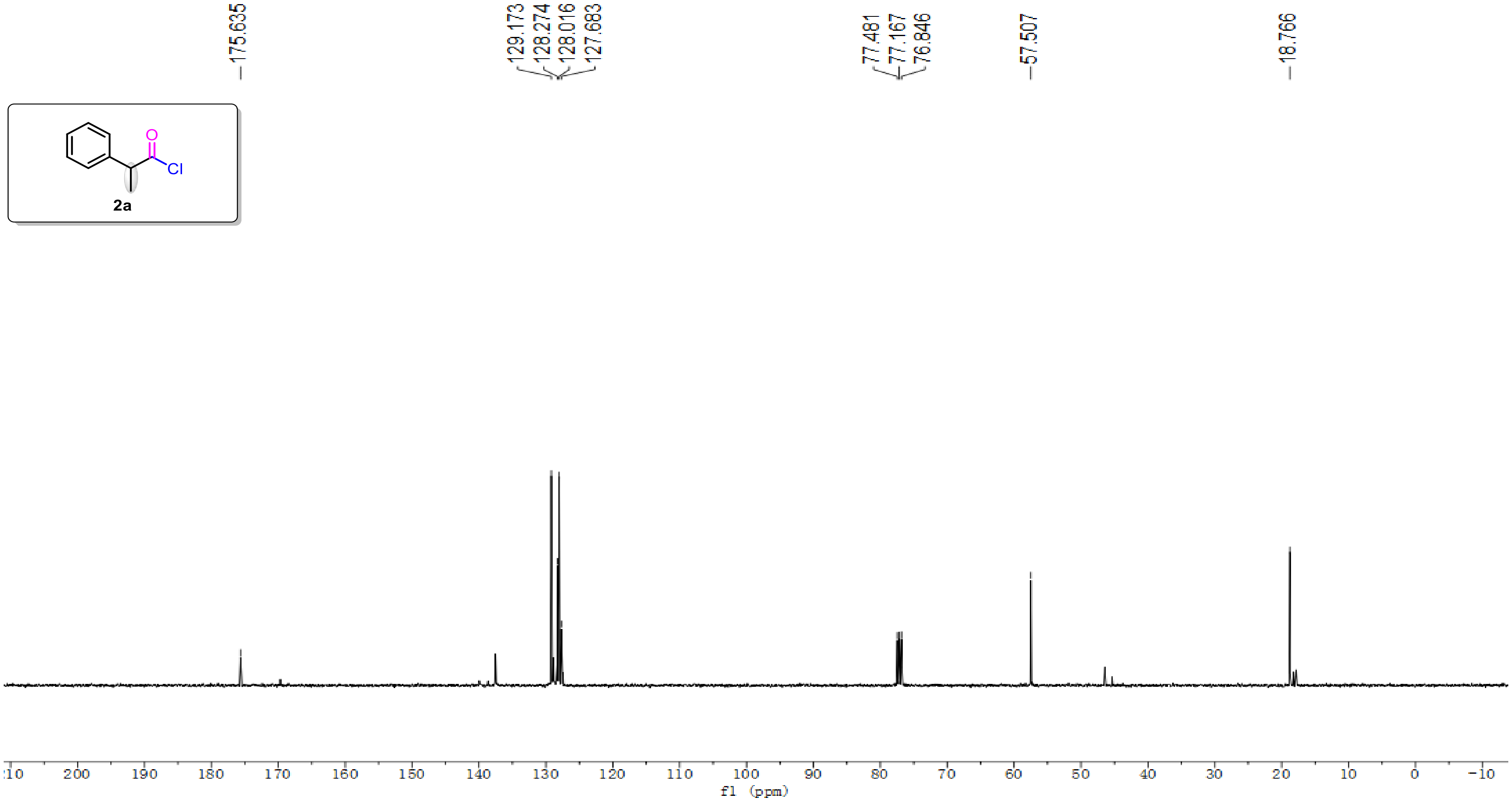

Supplementary Fig. 16. ^{1}H NMR Spectra (400 MHz, $CDCl_3$) of **2a'**

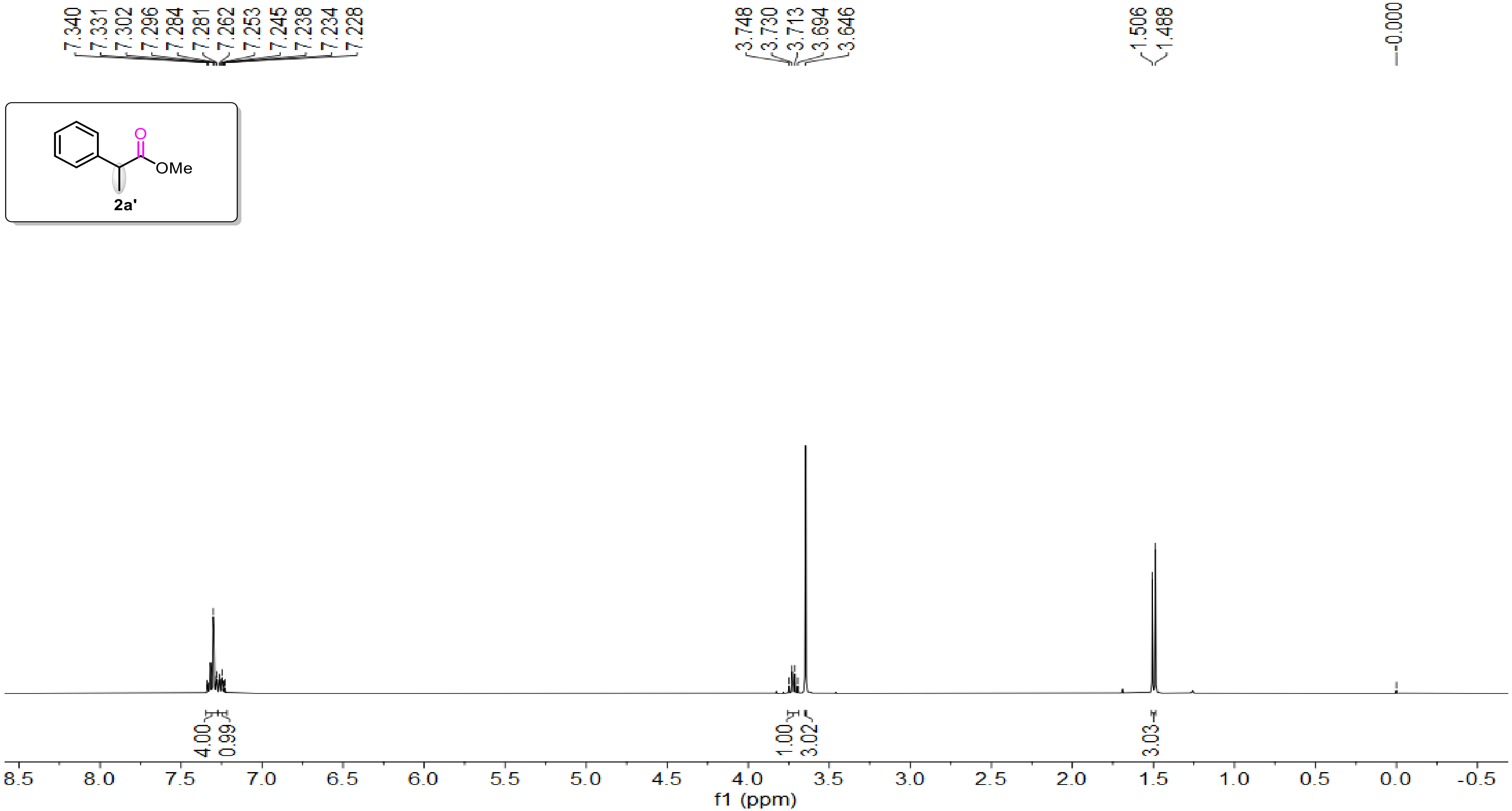

Supplementary Fig. 17. ^{13}C NMR Spectra (101 MHz, $CDCl_3$) of **2a'**

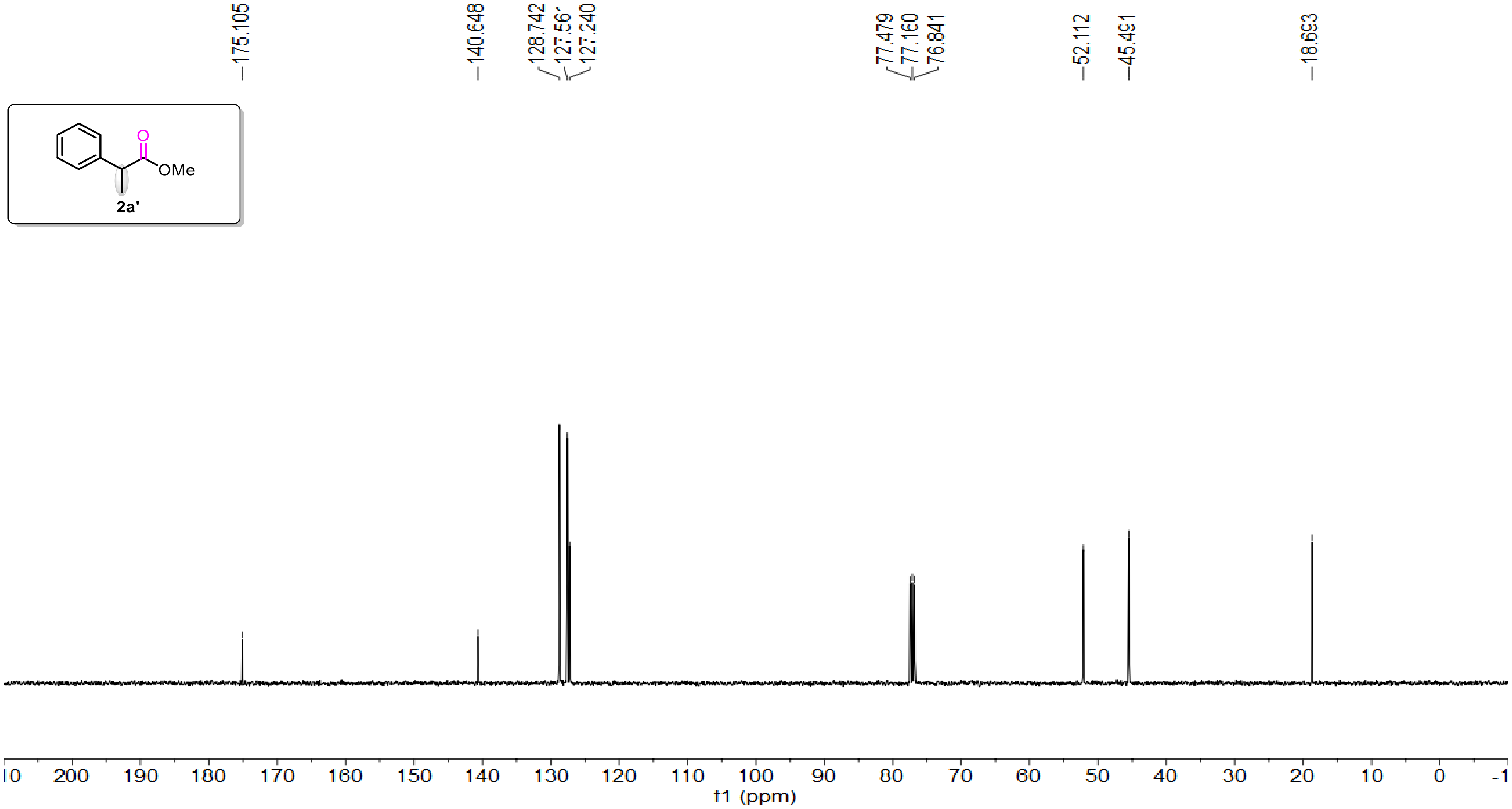

Supplementary Fig. 18. ^{1}H NMR Spectra (400 MHz, $CDCl_3$) of **2b'**

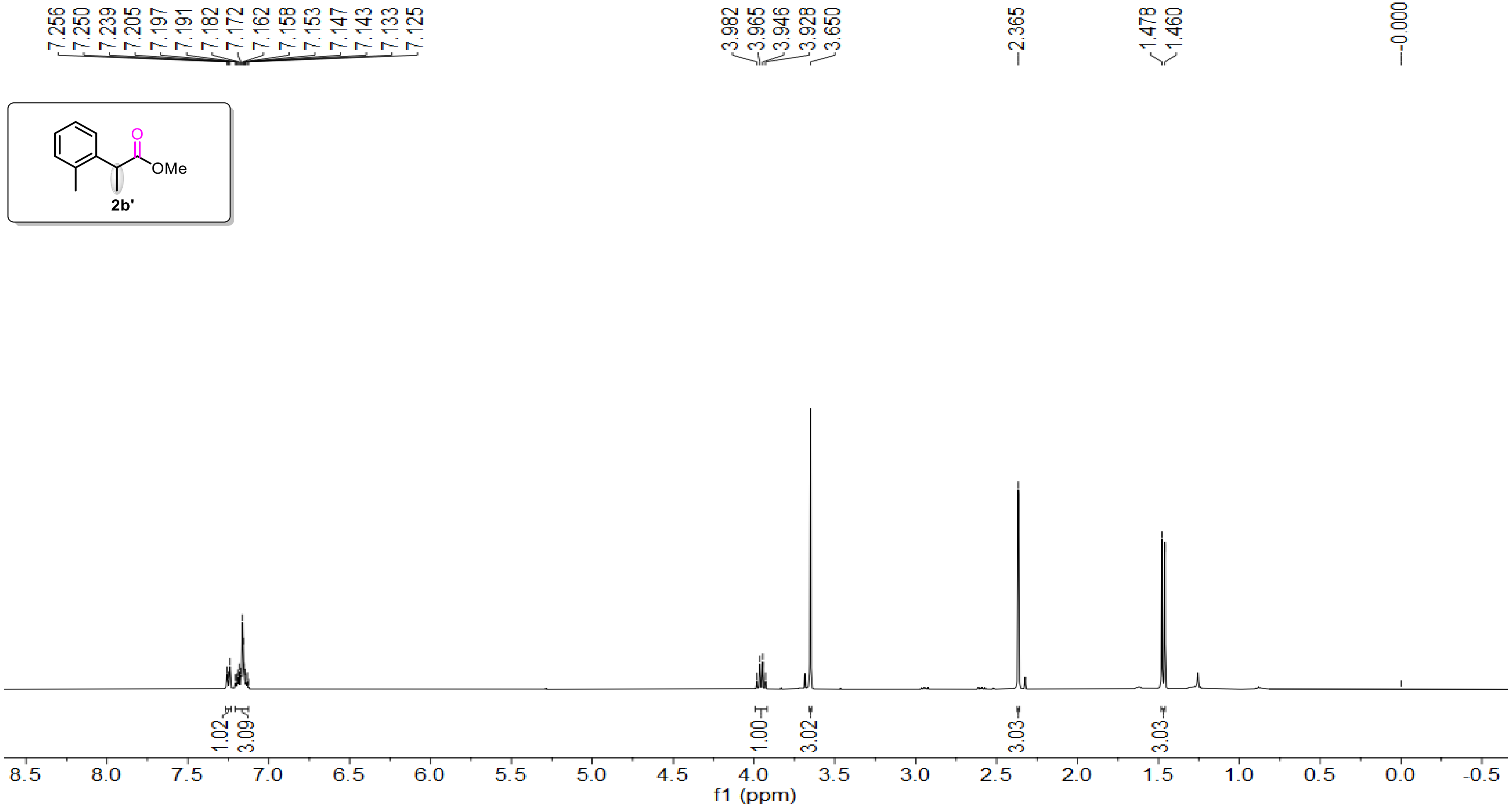

Supplementary Fig. 19. ^{13}C NMR Spectra (101 MHz, $CDCl_3$) of **2b'**

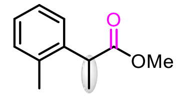

2b'

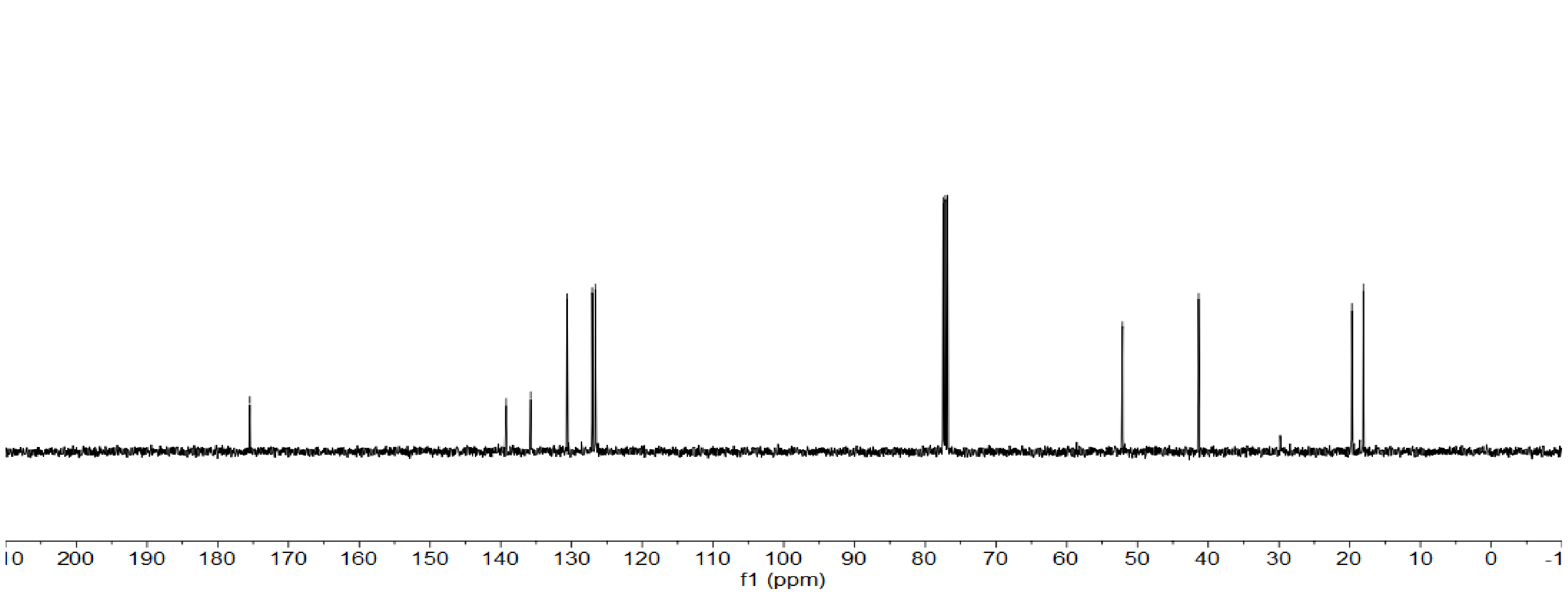

Supplementary Fig. 20. ^{1}H NMR Spectra (400 MHz, $CDCl_3$) of **2c'**

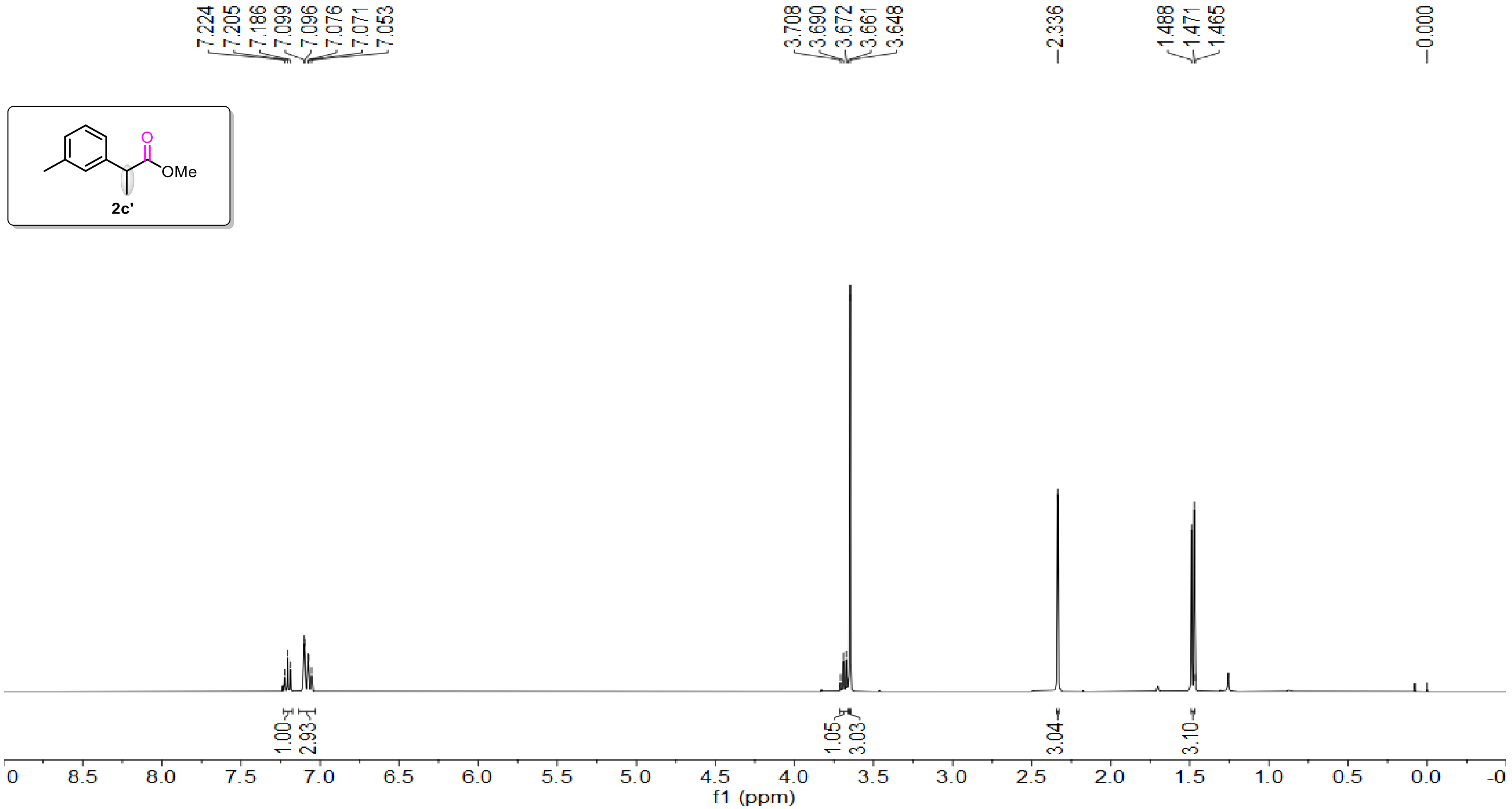

Supplementary Fig. 21. ^{13}C NMR Spectra (101 MHz, $CDCl_3$) of **2c'**

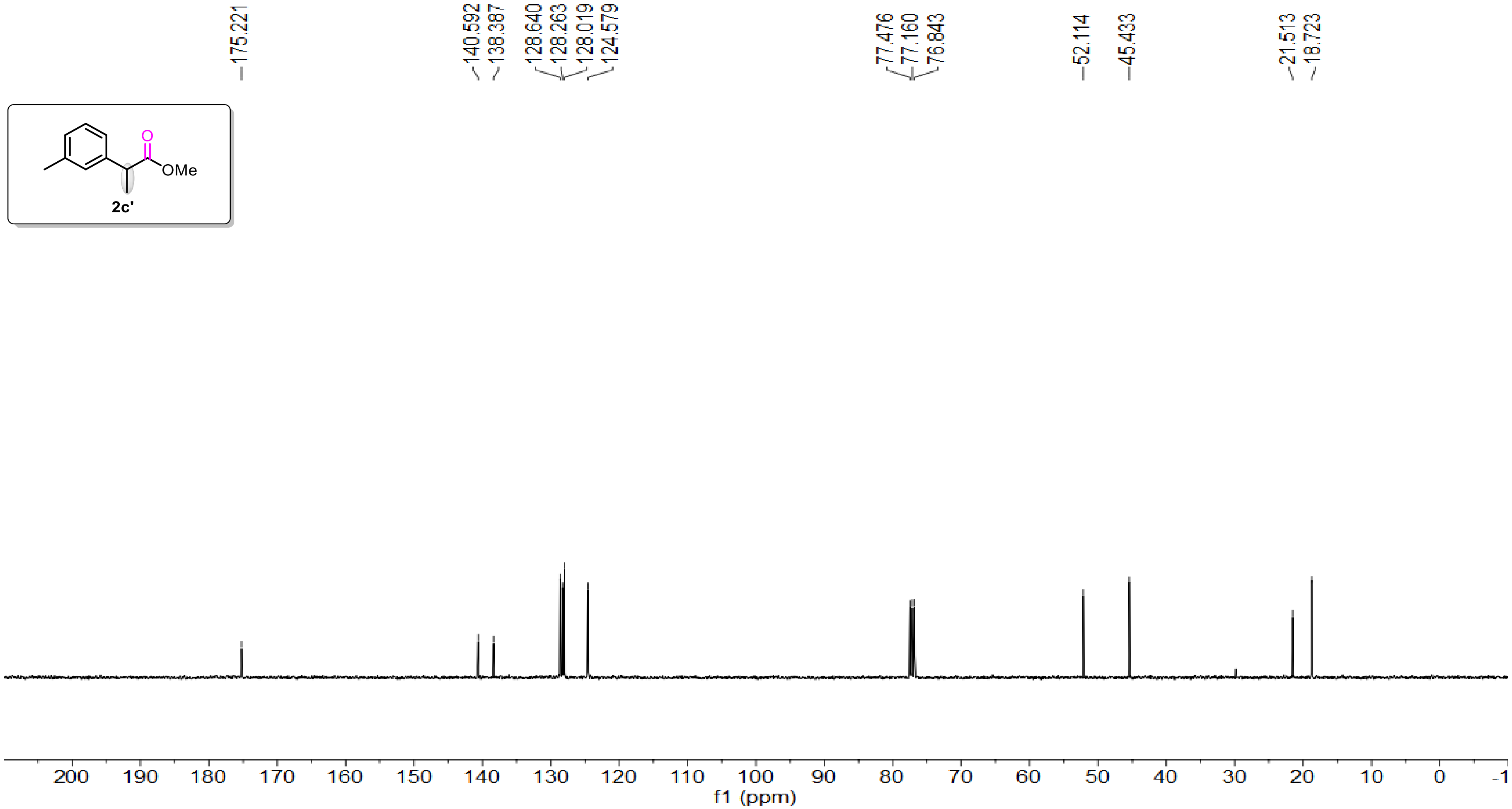

Supplementary Fig. 22. ^{1}H NMR Spectra (400 MHz, $CDCl_3$) of **2d'**

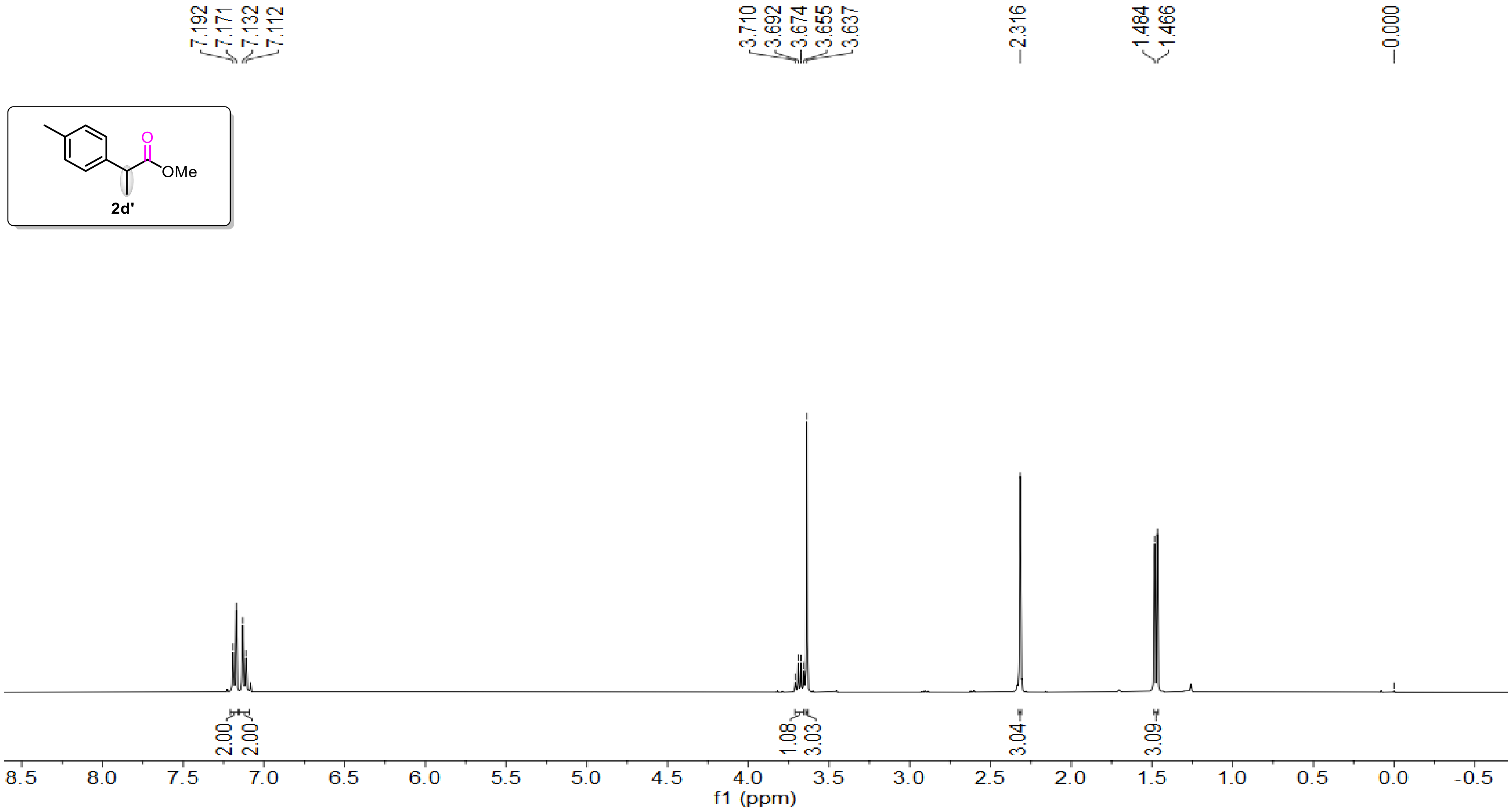

Supplementary Fig. 23. ^{13}C NMR Spectra (101 MHz, $CDCl_3$) of **2d'**

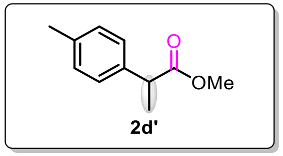

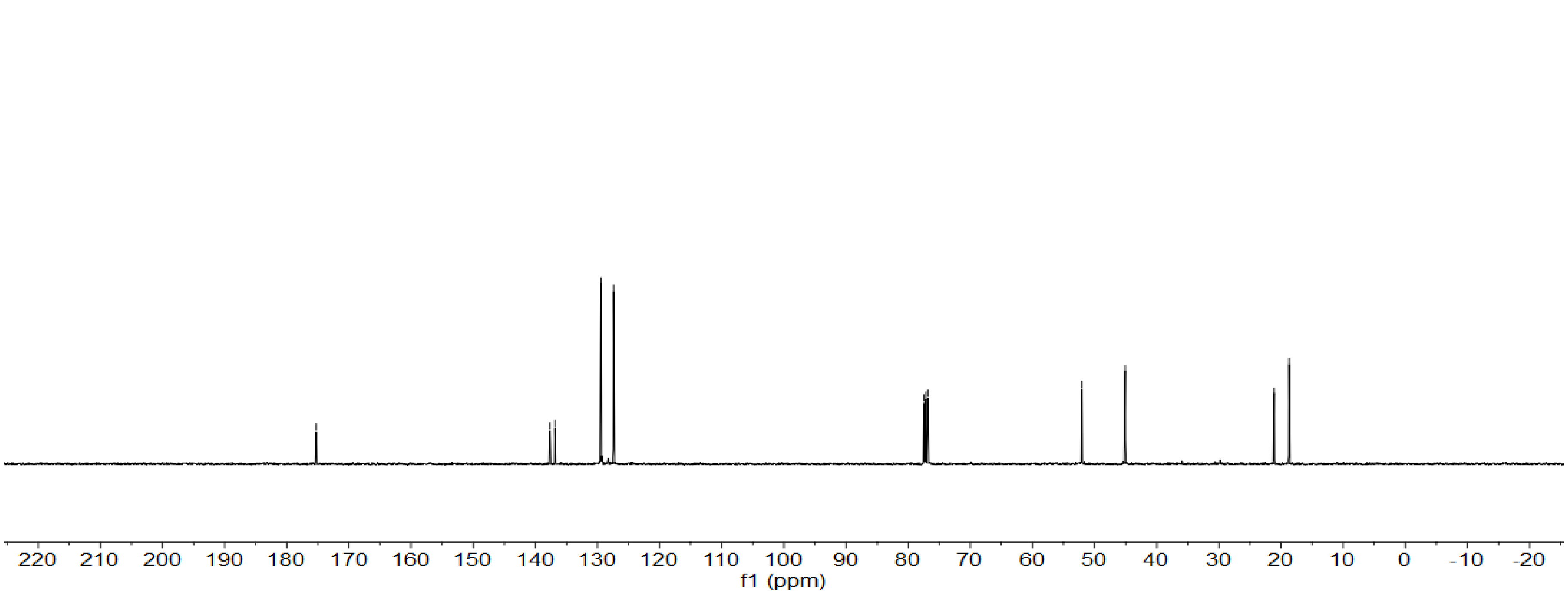

Supplementary Fig. 24. ^{1}H NMR Spectra (400 MHz, $CDCl_3$) of **2e'**

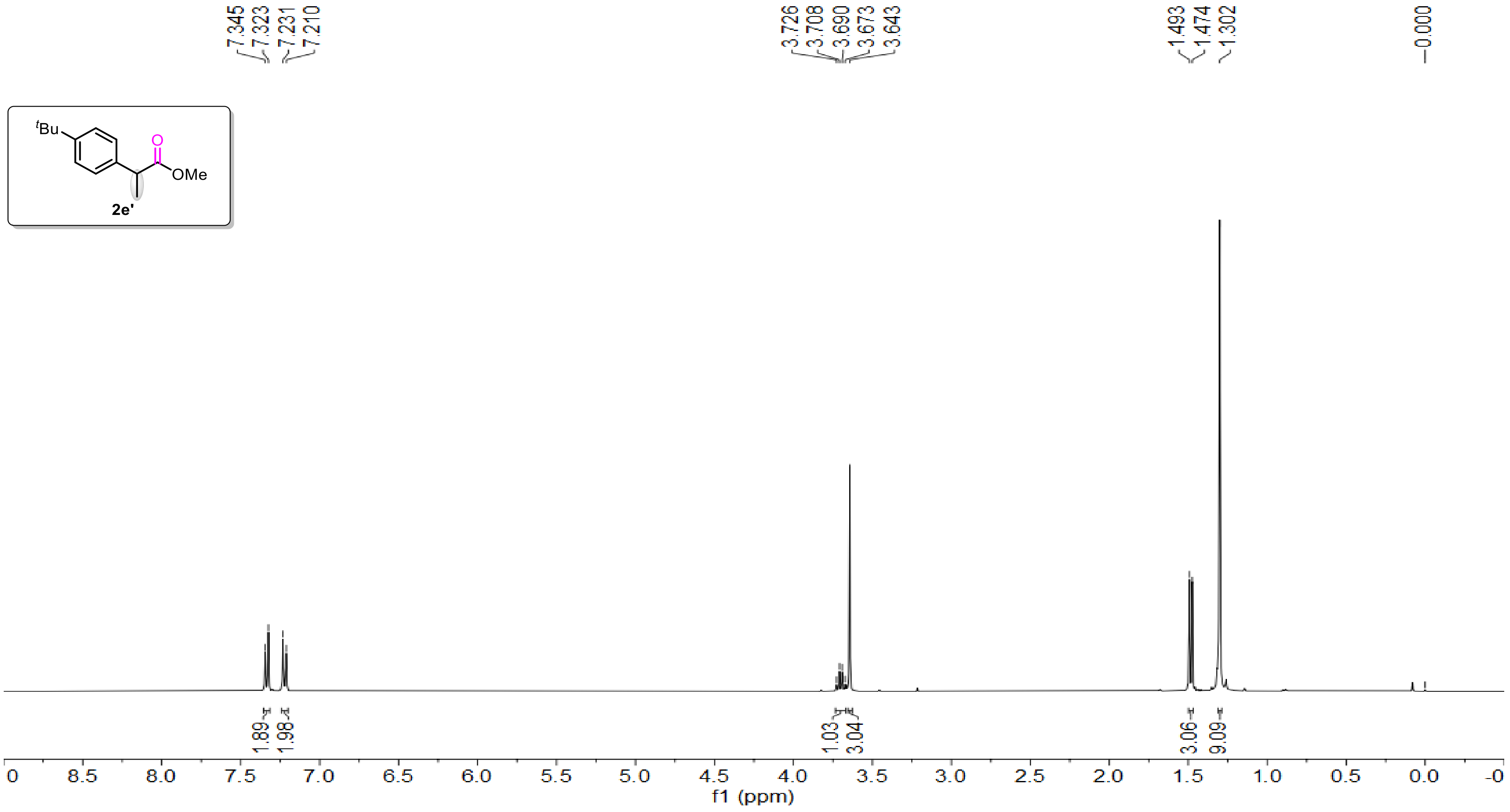

Supplementary Fig. 25. ^{13}C NMR Spectra (101 MHz, $CDCl_3$) of **2e'**

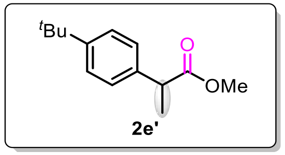

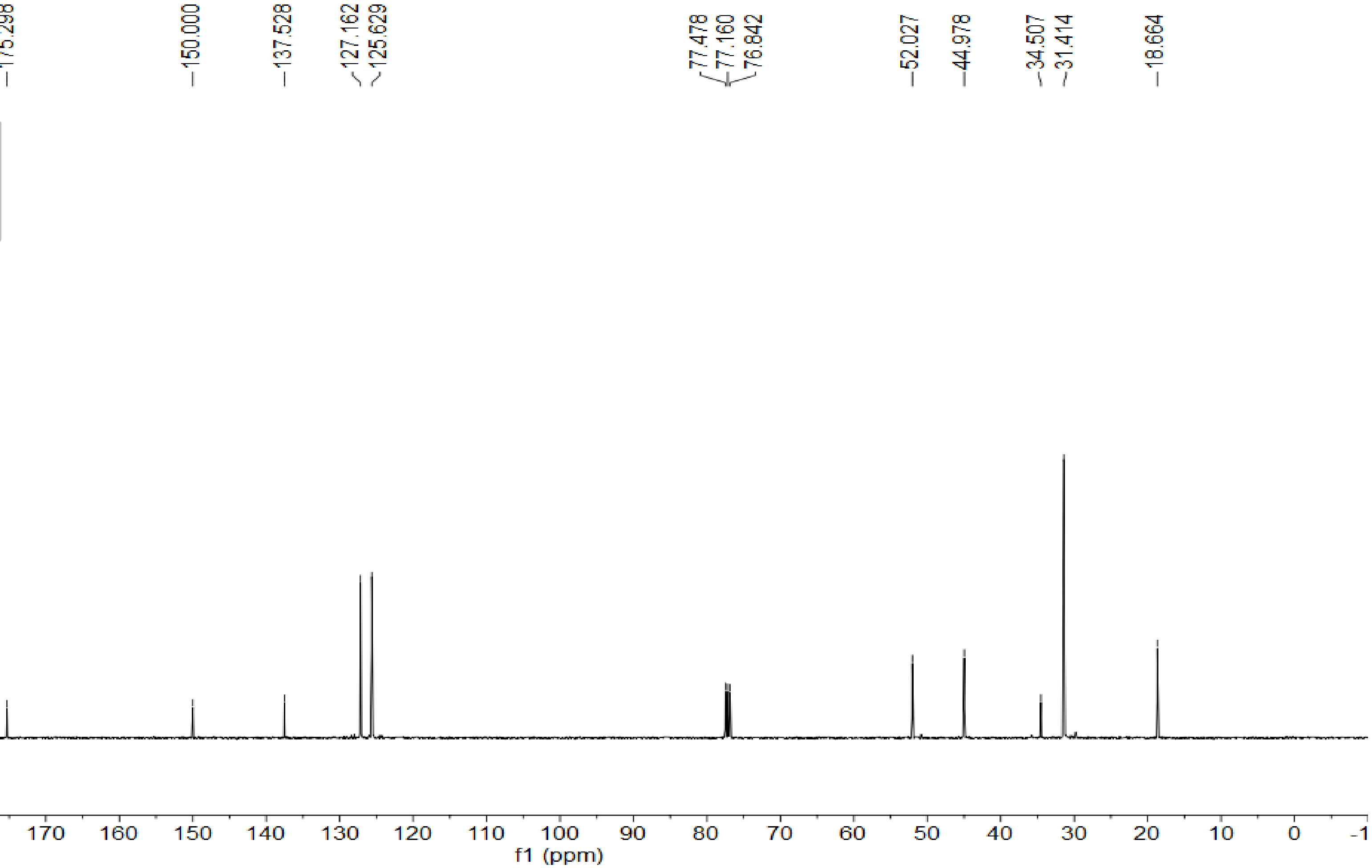

Supplementary Fig. 26. ^{1}H NMR Spectra (400 MHz, $CDCl_3$) of **2f'**

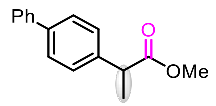

2f'

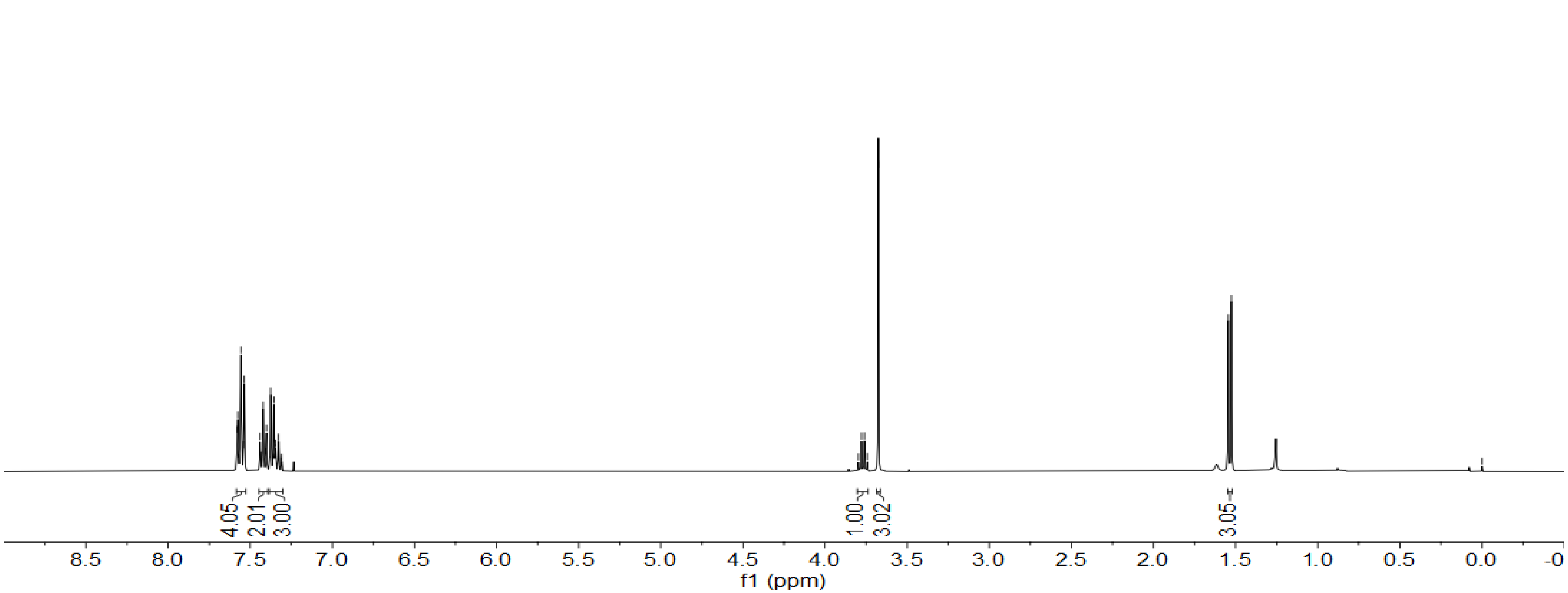

Supplementary Fig. 27. ^{13}C NMR Spectra (101 MHz, CDCl$_3$) of **2f'**

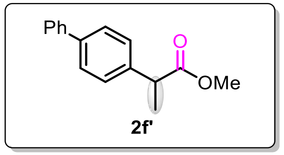

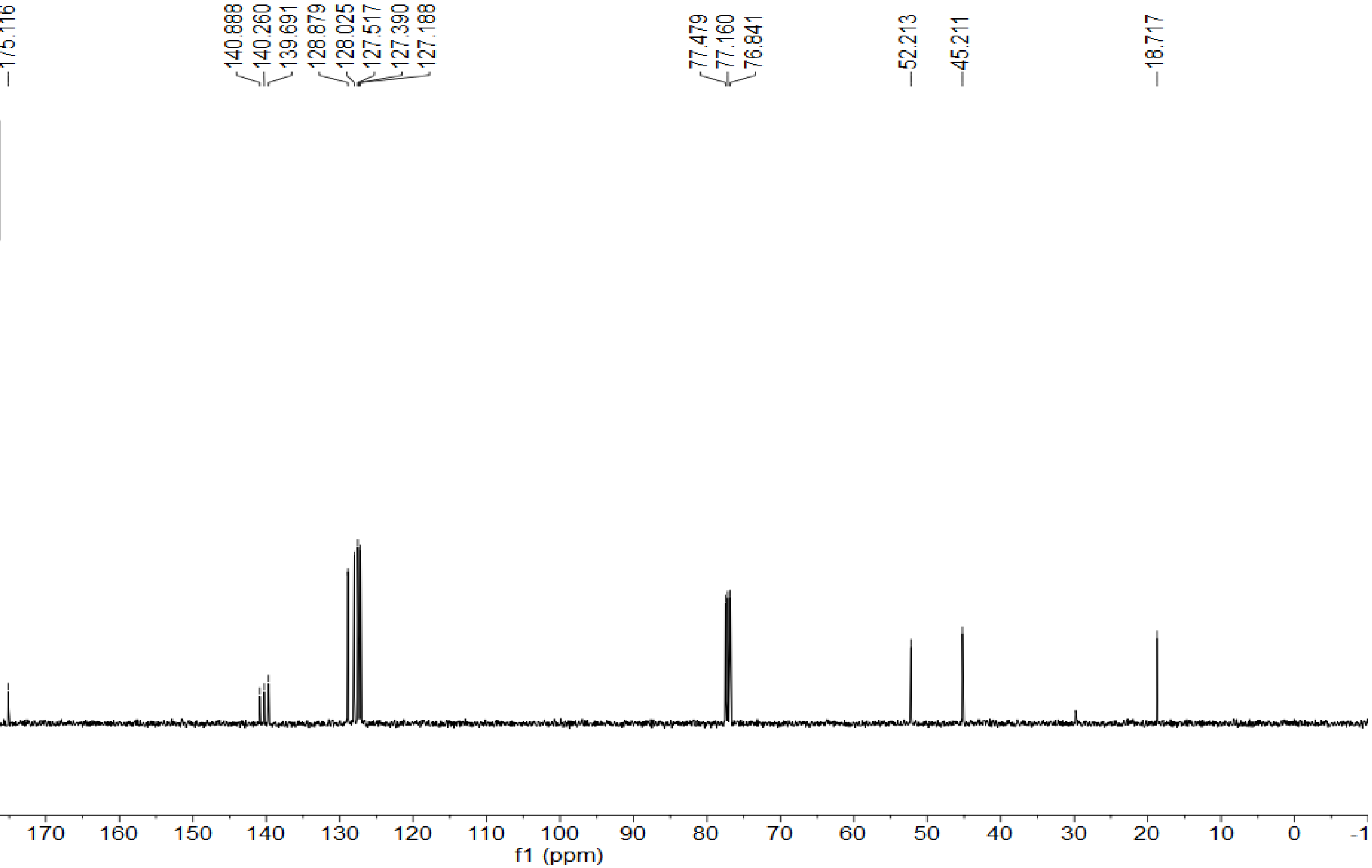

Supplementary Fig. 28. ^{1}H NMR Spectra (400 MHz, $CDCl_3$) of **2g'**

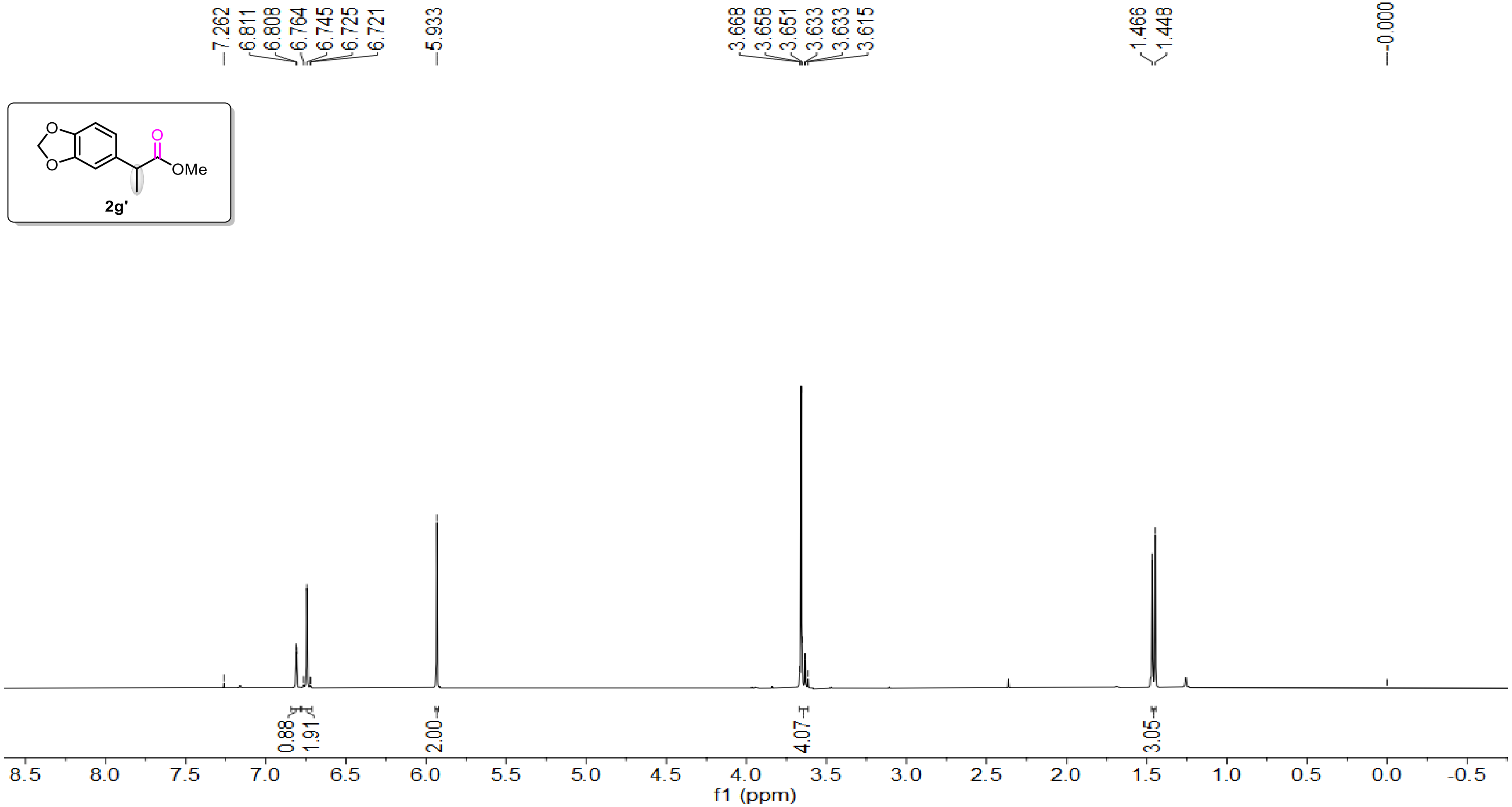

Supplementary Fig. 29. ^{13}C NMR Spectra (101 MHz, $CDCl_3$) of **2g'**

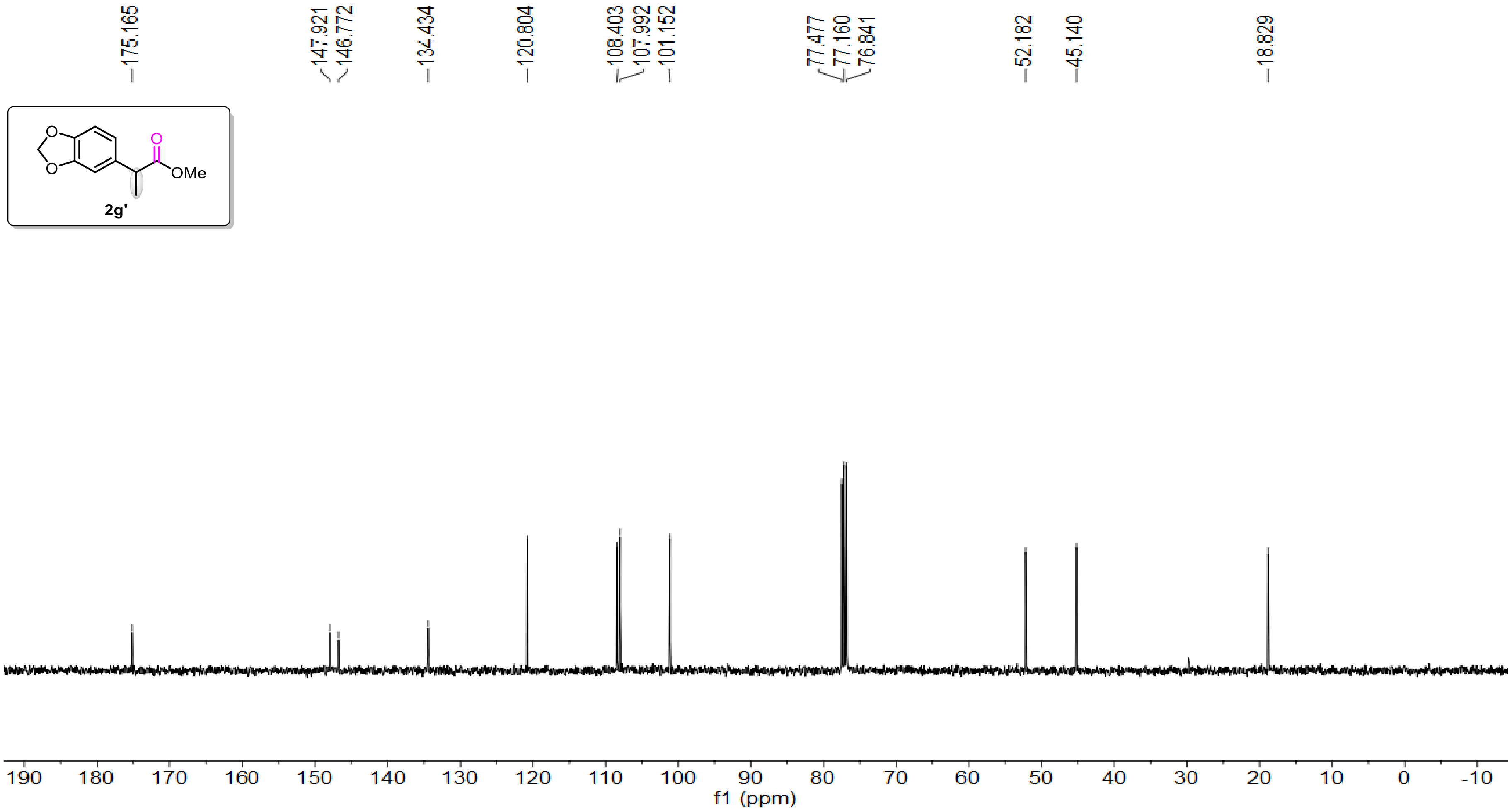

Supplementary Fig. 30. ^{1}H NMR Spectra (400 MHz, $CDCl_3$) of **2h'**

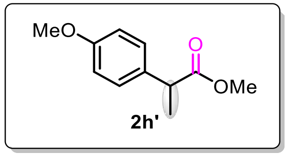

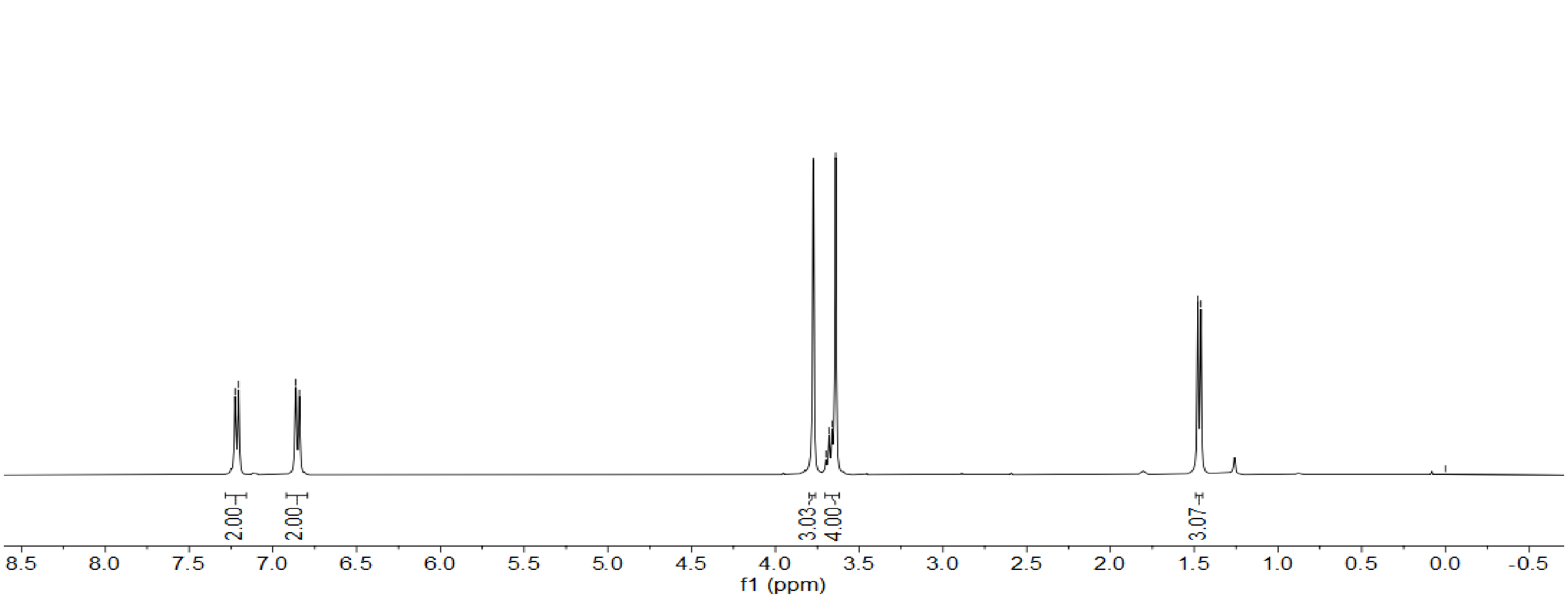

Supplementary Fig. 31. ^{13}C NMR Spectra (101 MHz, $CDCl_3$) of **2h'**

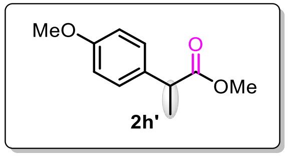

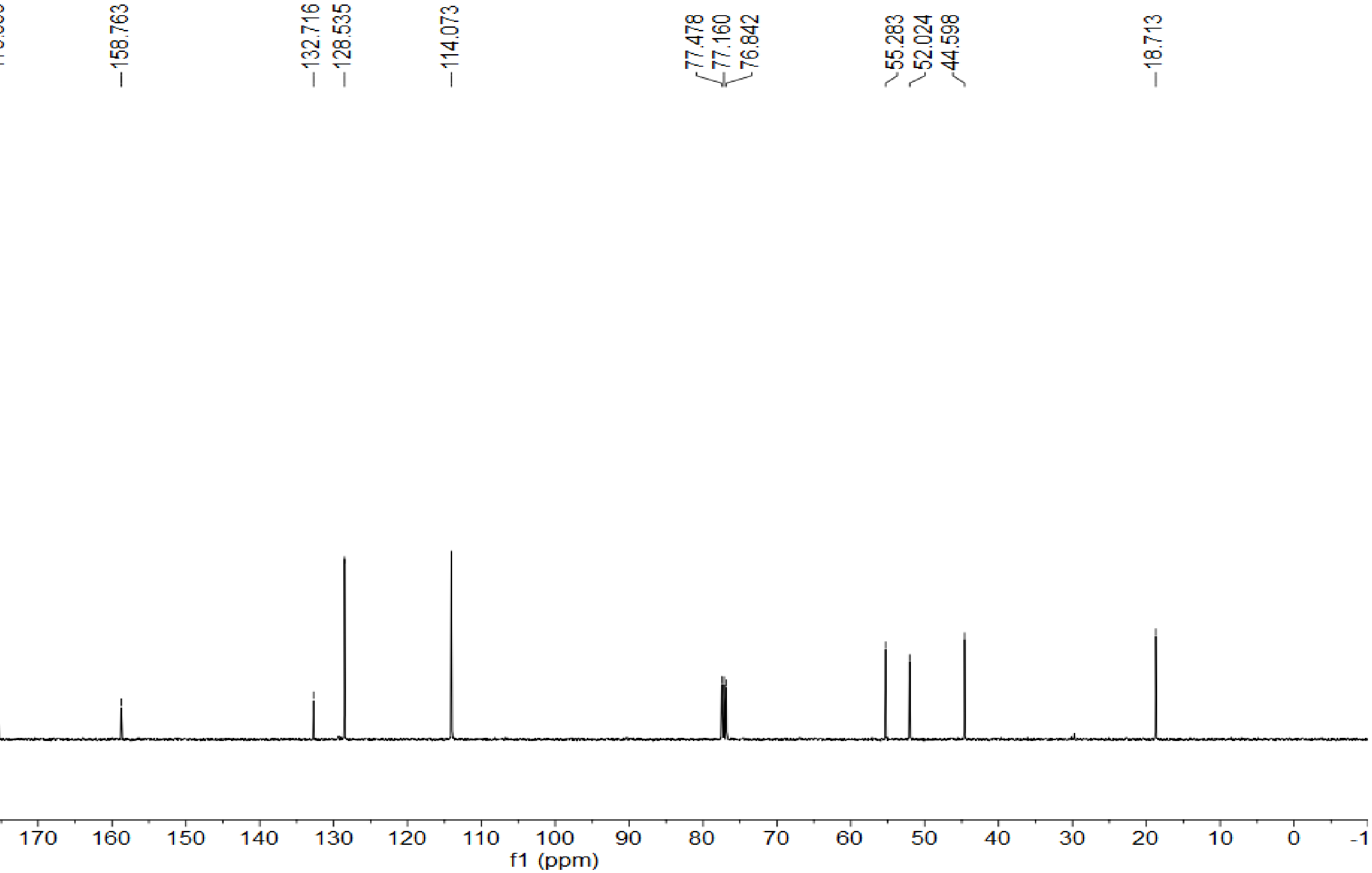

Supplementary Fig. 32. ^{1}H NMR Spectra (400 MHz, $CDCl_3$) of **2i'**

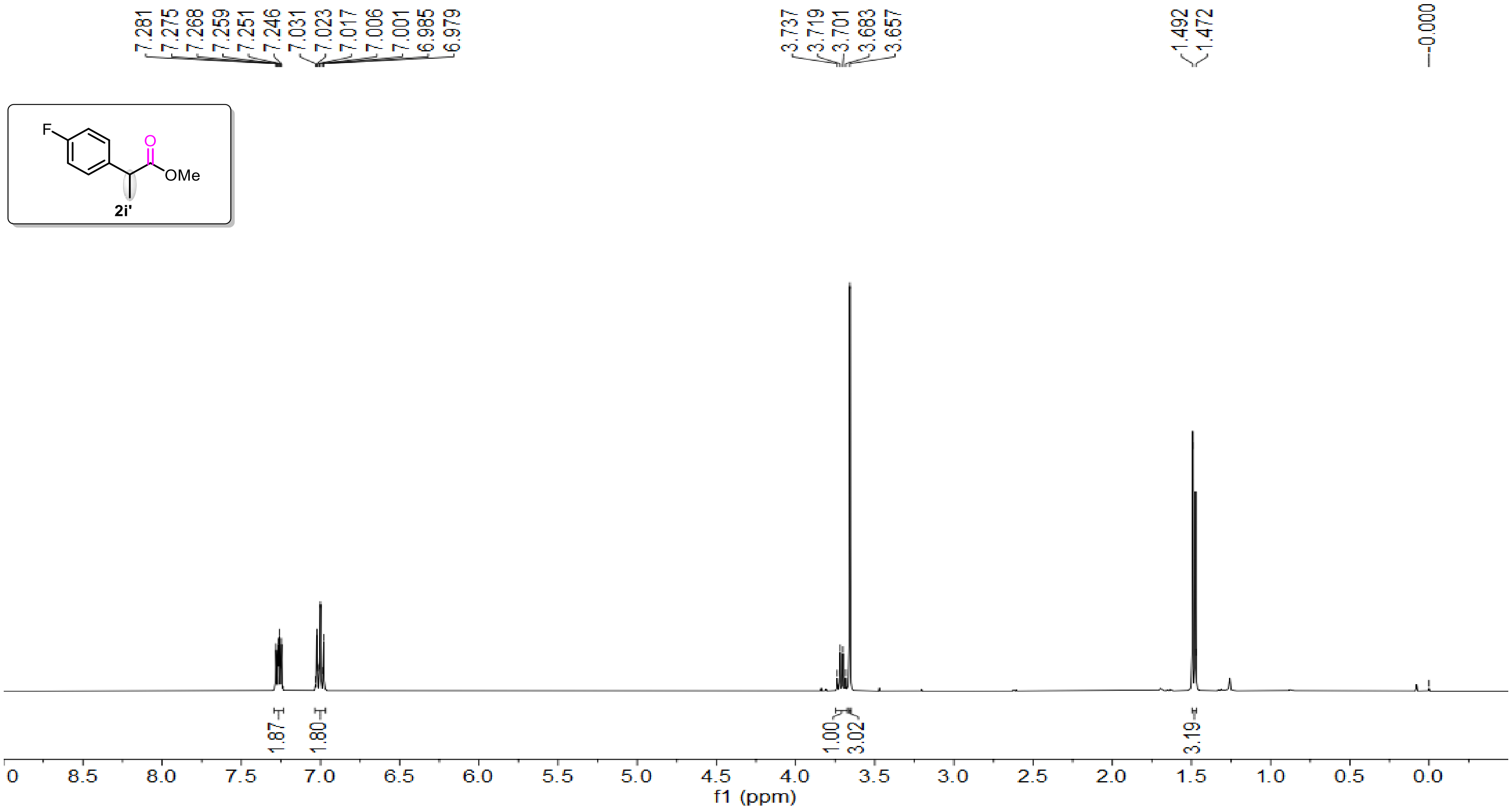

Supplementary Fig. 33. ^{13}C NMR Spectra (101 MHz, $CDCl_3$) of **2i'**

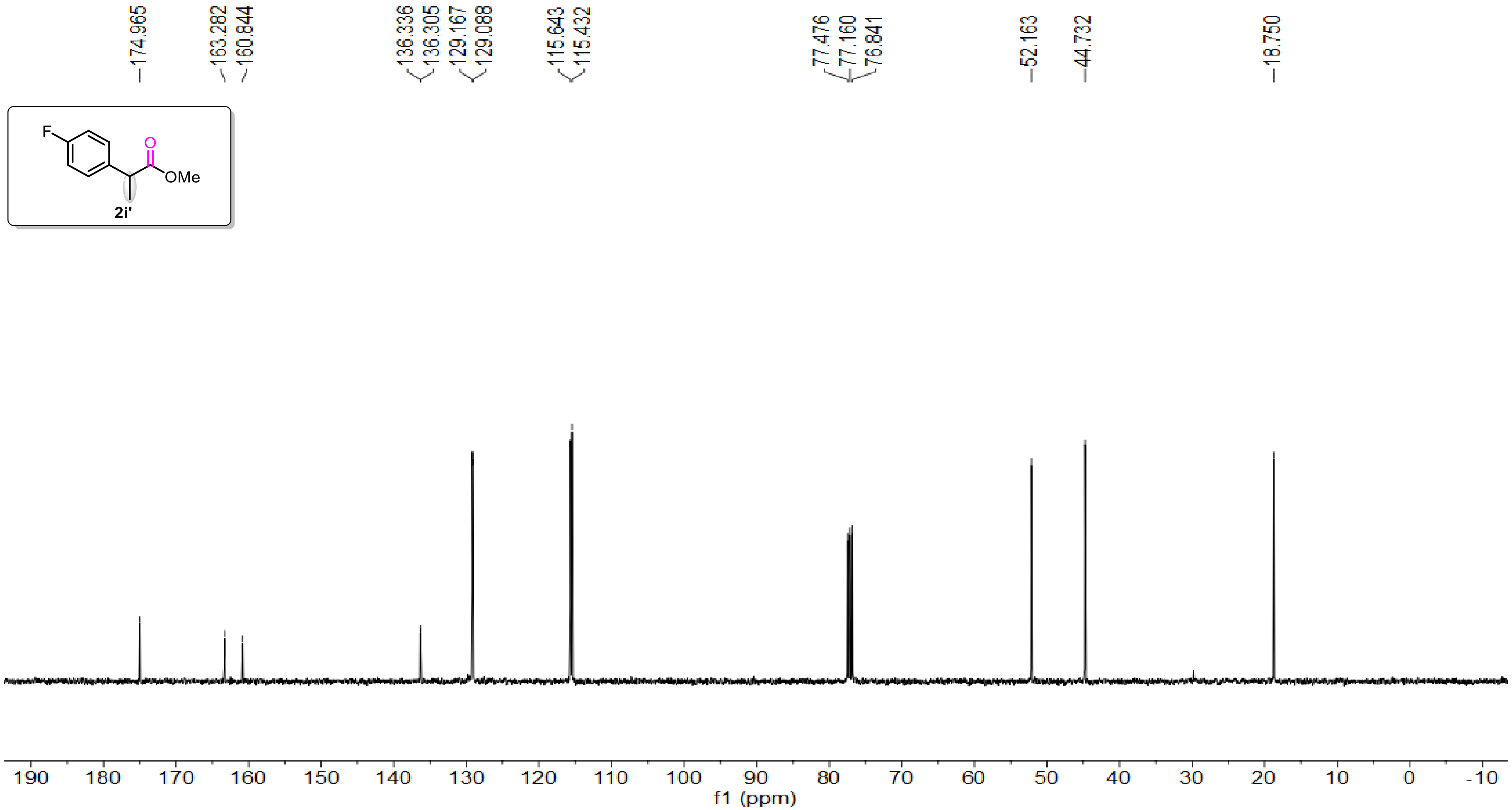

Supplementary Fig. 34. ^{1}H NMR Spectra (400 MHz, $CDCl_3$) of **2j'**

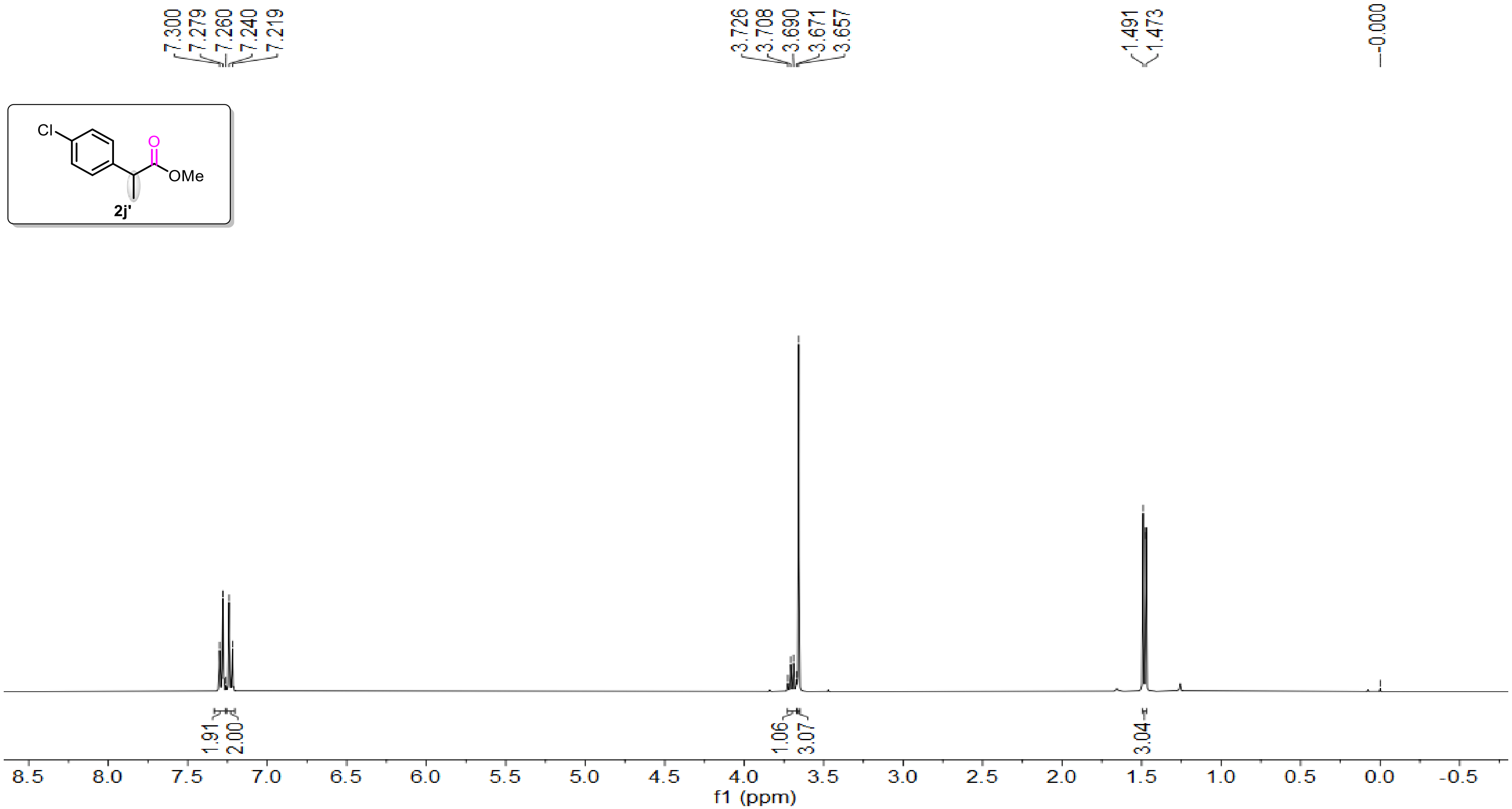

Supplementary Fig. 35. ^{13}C NMR Spectra (101 MHz, $CDCl_3$) of **2j'**

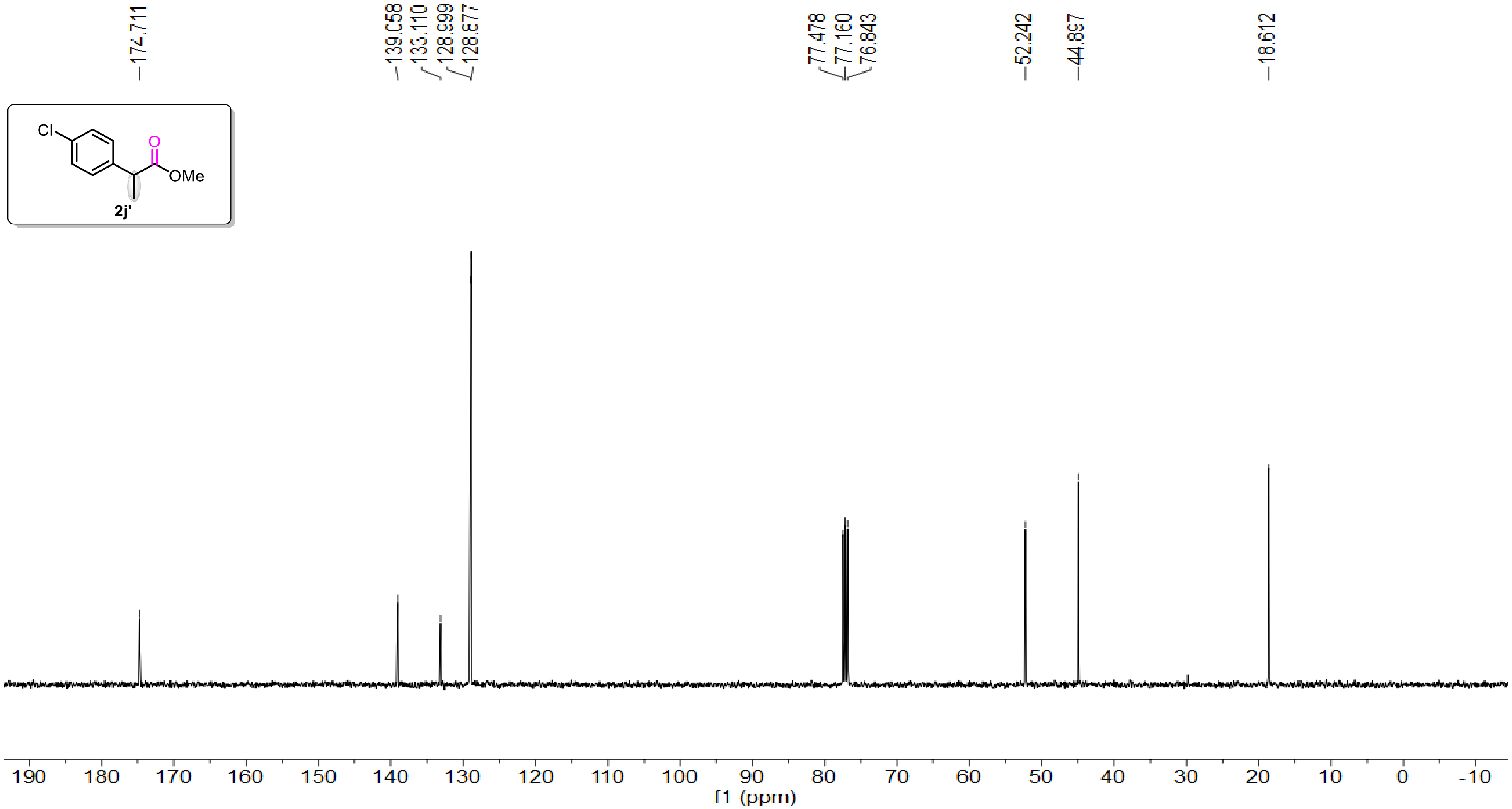

Supplementary Fig. 36. ^{1}H NMR Spectra (400 MHz, $CDCl_3$) of **2k'**

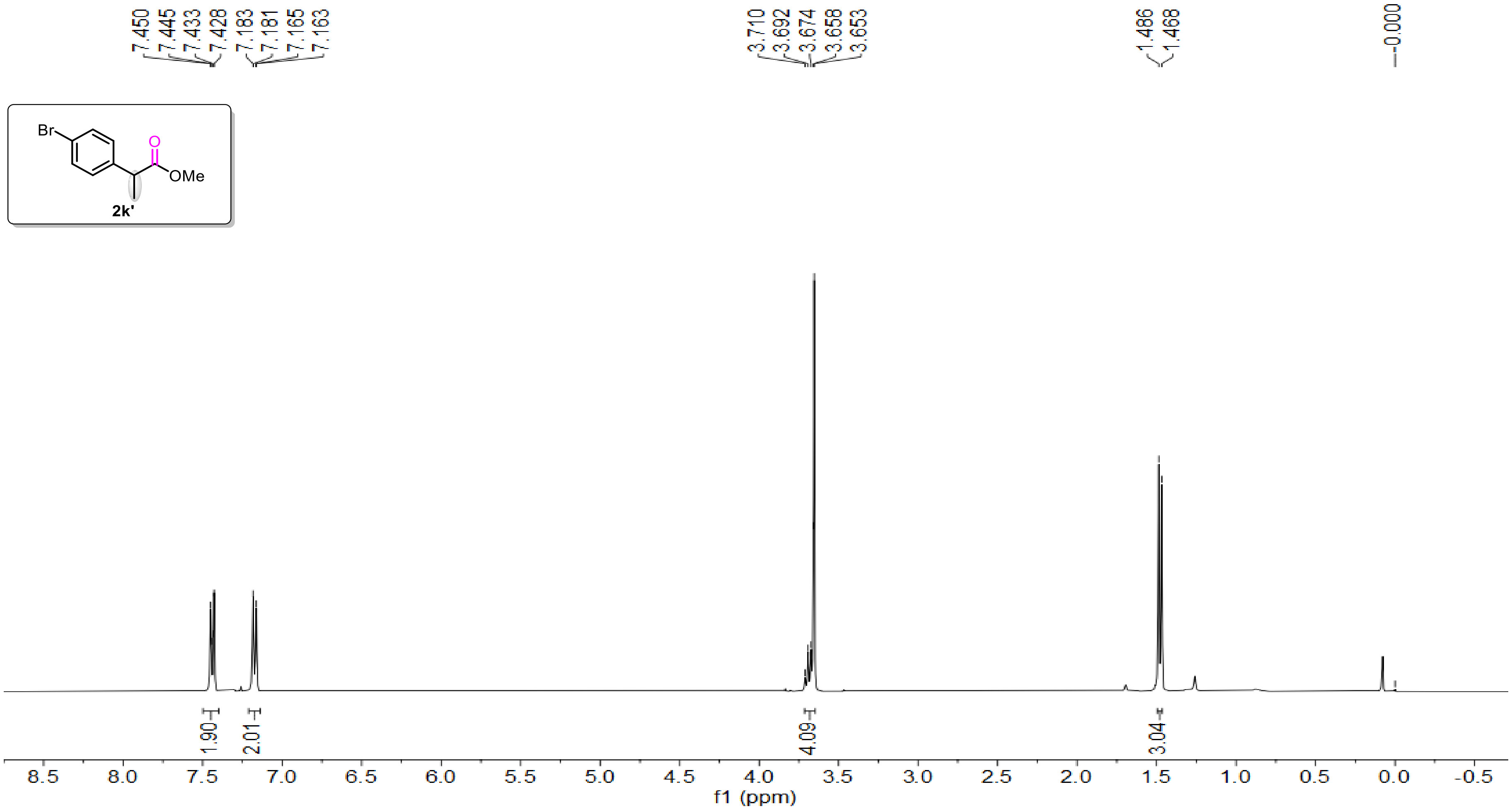

Supplementary Fig. 37. ^{13}C NMR Spectra (101 MHz, $CDCl_3$) of **2k'**

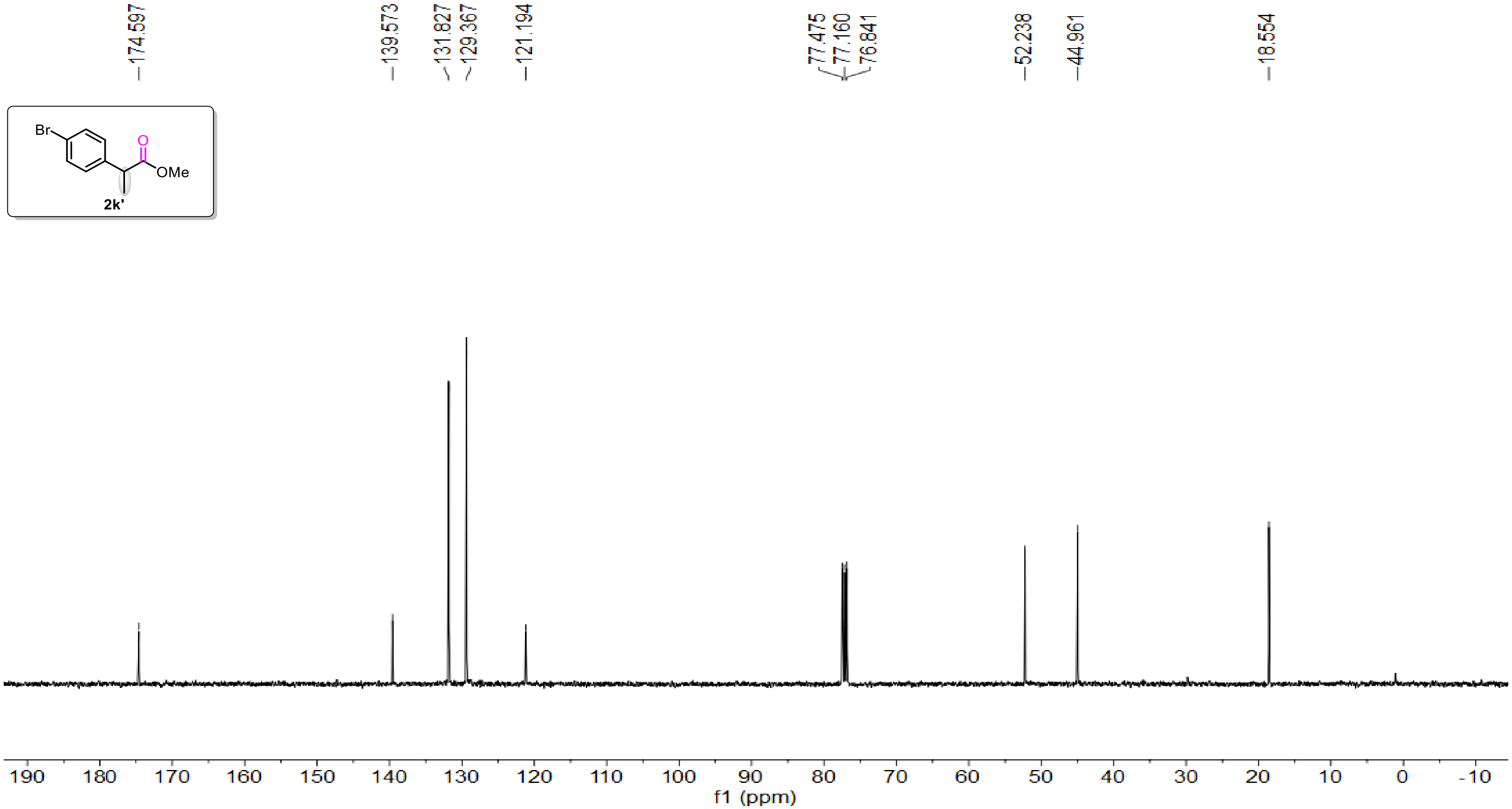

Supplementary Fig. 38. ^{1}H NMR Spectra (400 MHz, $CDCl_3$) of **2l'**

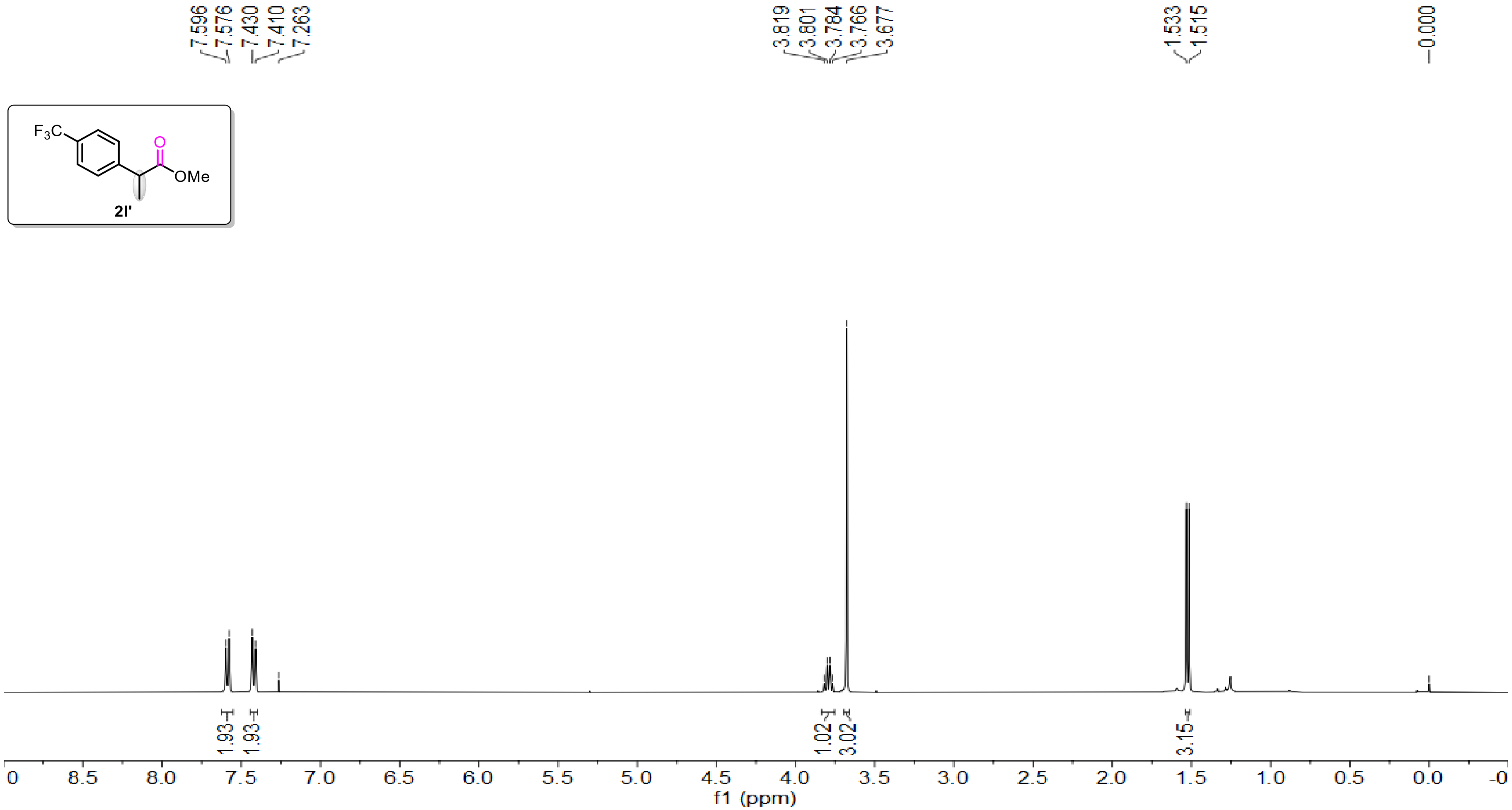

Supplementary Fig. 39. ^{13}C NMR Spectra (101 MHz, $CDCl_3$) of **2l'**

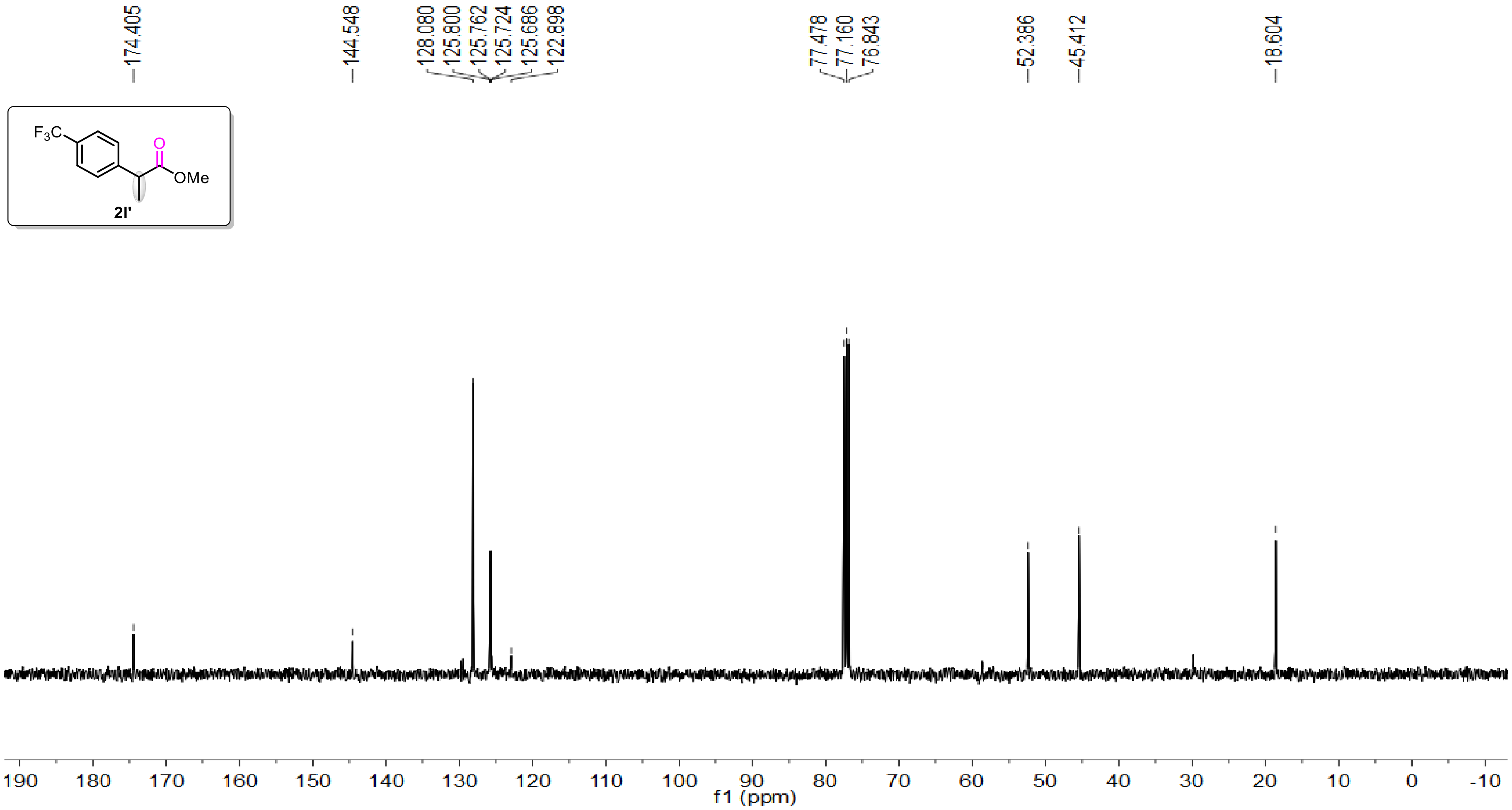

Supplementary Fig. 40. ^{1}H NMR Spectra (400 MHz, $CDCl_3$) of **2m'**

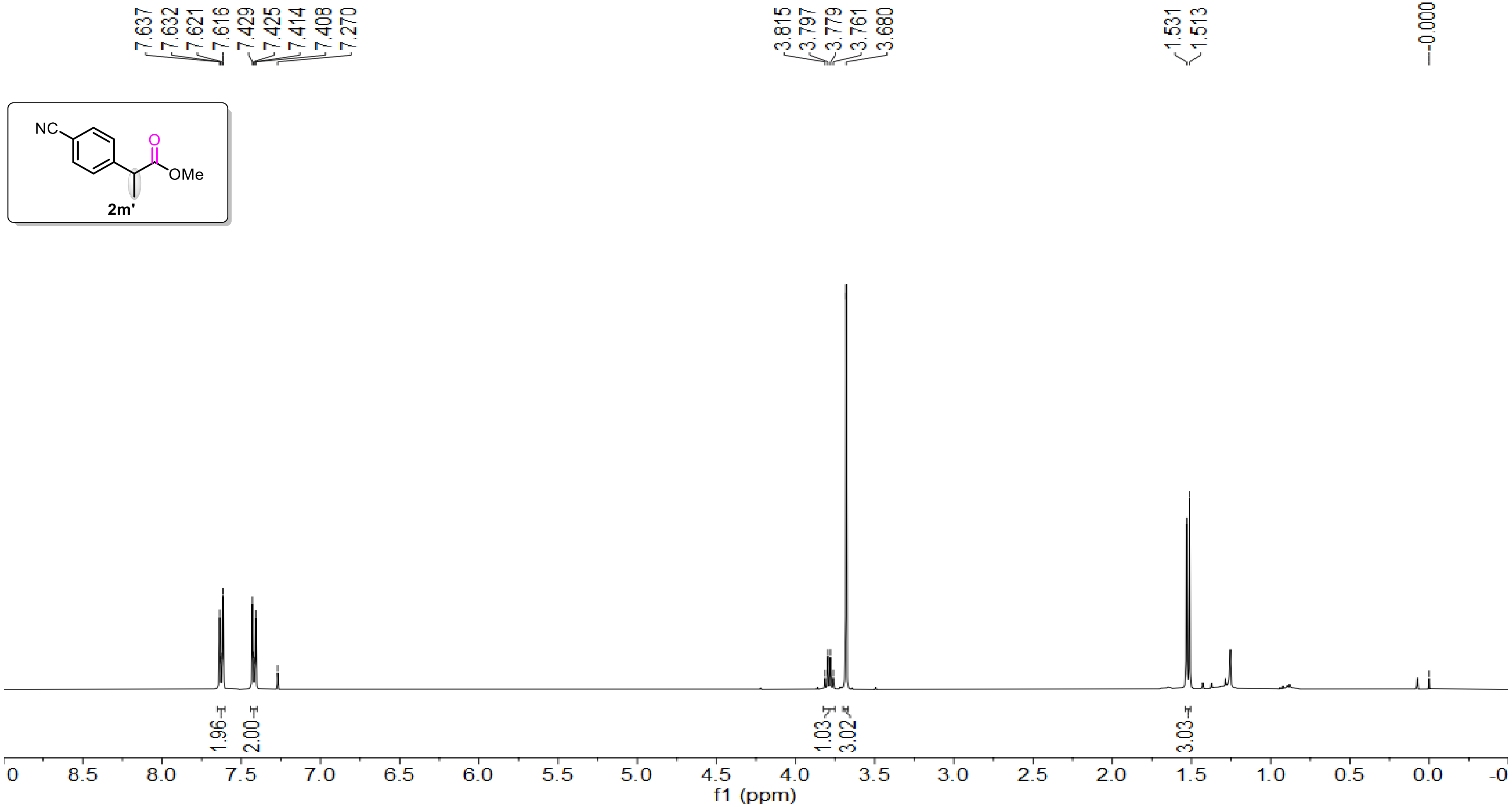

Supplementary Fig. 41. ^{13}C NMR Spectra (101 MHz, $CDCl_3$) of **2m'**

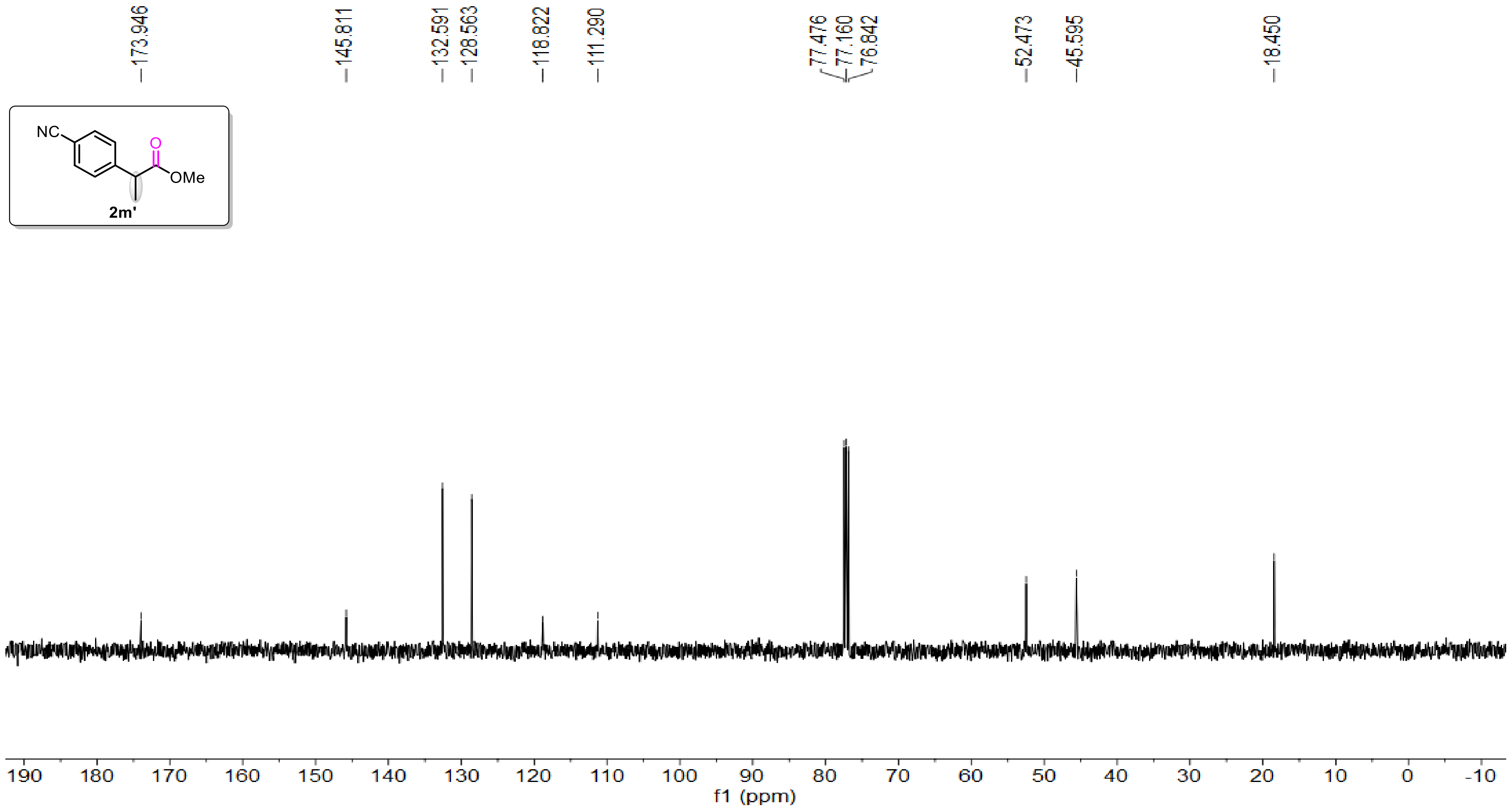

Supplementary Fig. 42. ^{1}H NMR Spectra (400 MHz, $CDCl_3$) of **2n'**

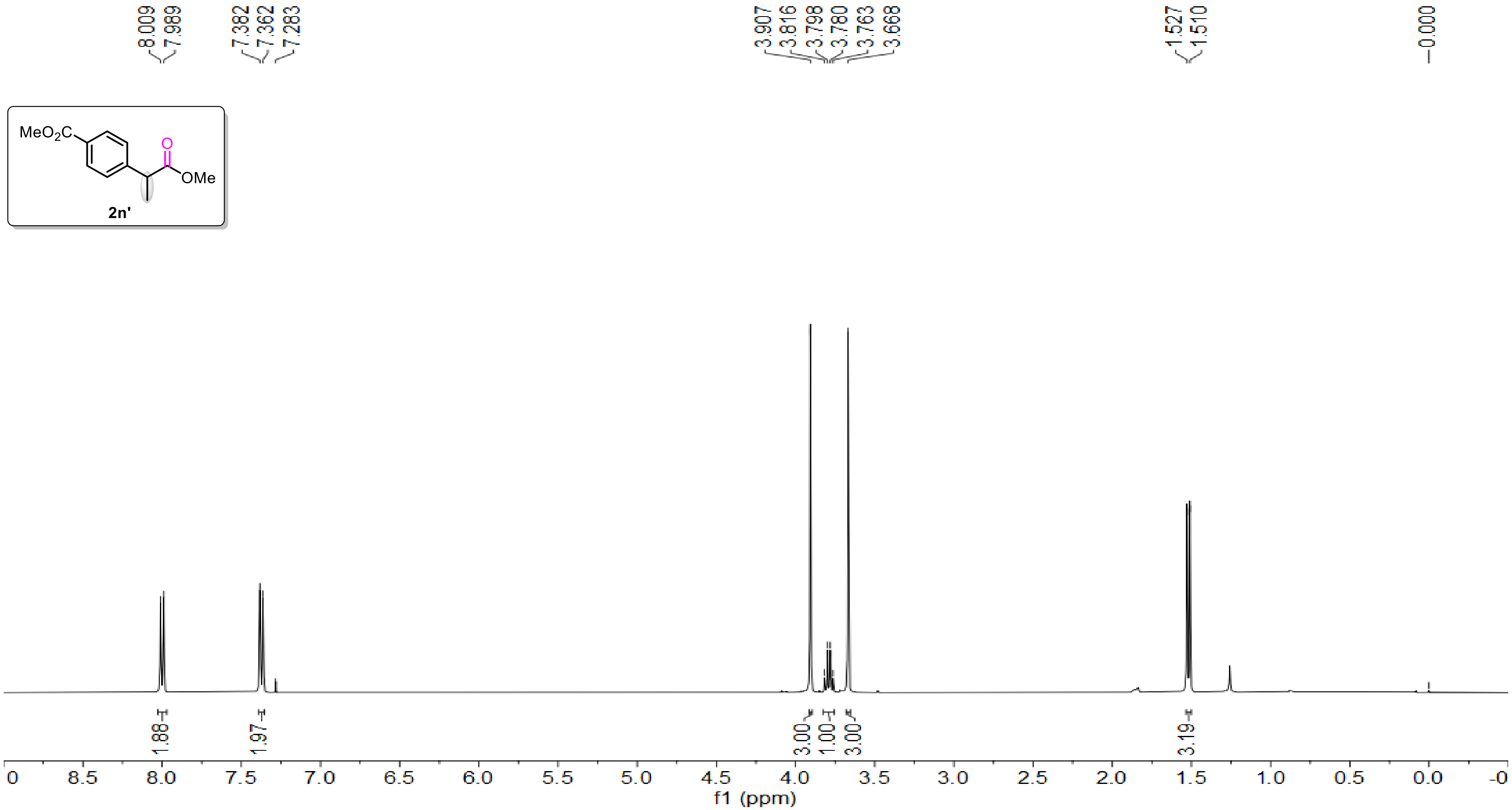

Supplementary Fig. 43. ^{13}C NMR Spectra (101 MHz, $CDCl_3$) of **2n'**

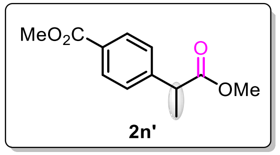

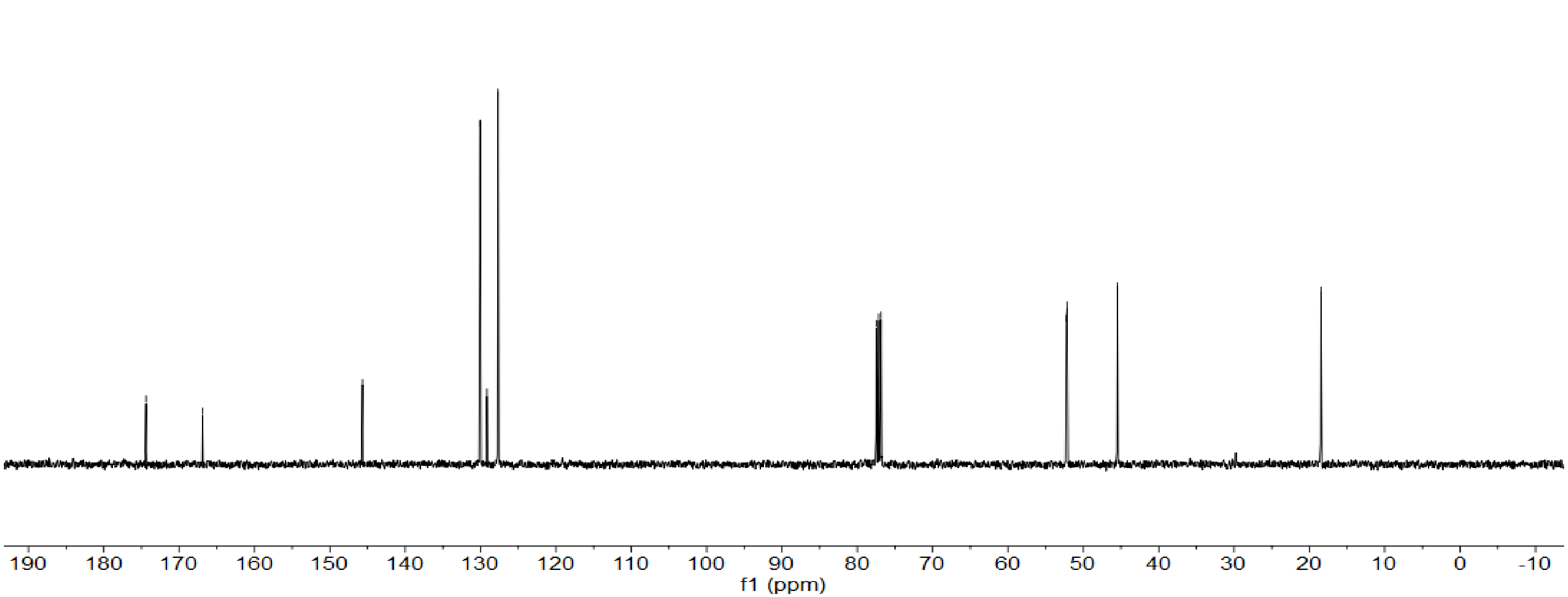

Supplementary Fig. 44. ^{1}H NMR Spectra (400 MHz, $CDCl_3$) of **2o'**

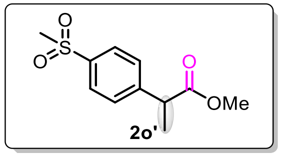

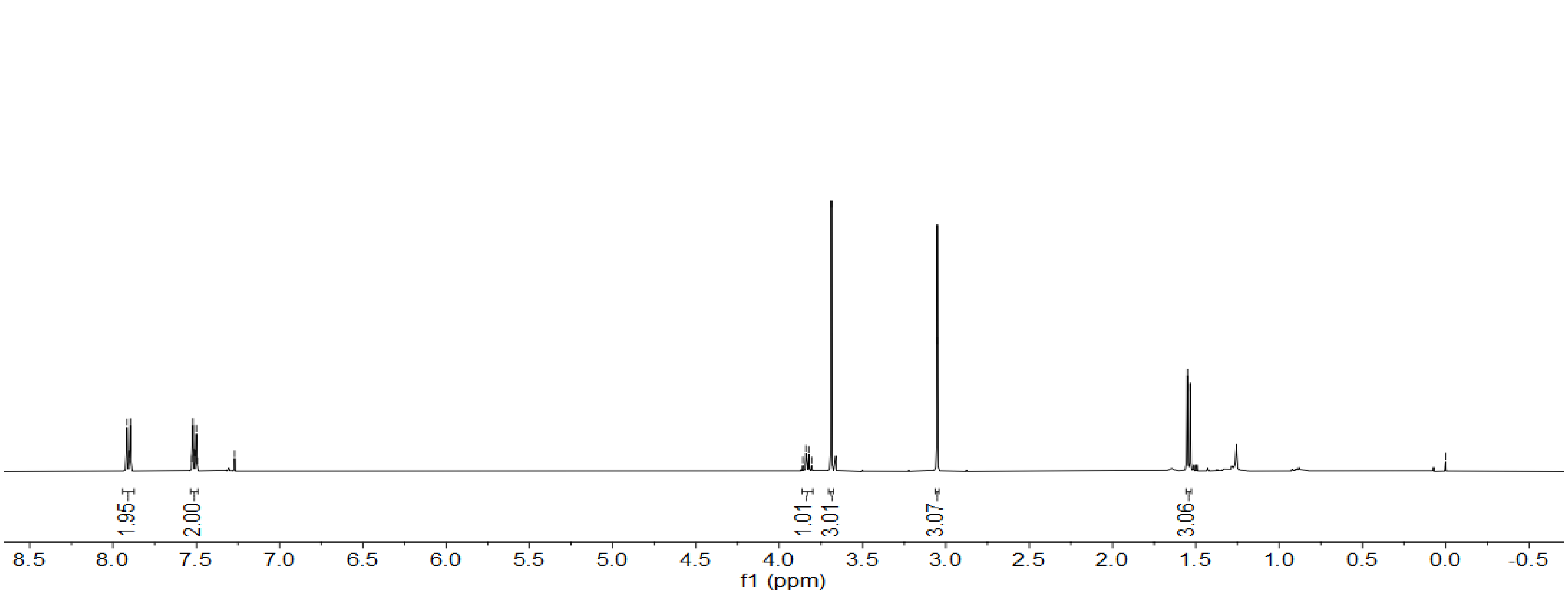

Supplementary Fig. 45. ^{13}C NMR Spectra (101 MHz, $CDCl_3$) of **2o'**

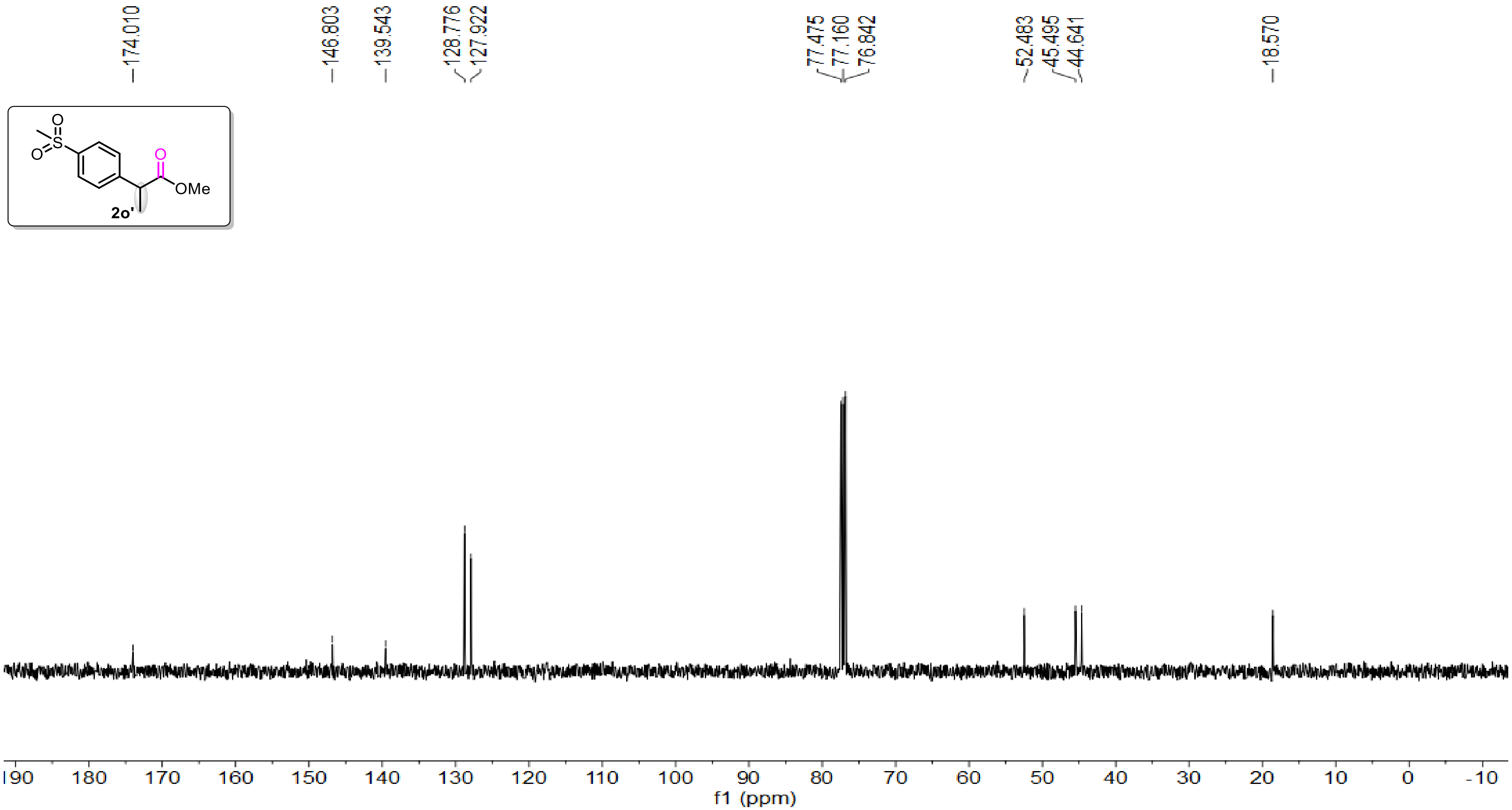

Supplementary Fig. 46. ^{1}H NMR Spectra (400 MHz, $CDCl_3$) of **2p'**

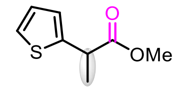

2p'

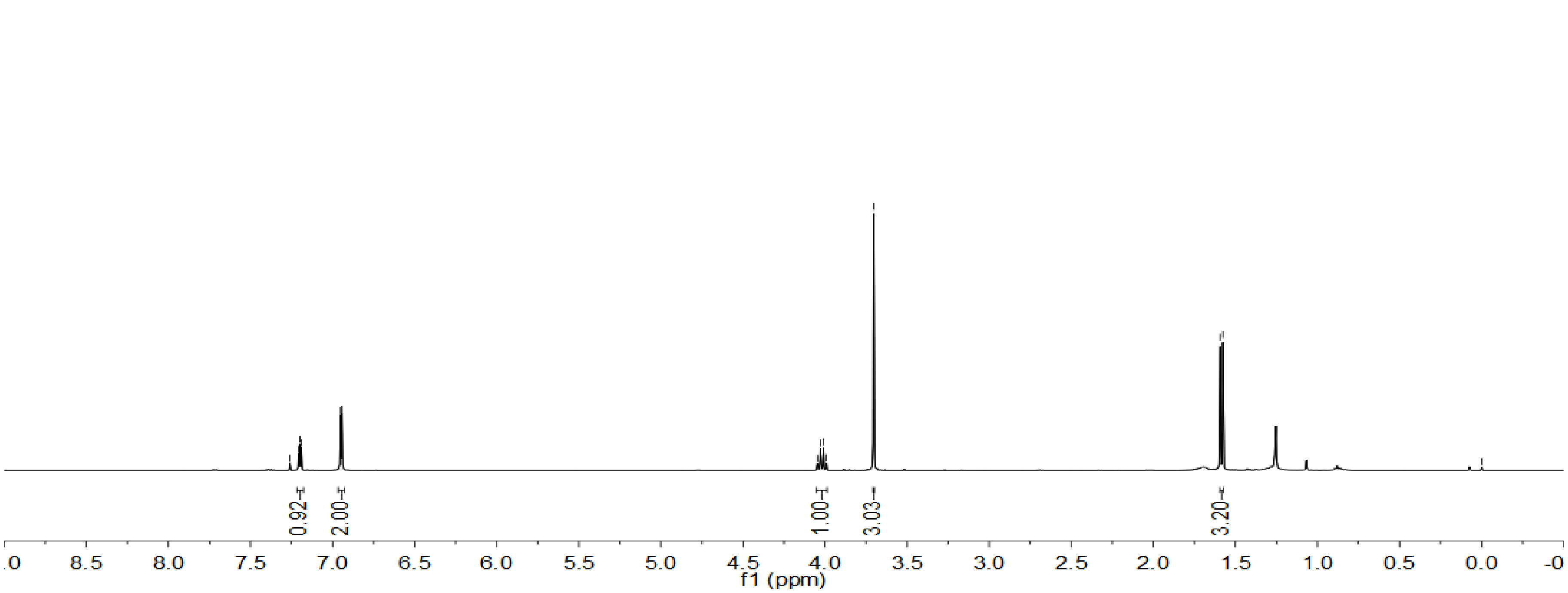

Supplementary Fig. 47. ^{13}C NMR Spectra (101 MHz, $CDCl_3$) of **2p'**

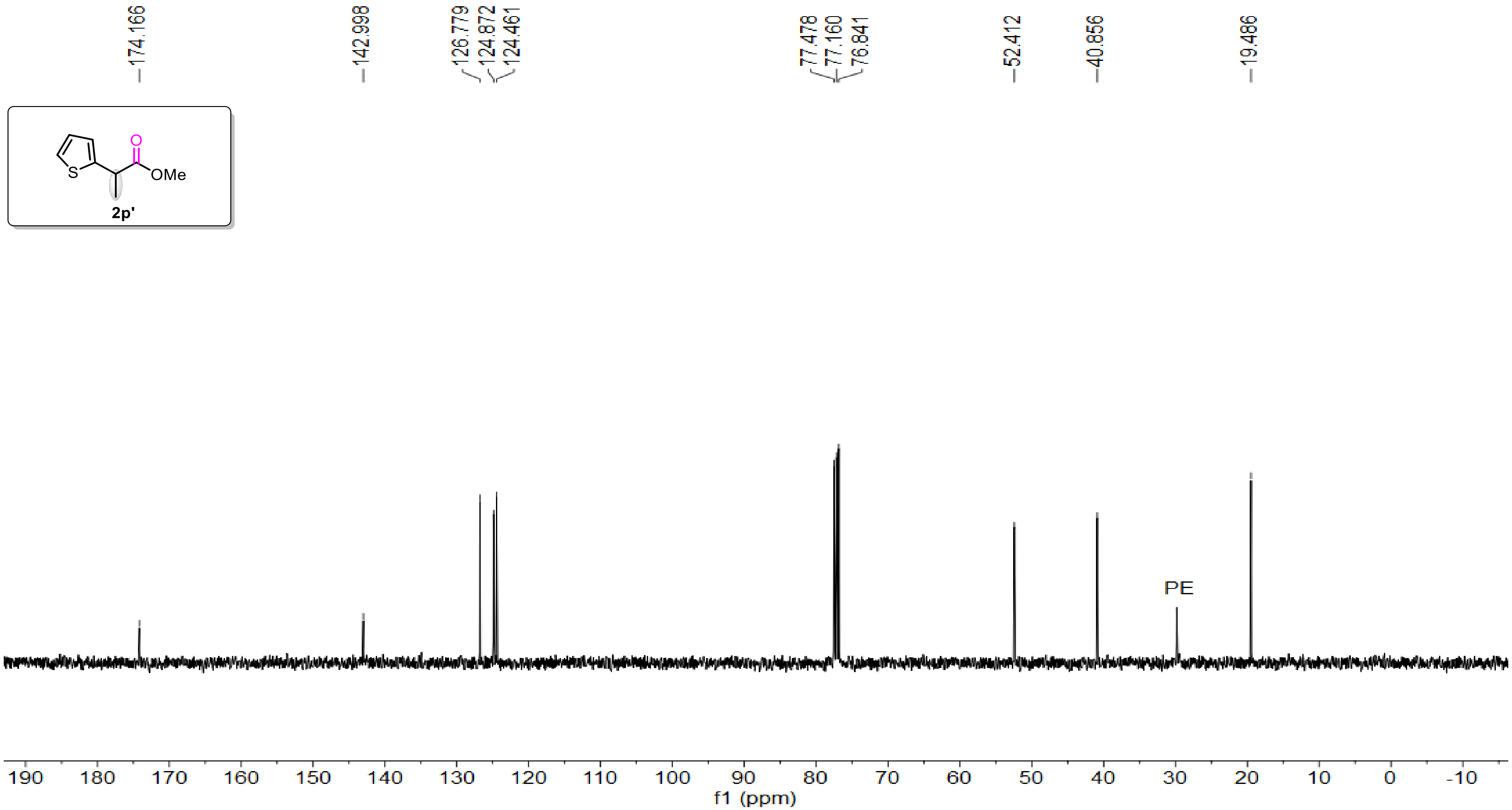

Supplementary Fig. 48. ^{1}H NMR Spectra (400 MHz, $CDCl_3$) of **2q'**

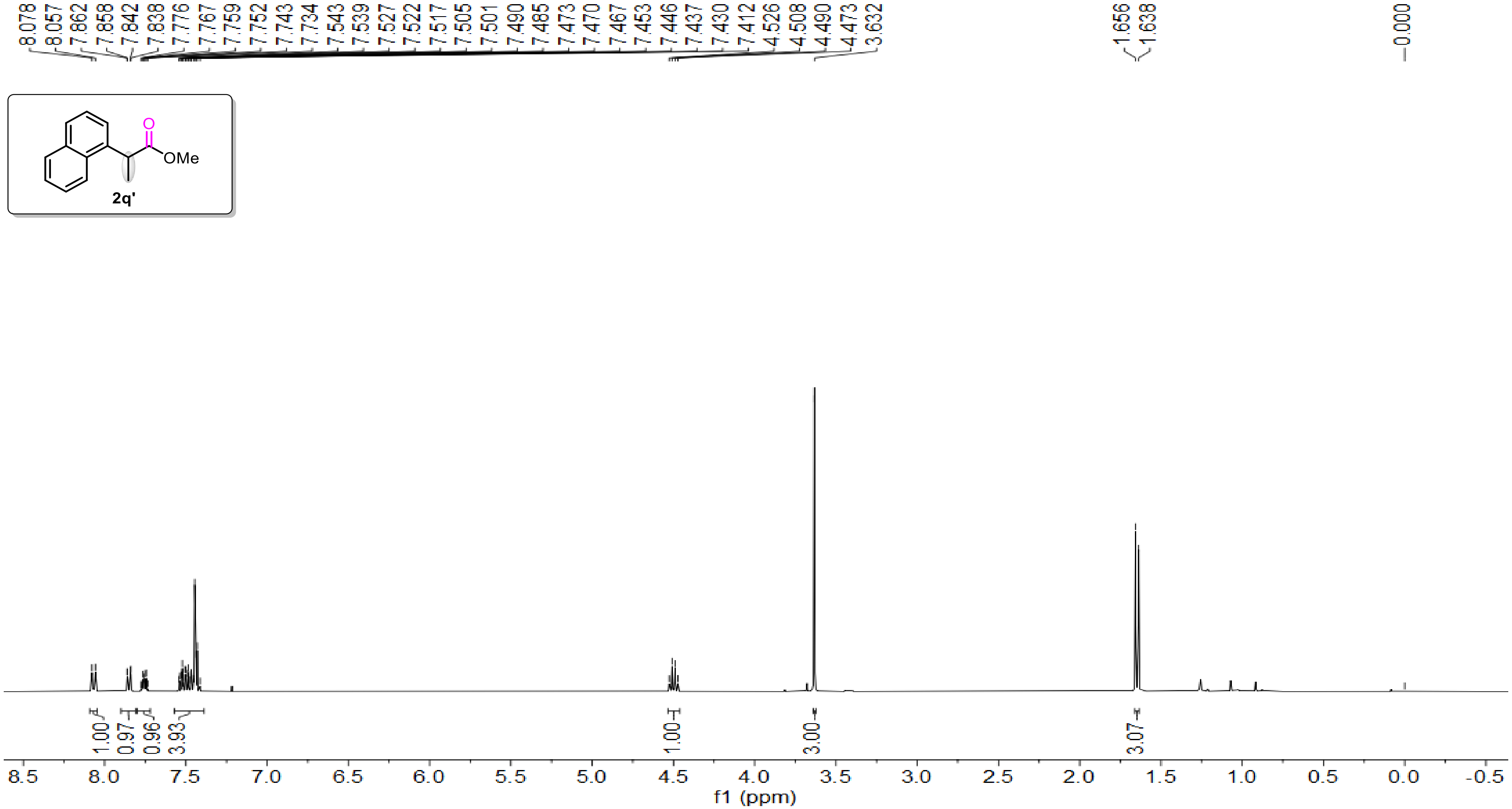

Supplementary Fig. 49. ^{13}C NMR Spectra (101 MHz, $CDCl_3$) of **2q'**

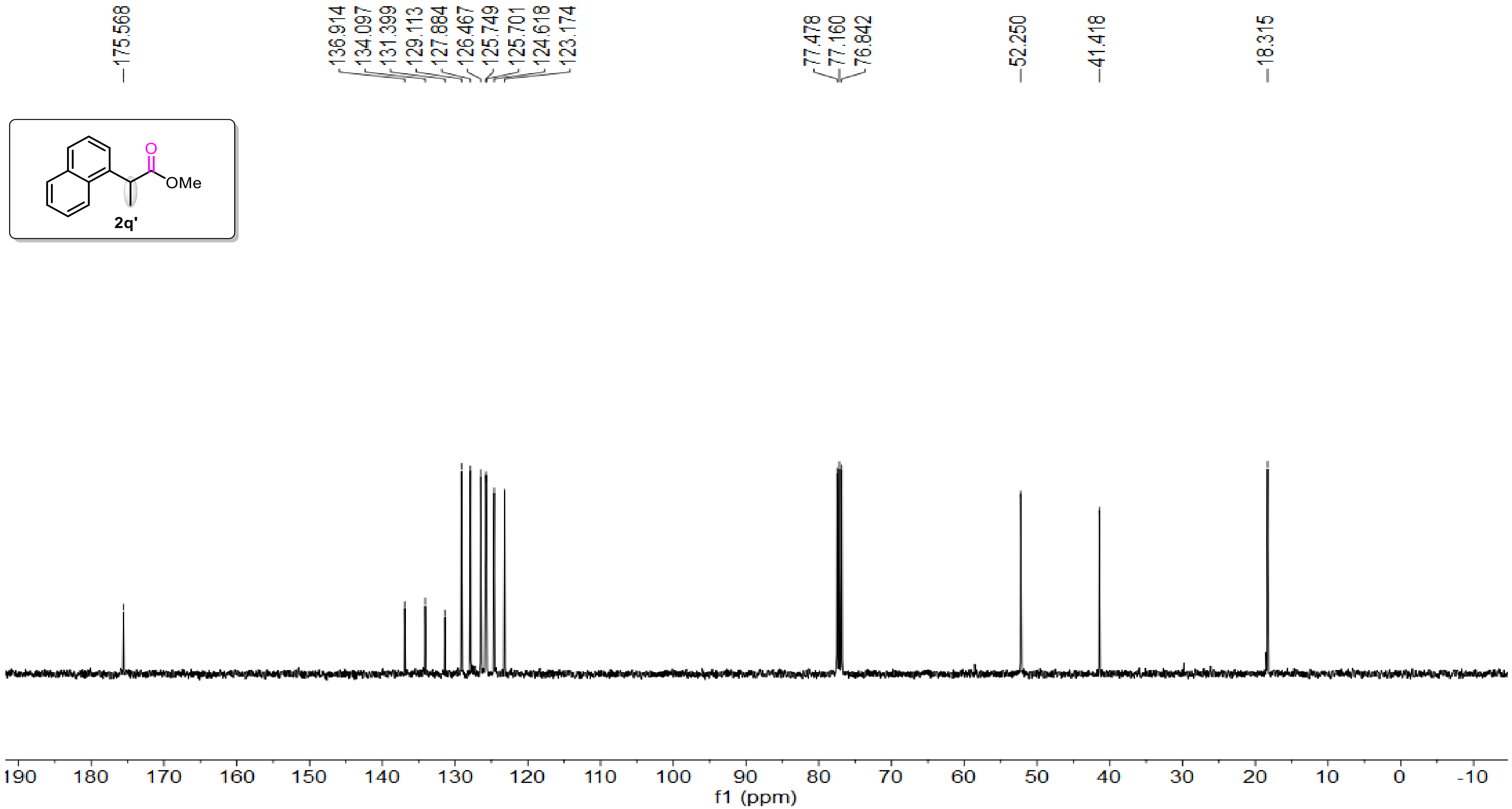

Supplementary Fig. 50. ^{1}H NMR Spectra (400 MHz, $CDCl_3$) of **2r'**

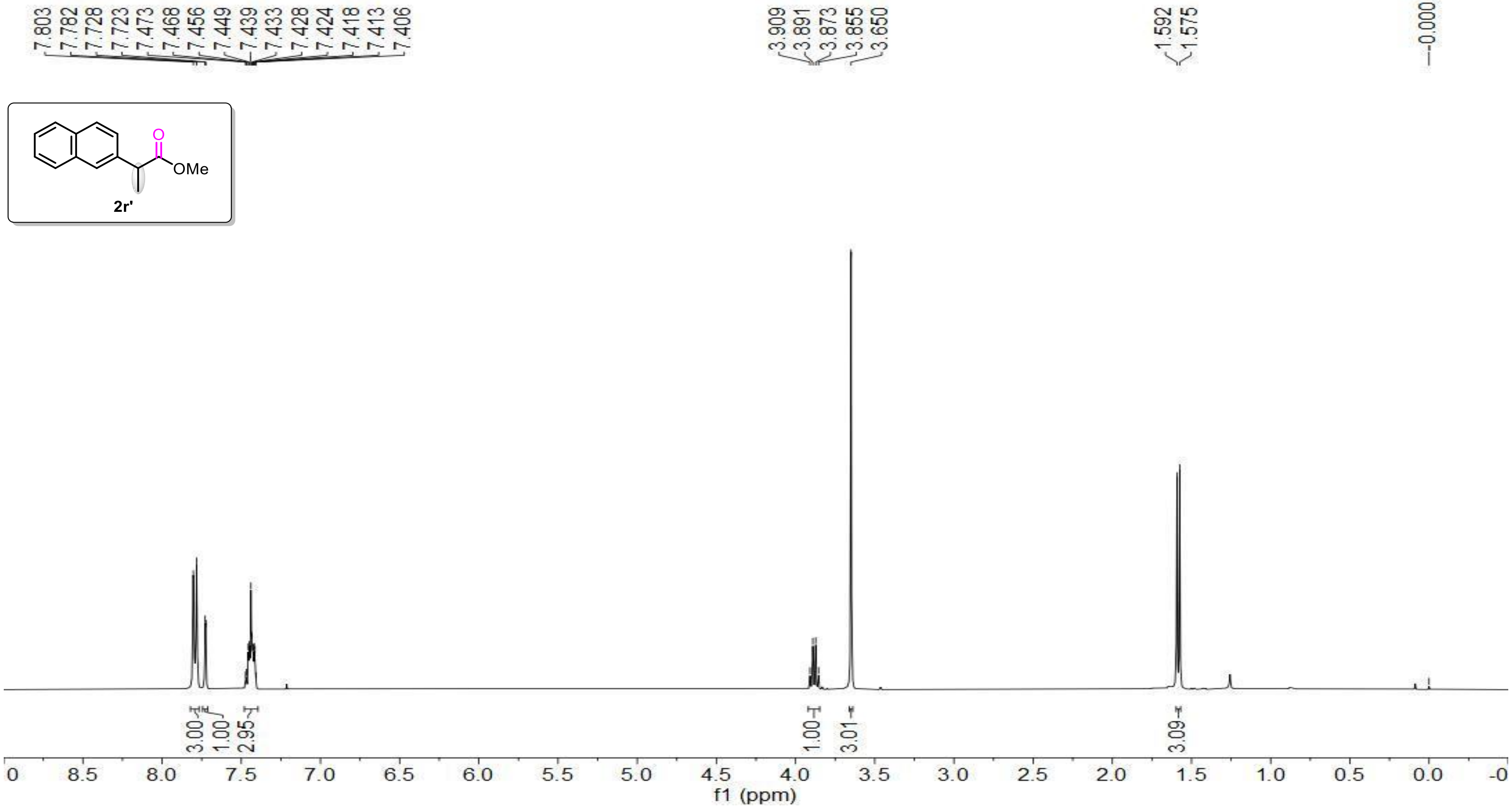

Supplementary Fig. 51. ^{13}C NMR Spectra (101 MHz, $CDCl_3$) of **2r'**

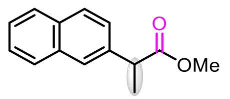

2r'

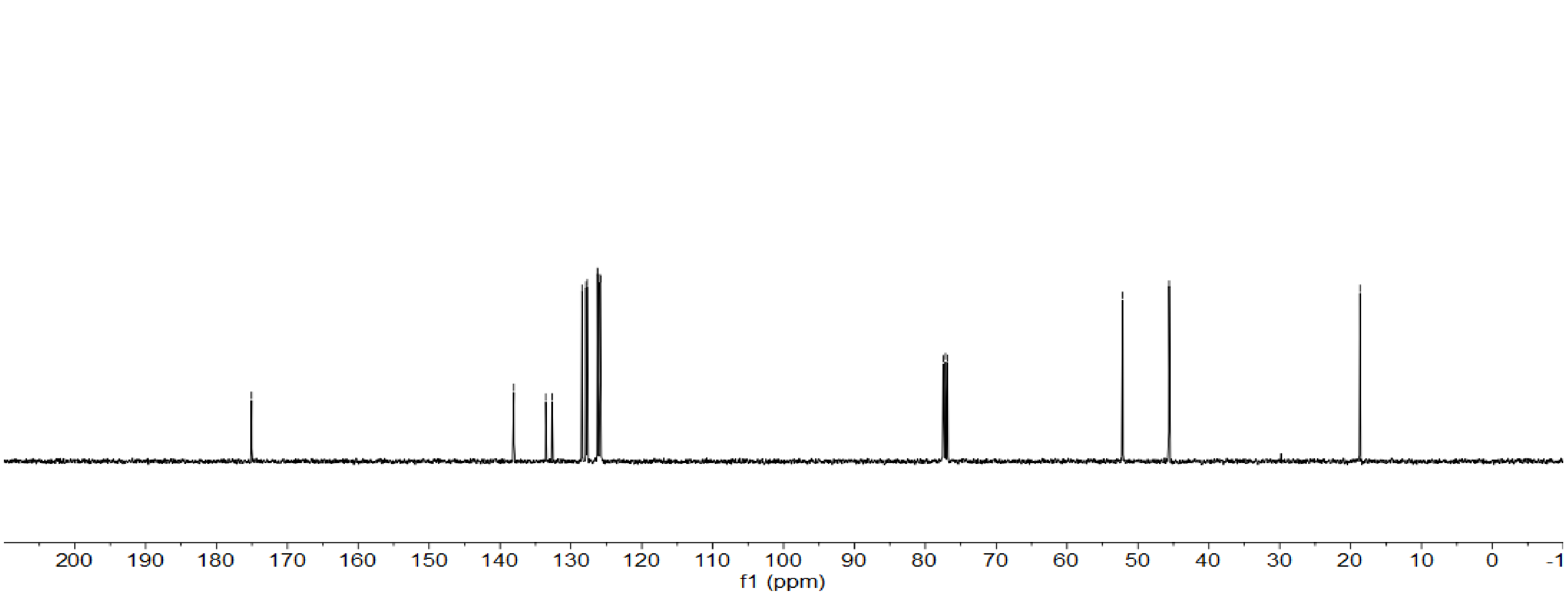

Supplementary Fig. 52. ^{1}H NMR Spectra (400 MHz, $CDCl_3$) of **crude 3-phenylpropanoyl chloride 3a**

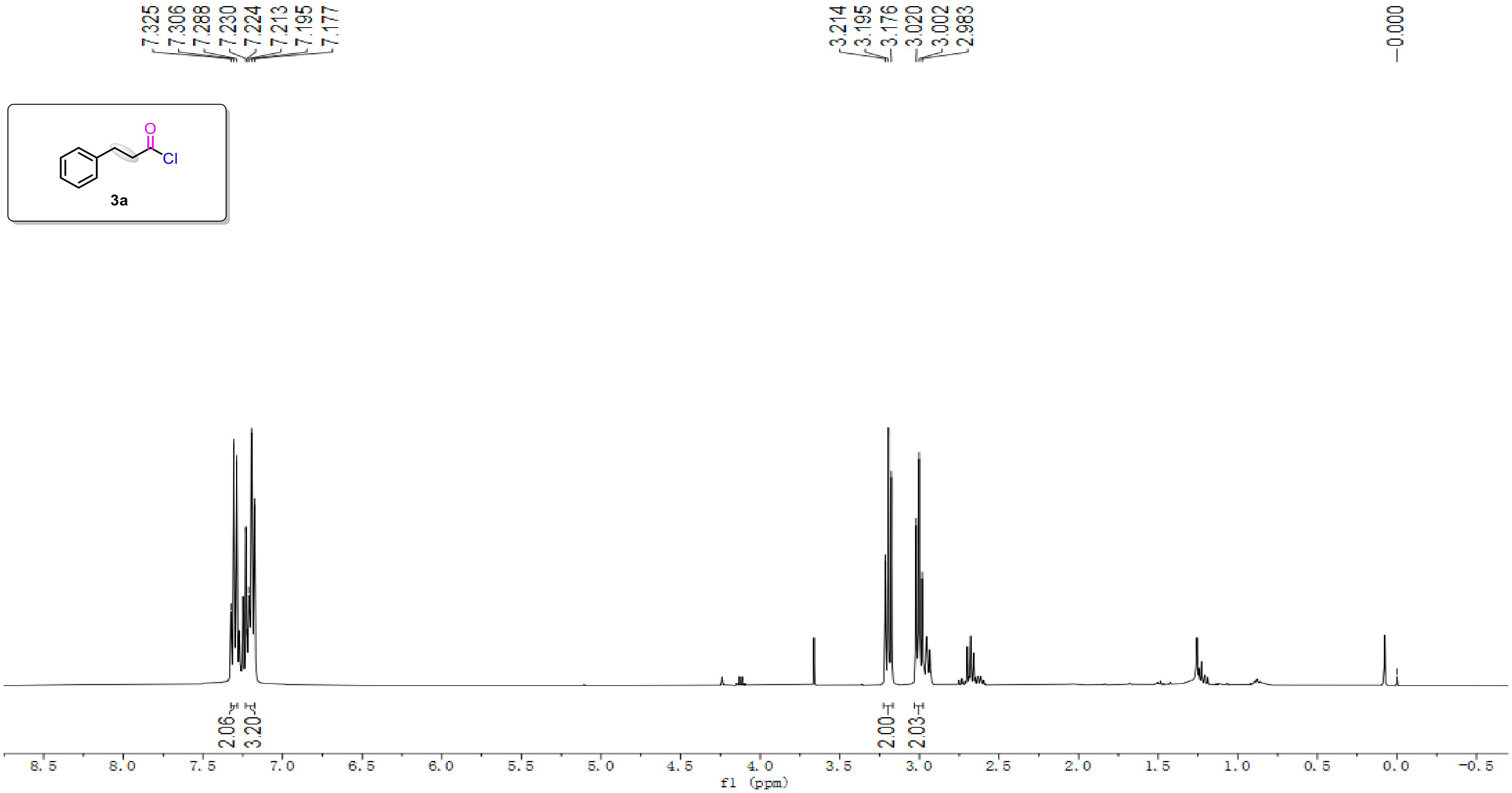

Supplementary Fig. 53. ^{13}C NMR Spectra (101 MHz, $CDCl_3$) of **crude 3-phenylpropanoyl chloride 3a**

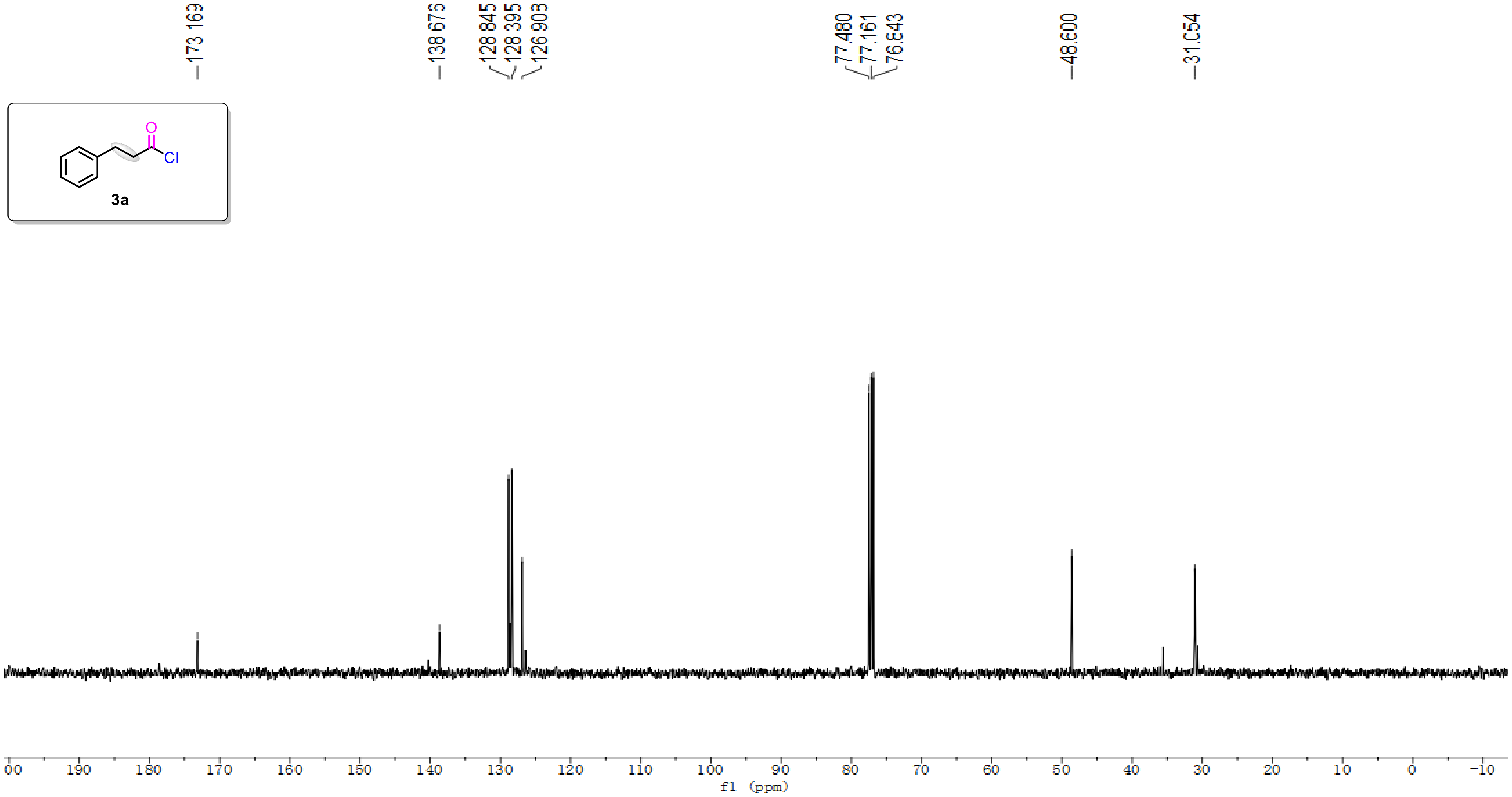

Supplementary Fig. 54. ^{1}H NMR Spectra (400 MHz, $CDCl_3$) of **3a'**

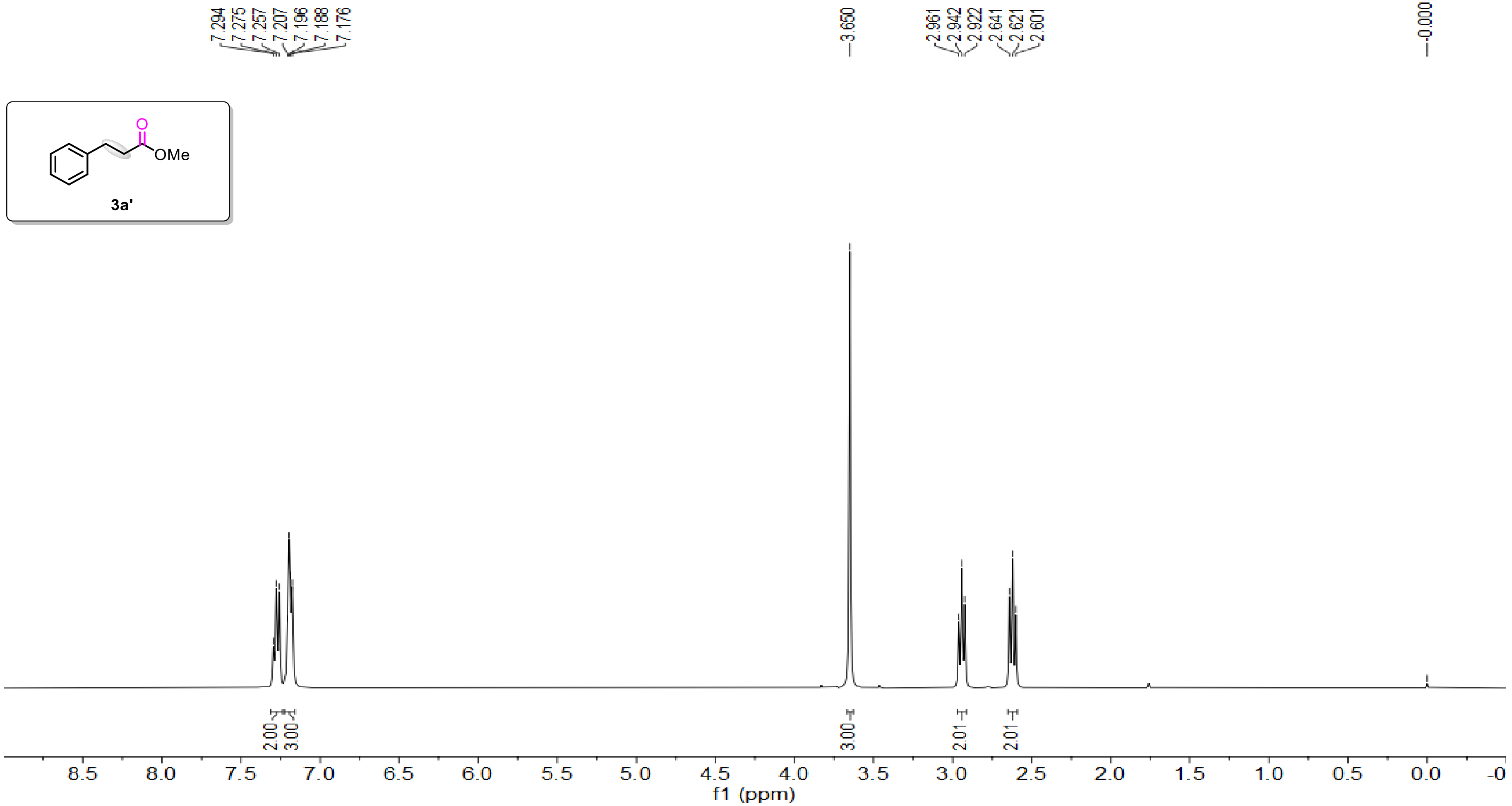

Supplementary Fig. 55. ^{13}C NMR Spectra (101 MHz, $CDCl_3$) of **3a'**

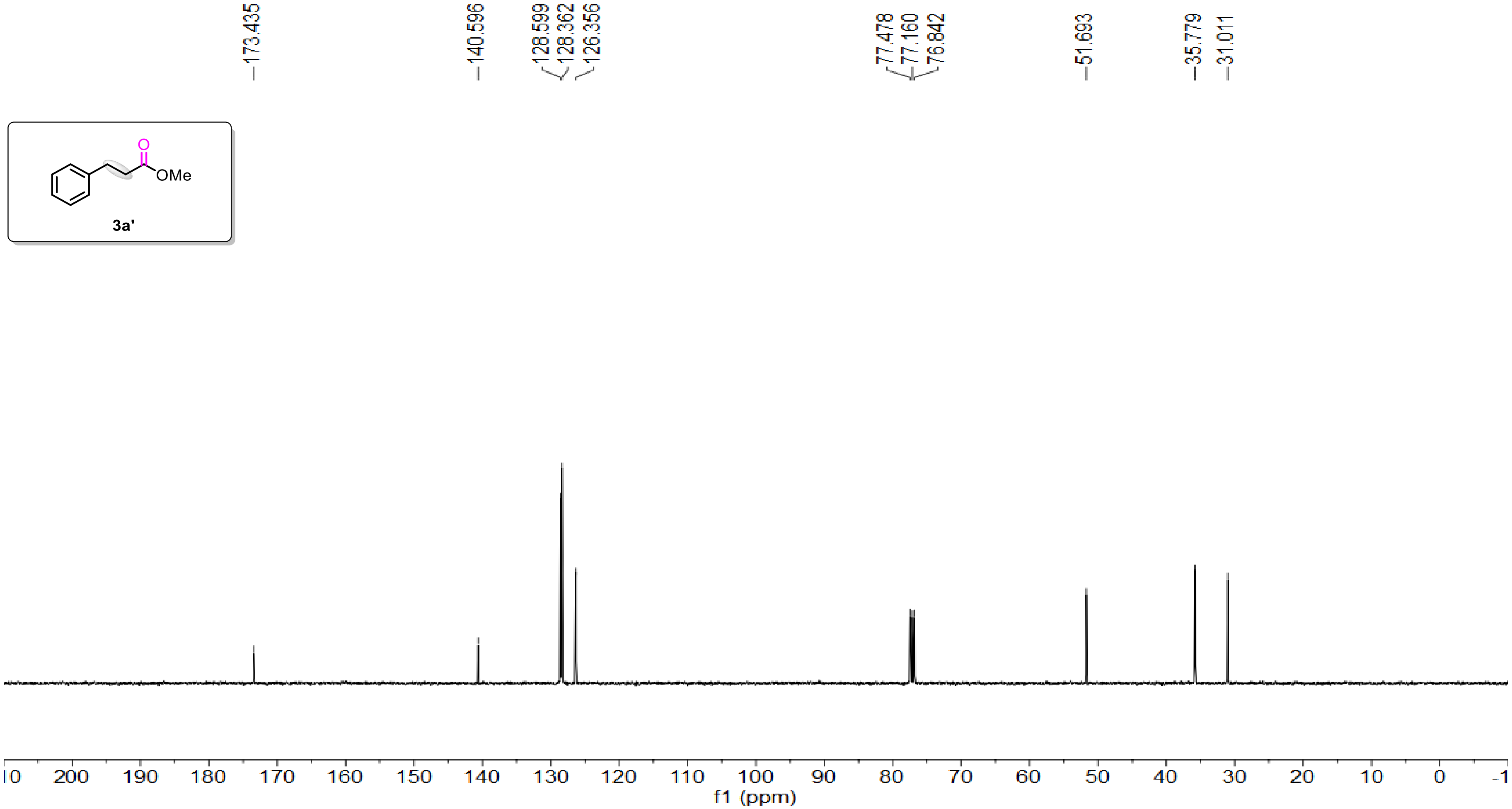

Supplementary Fig. 56. ^{1}H NMR Spectra (400 MHz, $CDCl_3$) of **3b'**

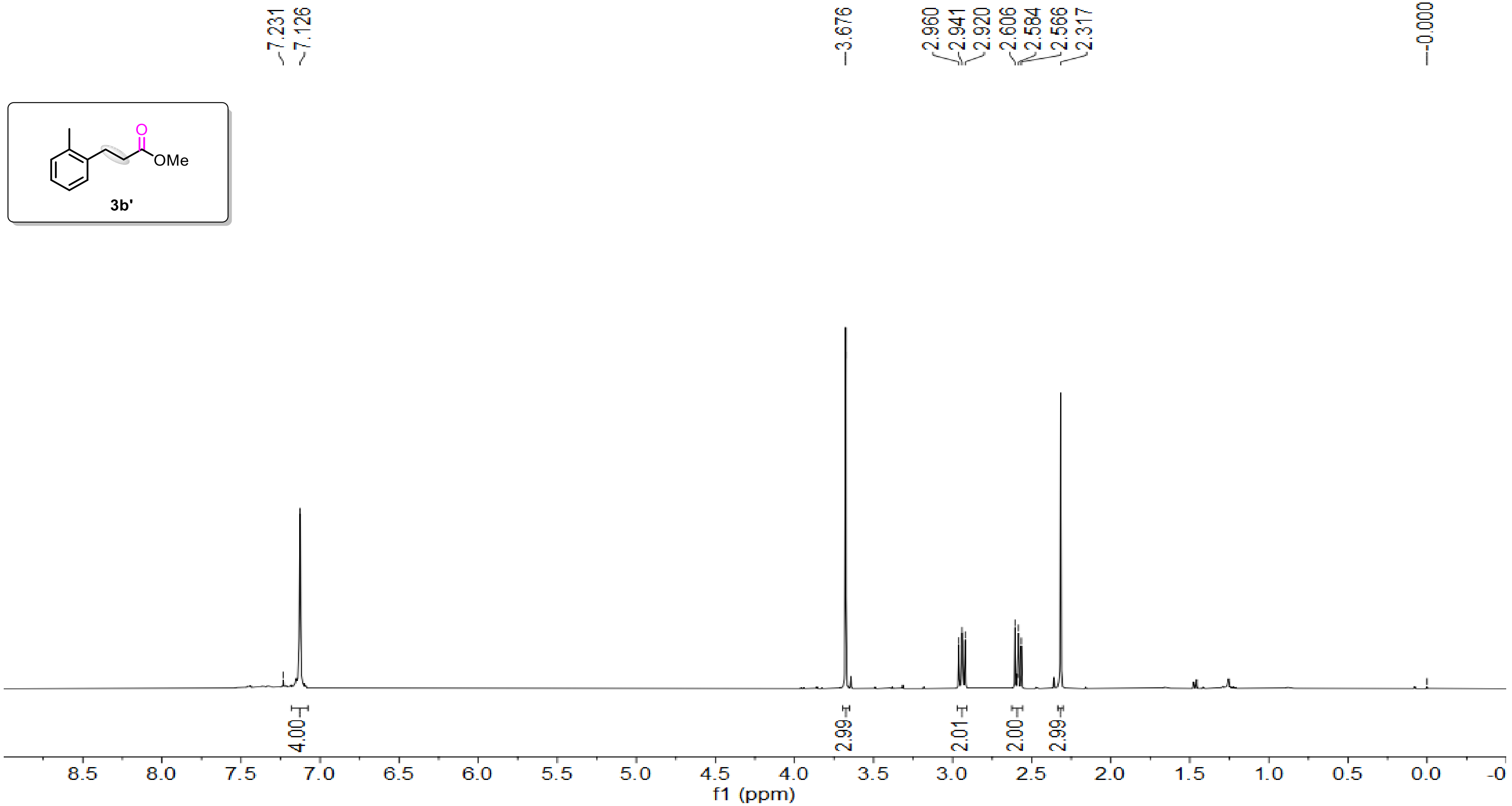

Supplementary Fig. 57. ^{13}C NMR Spectra (101 MHz, $CDCl_3$) of **3b'**

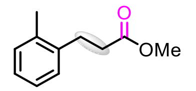

3b'

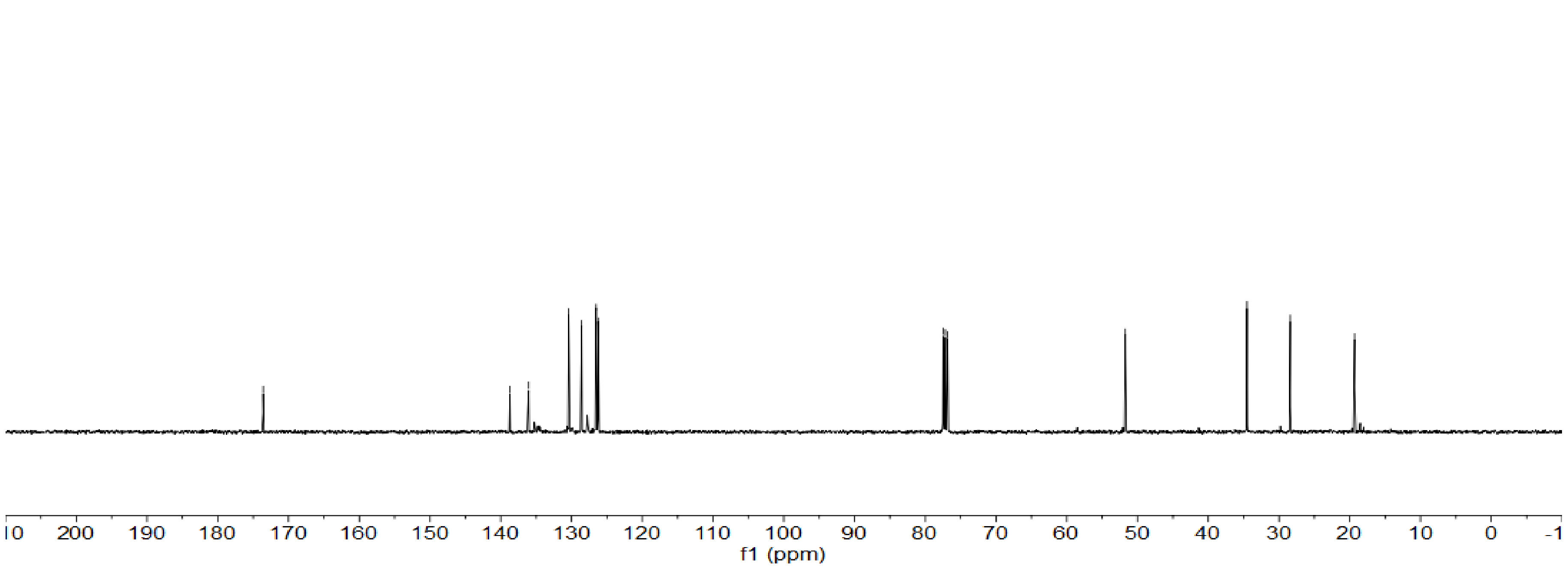

Supplementary Fig. 58. ^{1}H NMR Spectra (400 MHz, $CDCl_3$) of **3c'**

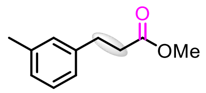

3c'

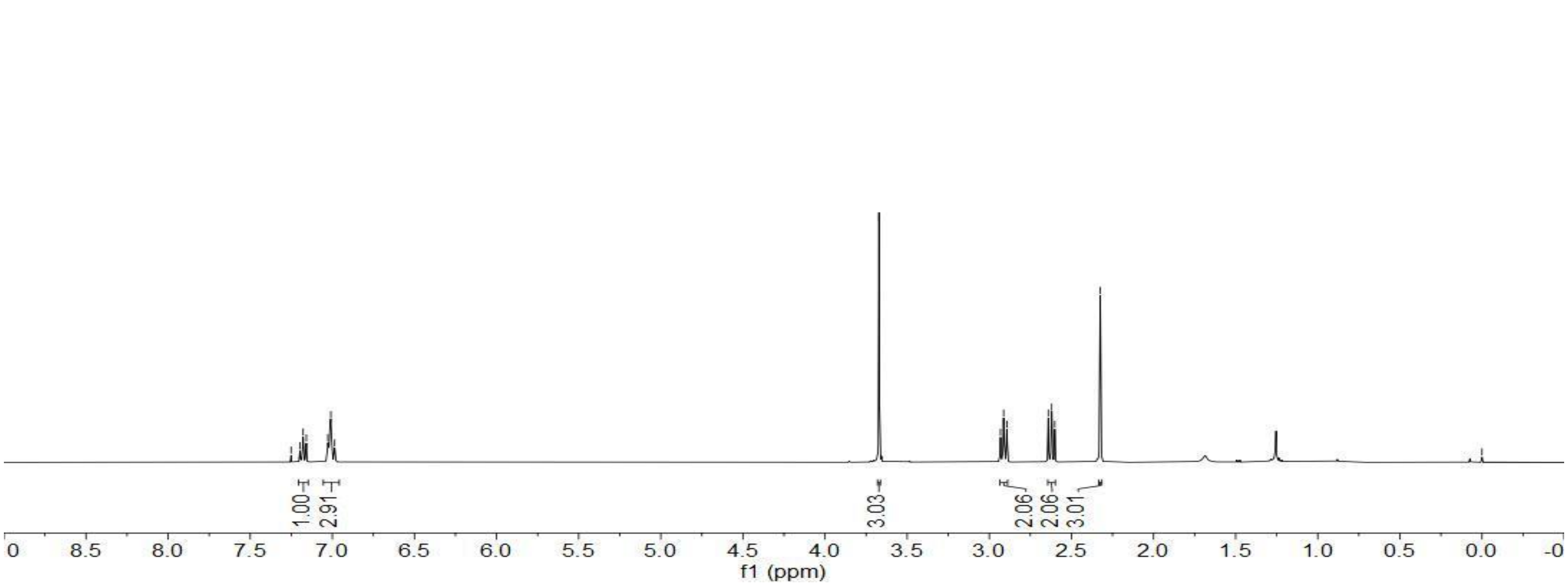

Supplementary Fig. 59. ^{13}C NMR Spectra (101 MHz, $CDCl_3$) of **3c'**

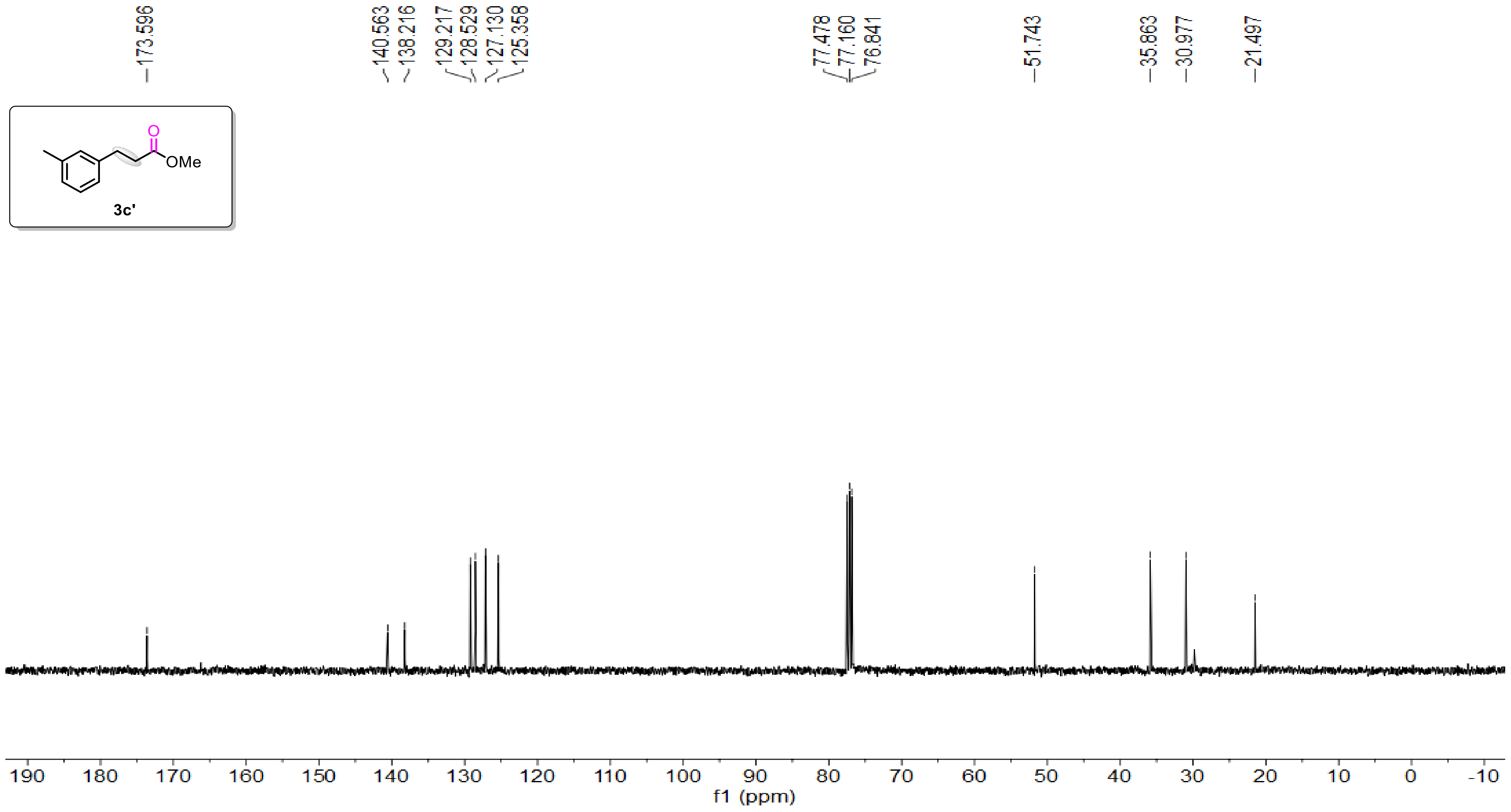

Supplementary Fig. 60. ^{1}H NMR Spectra (400 MHz, $CDCl_3$) of **3d'**

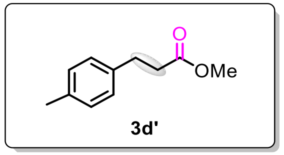

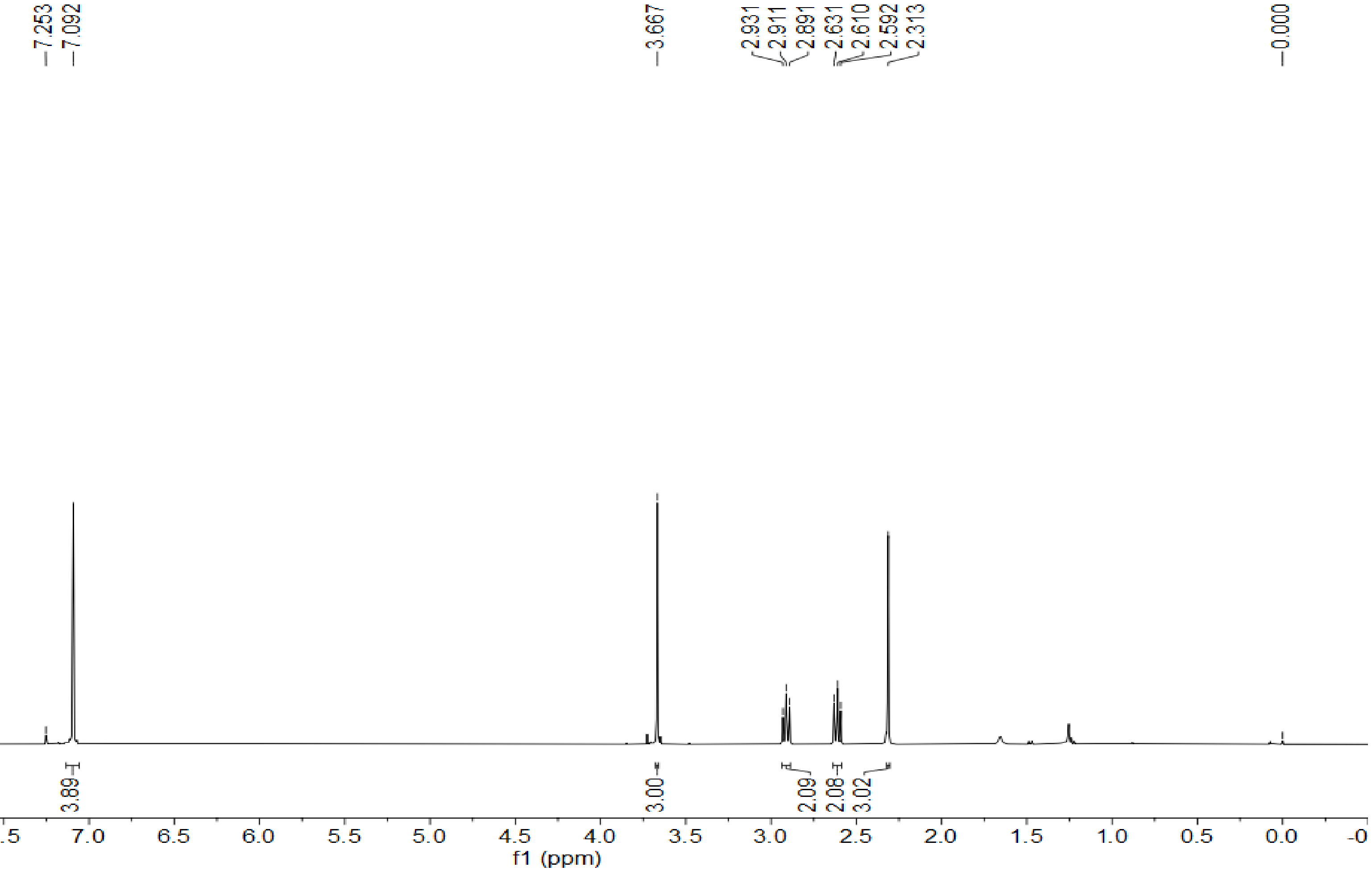

Supplementary Fig. 61. ^{13}C NMR Spectra (101 MHz, $CDCl_3$) of **3d'**

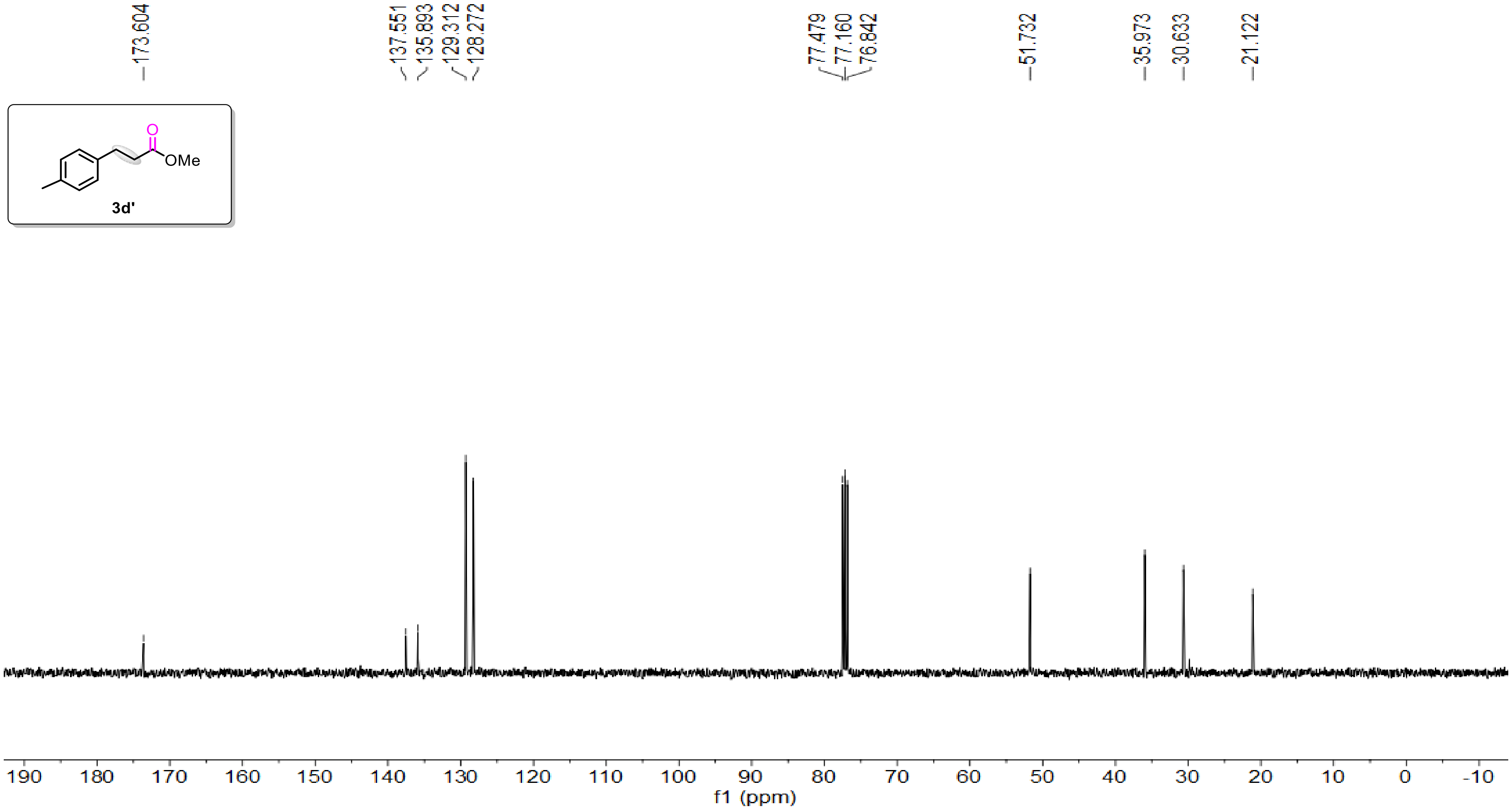

Supplementary Fig. 62. ^{1}H NMR Spectra (400 MHz, $CDCl_3$) of **3e'**

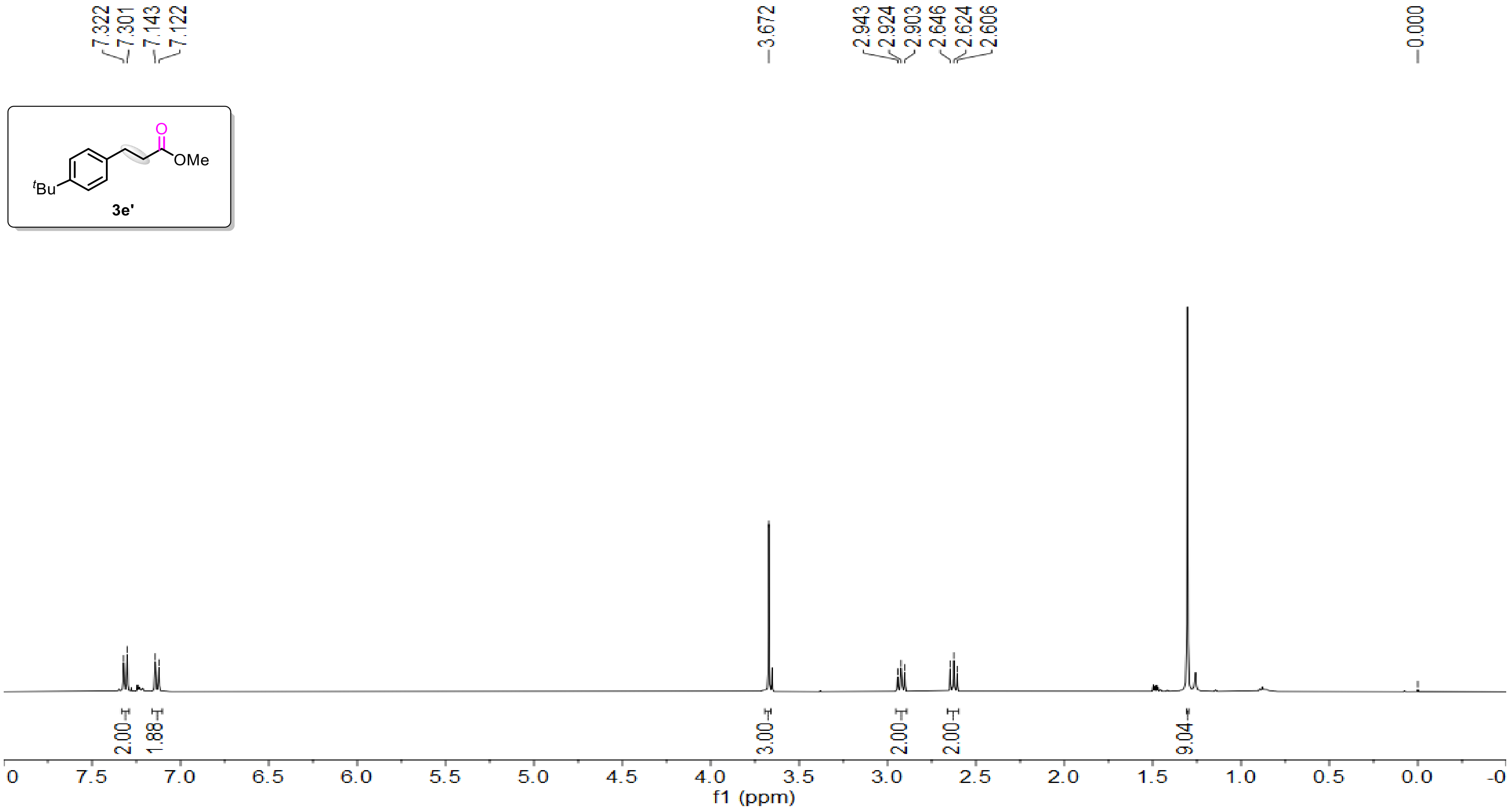

Supplementary Fig. 63. ^{13}C NMR Spectra (101 MHz, $CDCl_3$) of **3e'**

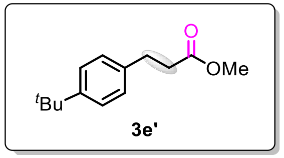

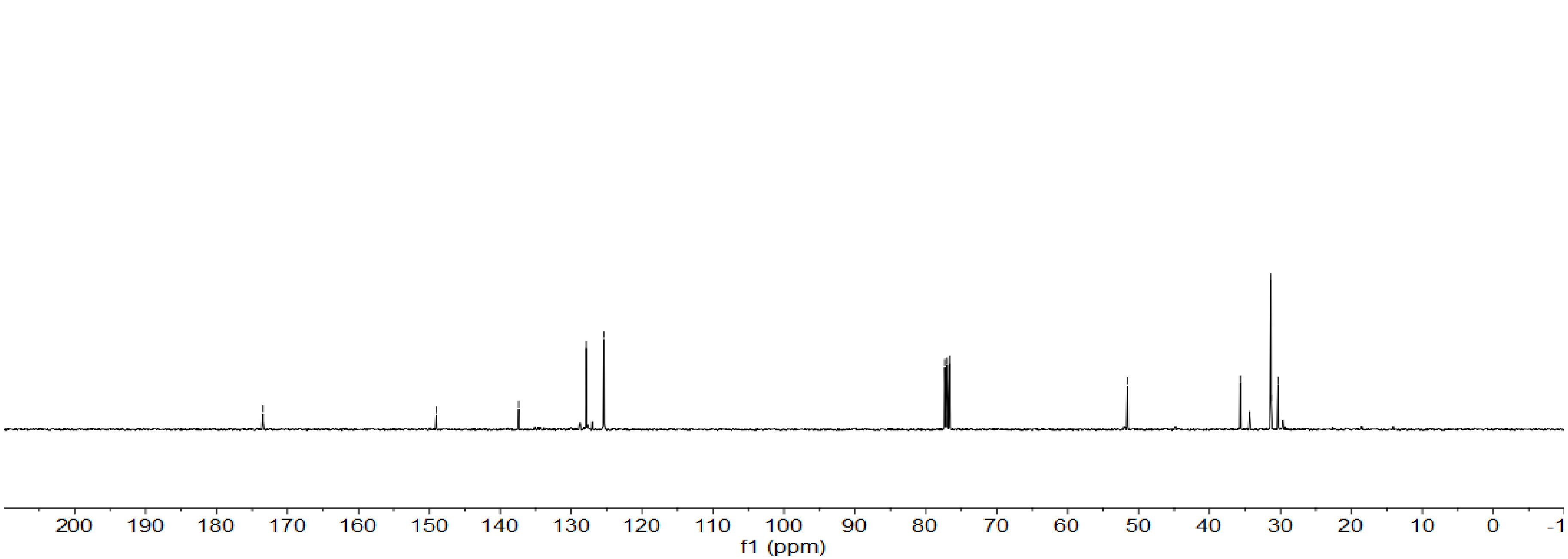

Supplementary Fig. 64. ^{1}H NMR Spectra (400 MHz, $CDCl_3$) of **3f'**

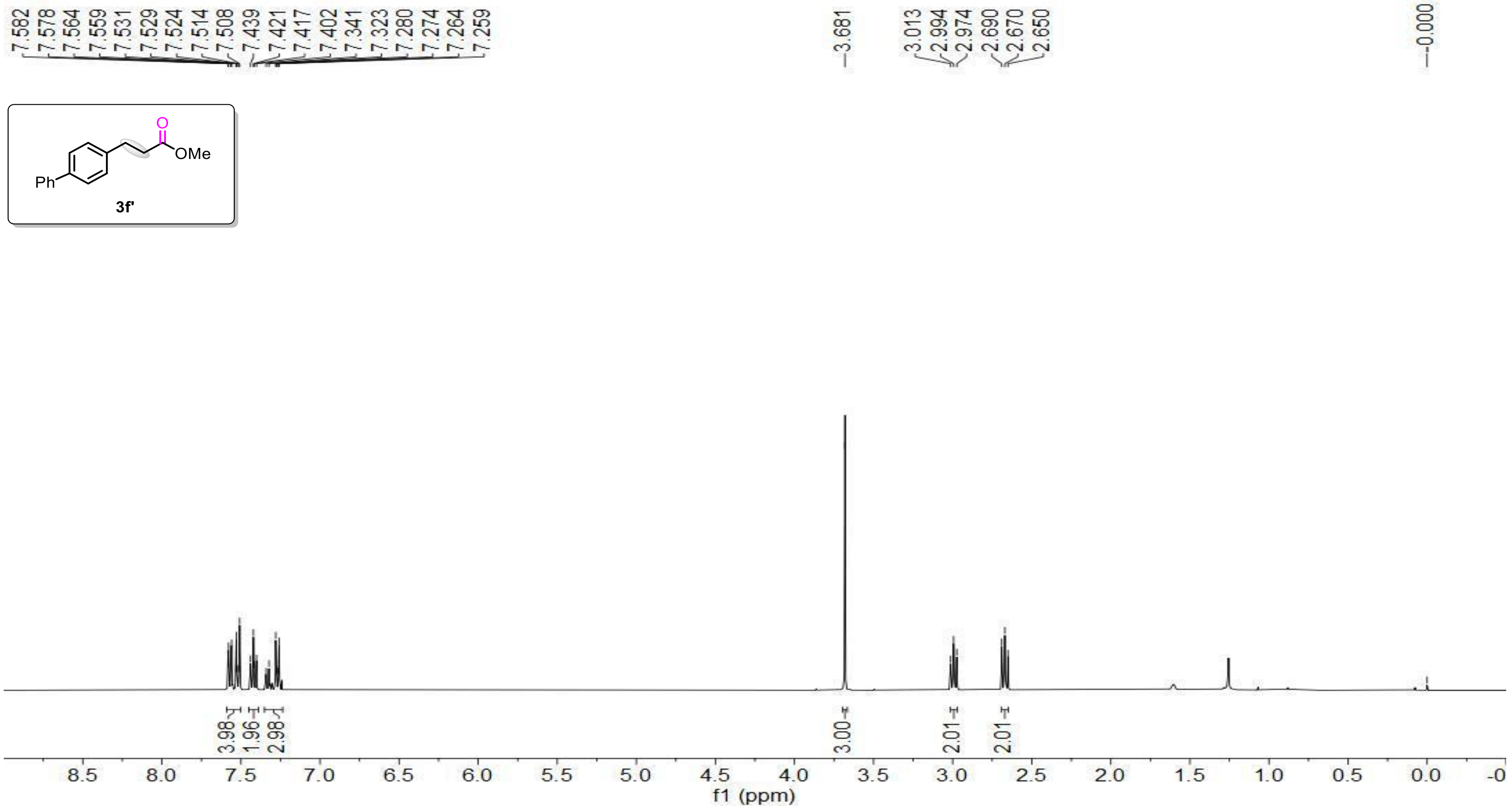

Supplementary Fig. 65. ^{13}C NMR Spectra (101 MHz, $CDCl_3$) of **3f'**

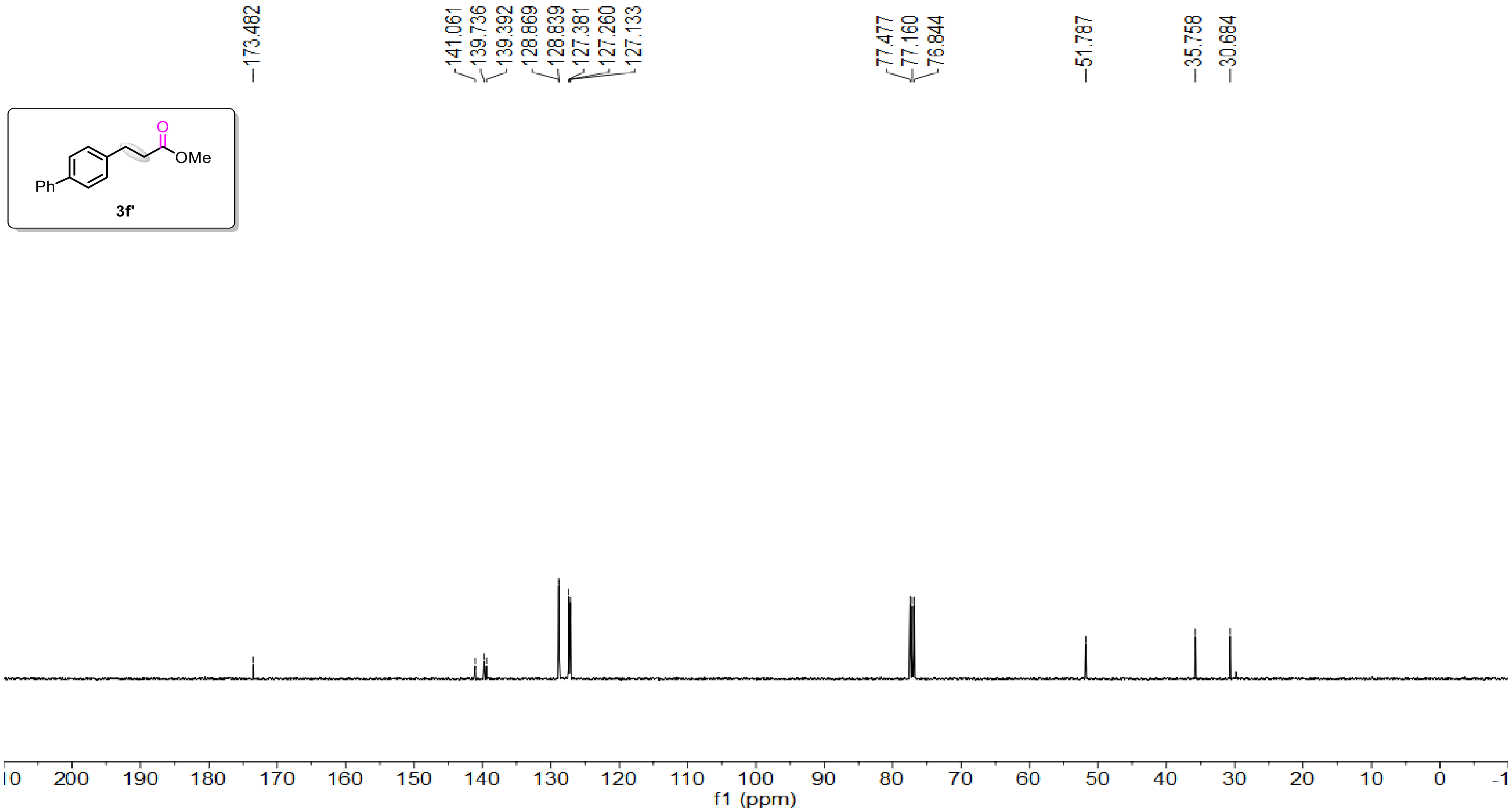

Supplementary Fig. 66. ^{1}H NMR Spectra (400 MHz, $CDCl_3$) of **3g'**

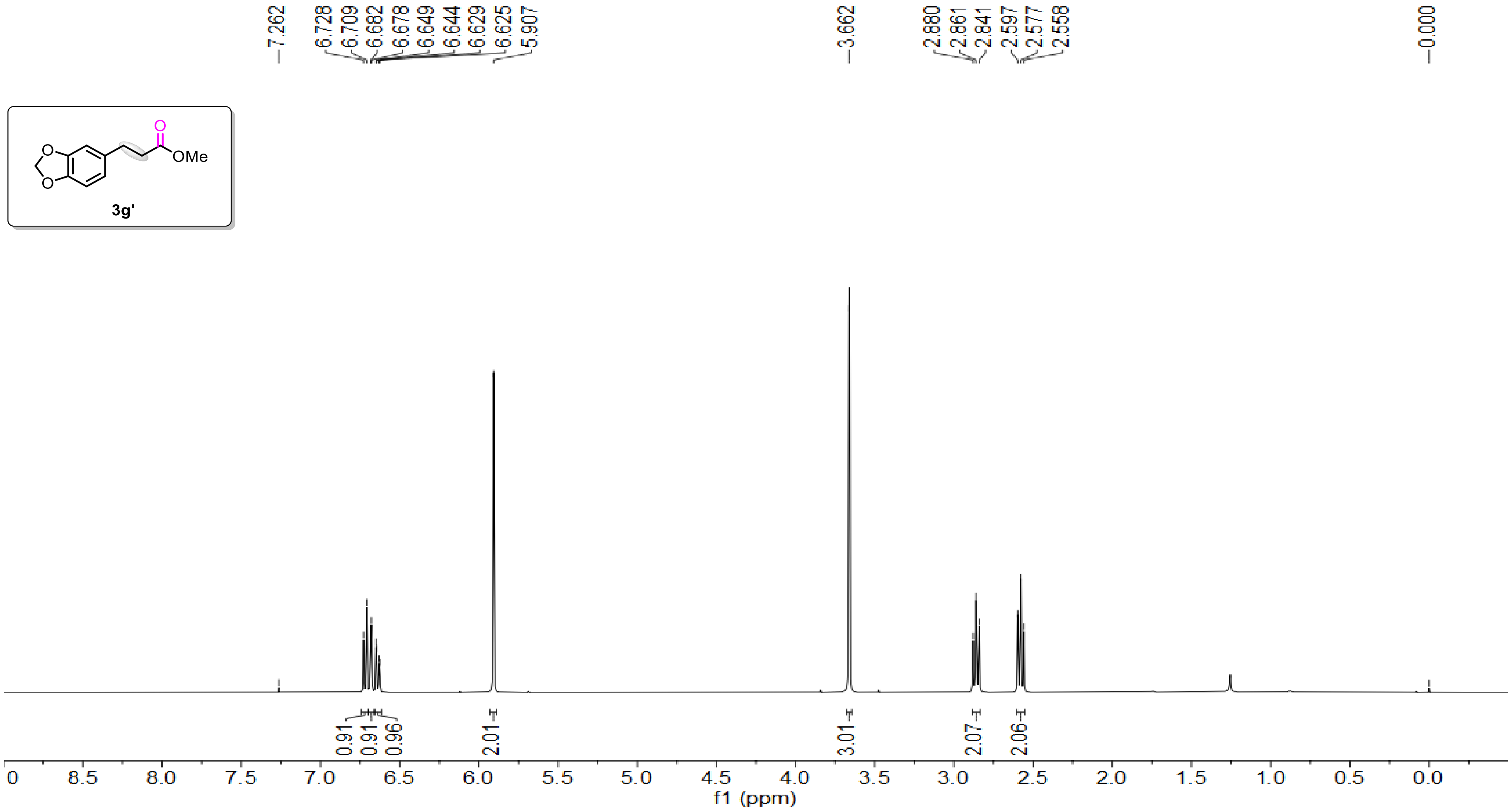

Supplementary Fig. 67. ^{13}C NMR Spectra (101 MHz, $CDCl_3$) of **3g'**

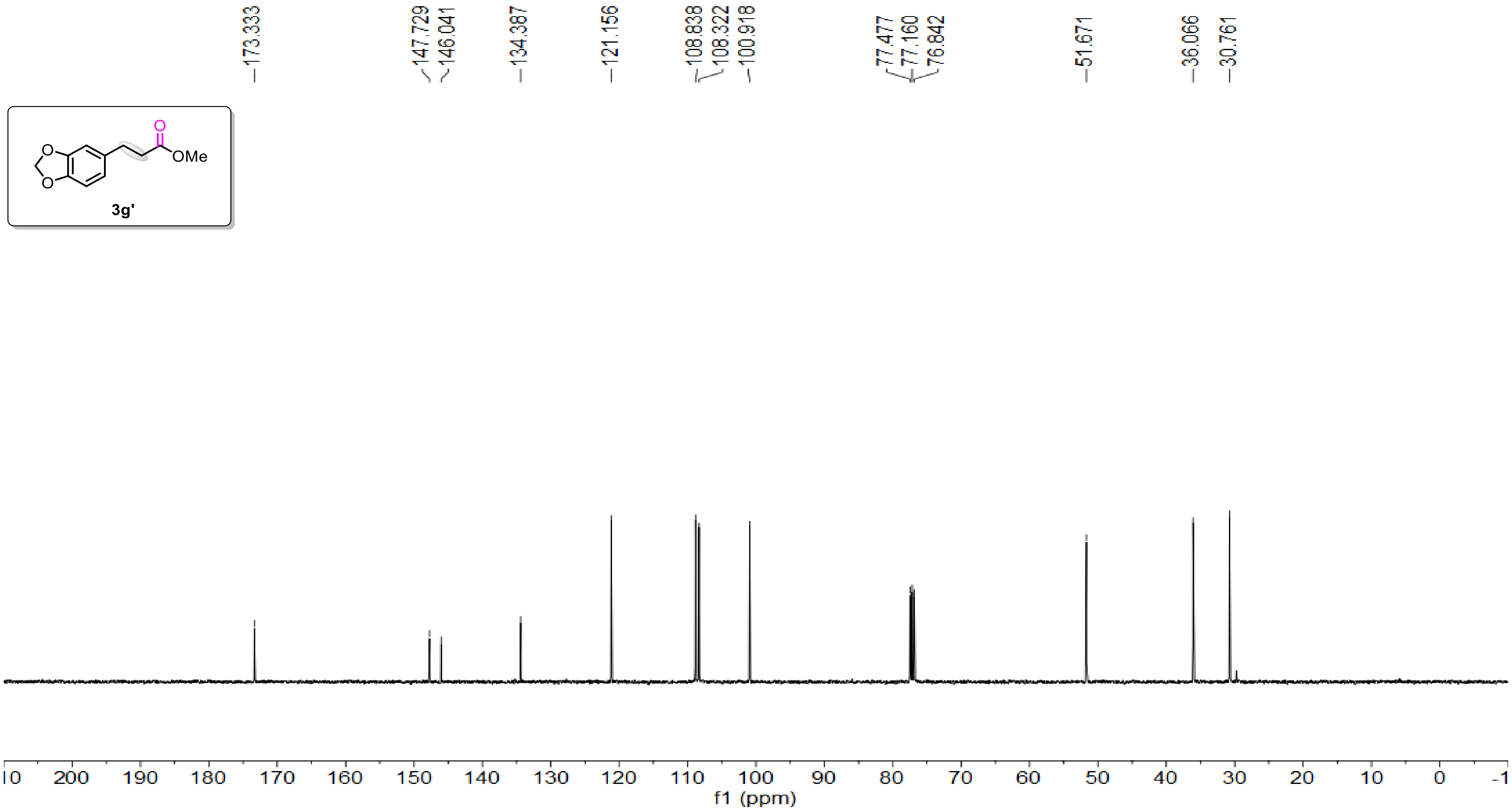

Supplementary Fig. 68. ^{1}H NMR Spectra (400 MHz, $CDCl_3$) of **3h'**

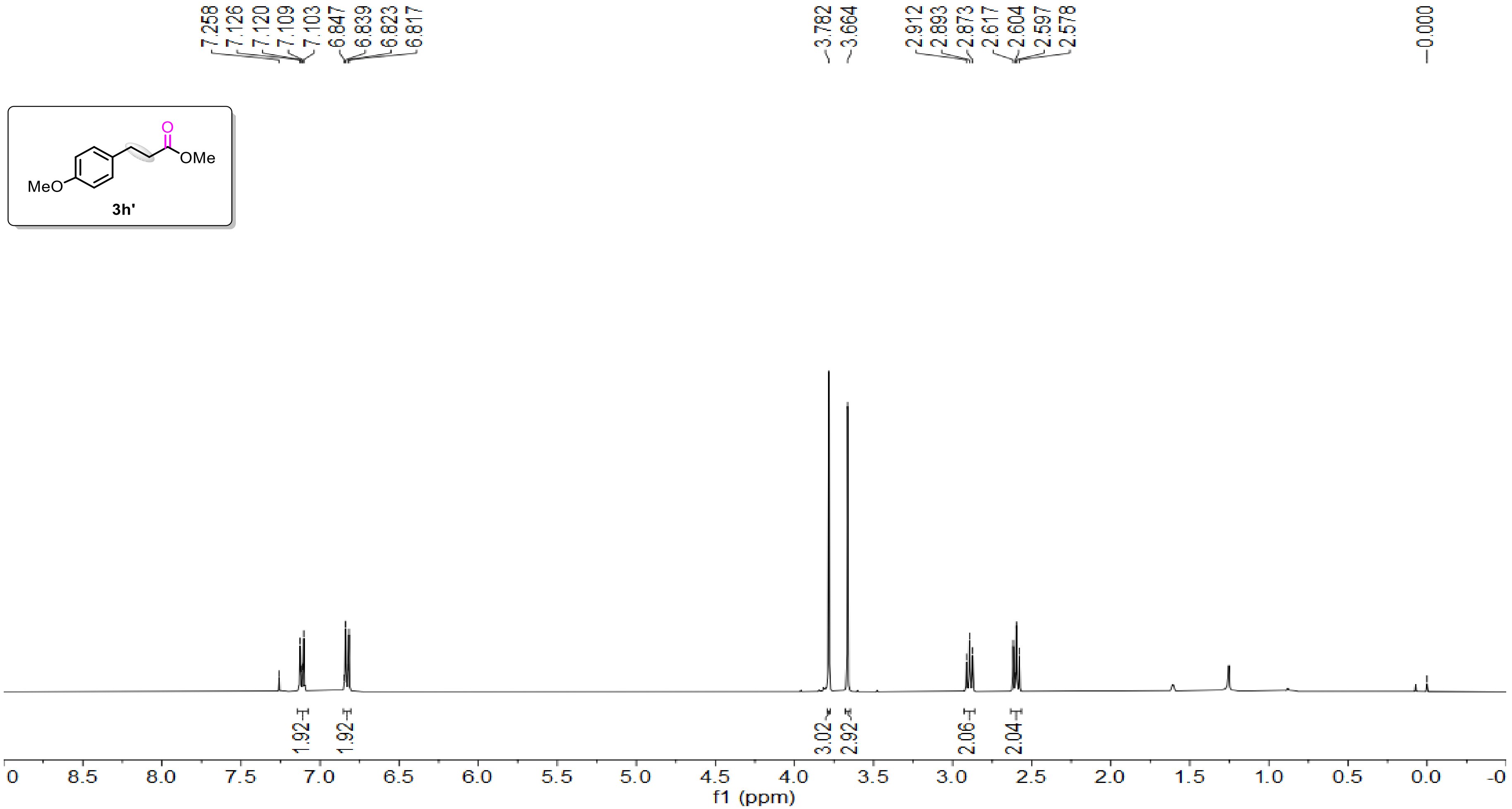

Supplementary Fig. 69. ^{13}C NMR Spectra (101 MHz, CDCl$_3$) of **3h'**

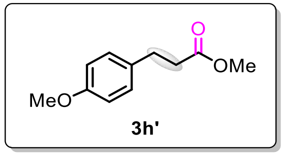

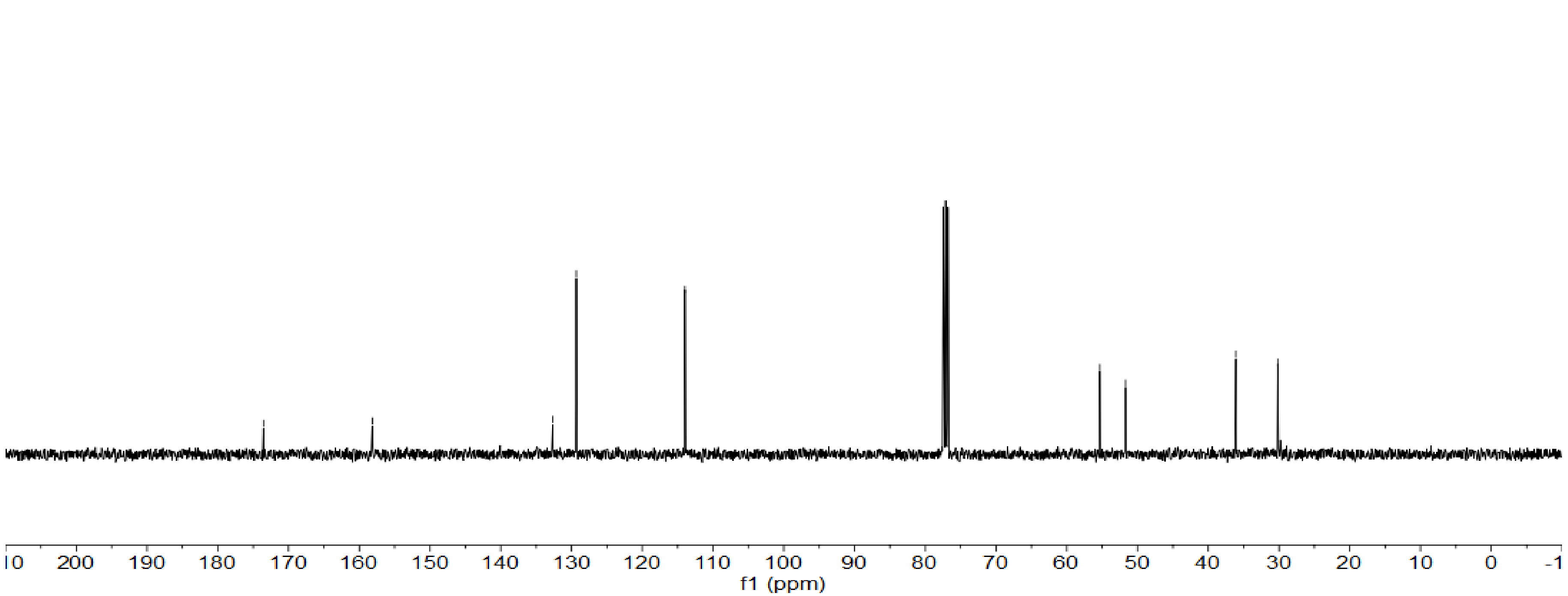

Supplementary Fig. 70. ^{1}H NMR Spectra (400 MHz, $CDCl_3$) of **3i'**

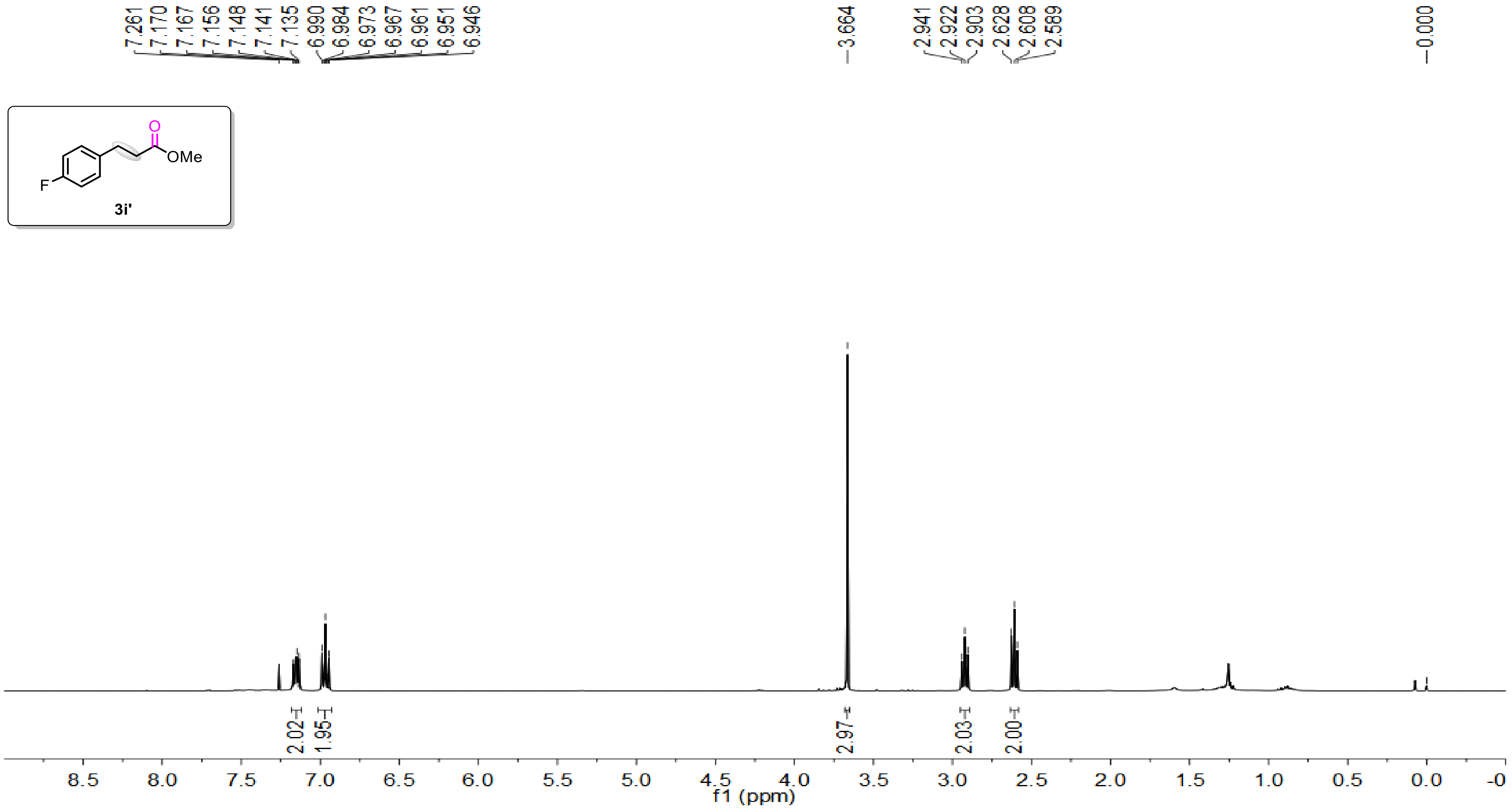

Supplementary Fig. 71. ^{13}C NMR Spectra (101 MHz, $CDCl_3$) of **3i'**

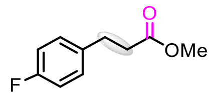

3i'

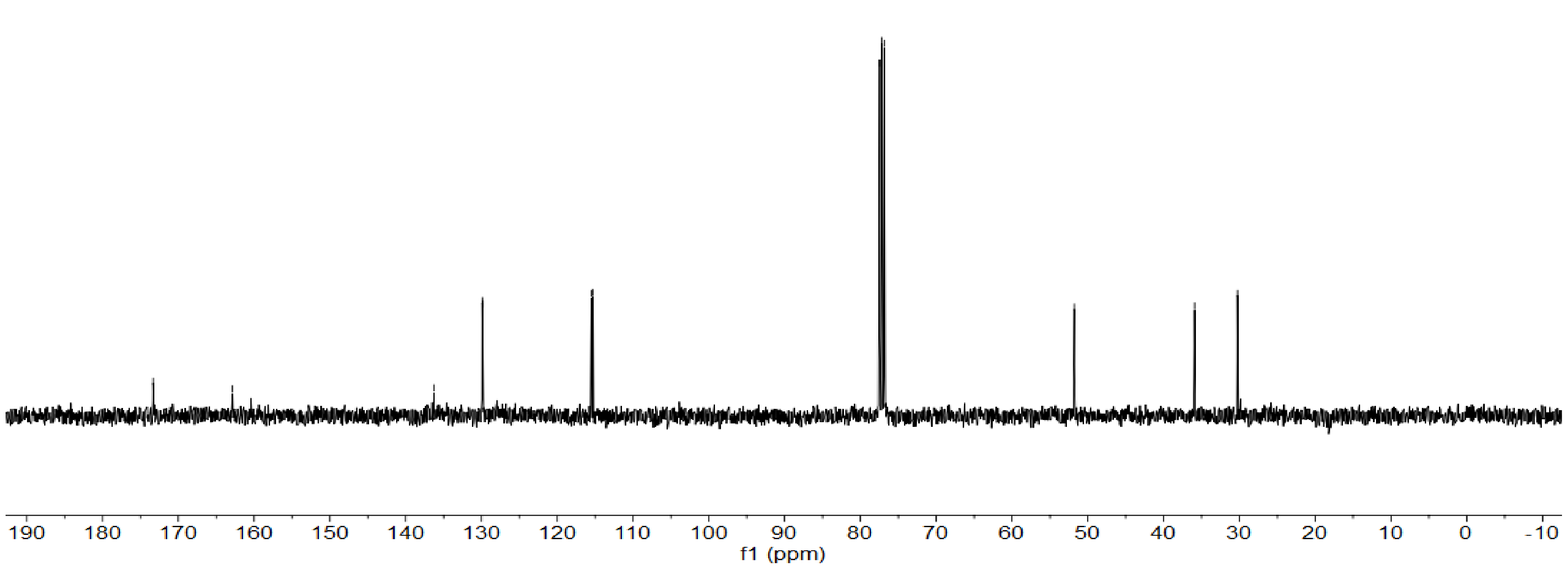

Supplementary Fig. 72. ^{1}H NMR Spectra (400 MHz, $CDCl_3$) of **3j'**

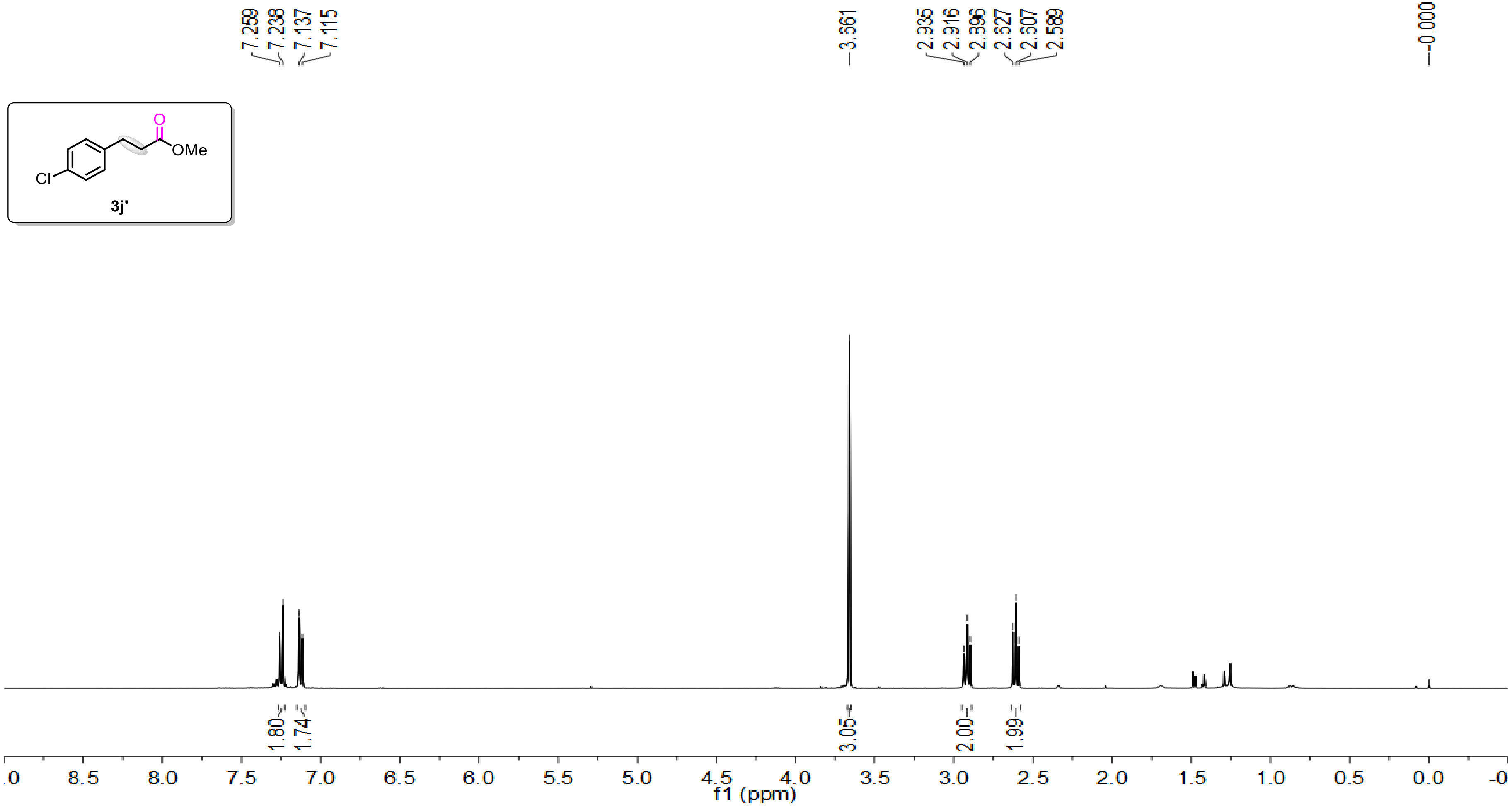

Supplementary Fig. 73. ^{13}C NMR Spectra (101 MHz, $CDCl_3$) of **3j'**

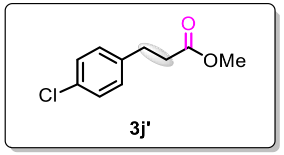

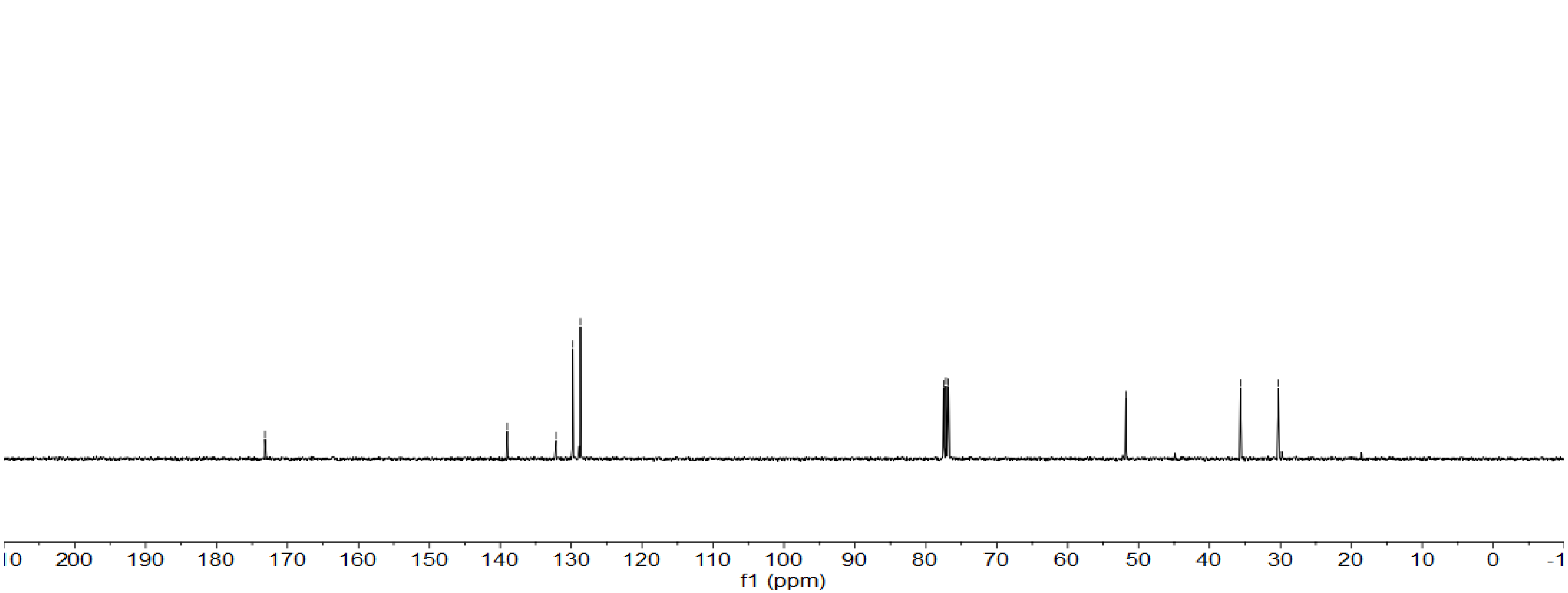

Supplementary Fig. 74. ^{1}H NMR Spectra (400 MHz, $CDCl_3$) of **3k'**

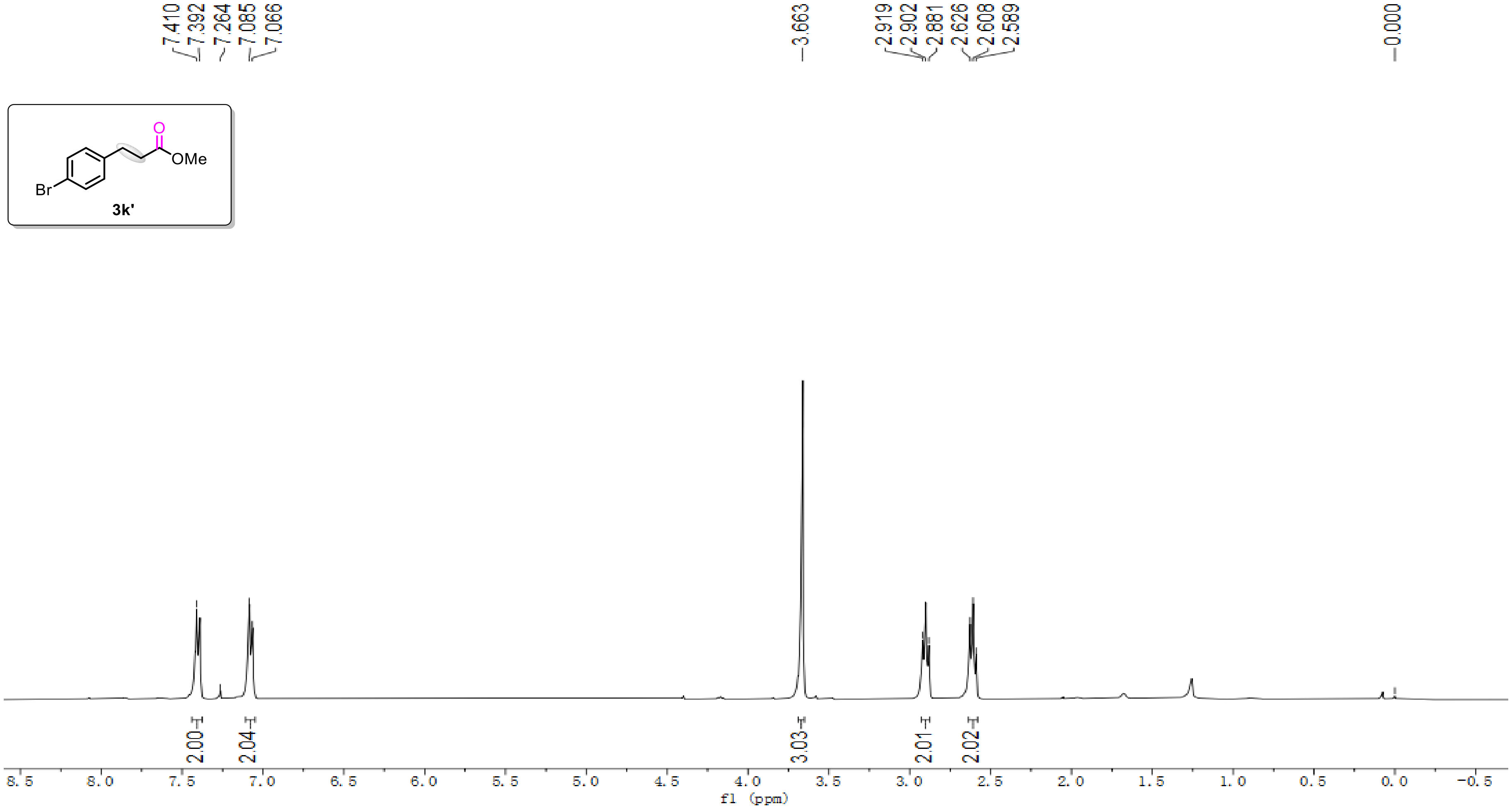

Supplementary Fig. 75. ^{13}C NMR Spectra (101 MHz, $CDCl_3$) of **3k'**

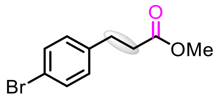

3k'

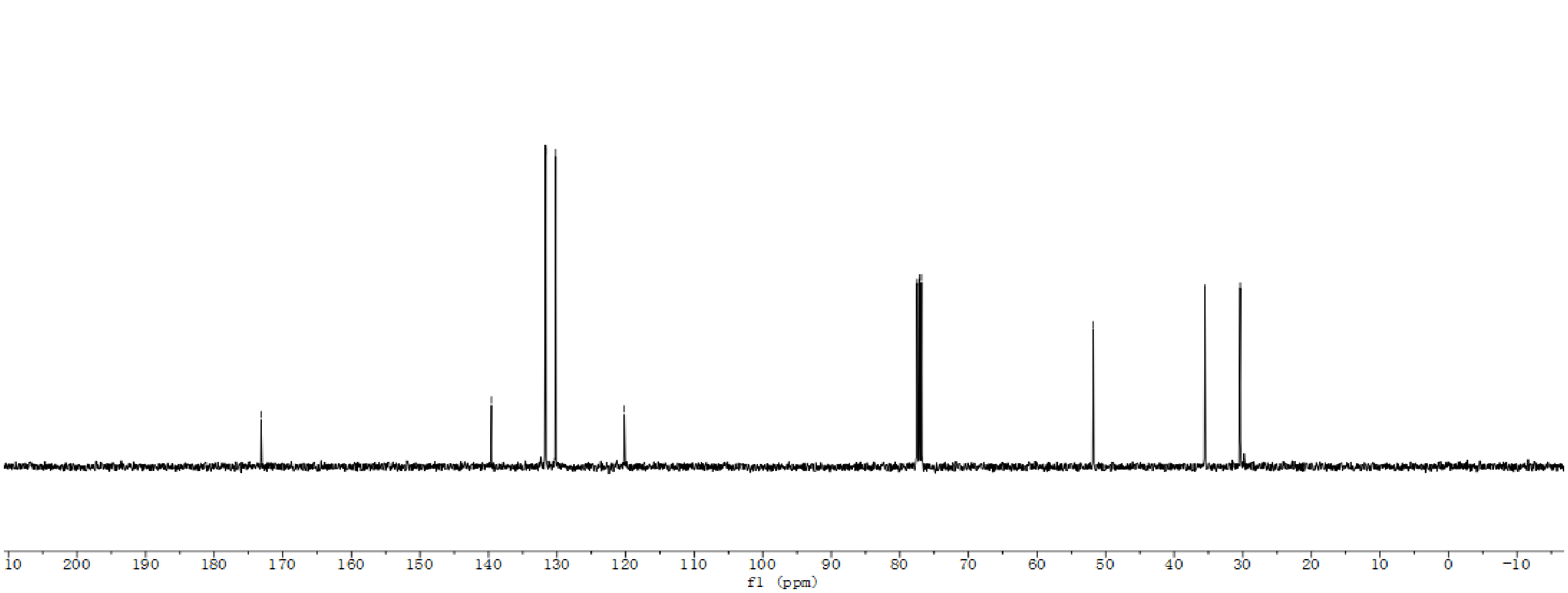

Supplementary Fig. 76. ^{1}H NMR Spectra (400 MHz, $CDCl_3$) of **3l'**

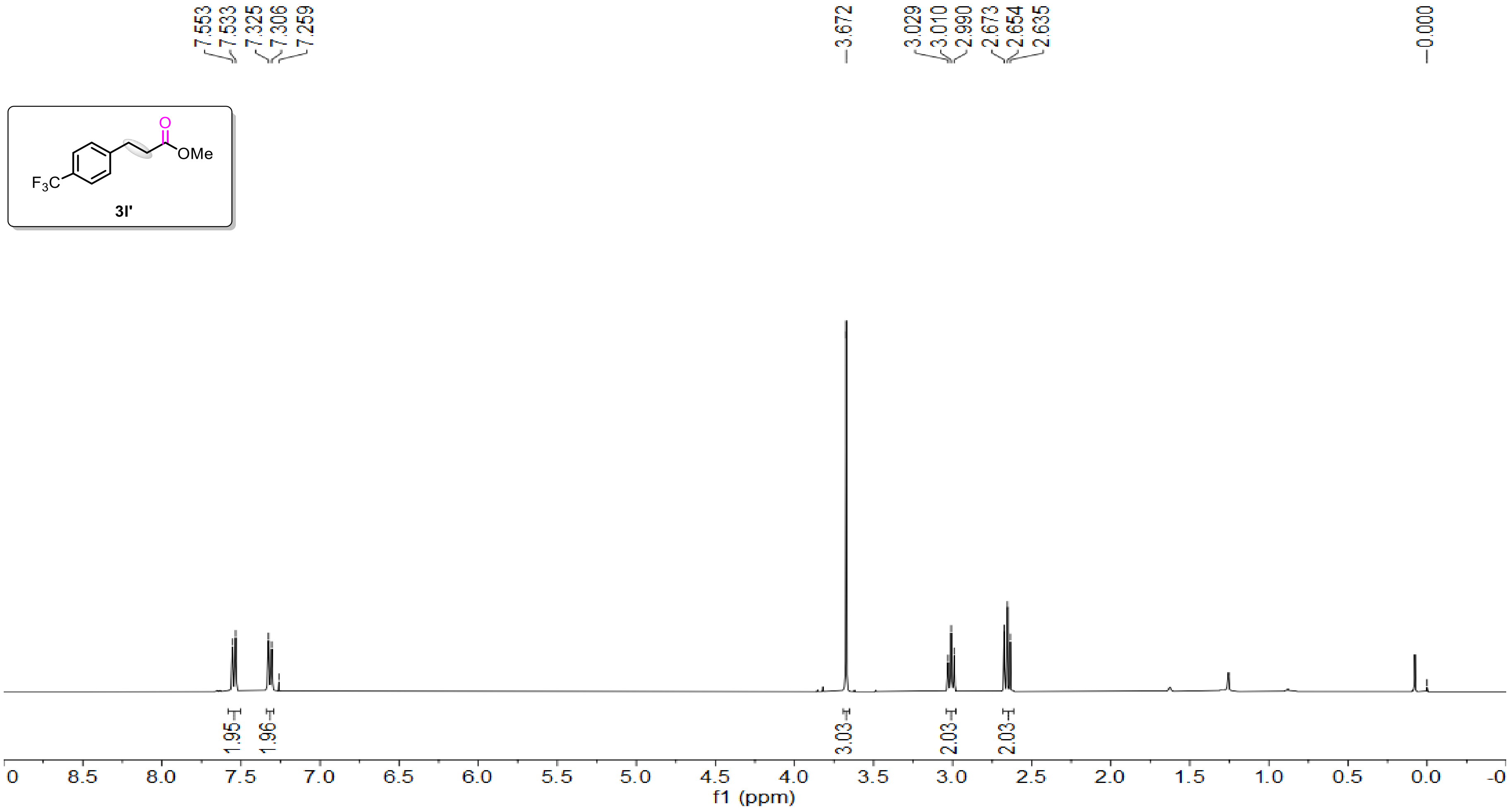

Supplementary Fig. 77. ^{13}C NMR Spectra (101 MHz, $CDCl_3$) of **3l'**

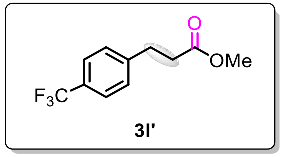

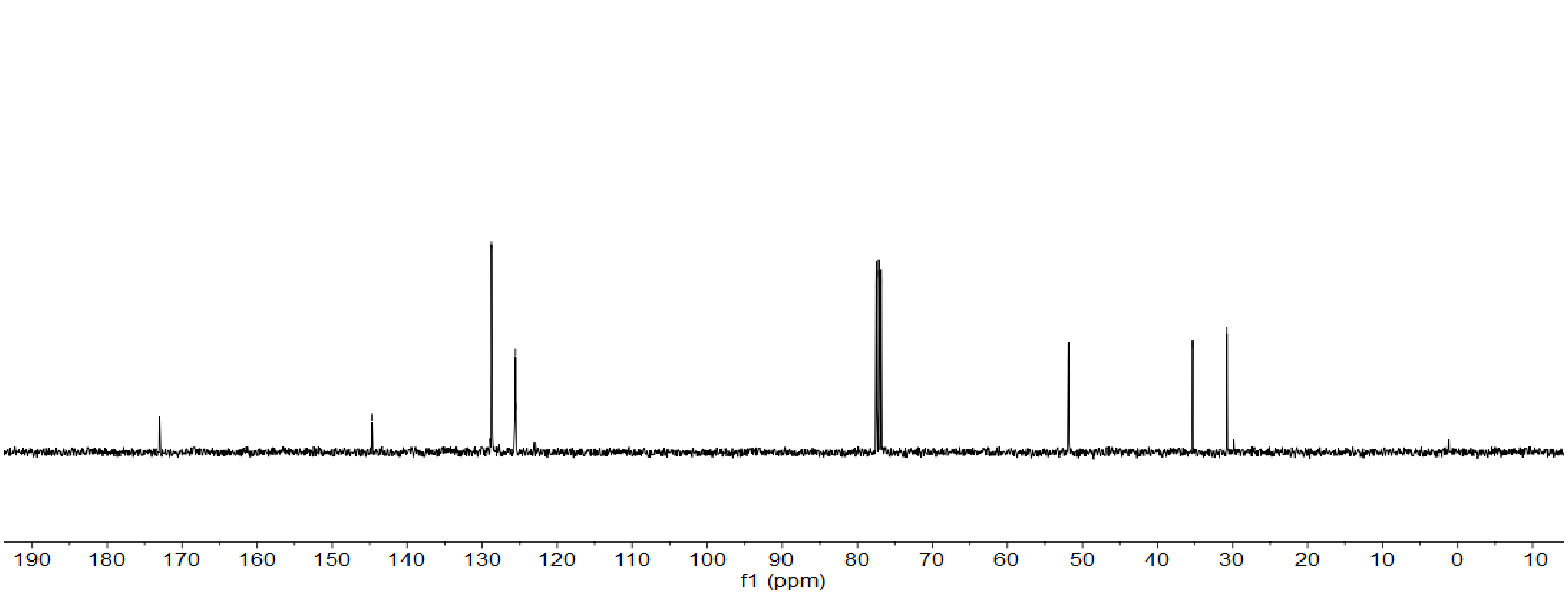

Supplementary Fig. 78. ^1H NMR Spectra (400 MHz, $CDCl_3$) of **3m'**

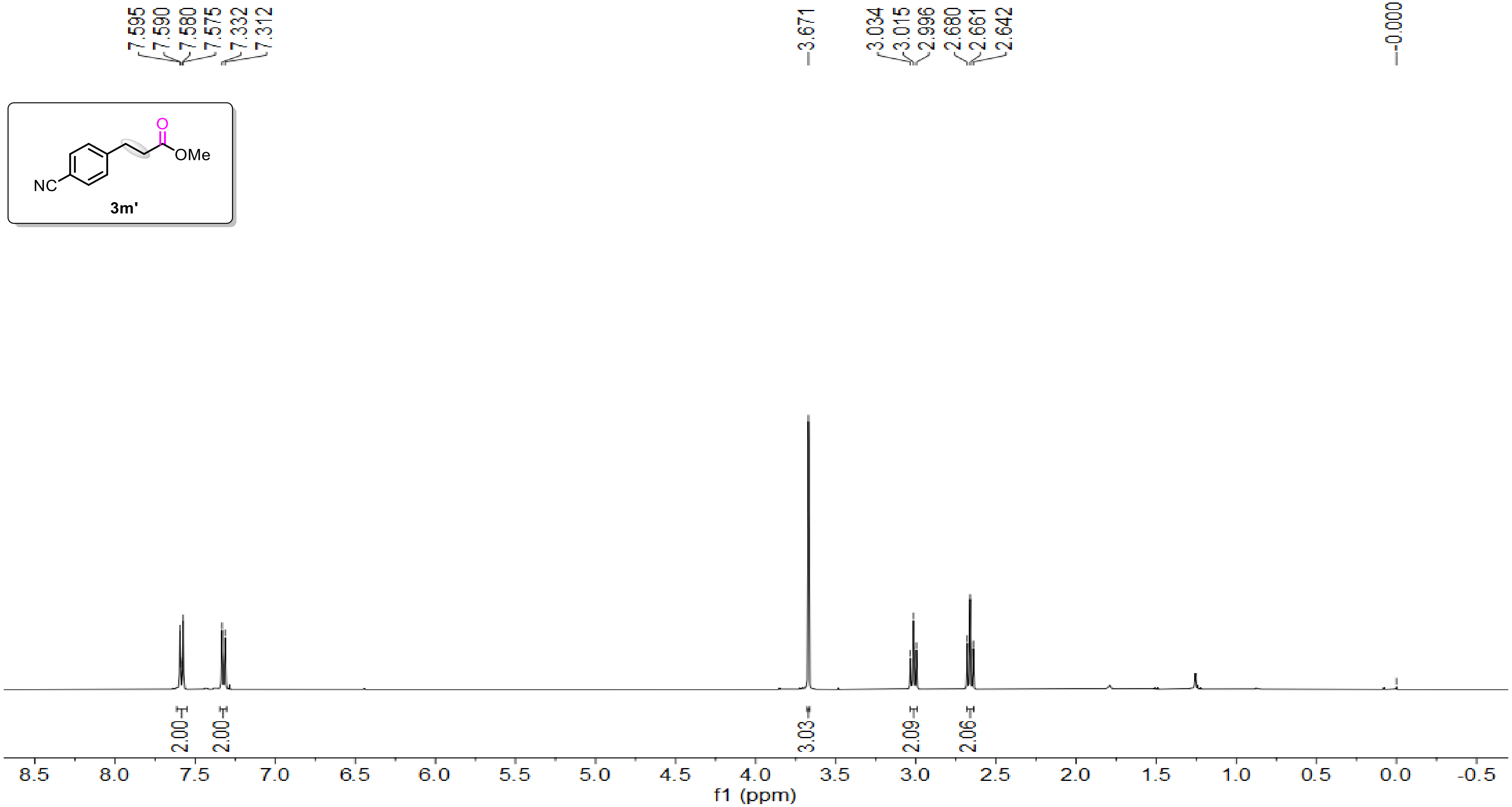

Supplementary Fig. 79. ^{13}C NMR Spectra (101 MHz, $CDCl_3$) of **3m'**

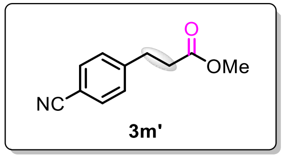

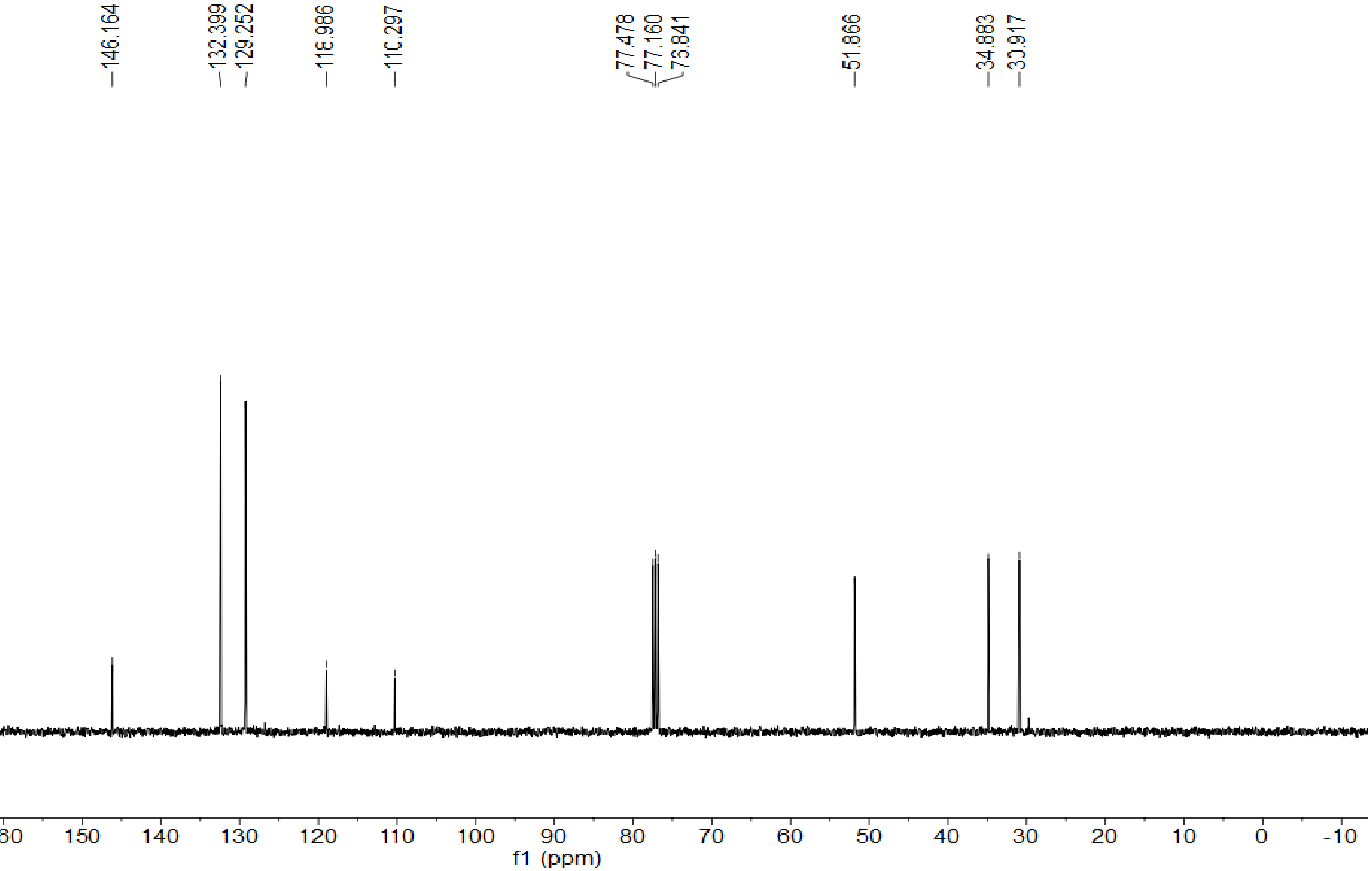

Supplementary Fig. 80. ^{1}H NMR Spectra (400 MHz, $CDCl_3$) of **3n'**

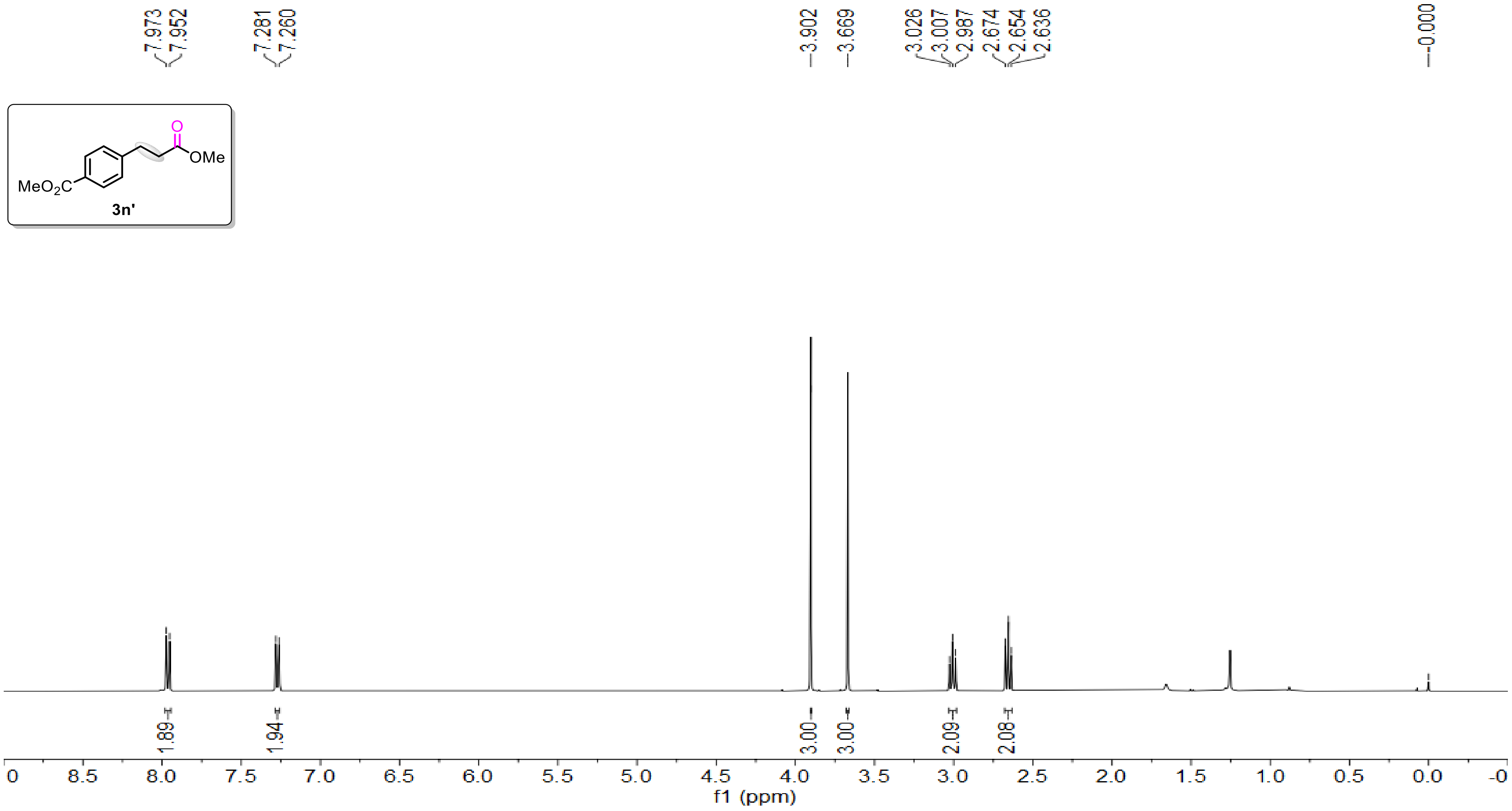

Supplementary Fig. 81. ^{13}C NMR Spectra (101 MHz, $CDCl_3$) of **3n'**

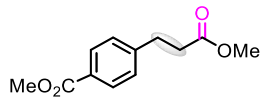

3n'

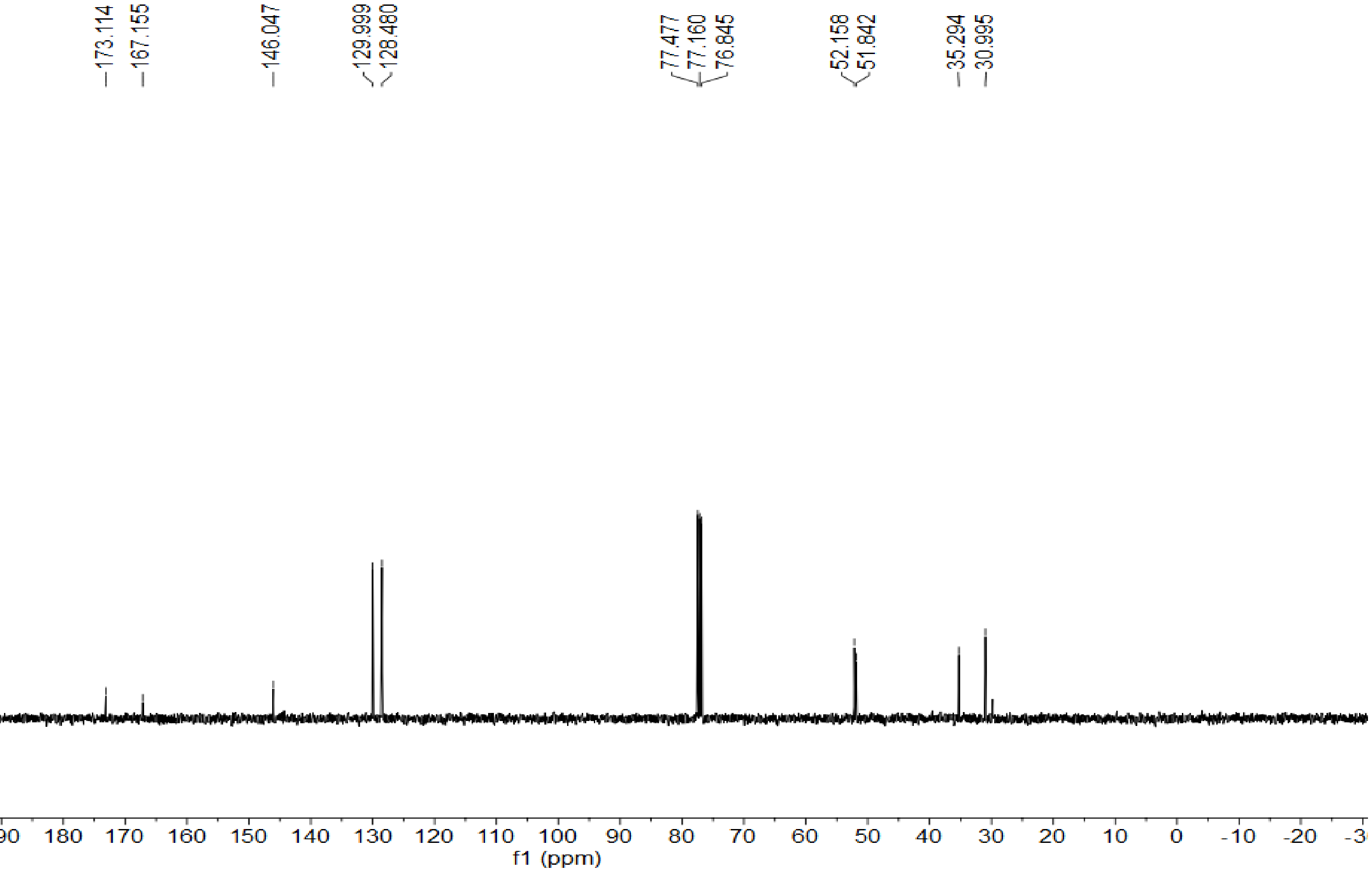

Supplementary Fig. 82. ^{1}H NMR Spectra (400 MHz, $CDCl_3$) of **3o'**

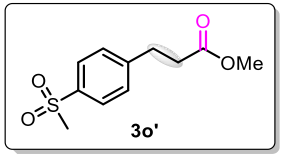

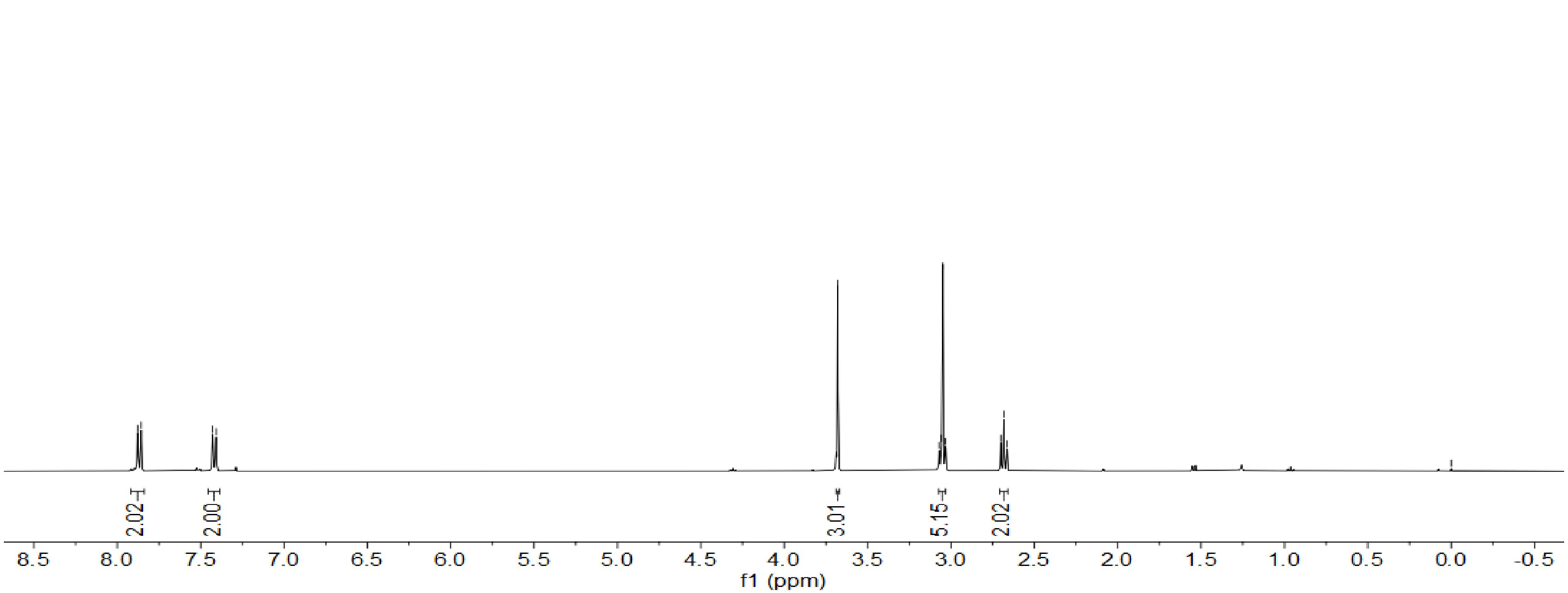

Supplementary Fig. 83. ^{13}C NMR Spectra (101 MHz, $CDCl_3$) of **3o'**

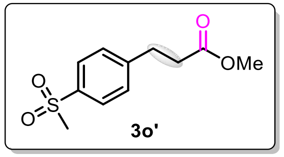

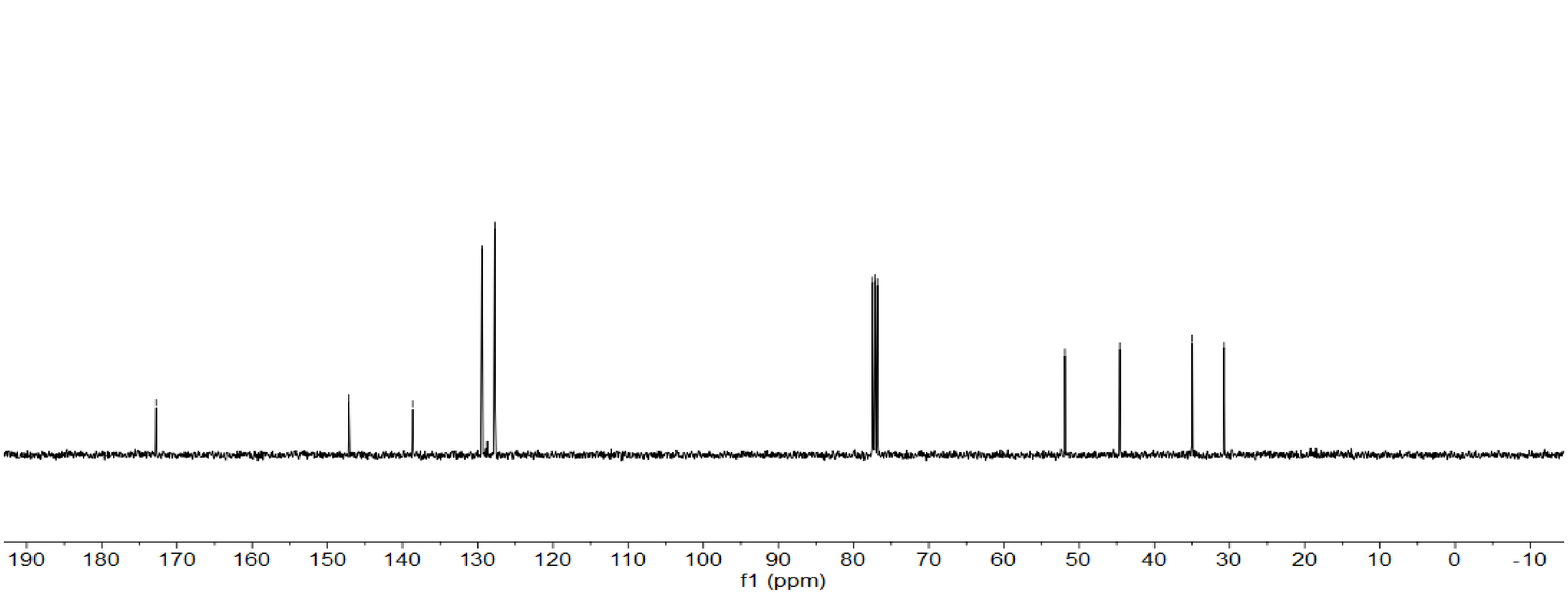

Supplementary Fig. 84. ^{1}H NMR Spectra (400 MHz, $CDCl_3$) of **3p'**

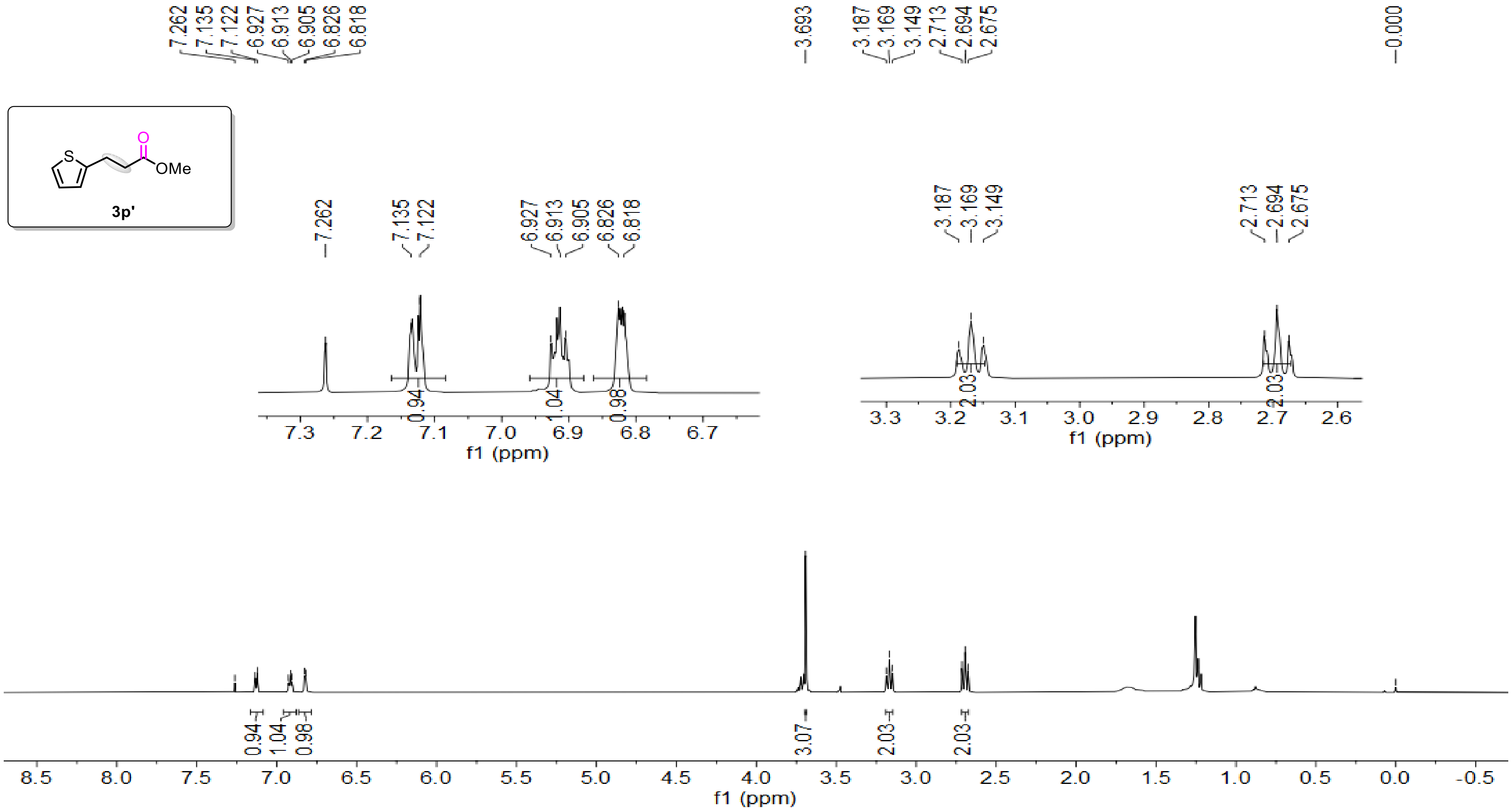

Supplementary Fig. 85. ^{13}C NMR Spectra (101 MHz, $CDCl_3$) of **3p'**

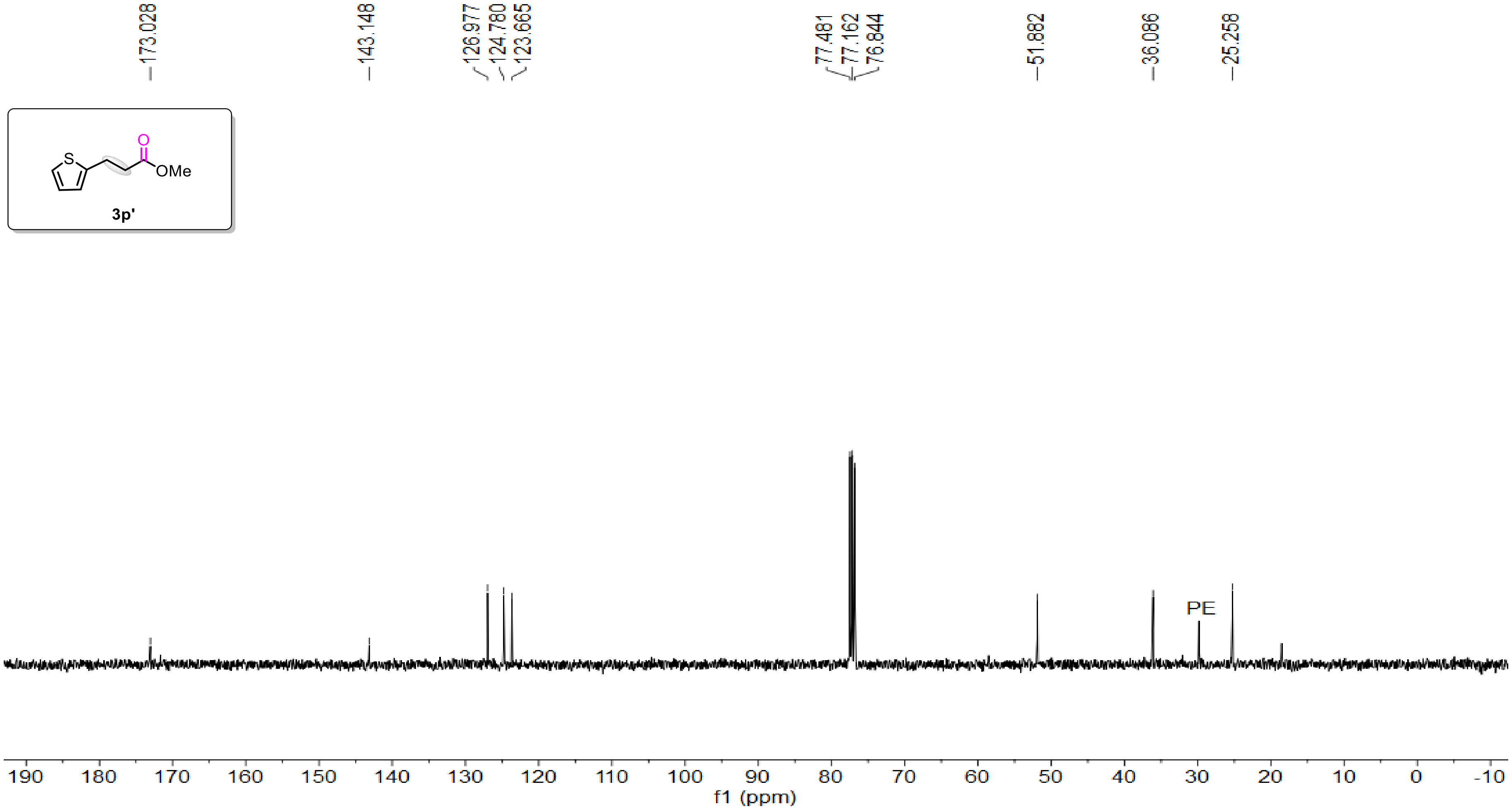

Supplementary Fig. 86. ^{1}H NMR Spectra (400 MHz, $CDCl_3$) of **3q'**

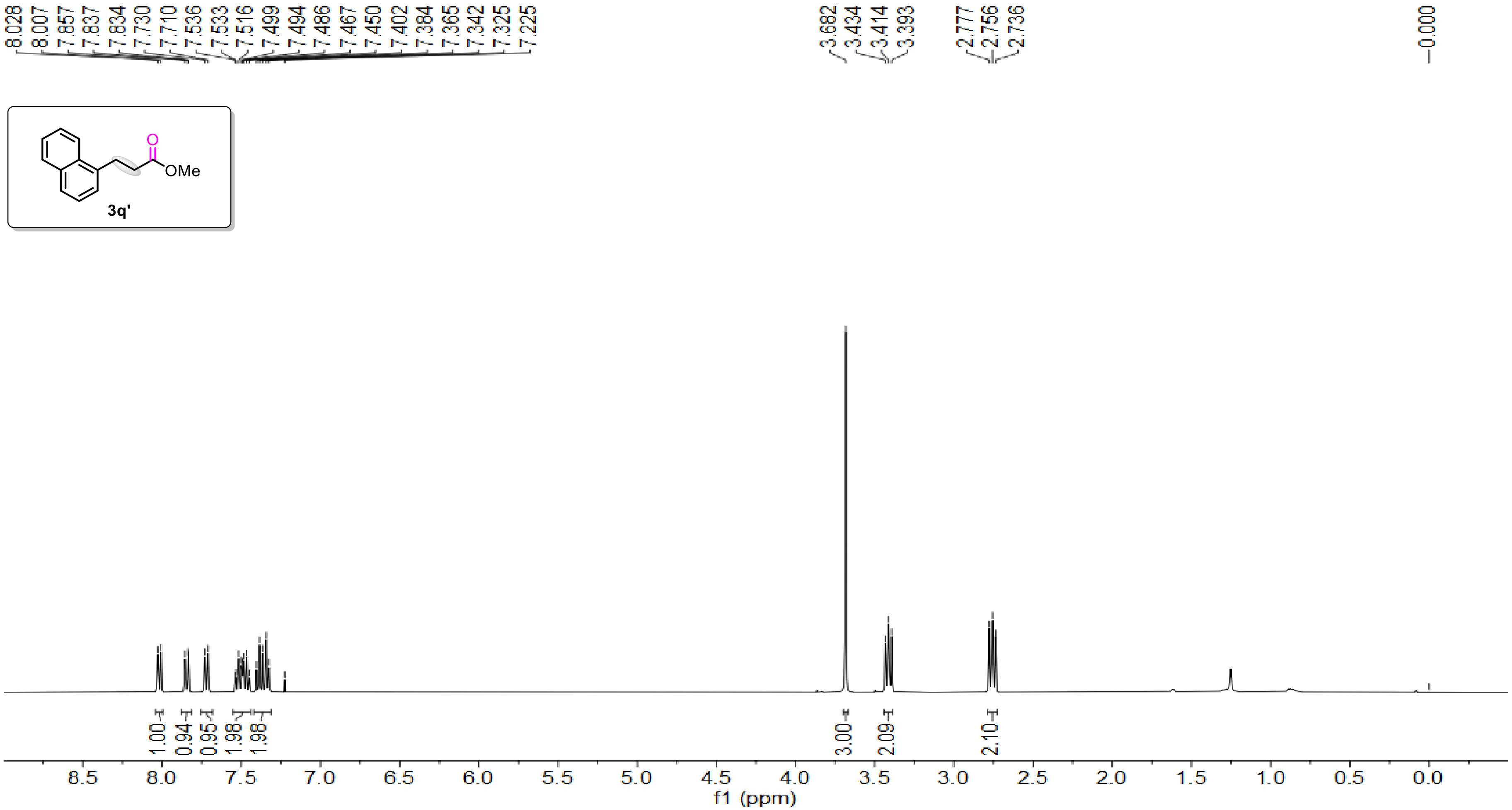

Supplementary Fig. 87. ^{13}C NMR Spectra (101 MHz, $CDCl_3$) of **3q'**

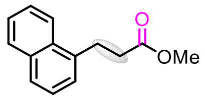

3q'

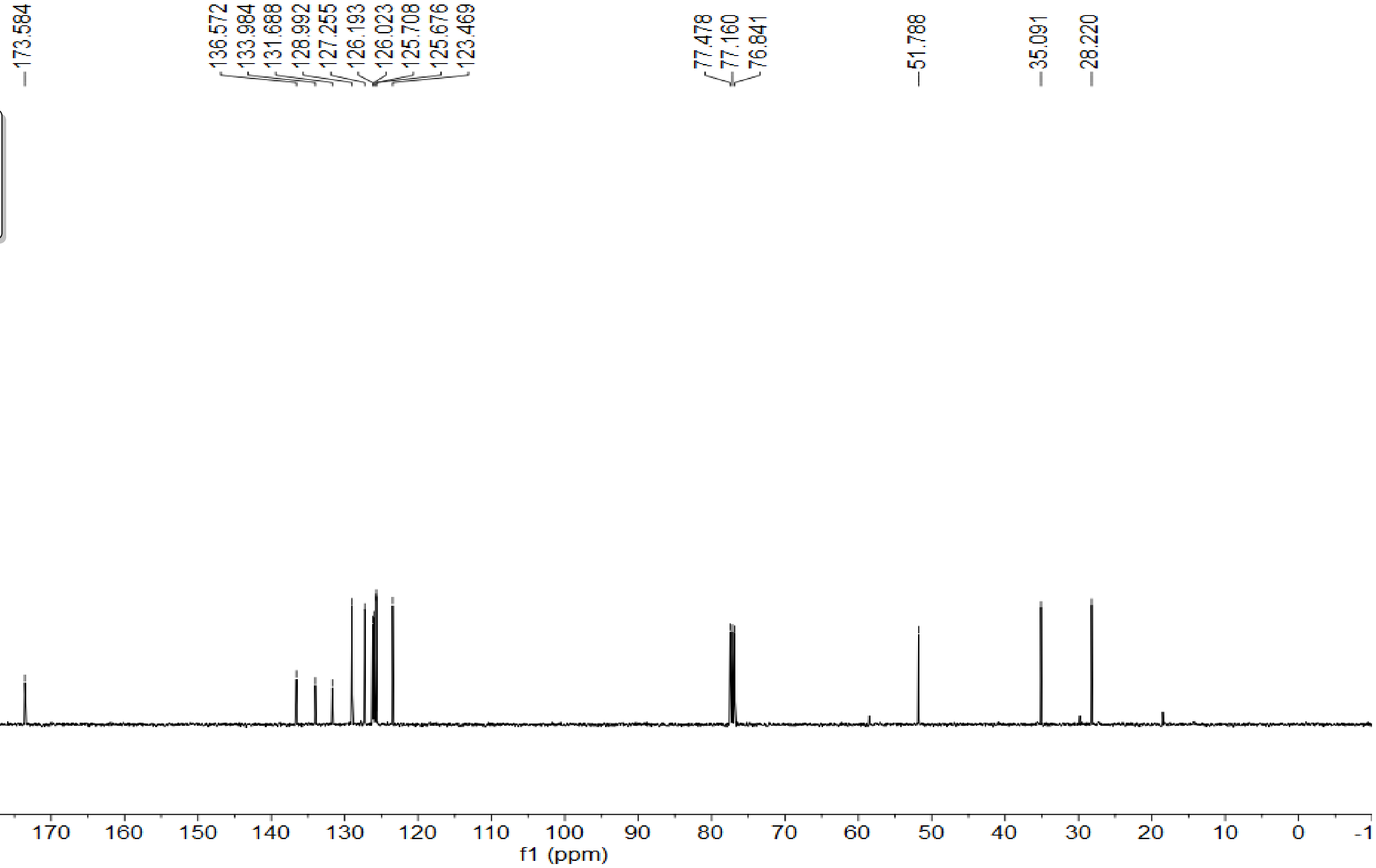

Supplementary Fig. 88. ^{1}H NMR Spectra (400 MHz, $CDCl_3$) of **3r'**

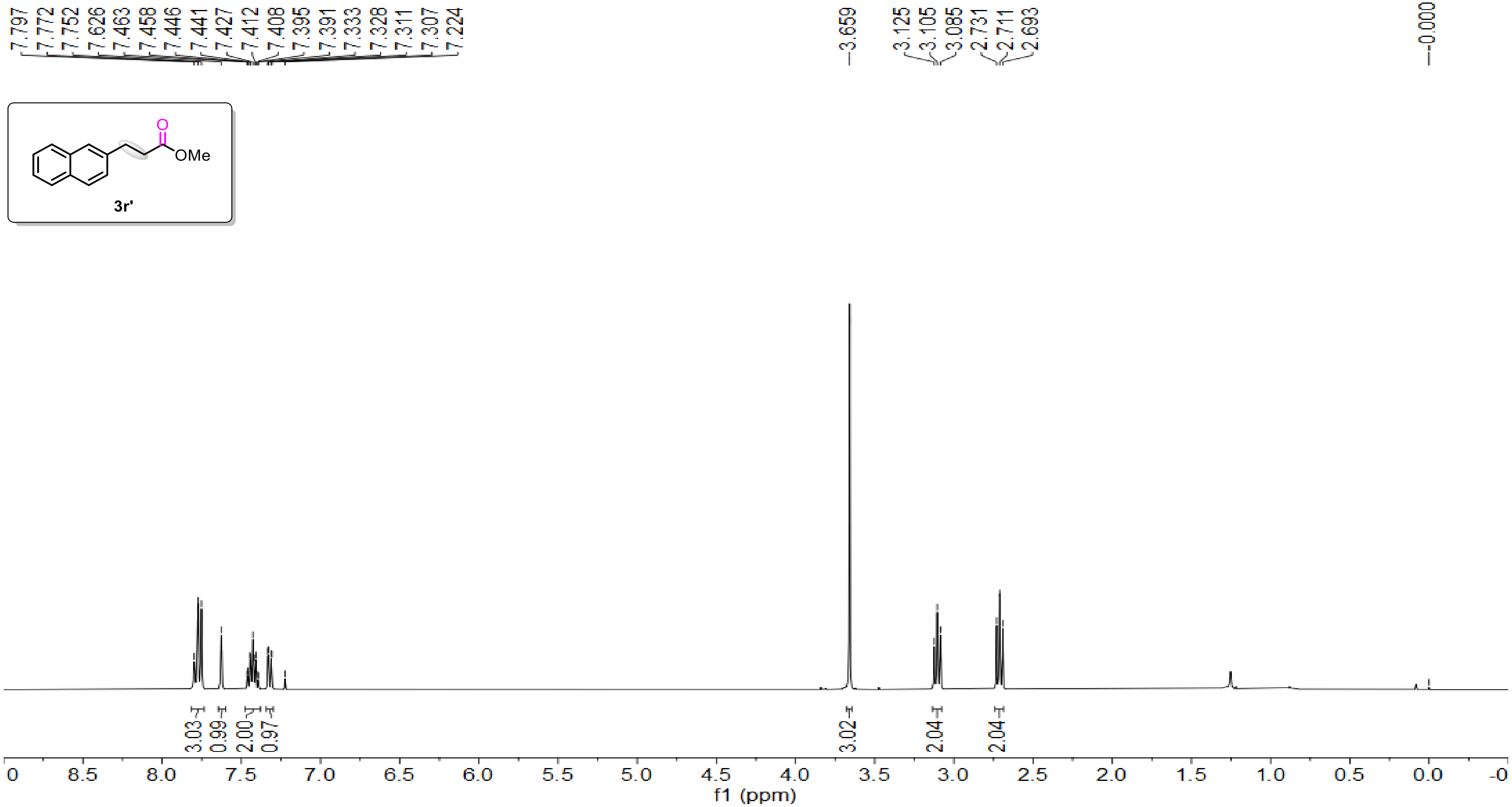

Supplementary Fig. 89. ^{13}C NMR Spectra (101 MHz, $CDCl_3$) of **3r'**

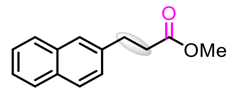

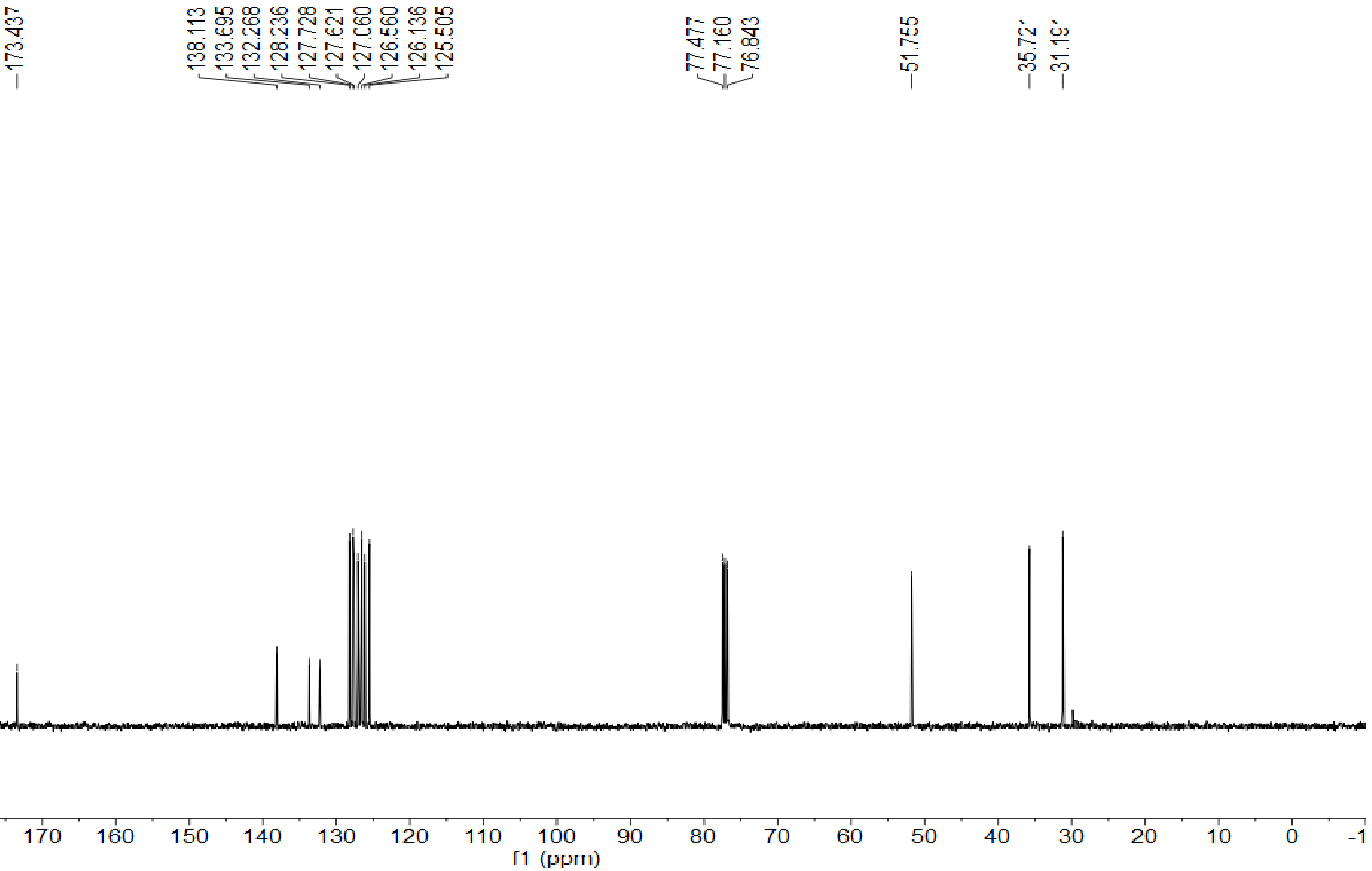

Supplementary Fig. 90. ^{1}H NMR Spectra (400 MHz, $CDCl_3$) of **4a'**

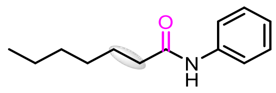

4a'

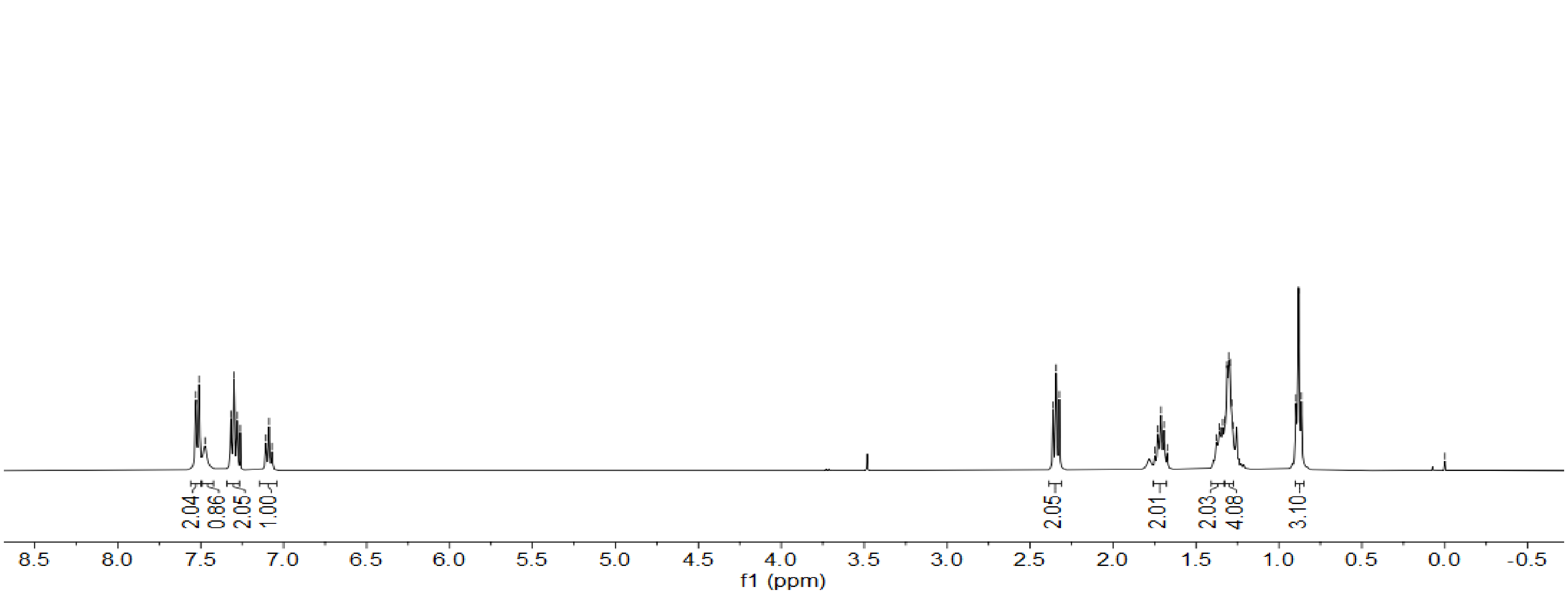

Supplementary Fig. 91. ^{13}C NMR Spectra (101 MHz, $CDCl_3$) of **4a'**

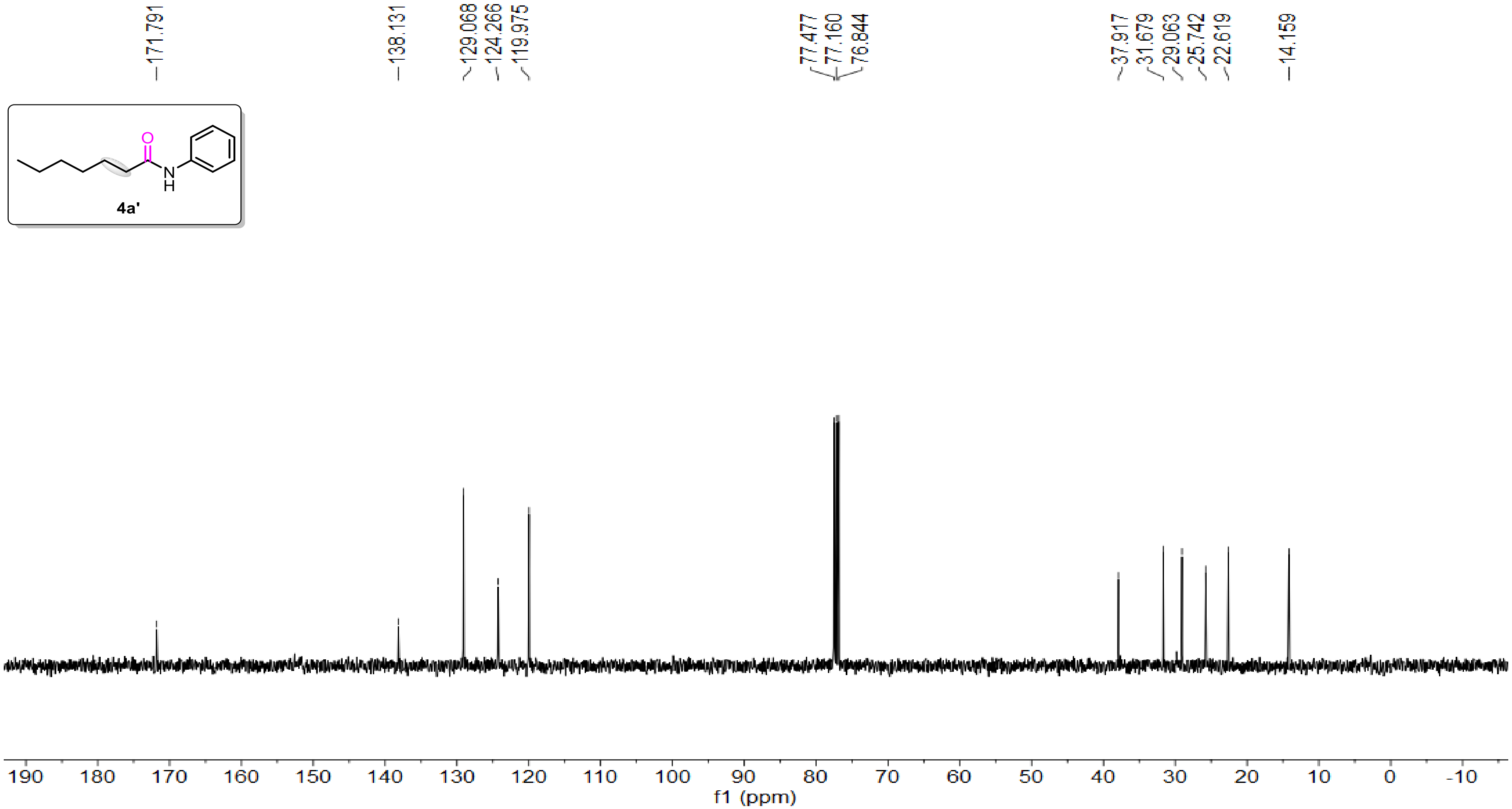

Supplementary Fig. 92. ^{1}H NMR Spectra (400 MHz, $CDCl_3$) of **4b'**

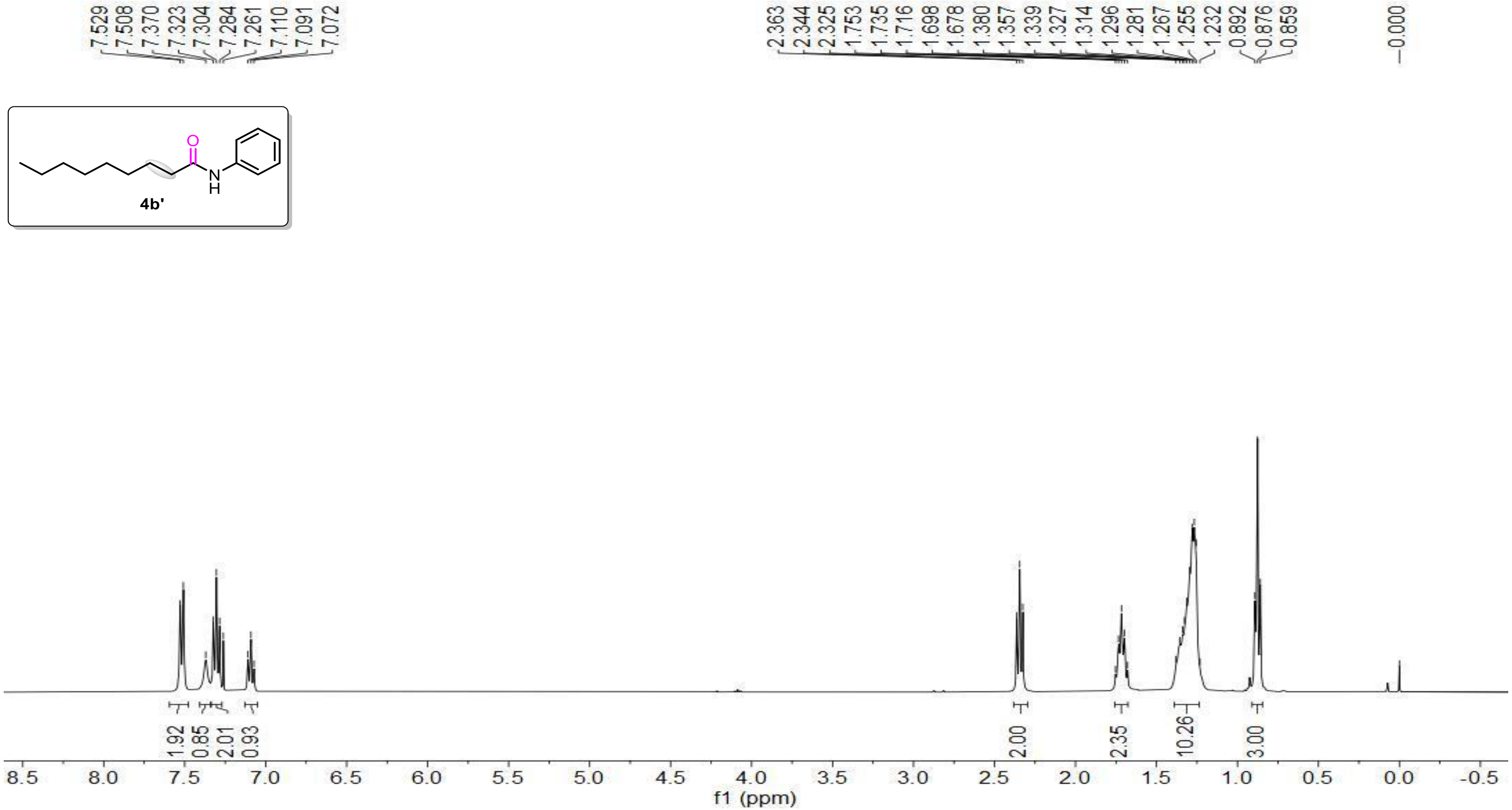

Supplementary Fig. 93. ^{13}C NMR Spectra (101 MHz, $CDCl_3$) of **4b'**

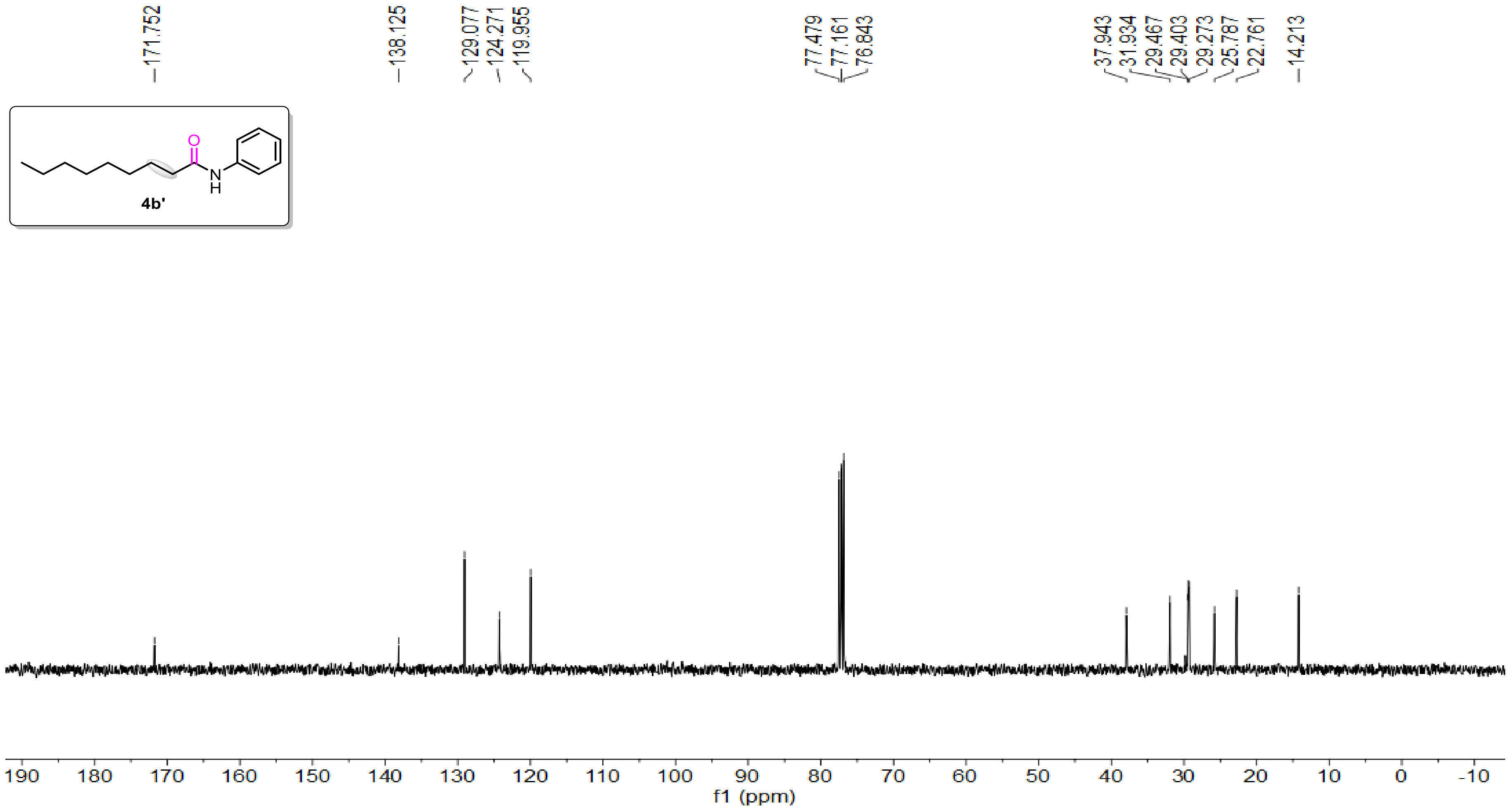

Supplementary Fig. 94. ^1H NMR Spectra (400 MHz, $CDCl_3$) of **4c'**

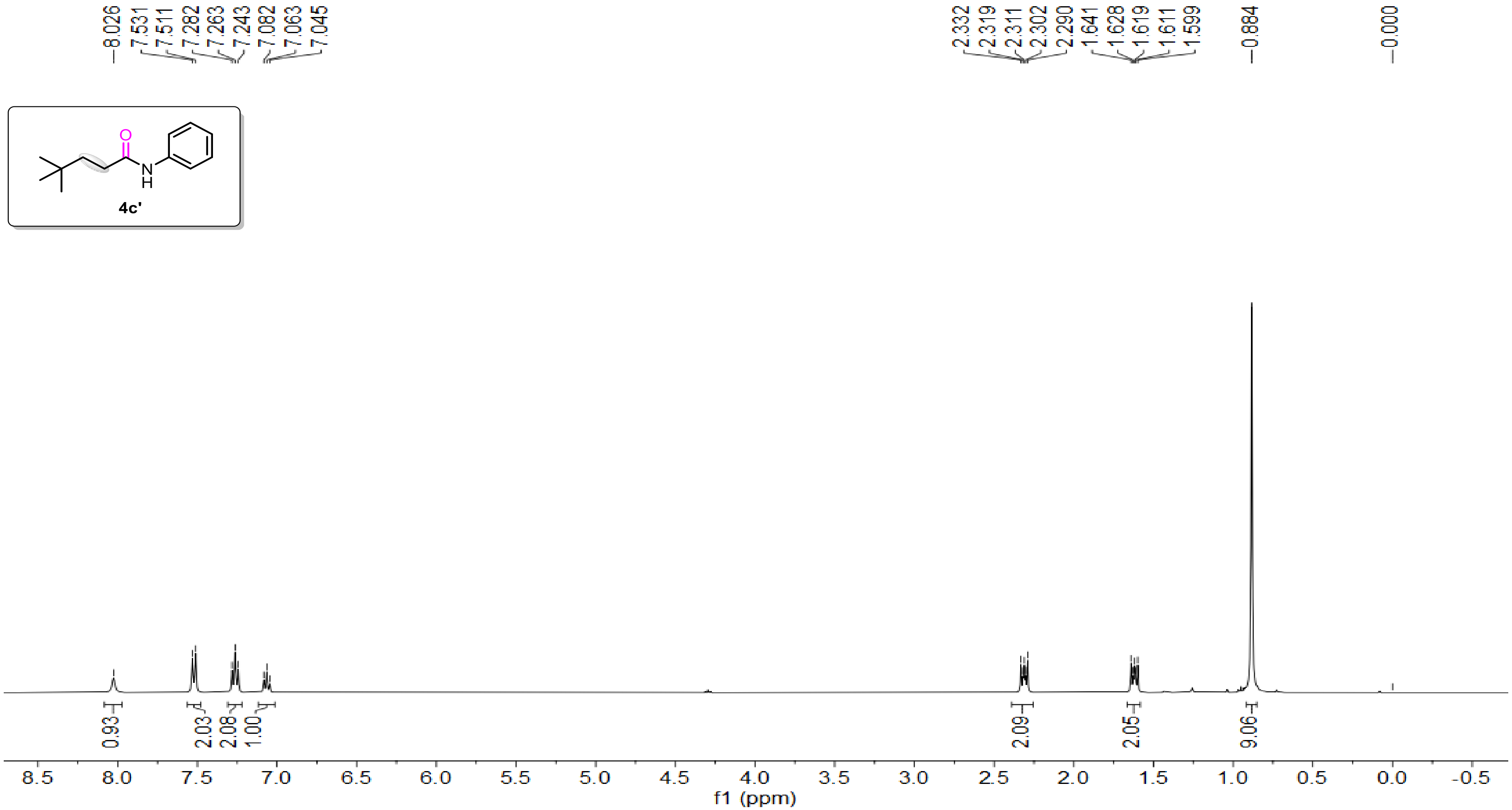

Supplementary Fig. 95. ^{13}C NMR Spectra (101 MHz, $CDCl_3$) of **4c'**

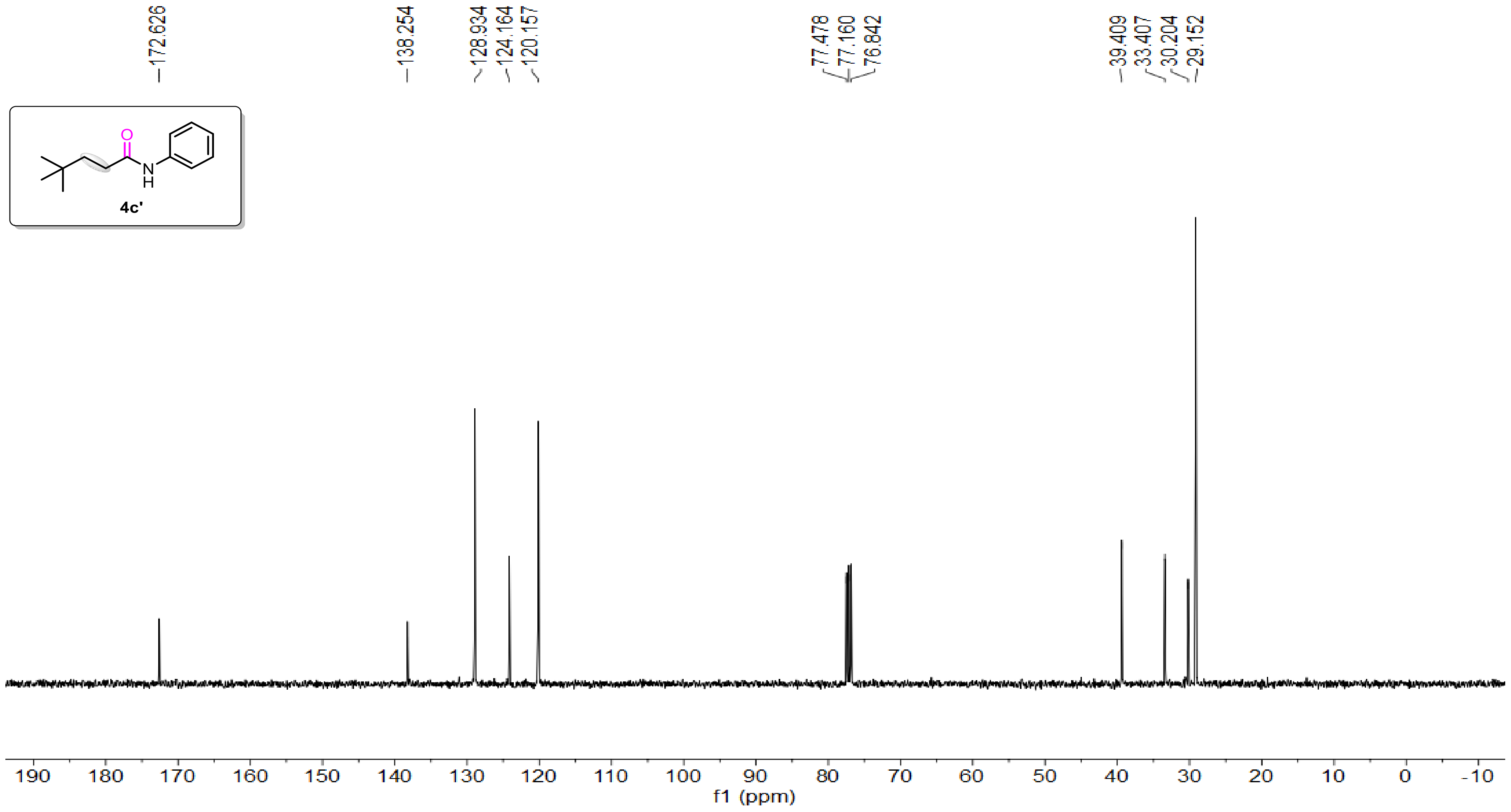

Supplementary Fig. 96. ^{1}H NMR Spectra (400 MHz, $CDCl_3$) of **4d'**

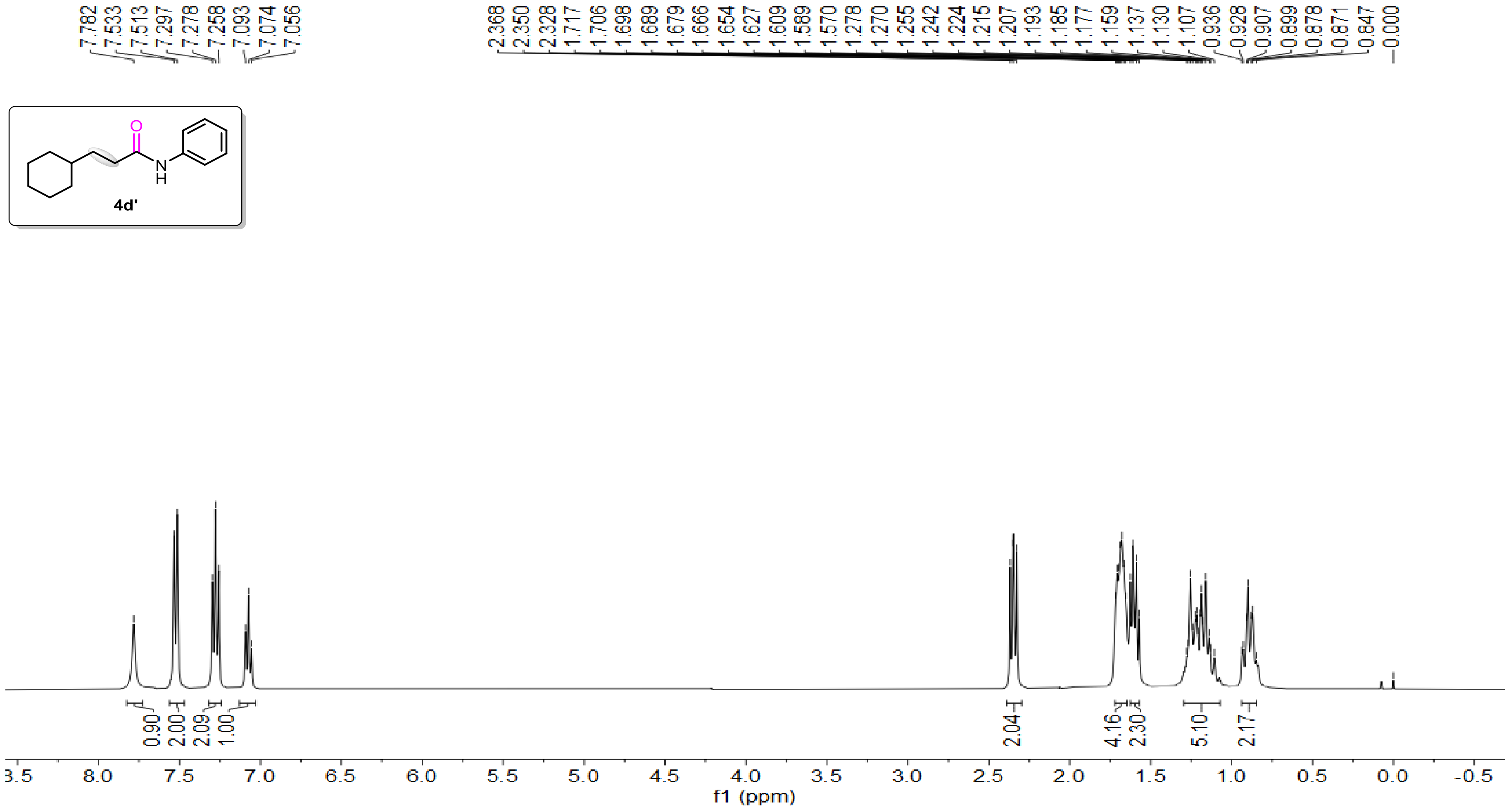

Supplementary Fig. 97. ^{13}C NMR Spectra (101 MHz, $CDCl_3$) of **4d'**

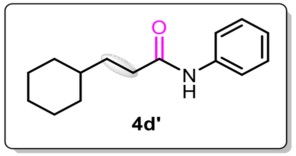

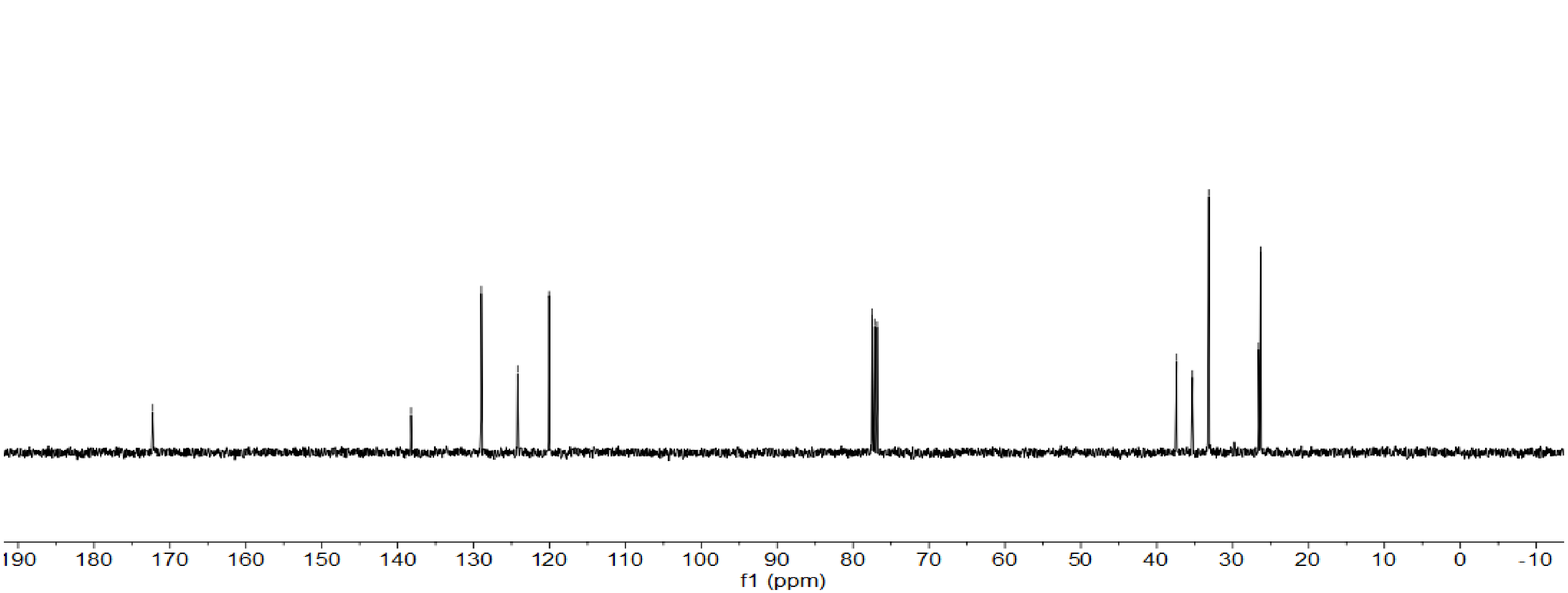

Supplementary Fig. 98. ^{1}H NMR Spectra (400 MHz, $CDCl_3$) of **4e'**

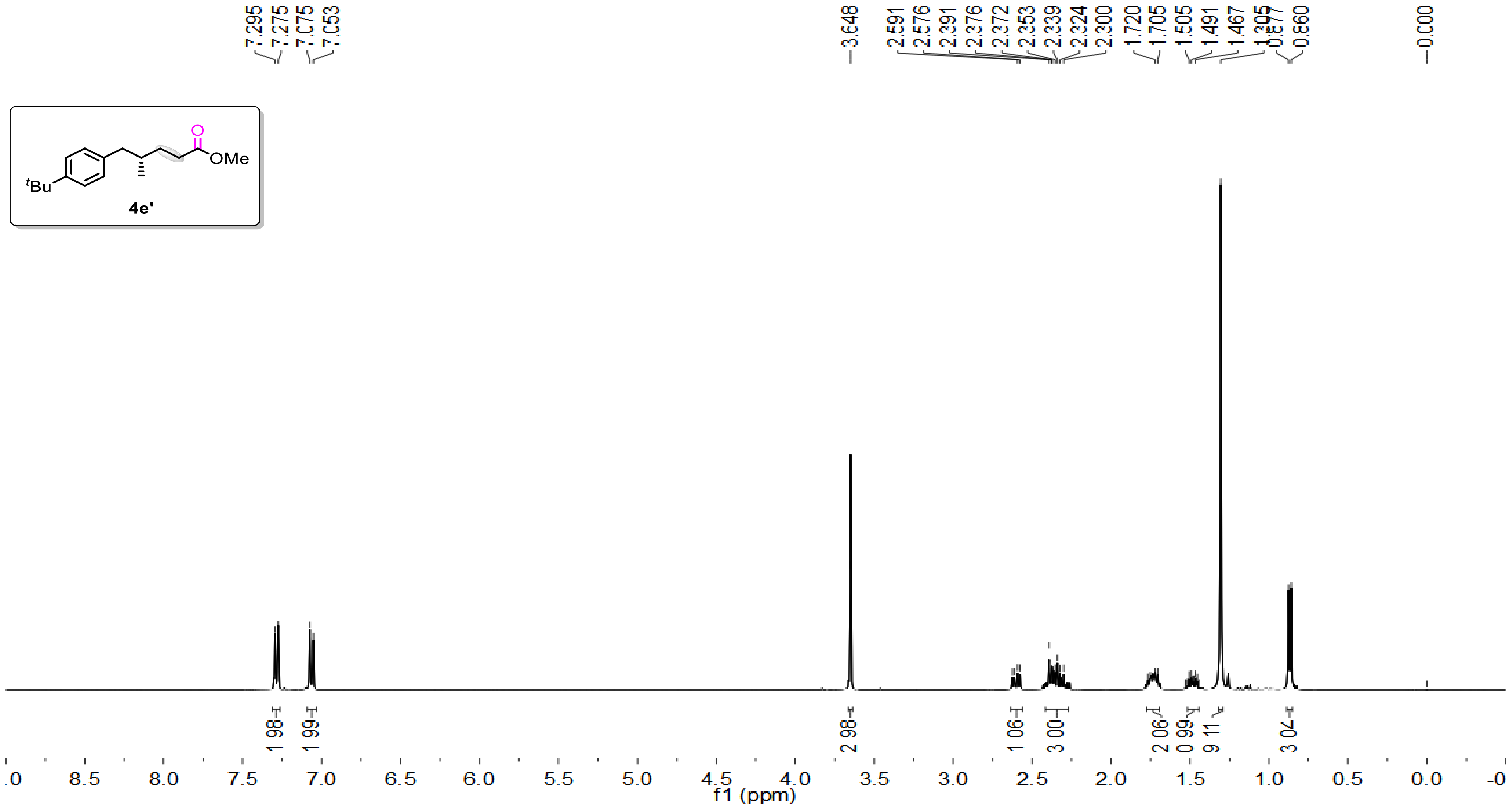

Supplementary Fig. 99. ^{13}C NMR Spectra (101 MHz, $CDCl_3$) of **4e'**

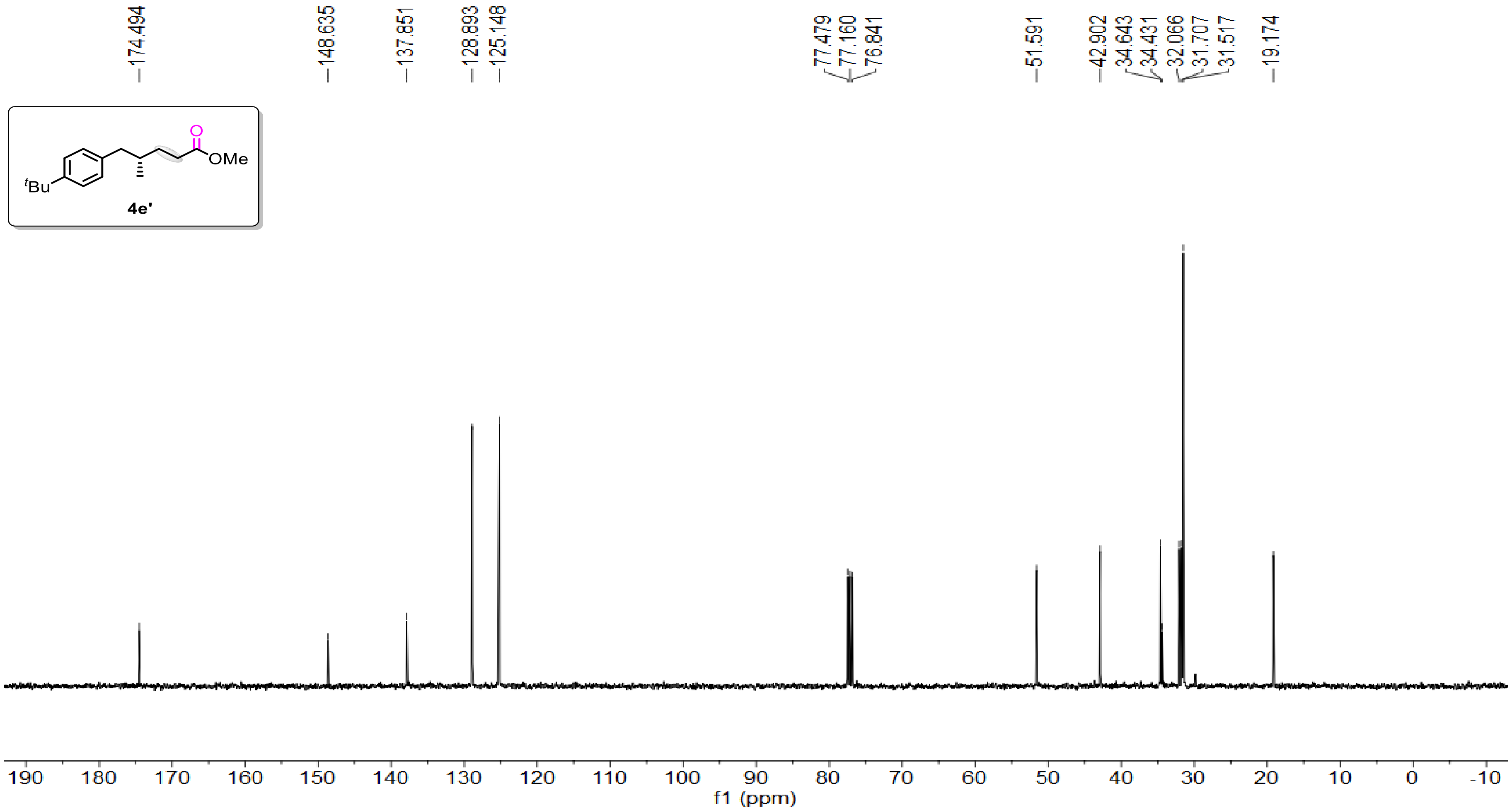

Supplementary Fig. 100. ^{1}H NMR Spectra (400 MHz, $CDCl_3$) of **4f'**

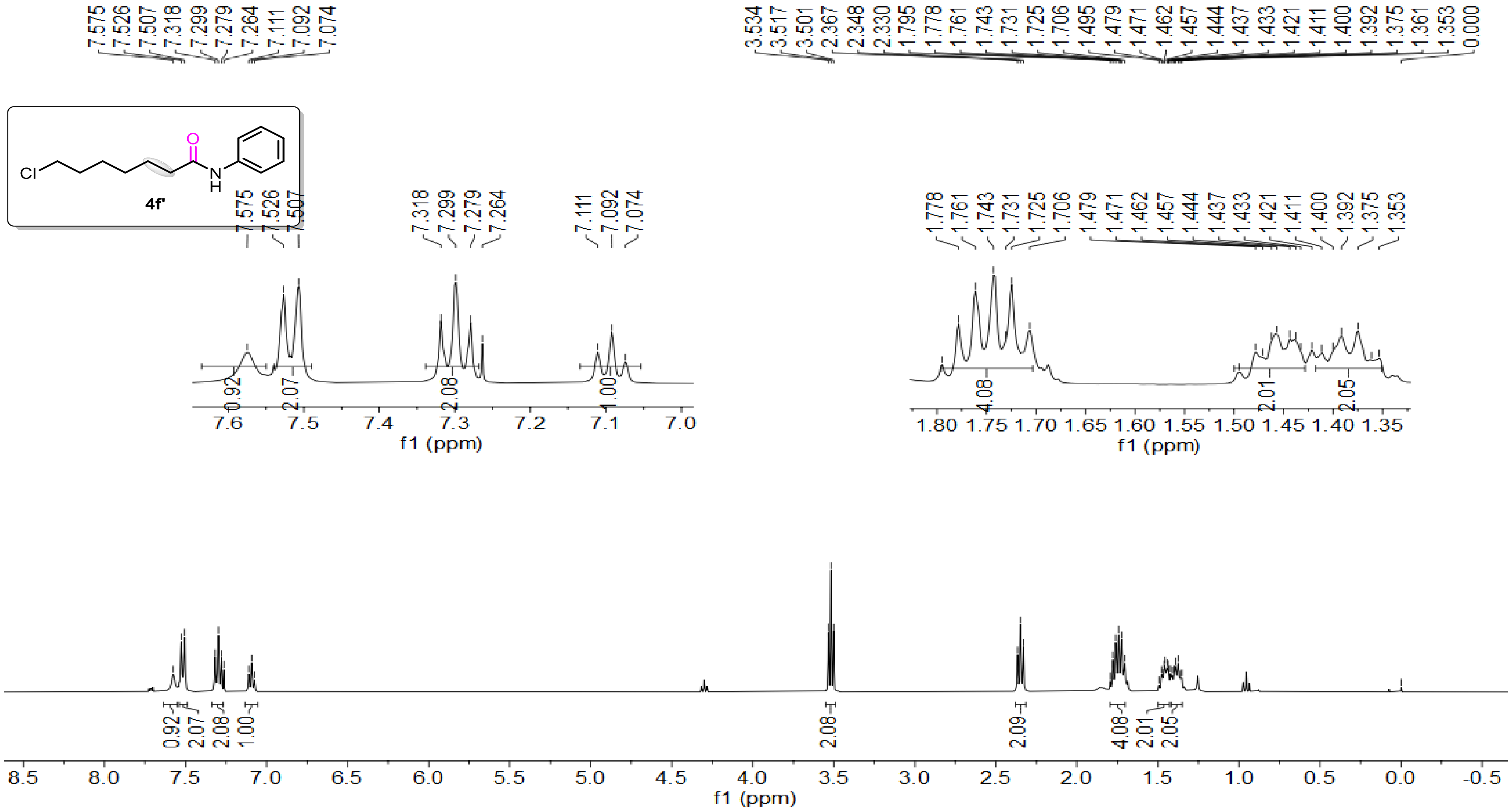

Supplementary Fig. 101. ^{13}C NMR Spectra (101 MHz, $CDCl_3$) of **4f'**

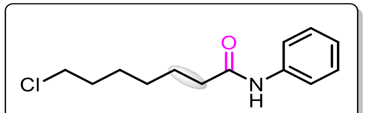

4f'

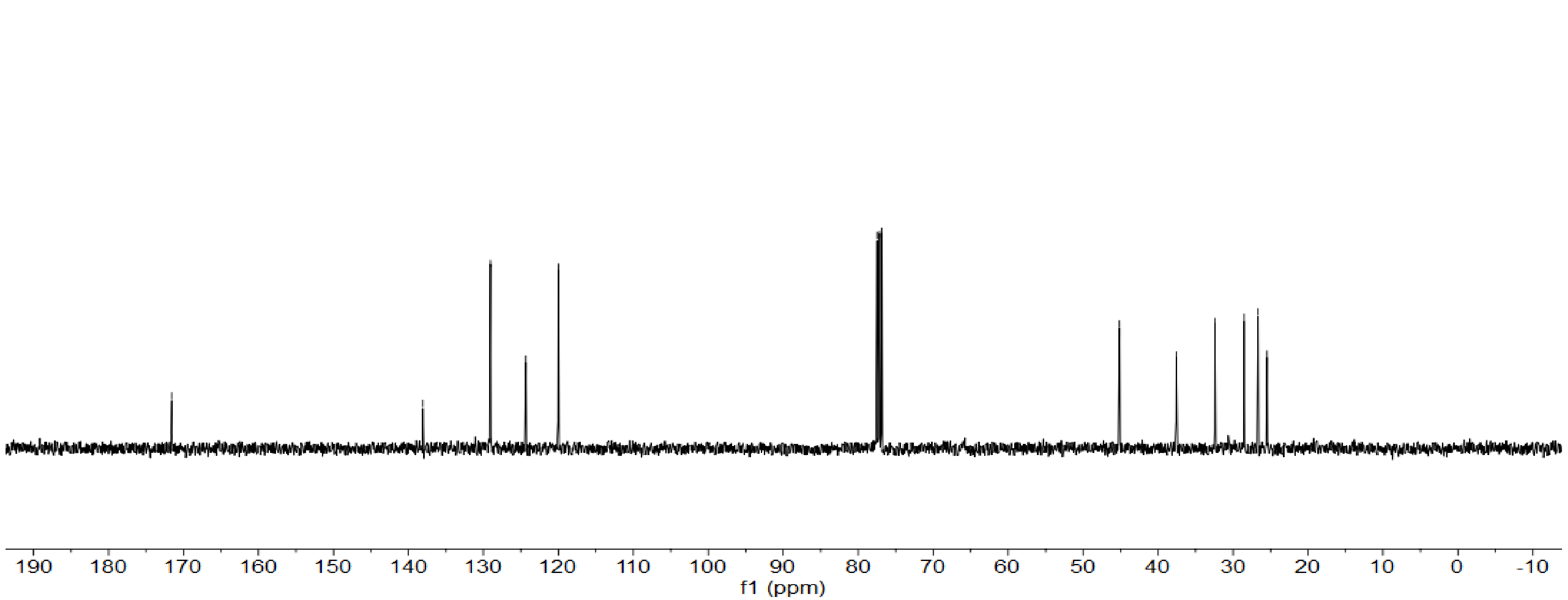

Supplementary Fig. 102. ^{1}H NMR Spectra (400 MHz, $CDCl_3$) of **4g'**

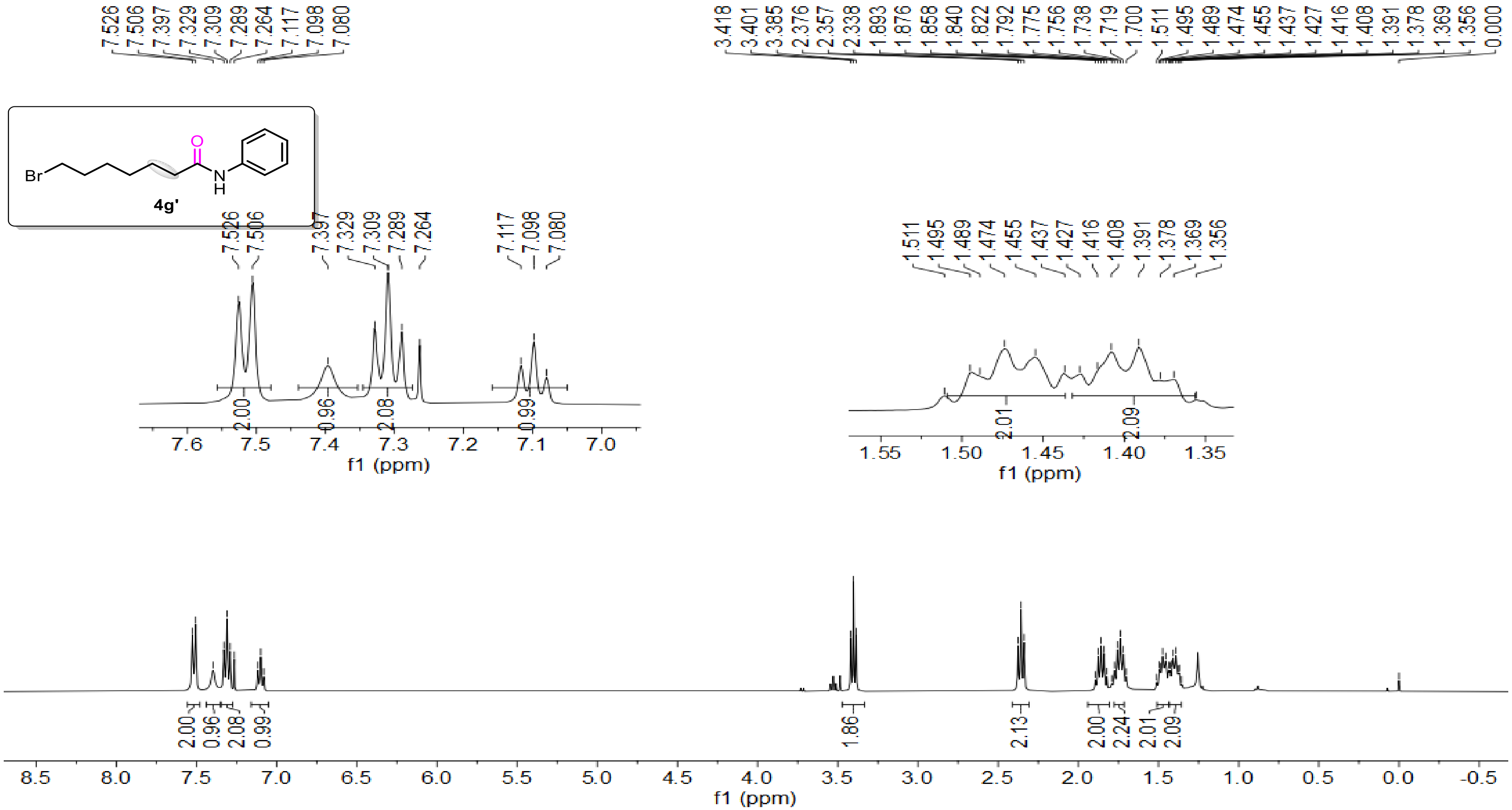

Supplementary Fig. 103. ^{13}C NMR Spectra (101 MHz, $CDCl_3$) of **4g'**

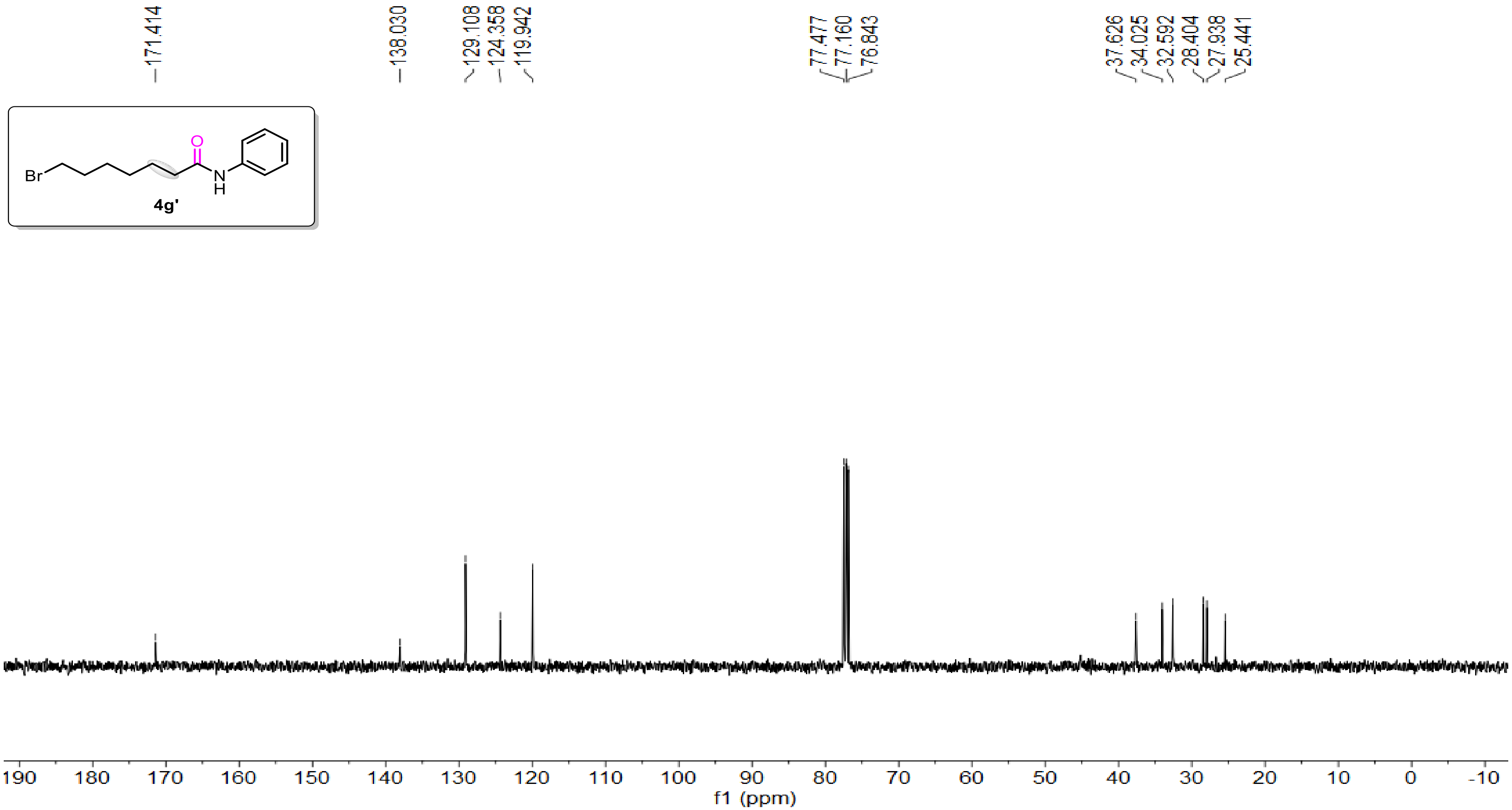

Supplementary Fig. 104. ^{1}H NMR Spectra (400 MHz, $CDCl_3$) of **4h'**

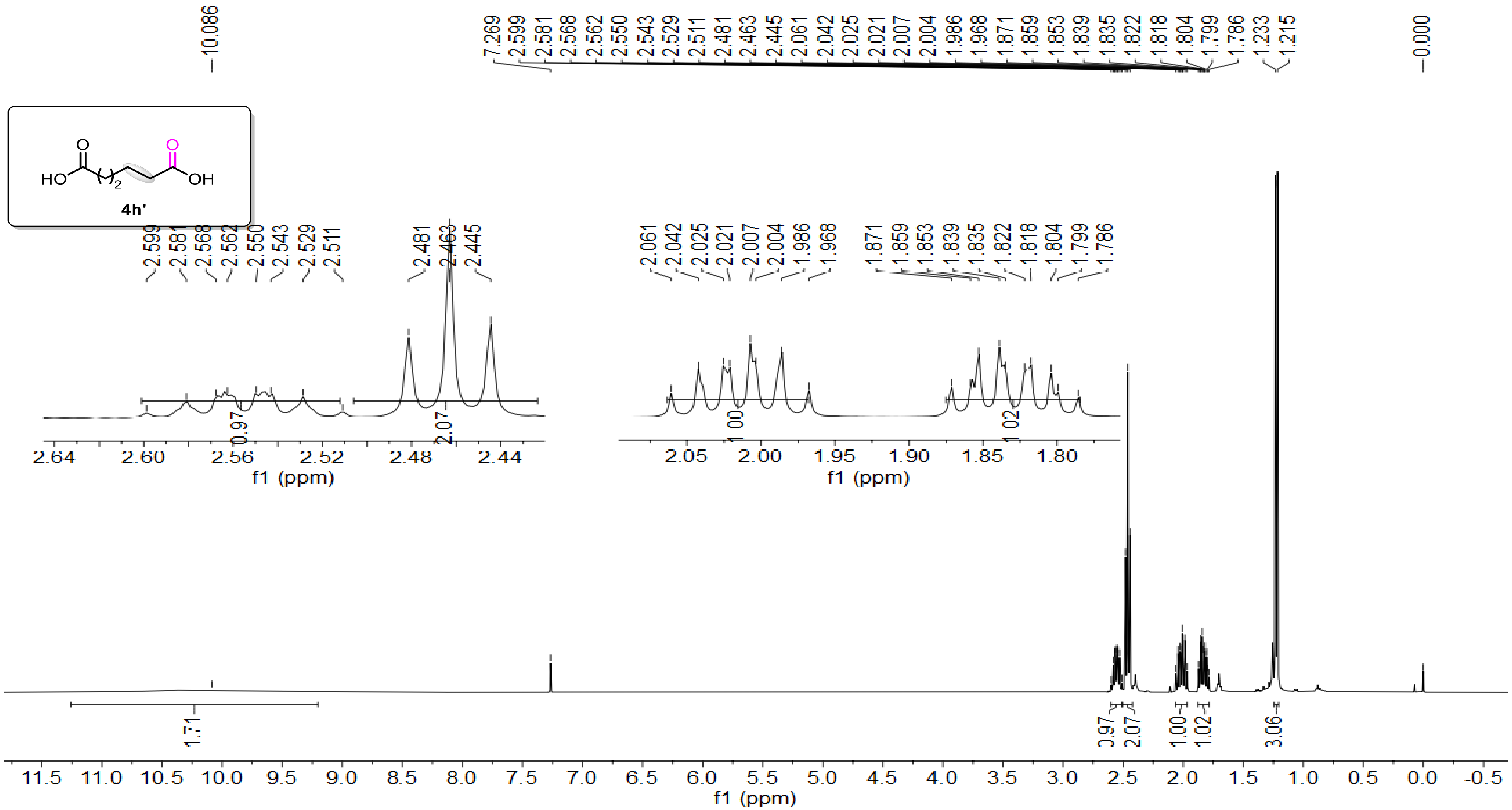

Supplementary Fig. 105. ^{13}C NMR Spectra (101 MHz, $CDCl_3$) of **4h'**

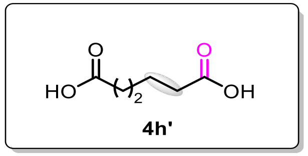

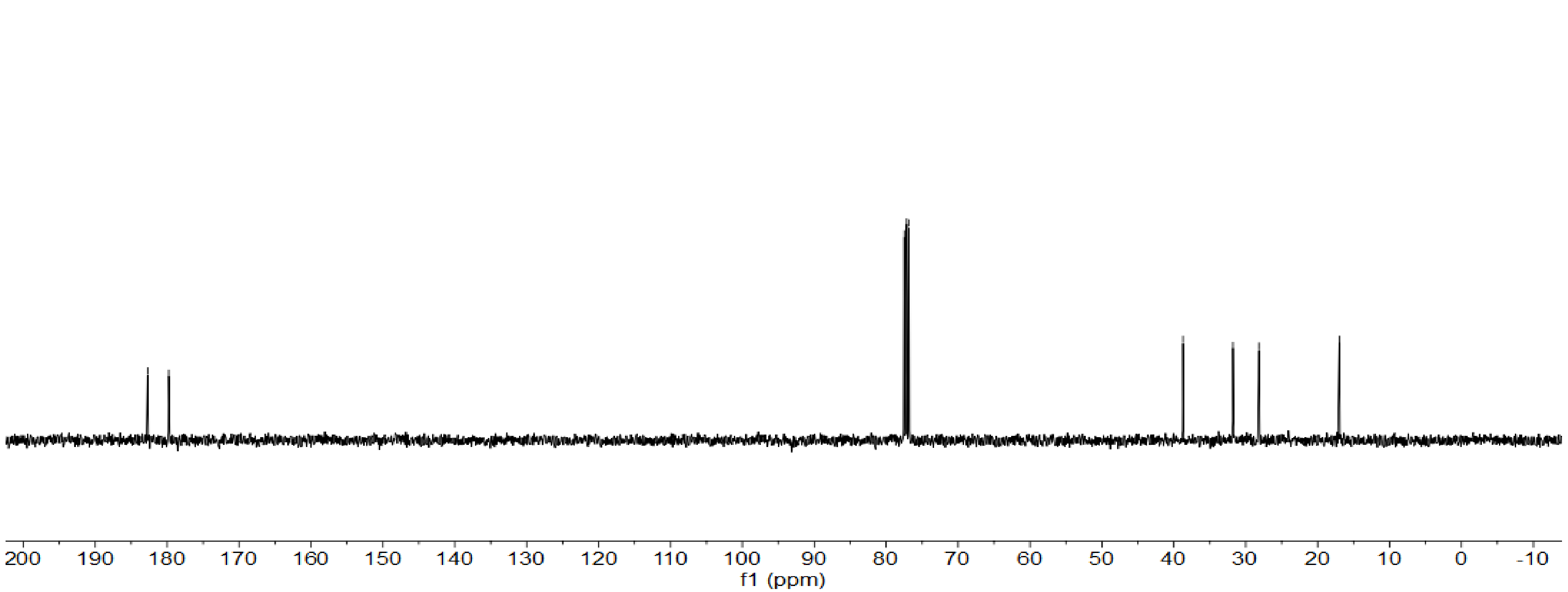

Supplementary Fig. 106. ^{1}H NMR Spectra (400 MHz, $CDCl_3$) of **4i'**

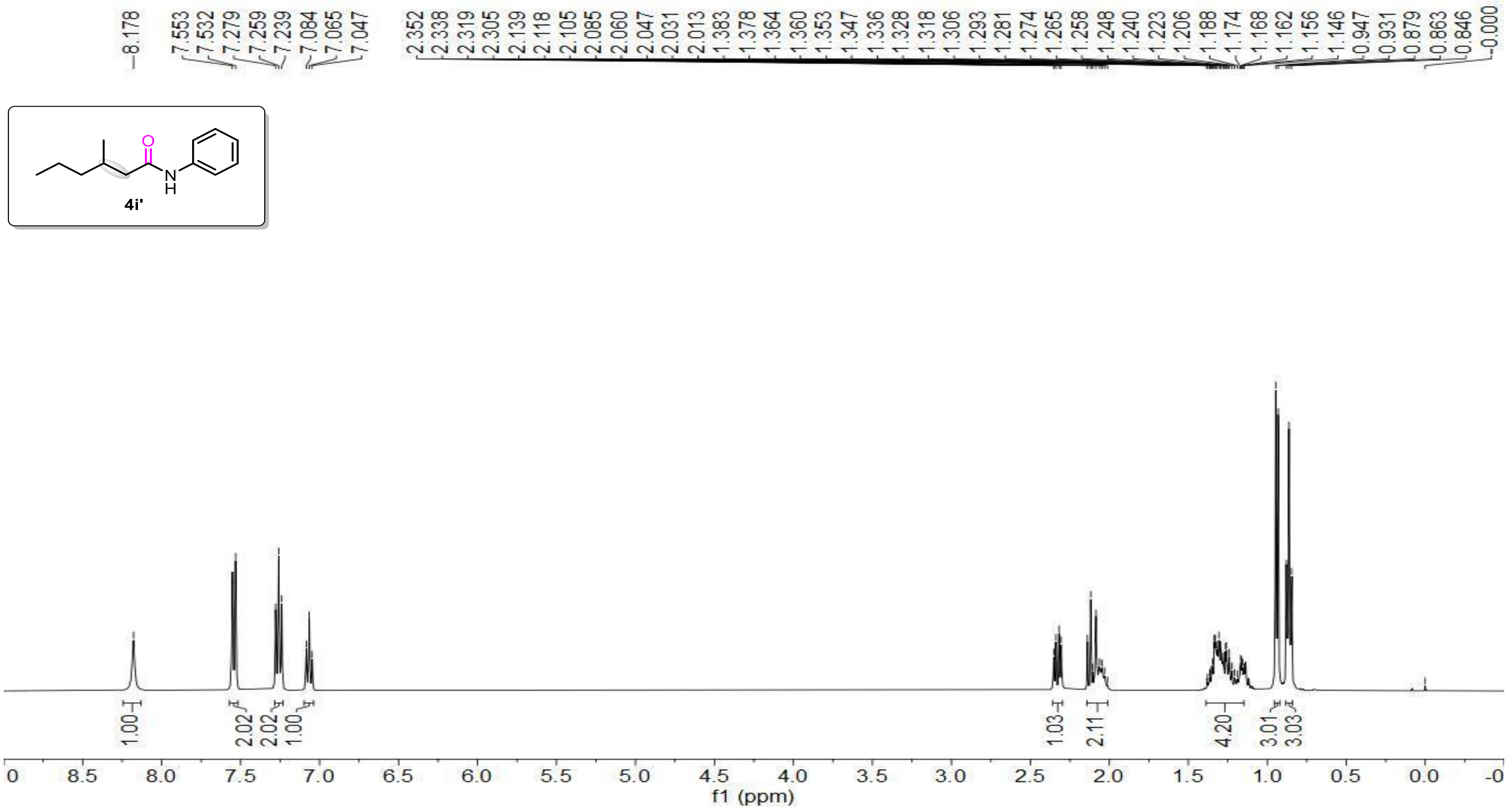

Supplementary Fig. 107. ^{13}C NMR Spectra (101 MHz, $CDCl_3$) of **4i'**

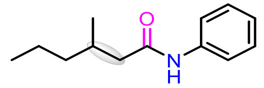

4i'

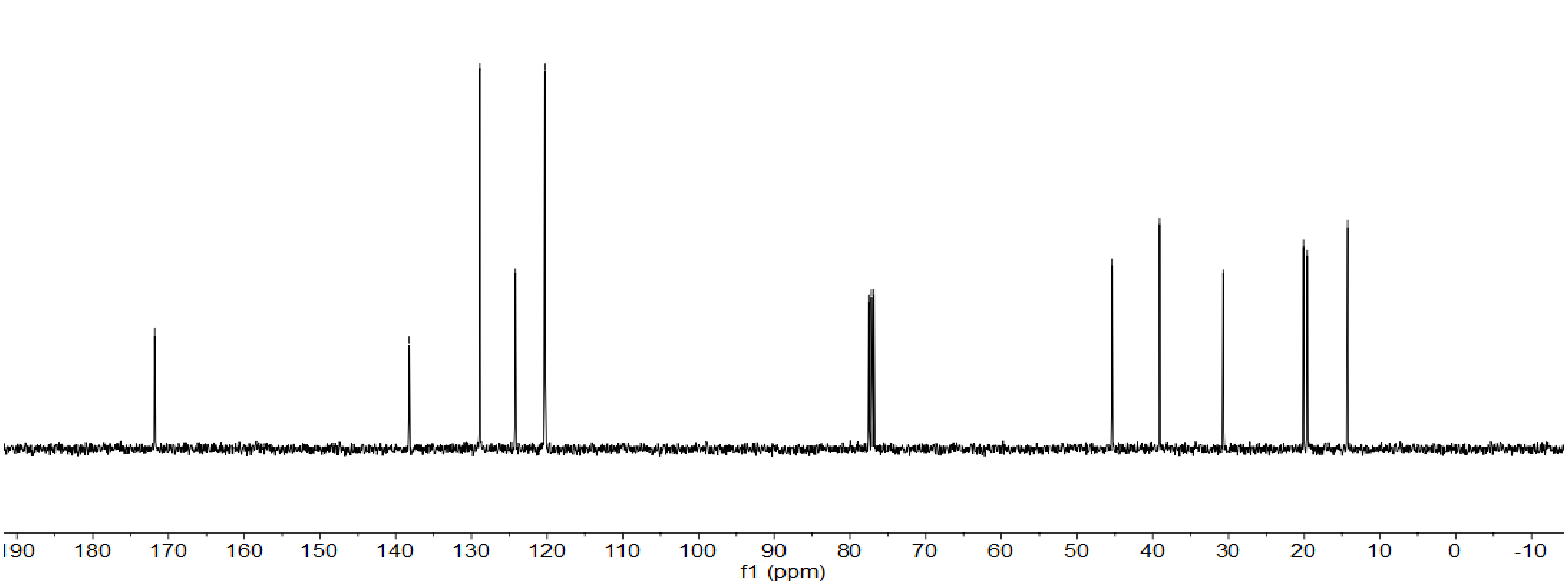

Supplementary Fig. 108. ^{1}H NMR Spectra (400 MHz, $CDCl_3$) of **4j'**

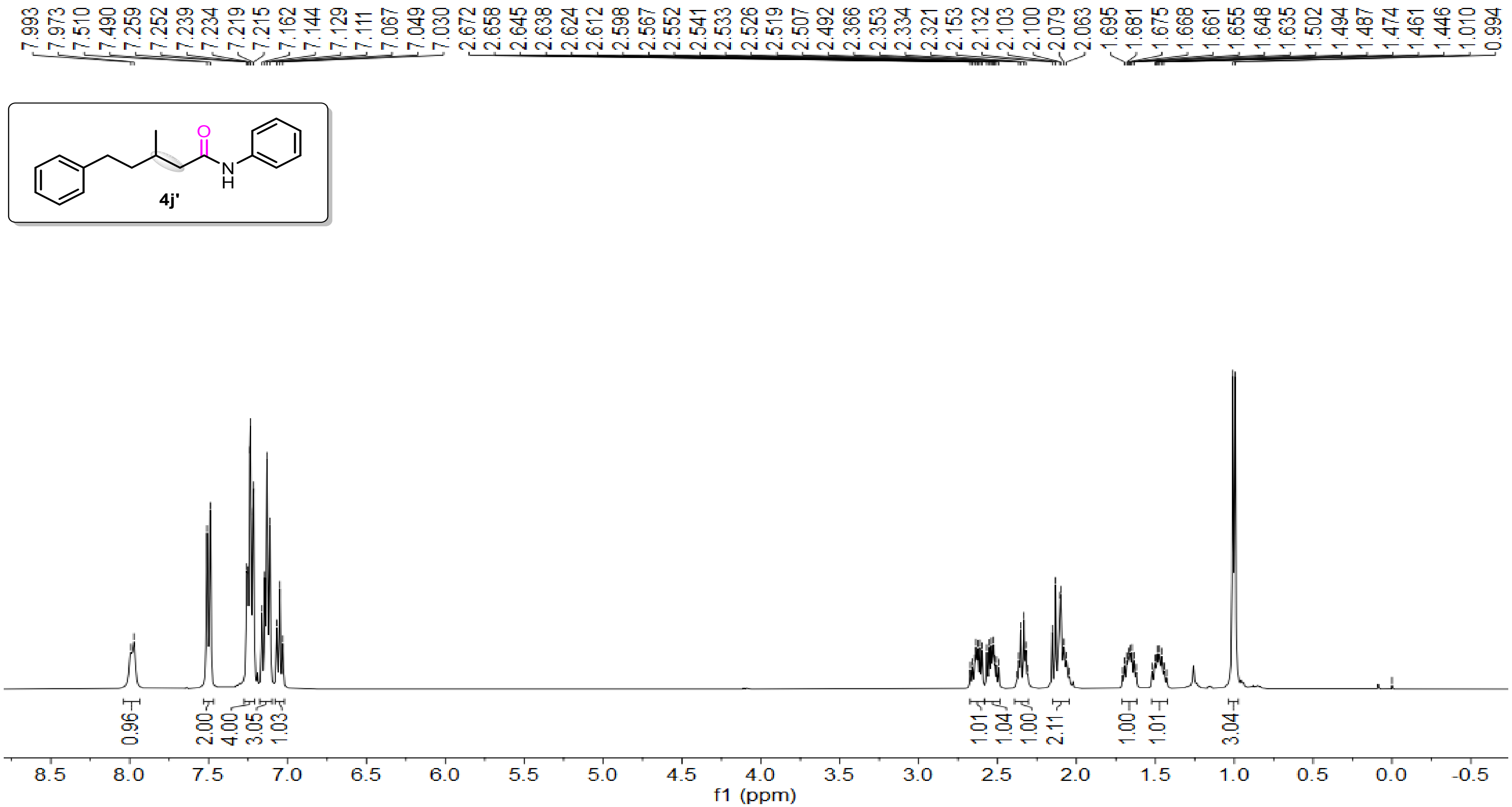

Supplementary Fig. 109. ^{13}C NMR Spectra (101 MHz, $CDCl_3$) of **4j'**

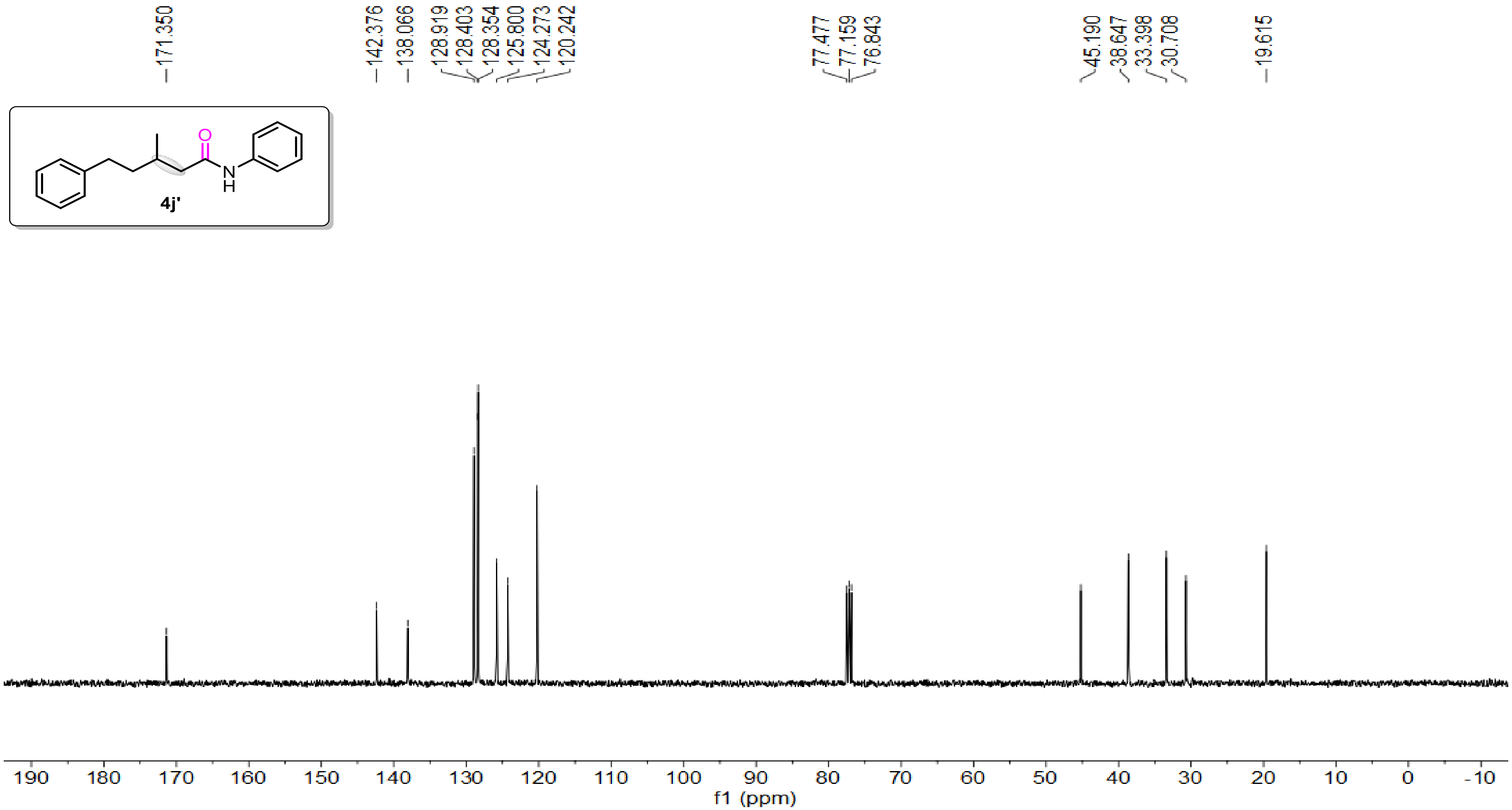

Supplementary Fig. 110. ^{1}H NMR Spectra (400 MHz, $CDCl_3$) of **4k'**

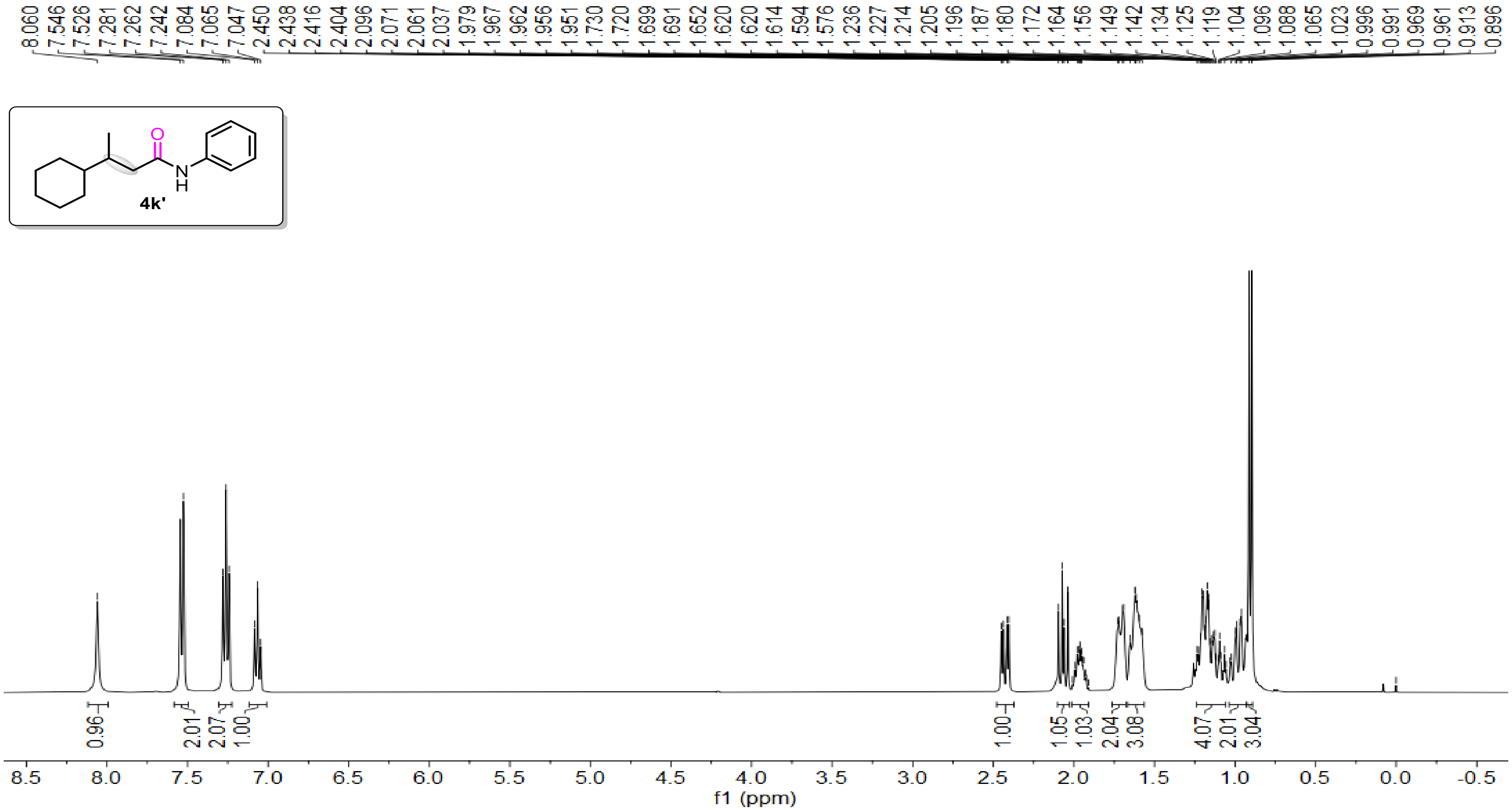

Supplementary Fig. 111. ^{13}C NMR Spectra (101 MHz, $CDCl_3$) of **4k'**

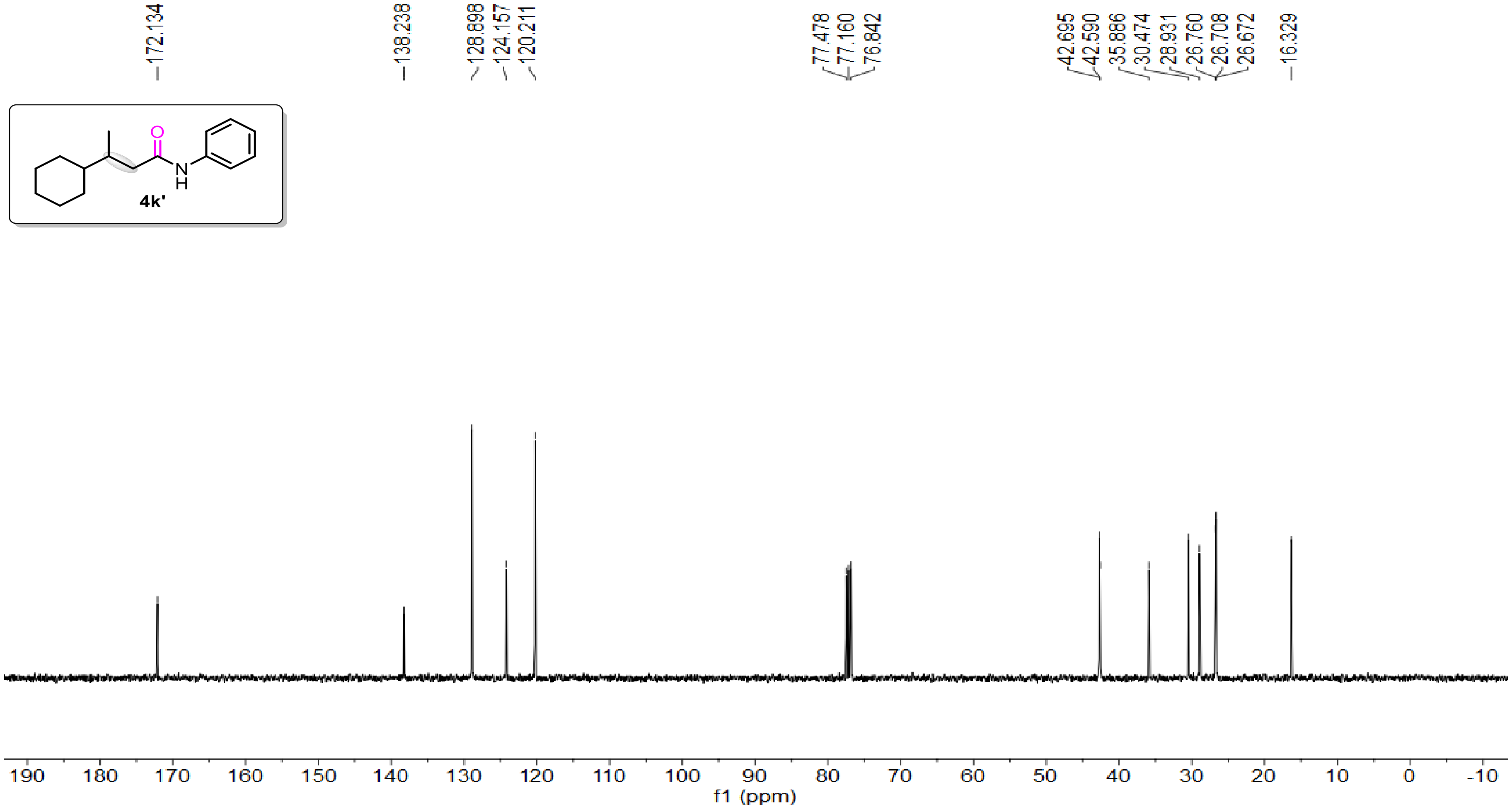

Supplementary Fig. 112. ^{1}H NMR Spectra (400 MHz, $CDCl_3$) of **4l'**

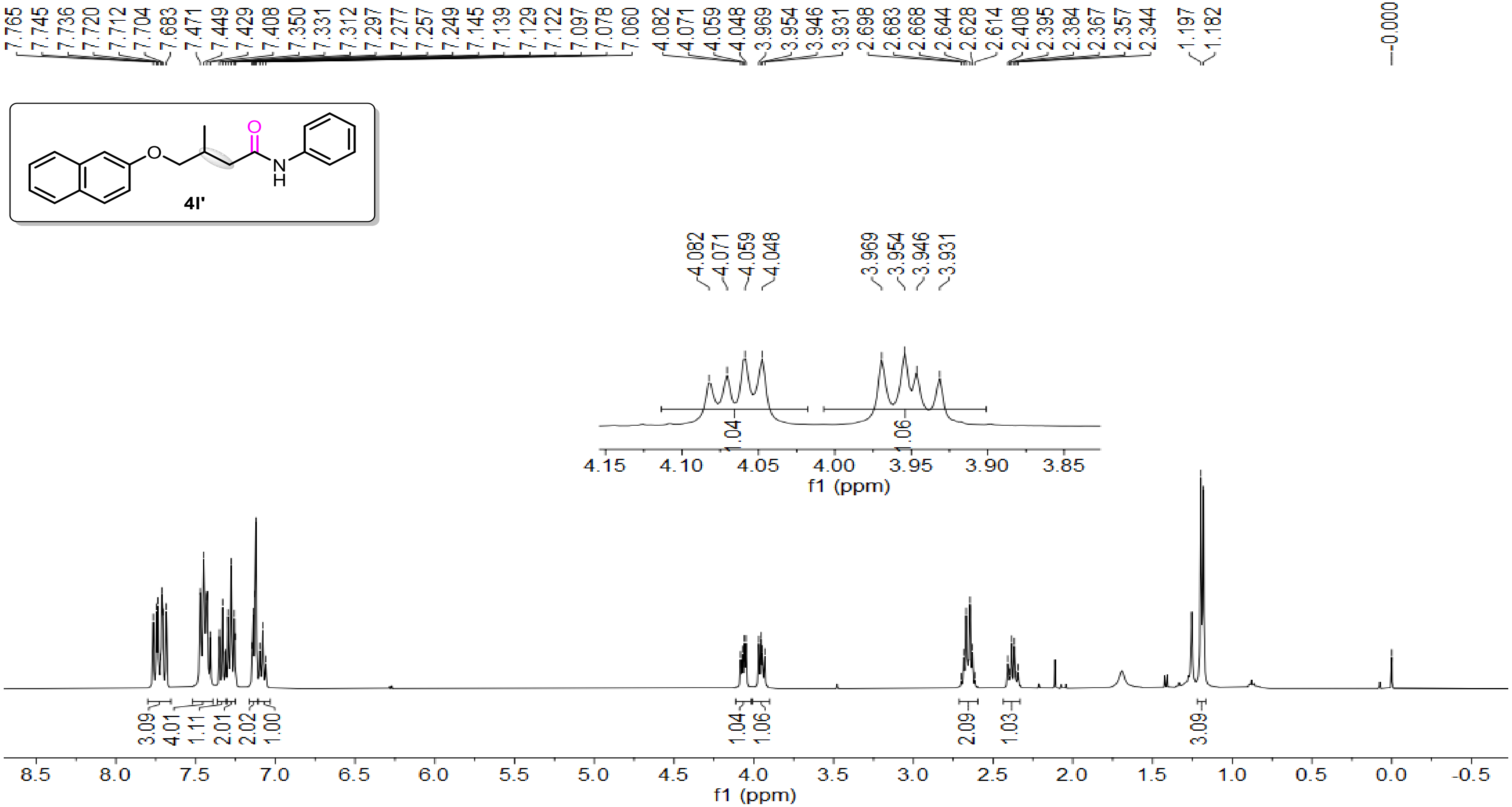

Supplementary Fig. 113. ^{13}C NMR Spectra (101 MHz, $CDCl_3$) of **4l'**

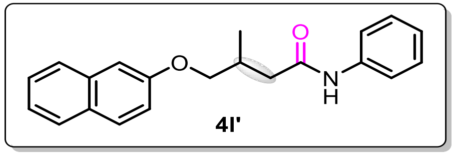

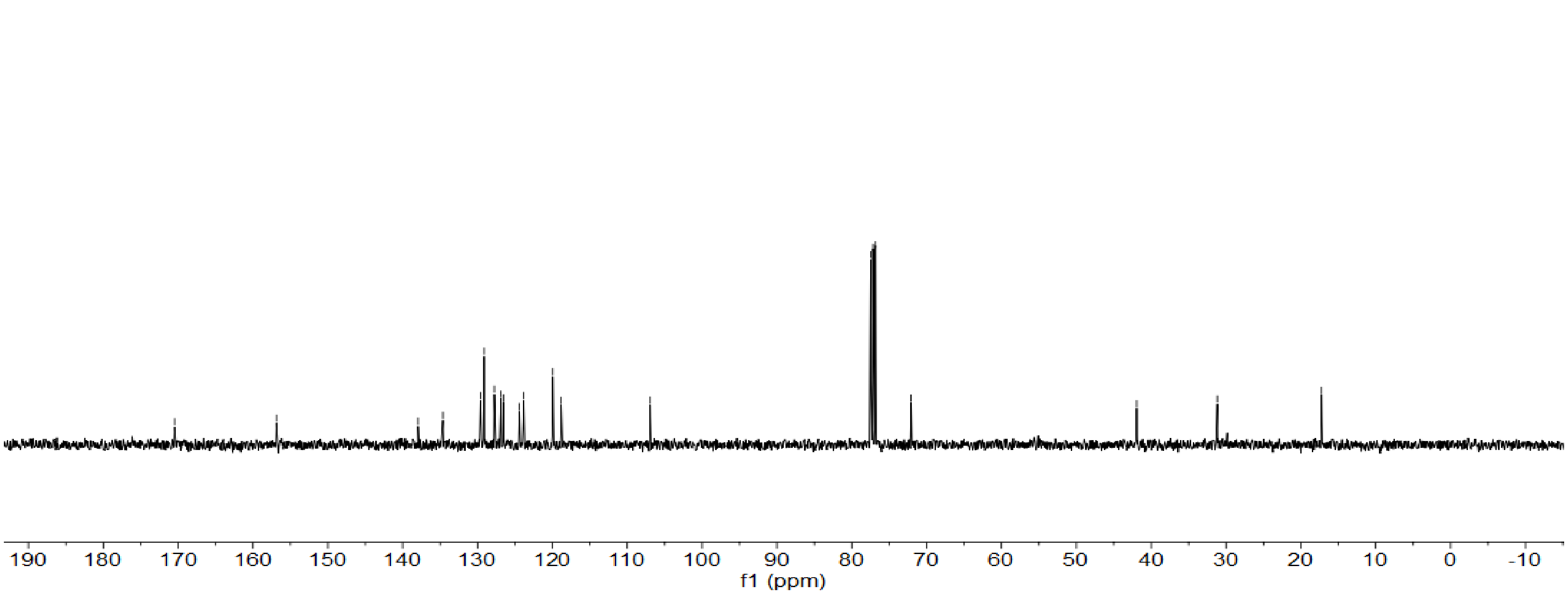

Supplementary Fig. 114. ^{1}H NMR Spectra (400 MHz, $CDCl_3$) of **4m'**

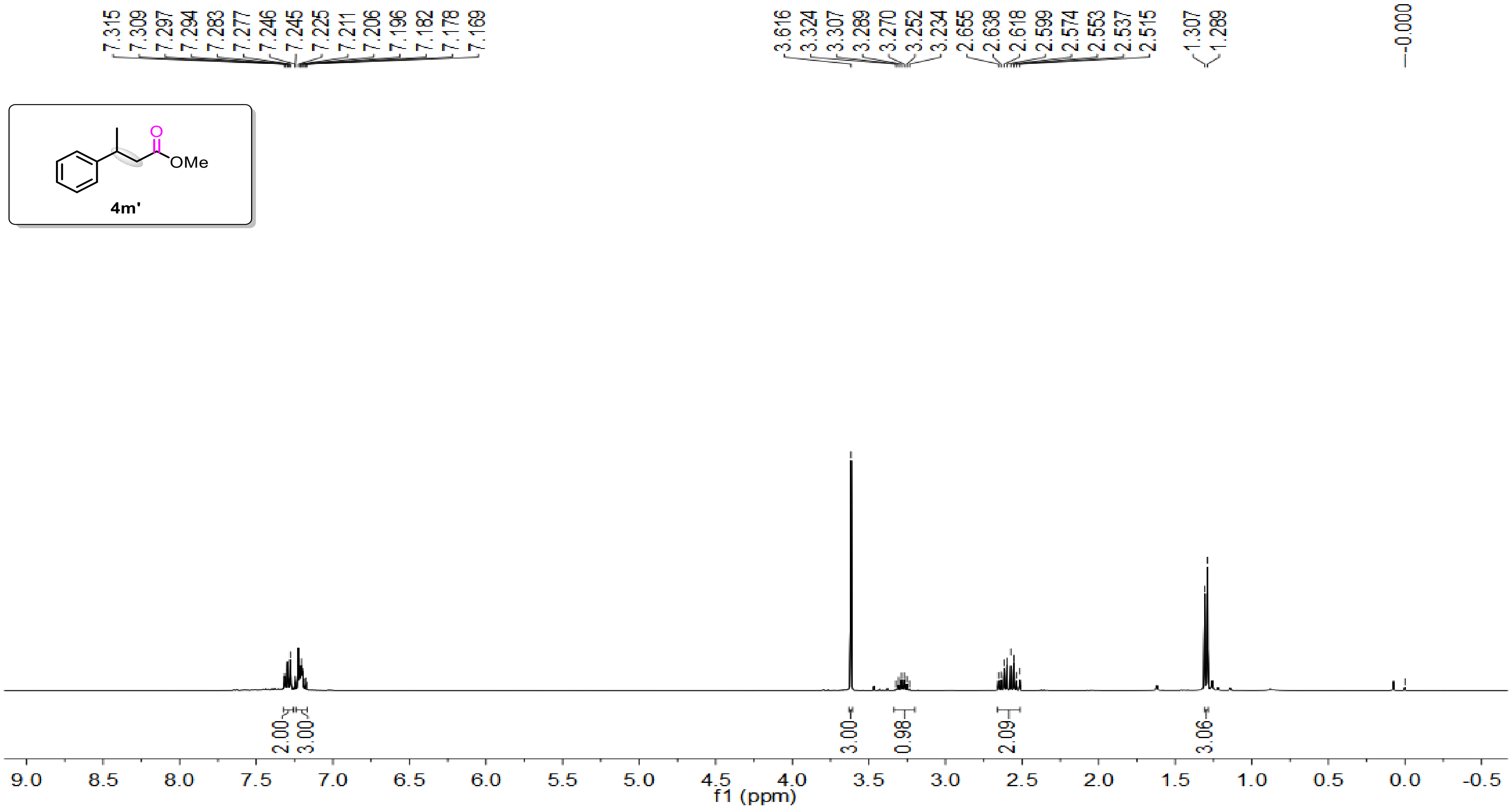

Supplementary Fig. 115. ^{13}C NMR Spectra (101 MHz, $CDCl_3$) of **4m'**

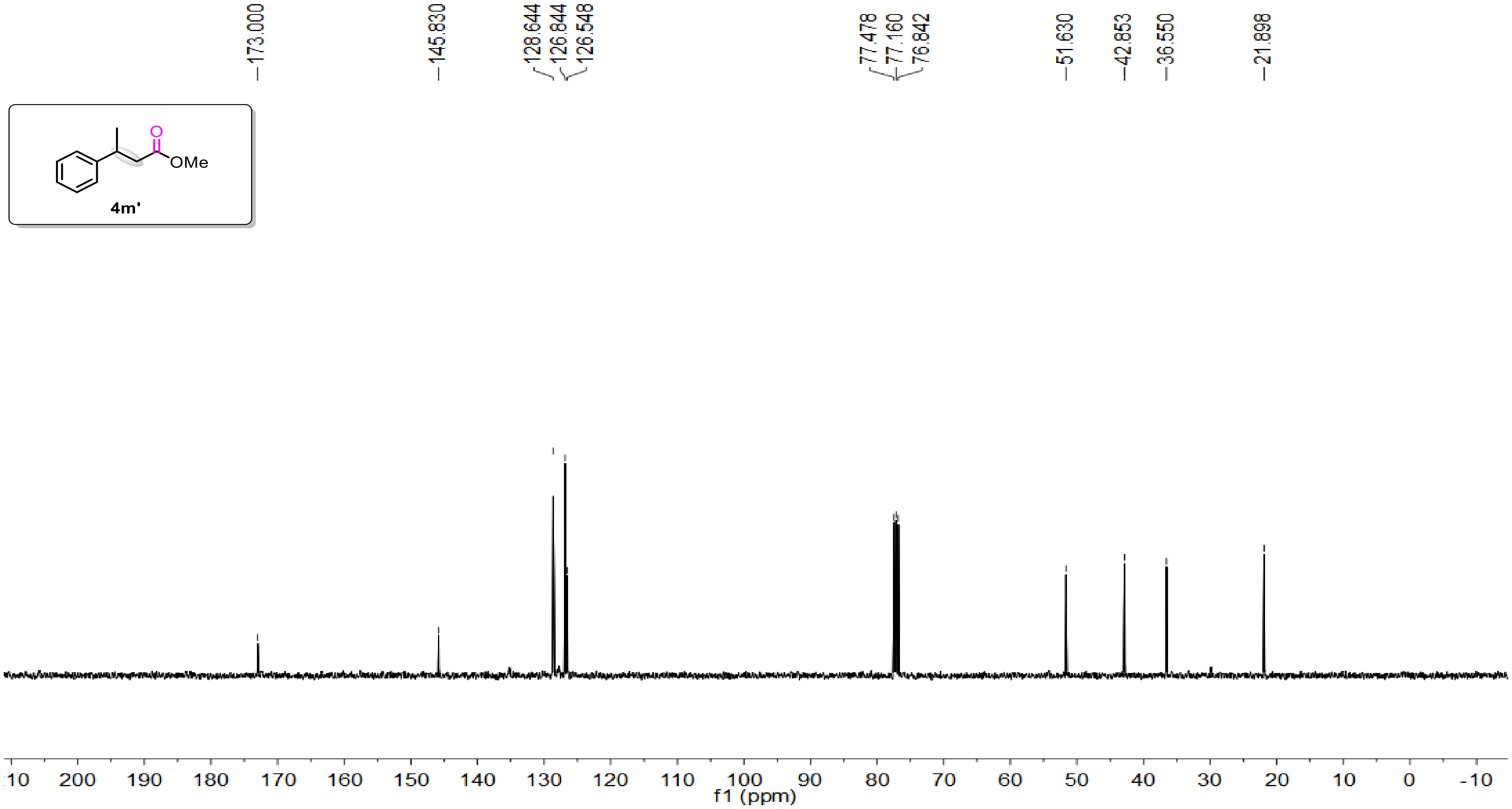

Supplementary Fig. 116. ^{1}H NMR Spectra (400 MHz, $CDCl_3$) of **4n'**

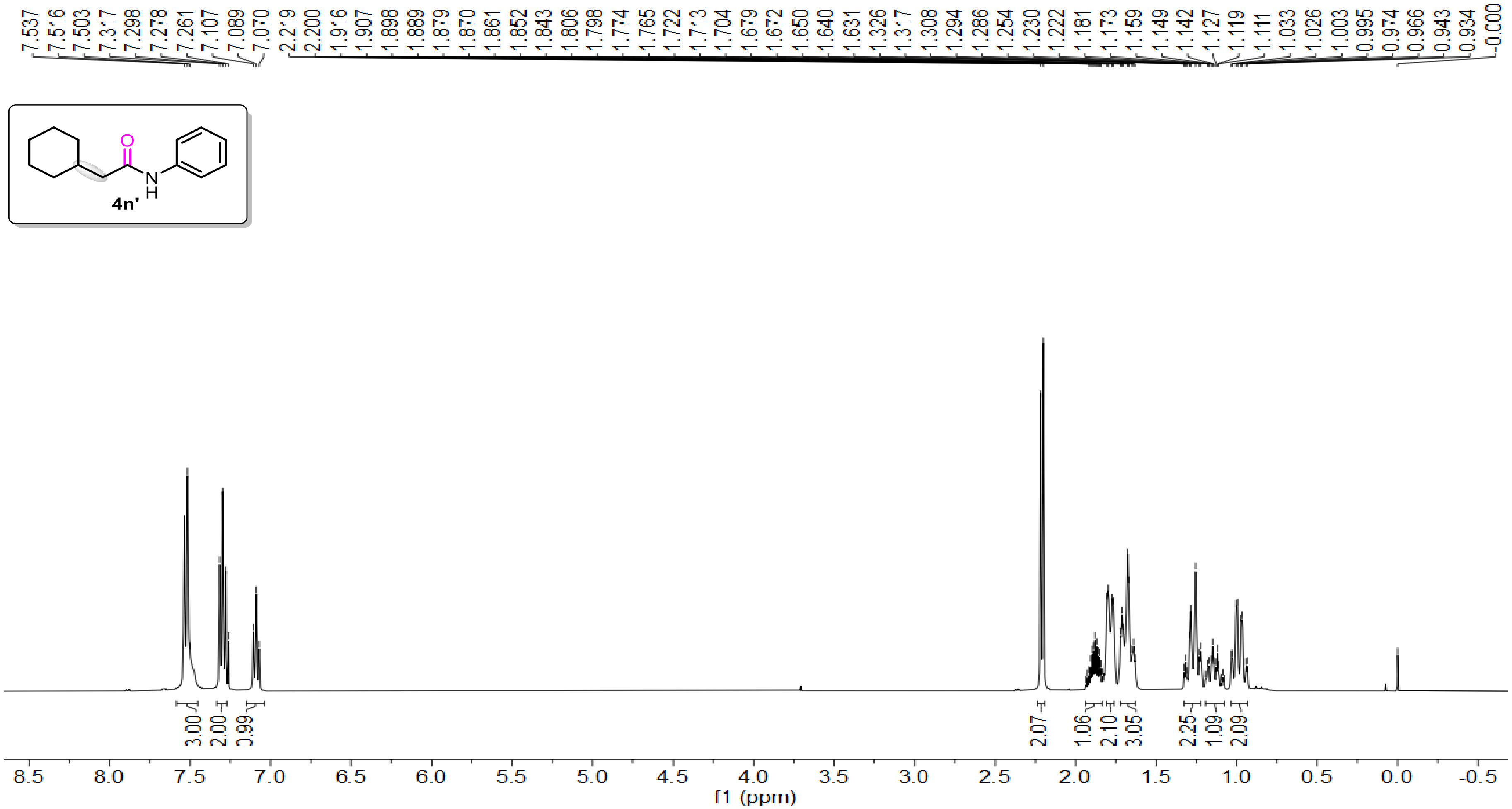

Supplementary Fig. 117. ^{13}C NMR Spectra (101 MHz, $CDCl_3$) of **4n'**

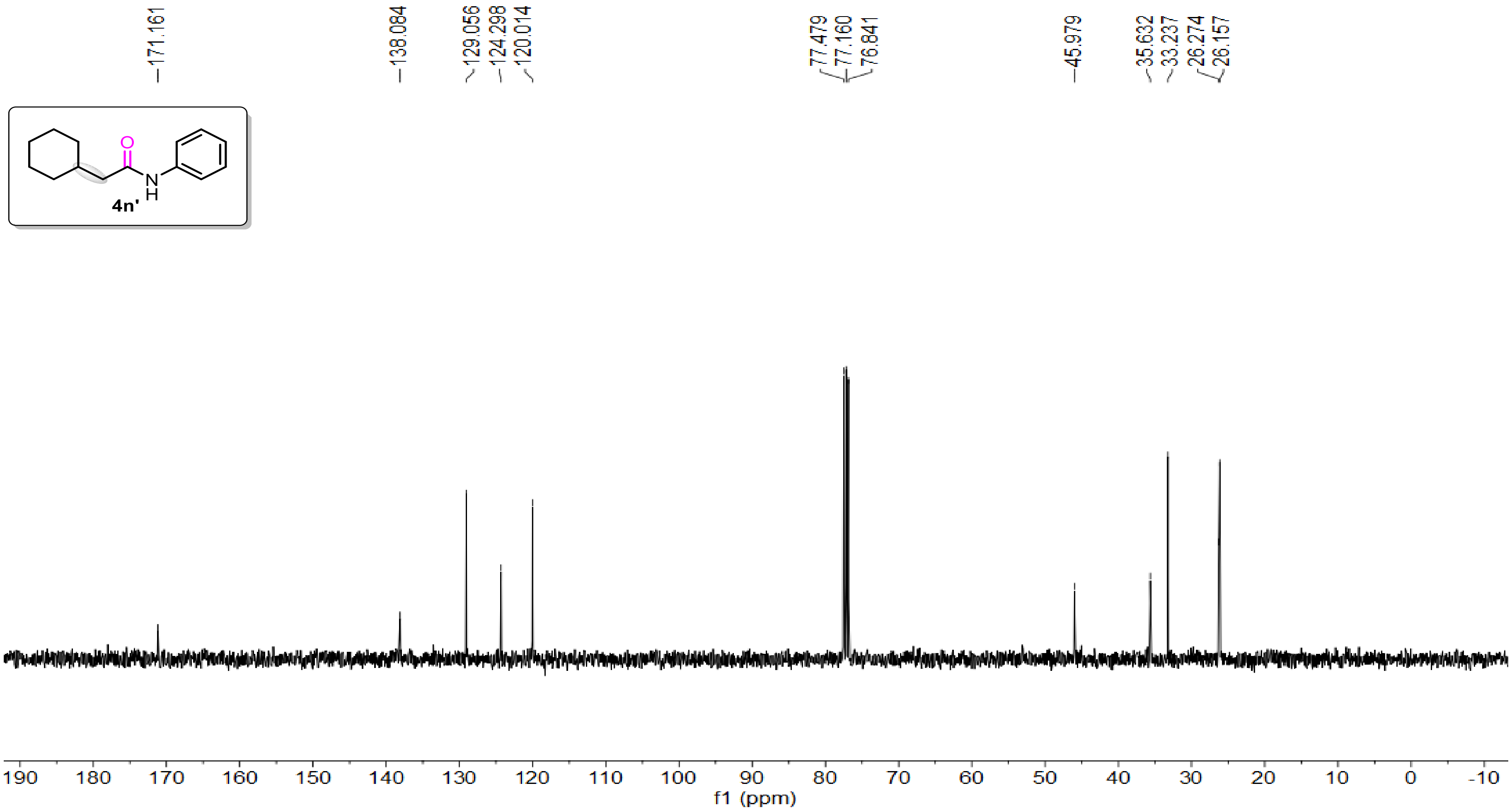

Supplementary Fig. 118. ^{1}H NMR Spectra (400 MHz, $CDCl_3$) of **4o'**

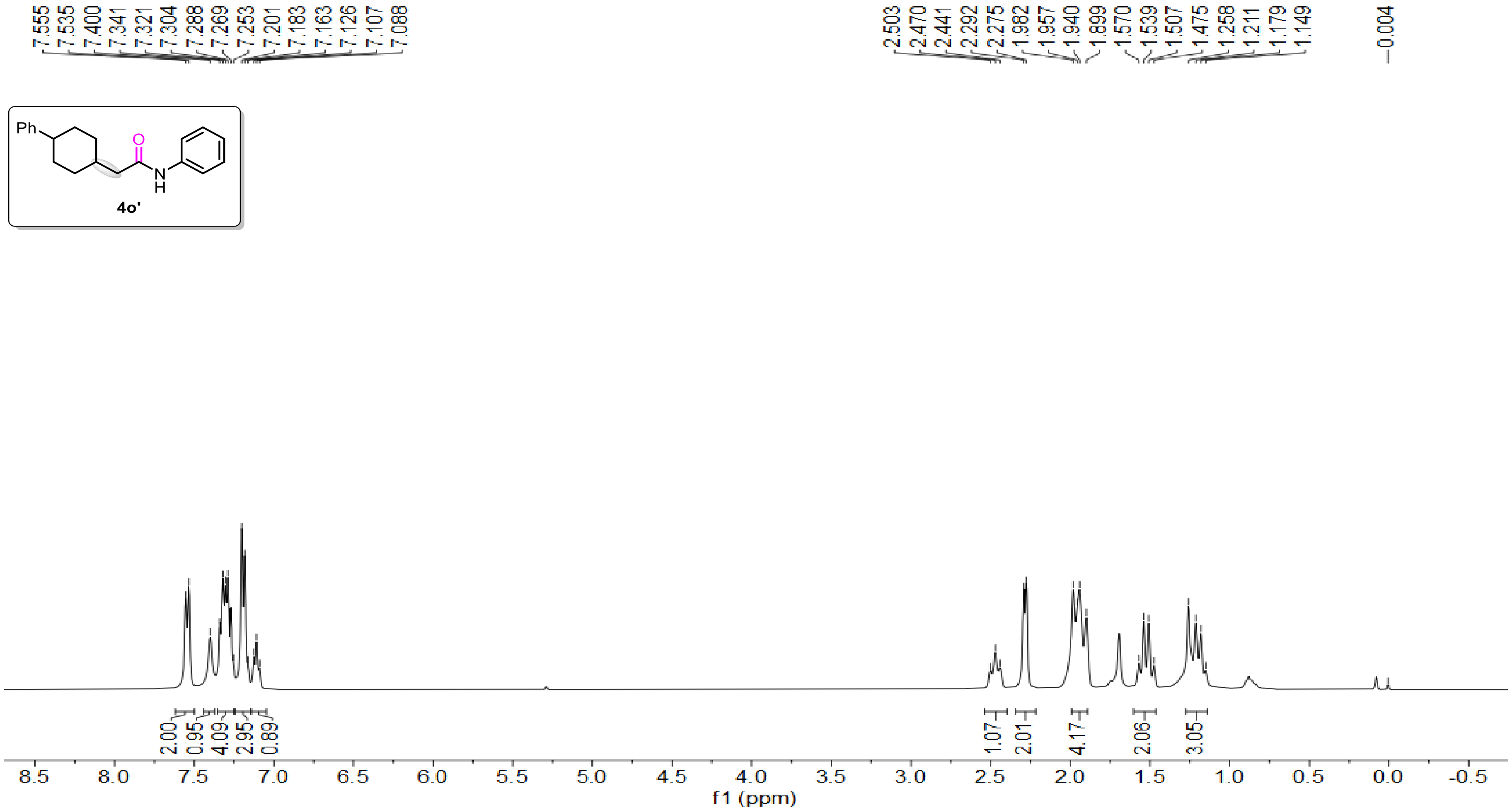

Supplementary Fig. 119. ^{13}C NMR Spectra (101 MHz, $CDCl_3$) of **4o'**

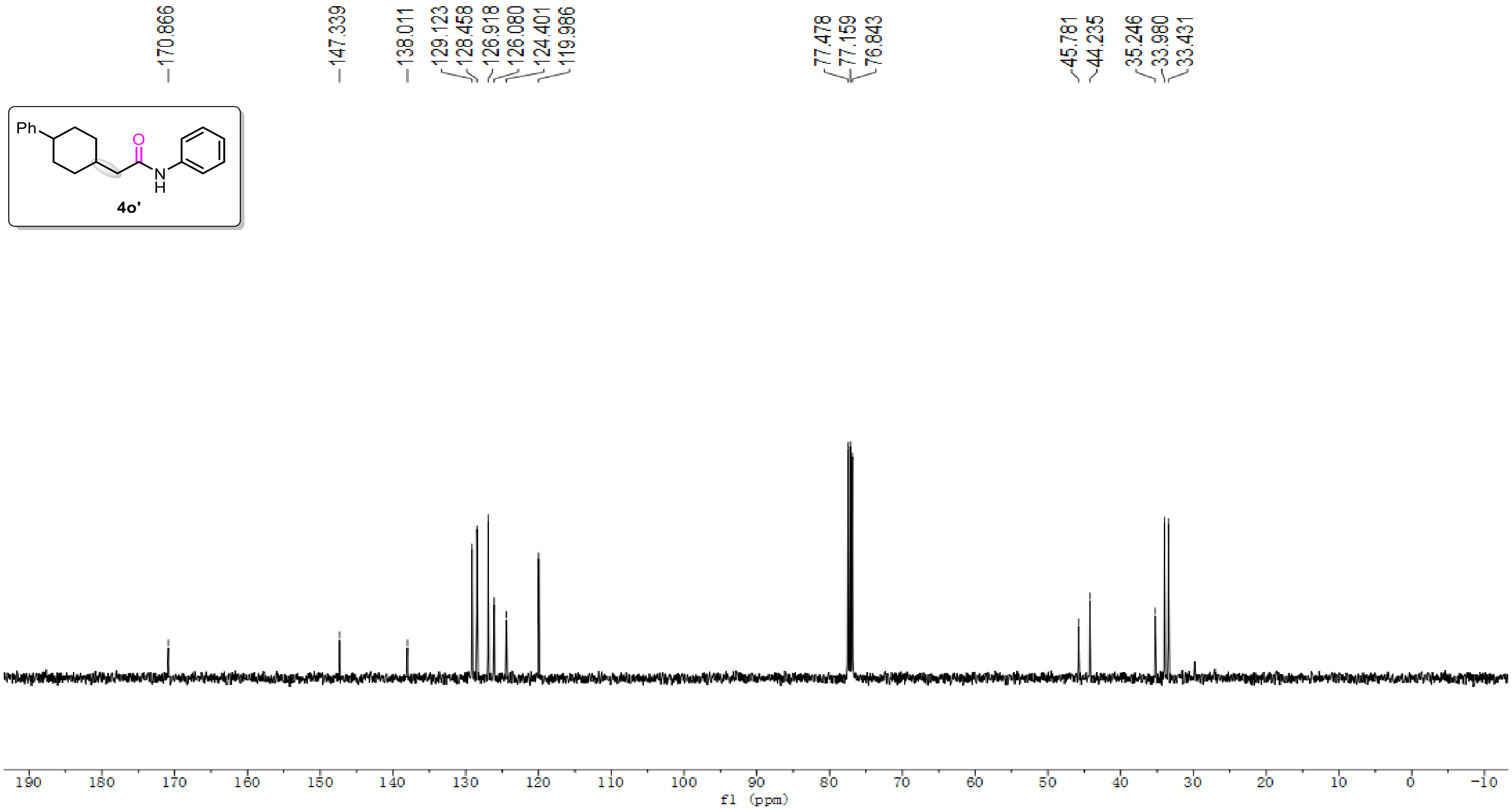

Supplementary Fig. 120. ^{1}H NMR Spectra (400 MHz, $CDCl_3$) of **4p'**

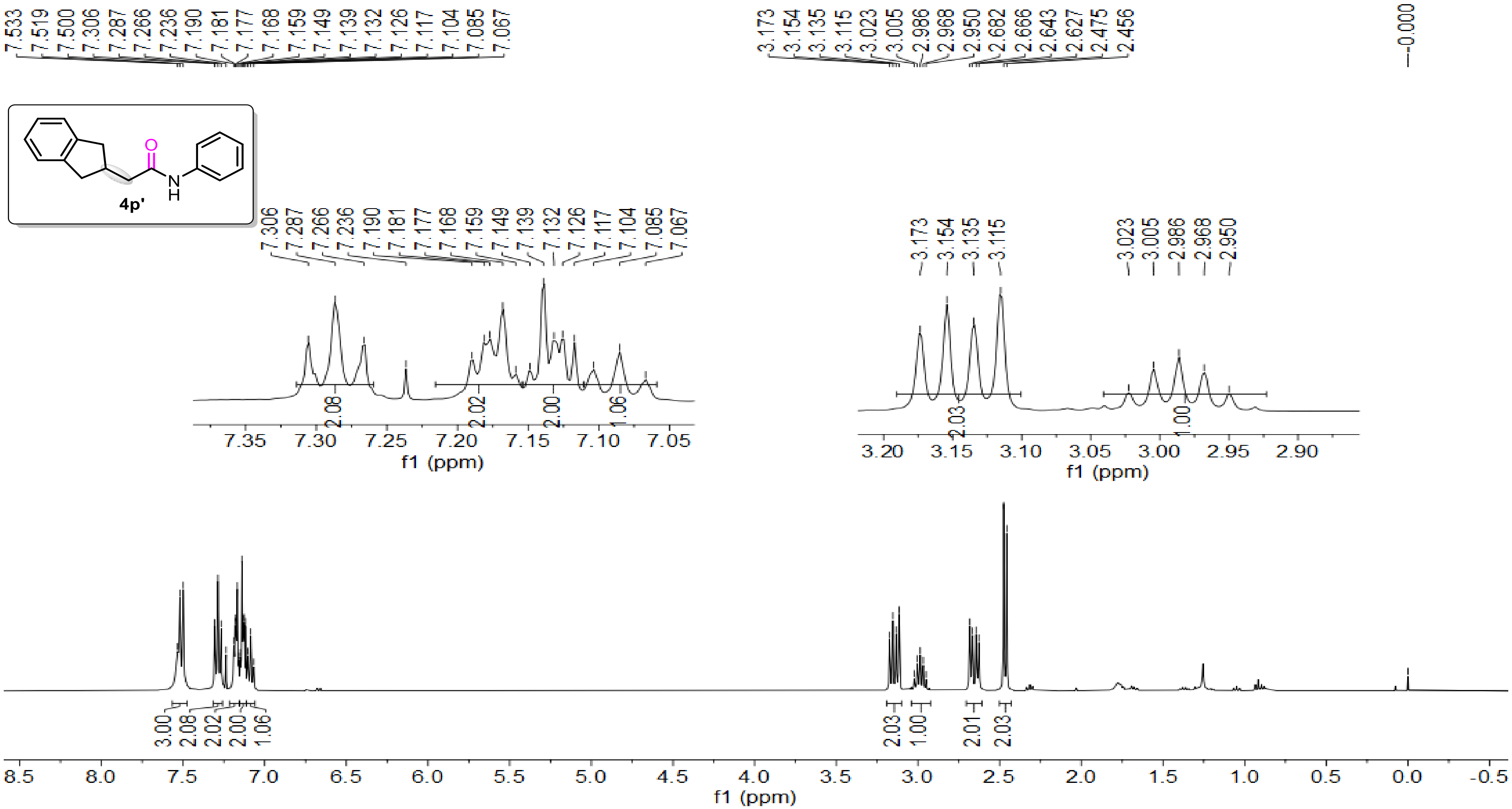

Supplementary Fig. 121. ^{13}C NMR Spectra (101 MHz, $CDCl_3$) of **4p'**

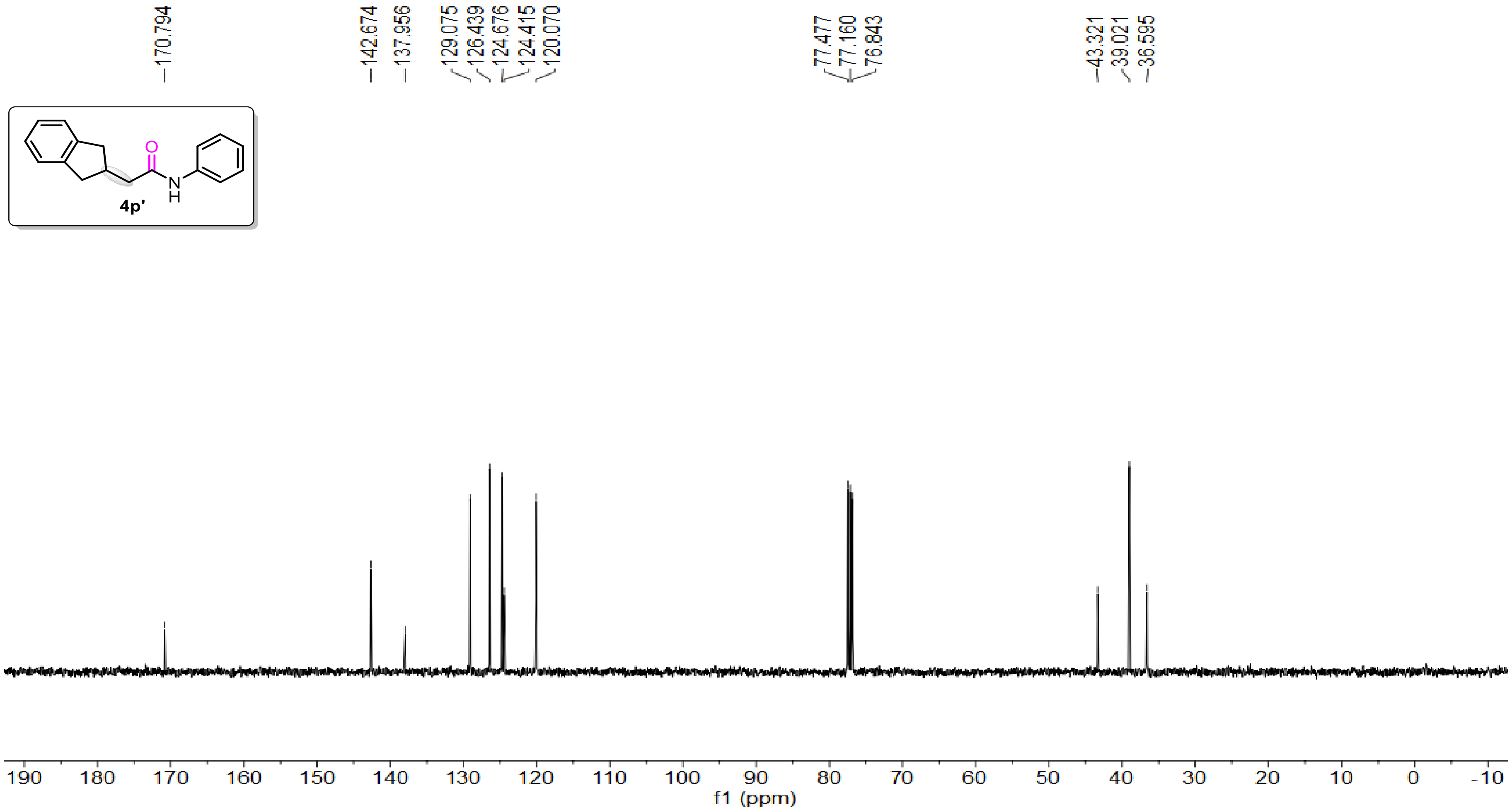

Supplementary Fig. 122. ^{1}H NMR Spectra (400 MHz, $CDCl_3$) of **4q'**

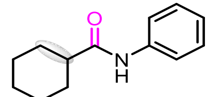

4q'

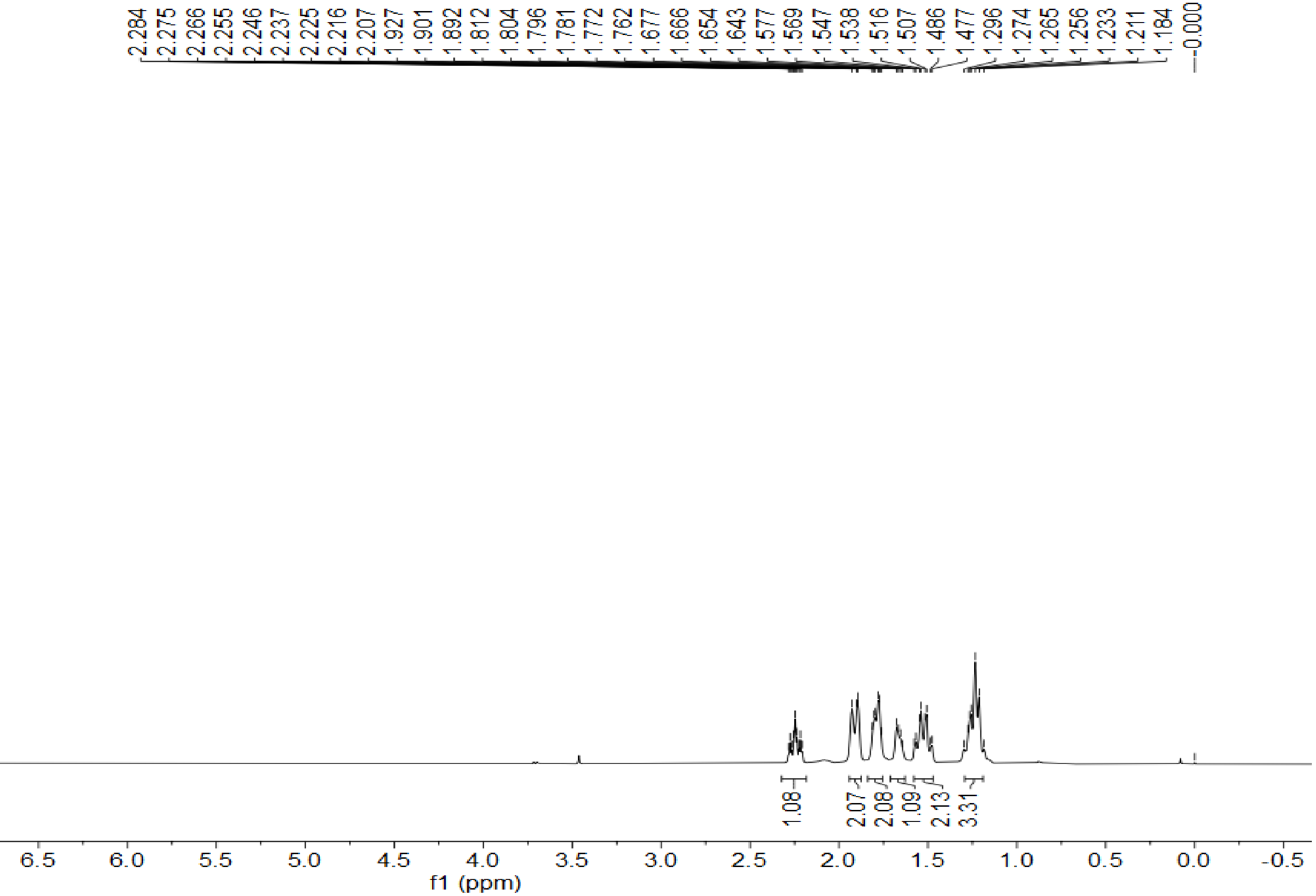

Supplementary Fig. 123. ^{13}C NMR Spectra (101 MHz, $CDCl_3$) of **4q'**

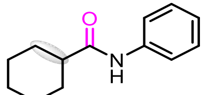

4q'

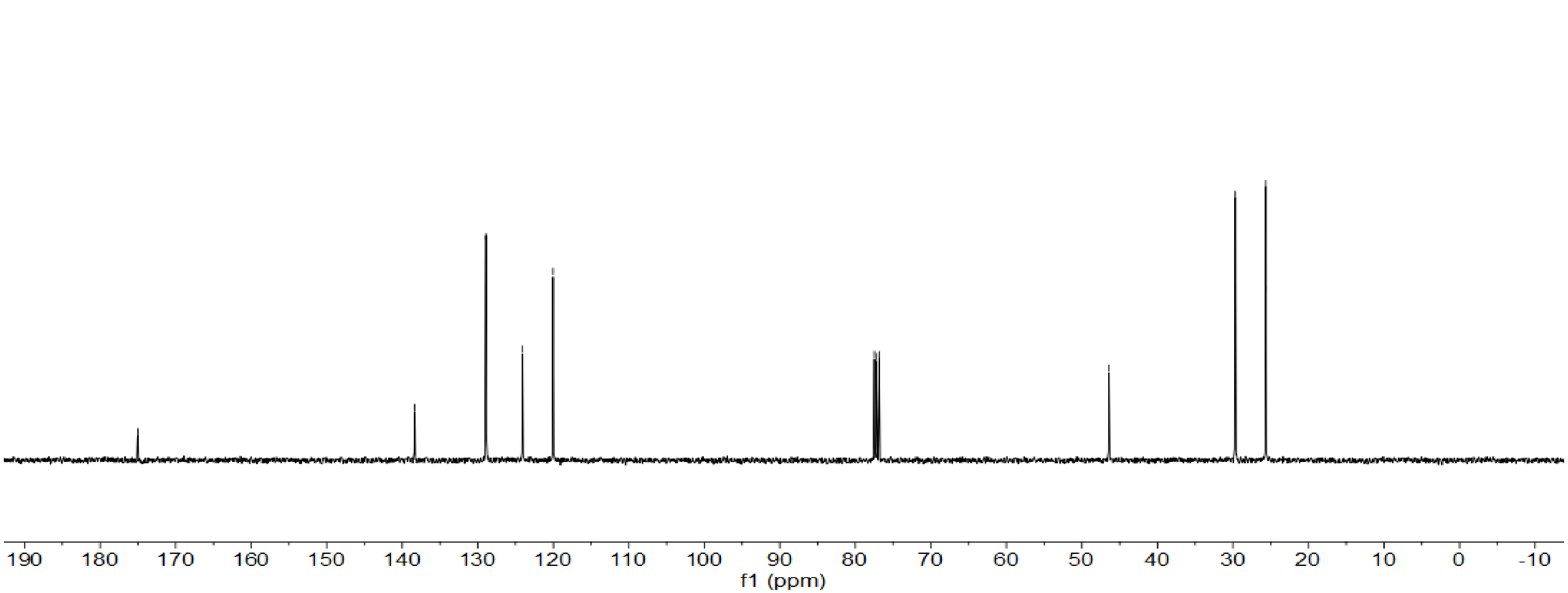

Supplementary Fig. 124. ^{1}H NMR Spectra (400 MHz, $CDCl_3$) of **4r'**

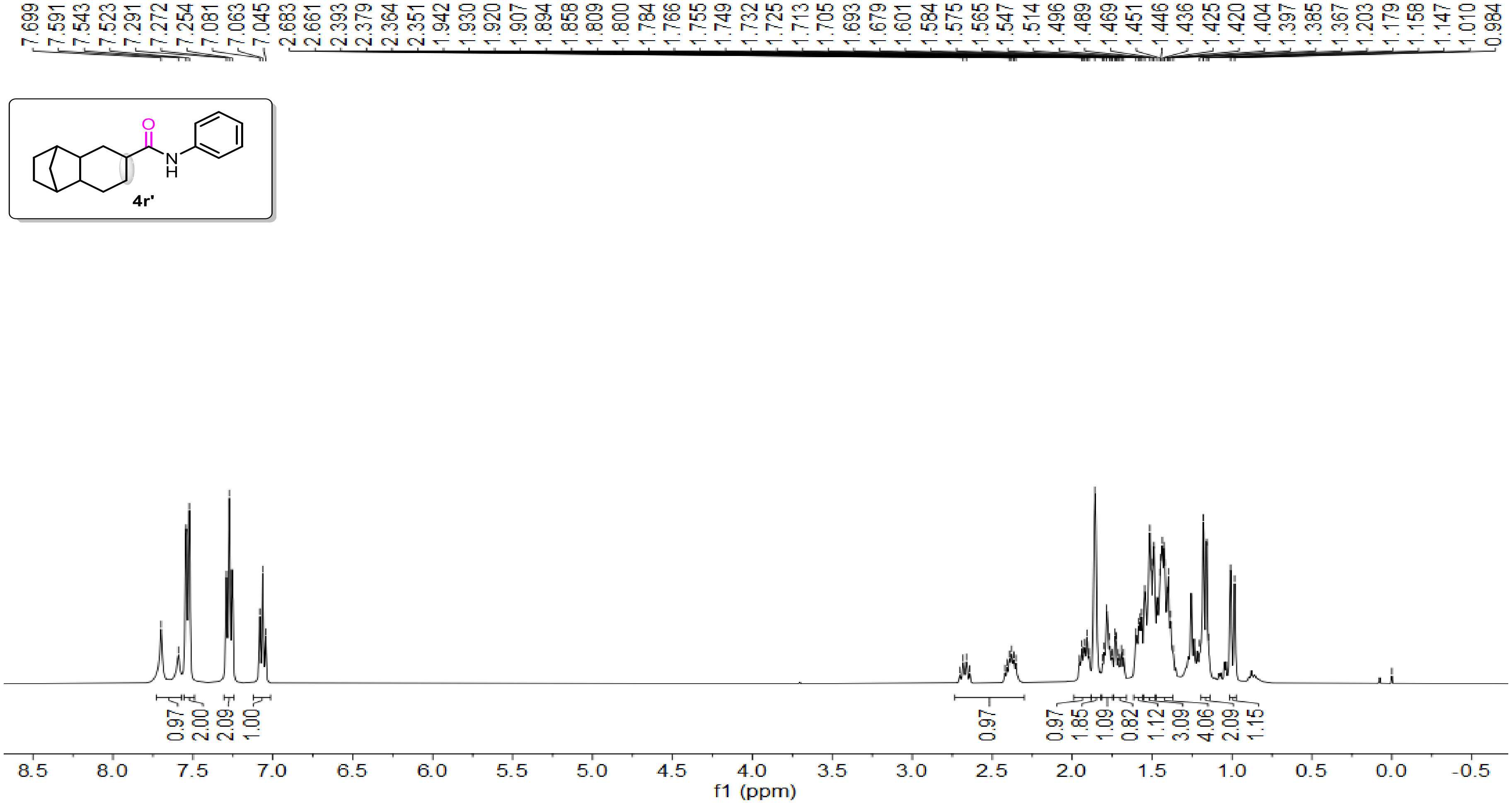

Supplementary Fig. 125. ^{13}C NMR Spectra (101 MHz, $CDCl_3$) of **4r'**

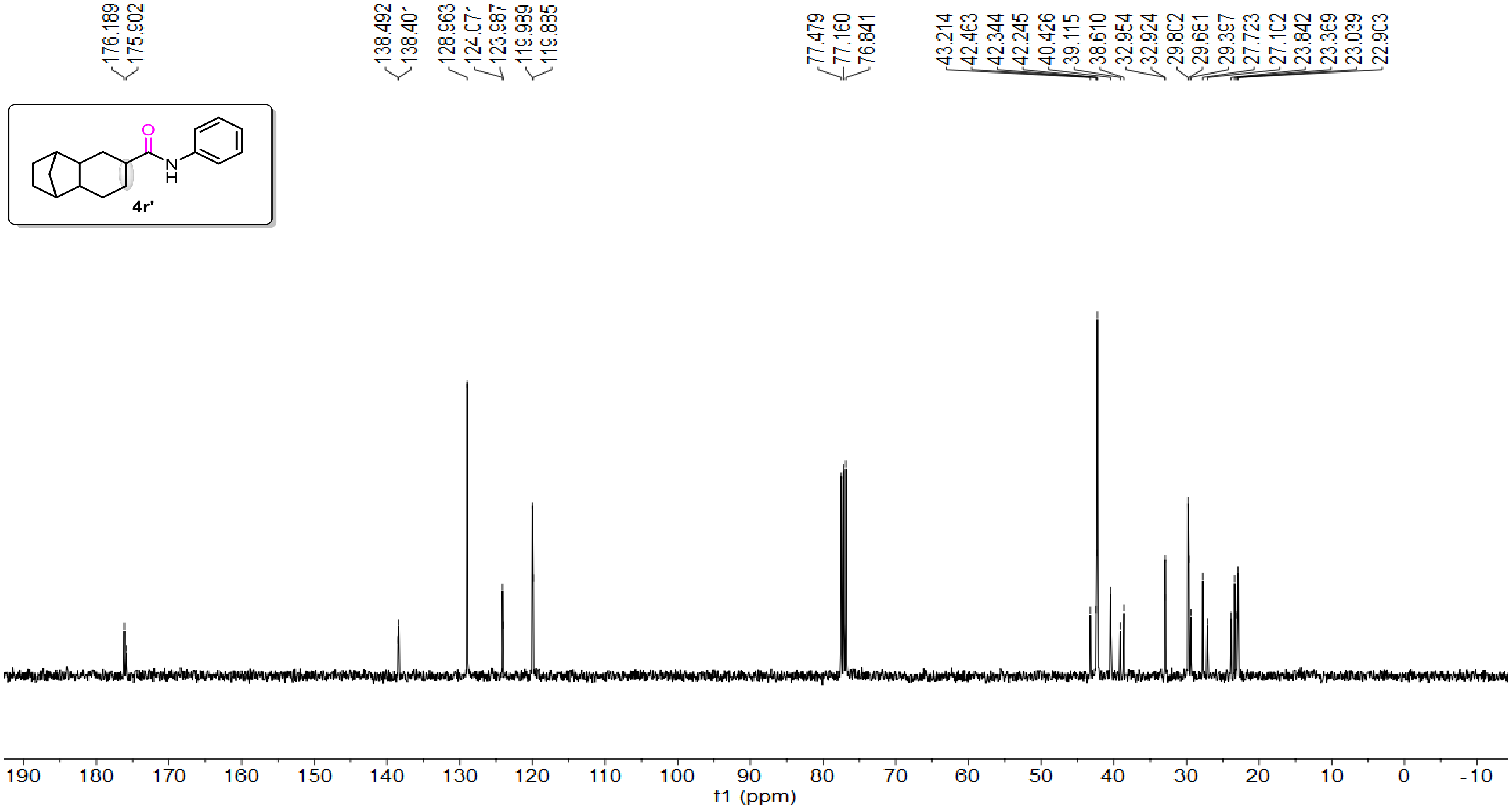

Supplementary Fig. 126. ^{1}H NMR Spectra (400 MHz, $CDCl_3$) of **4s'**

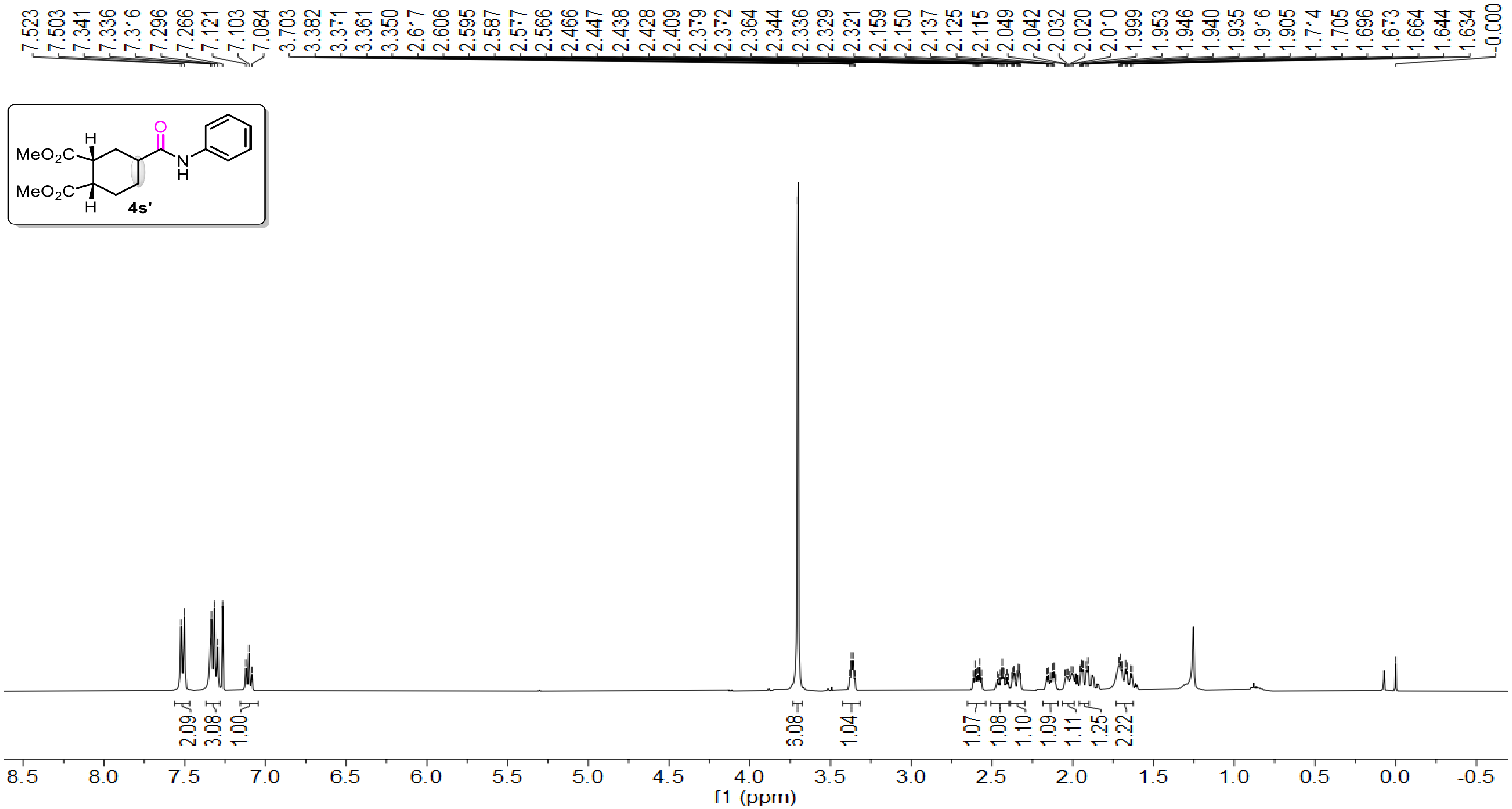

Supplementary Fig. 127. ^{13}C NMR Spectra (101 MHz, $CDCl_3$) of **4s'**

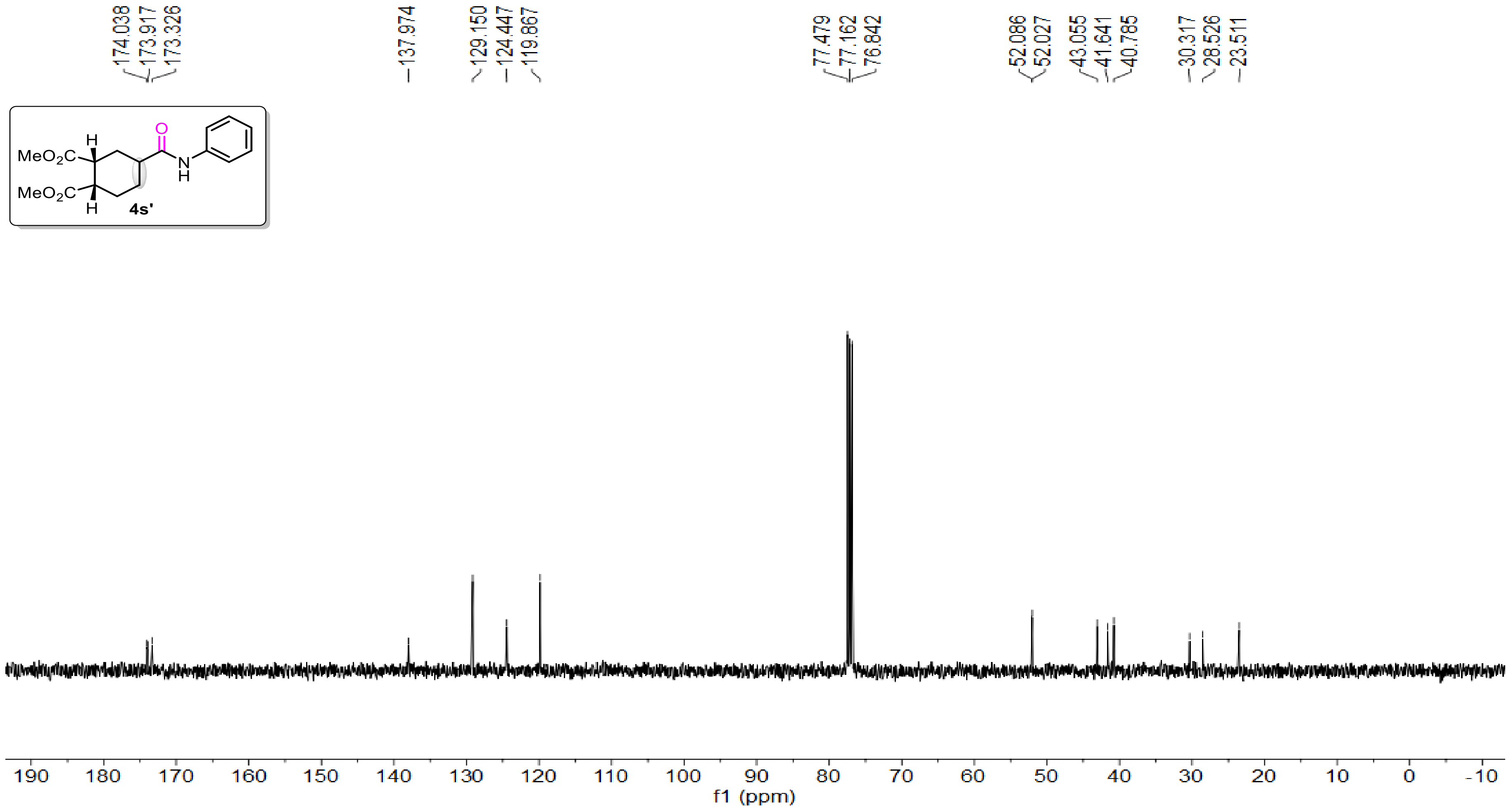

Supplementary Fig. 128. ^{1}H NMR Spectra (400 MHz, $CDCl_3$) of **4t'**

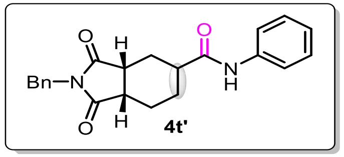

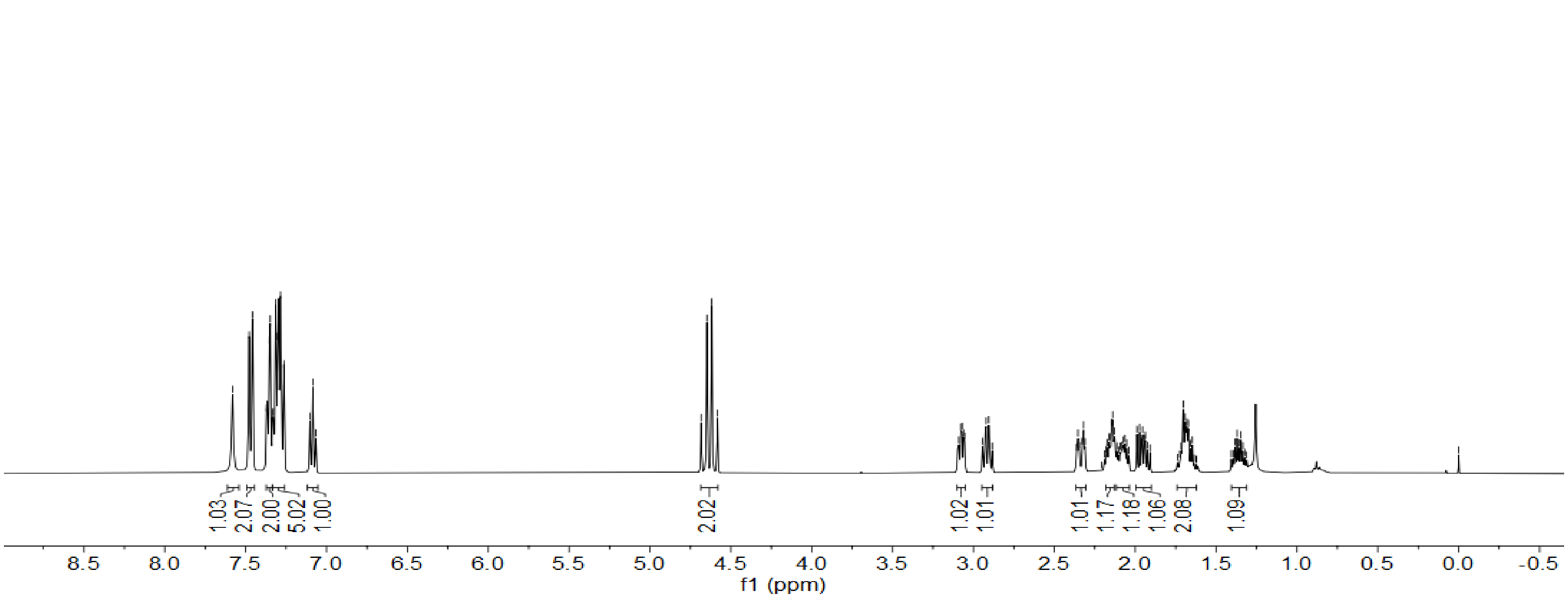

Supplementary Fig. 129. ^{13}C NMR Spectra (101 MHz, $CDCl_3$) of **4t'**

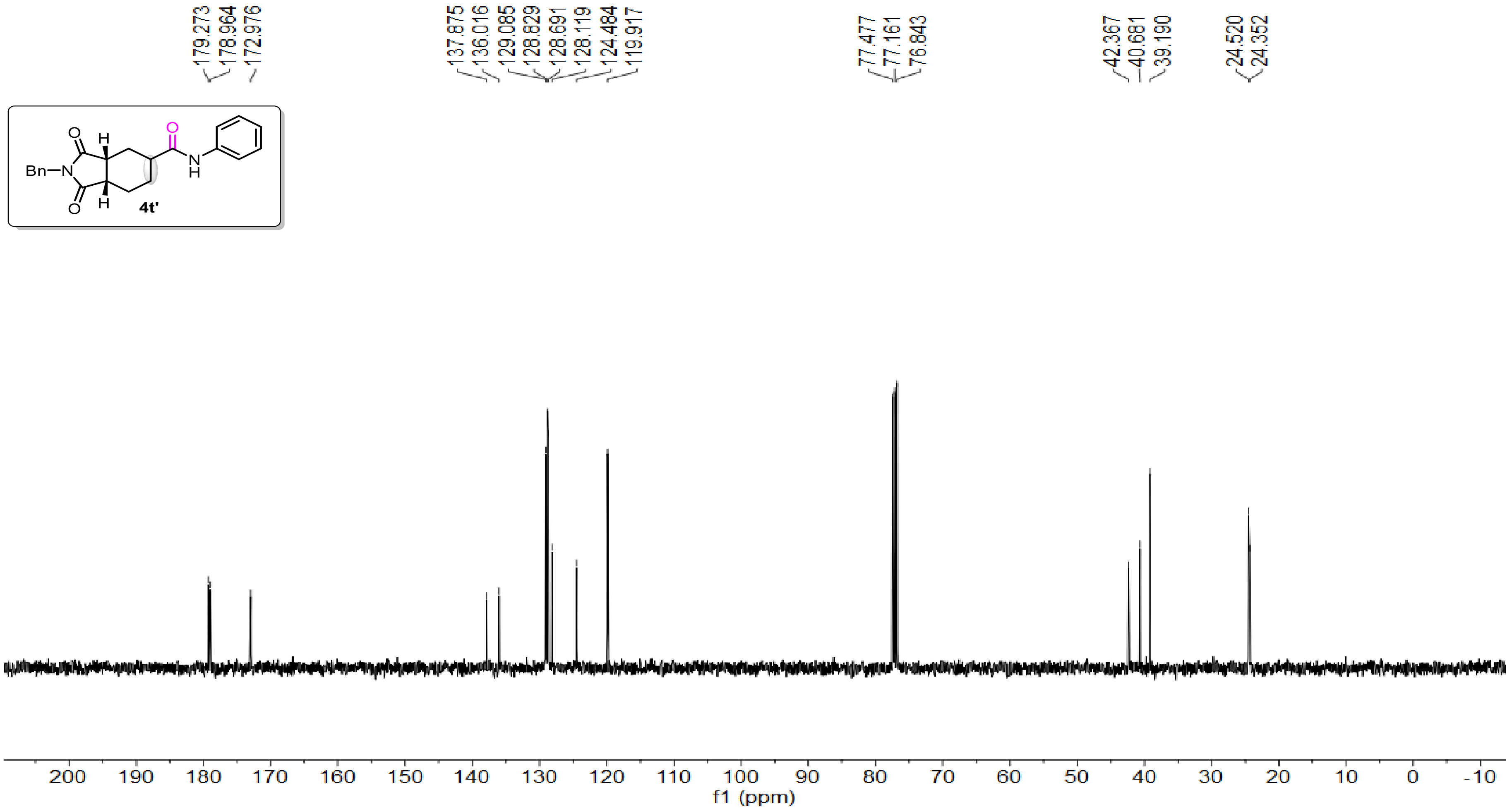

Supplementary Fig. 130. ^{1}H NMR Spectra (400 MHz, $CDCl_3$) of **4u'**

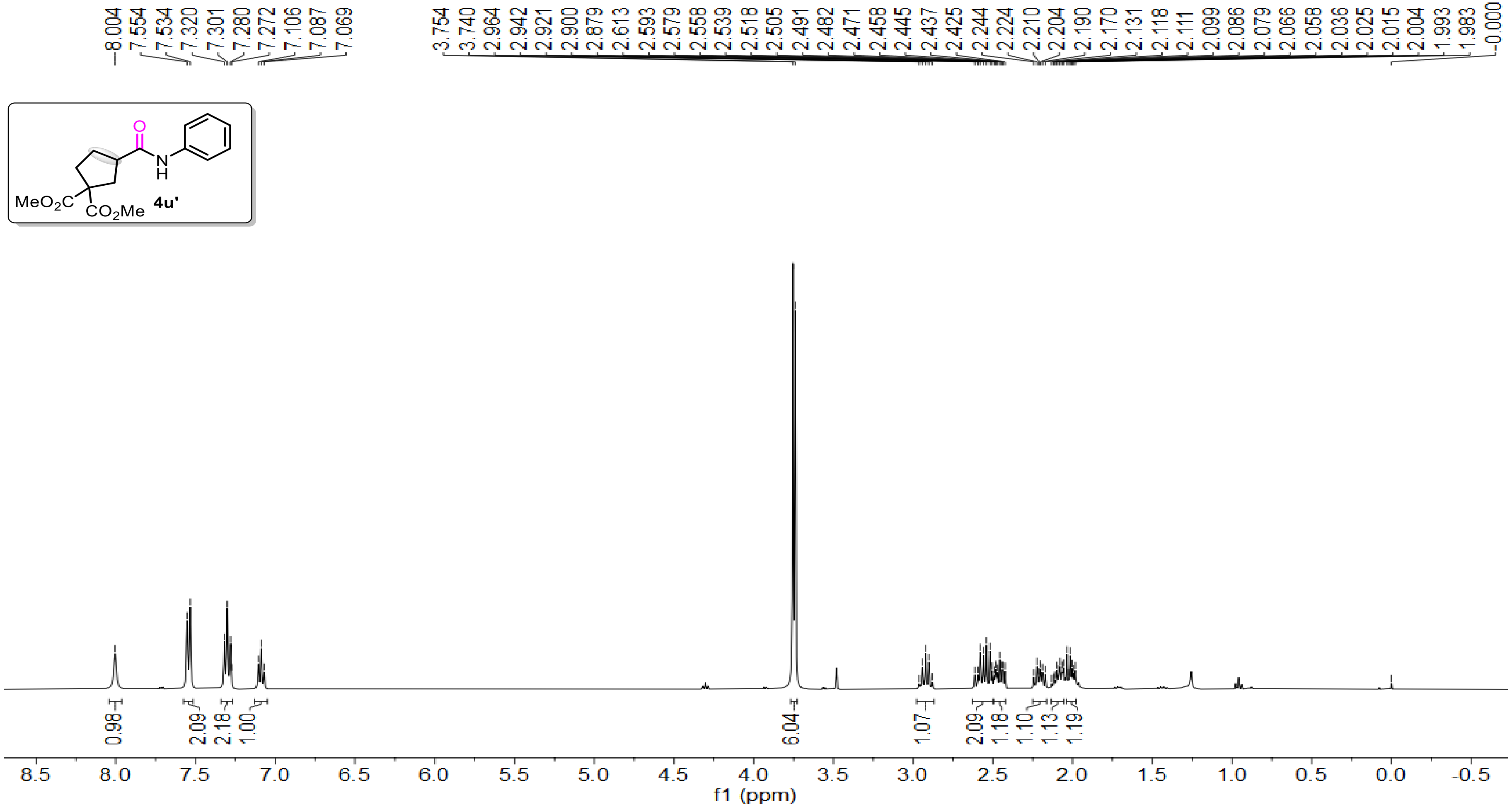

Supplementary Fig. 131. ^{13}C NMR Spectra (101 MHz, $CDCl_3$) of **4u'**

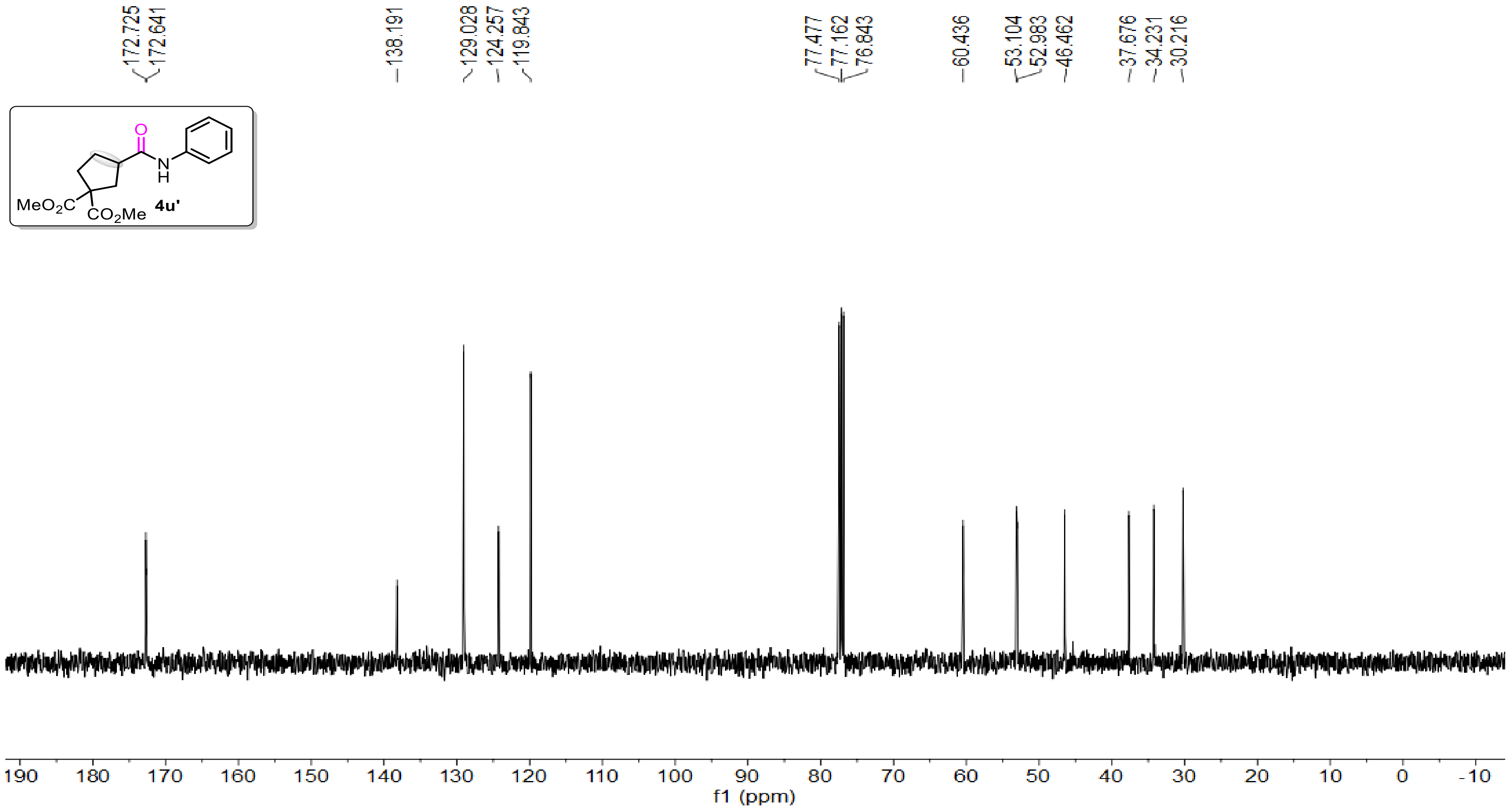

Supplementary Fig. 132. ^{1}H NMR Spectra (400 MHz, $CDCl_3$) of **4v'**

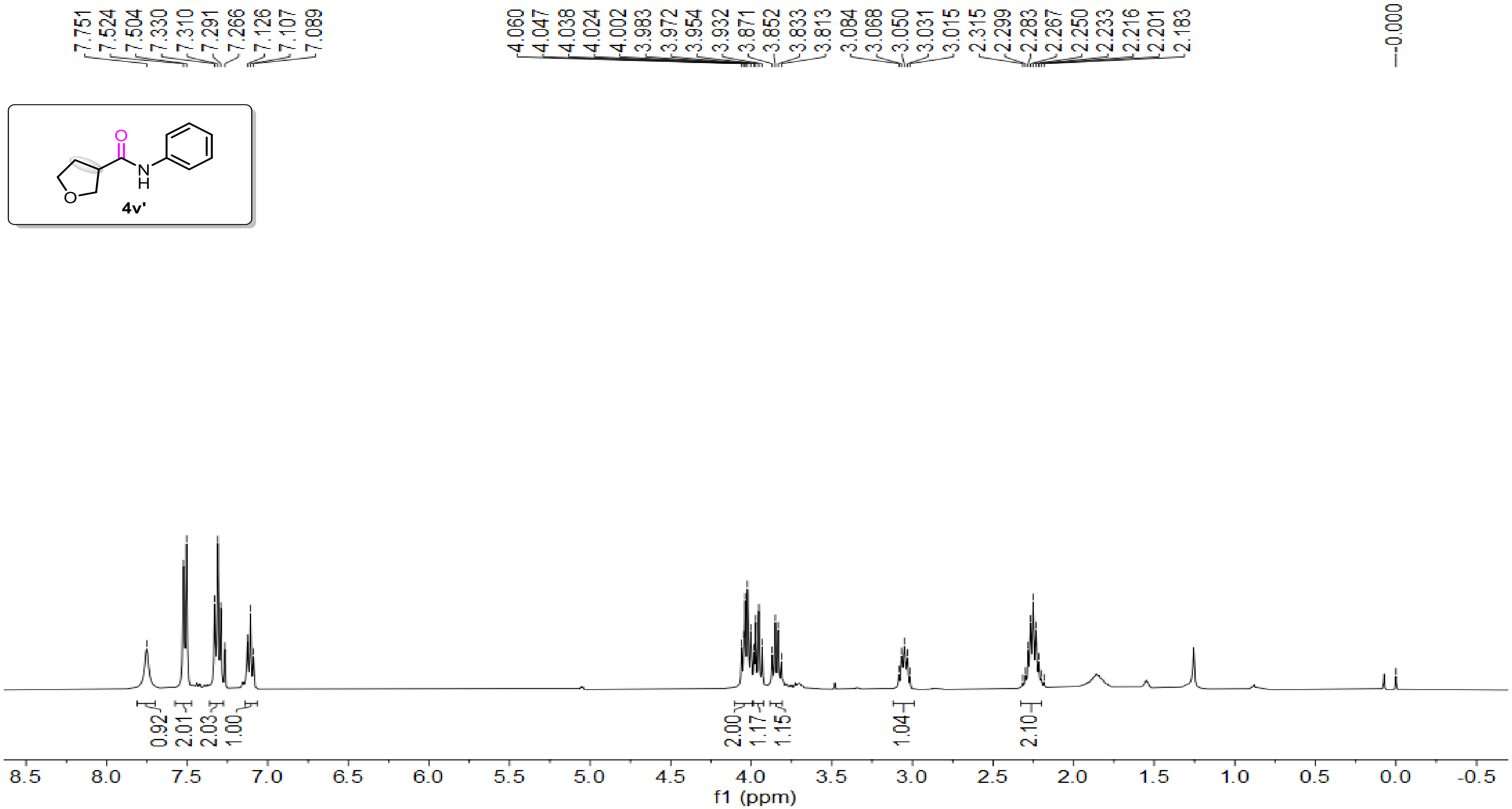

Supplementary Fig. 133. ^{13}C NMR Spectra (101 MHz, $CDCl_3$) of **4v'**

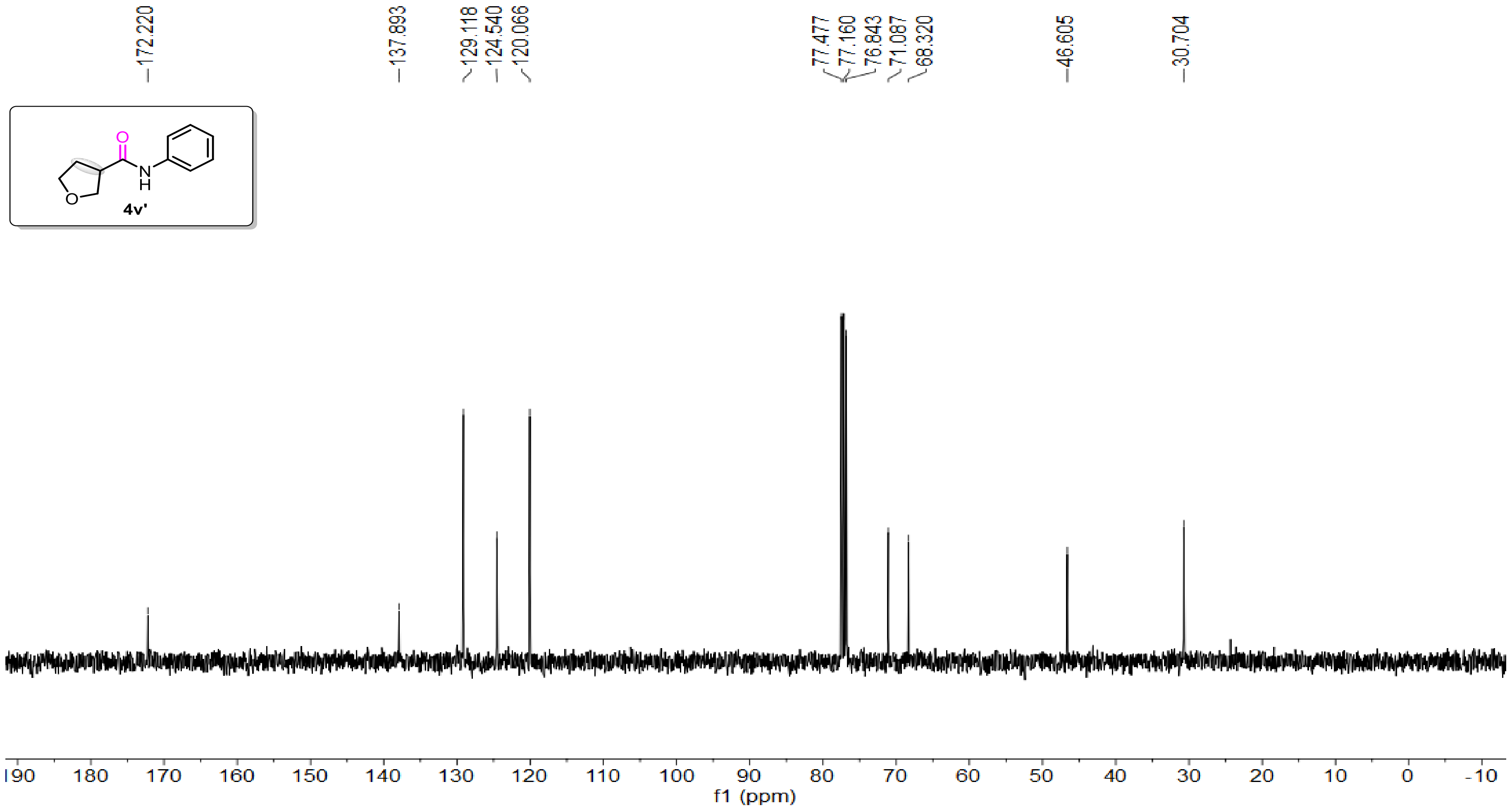

Supplementary Fig. 134. ^{1}H NMR Spectra (400 MHz, $CDCl_3$) of **4w'**

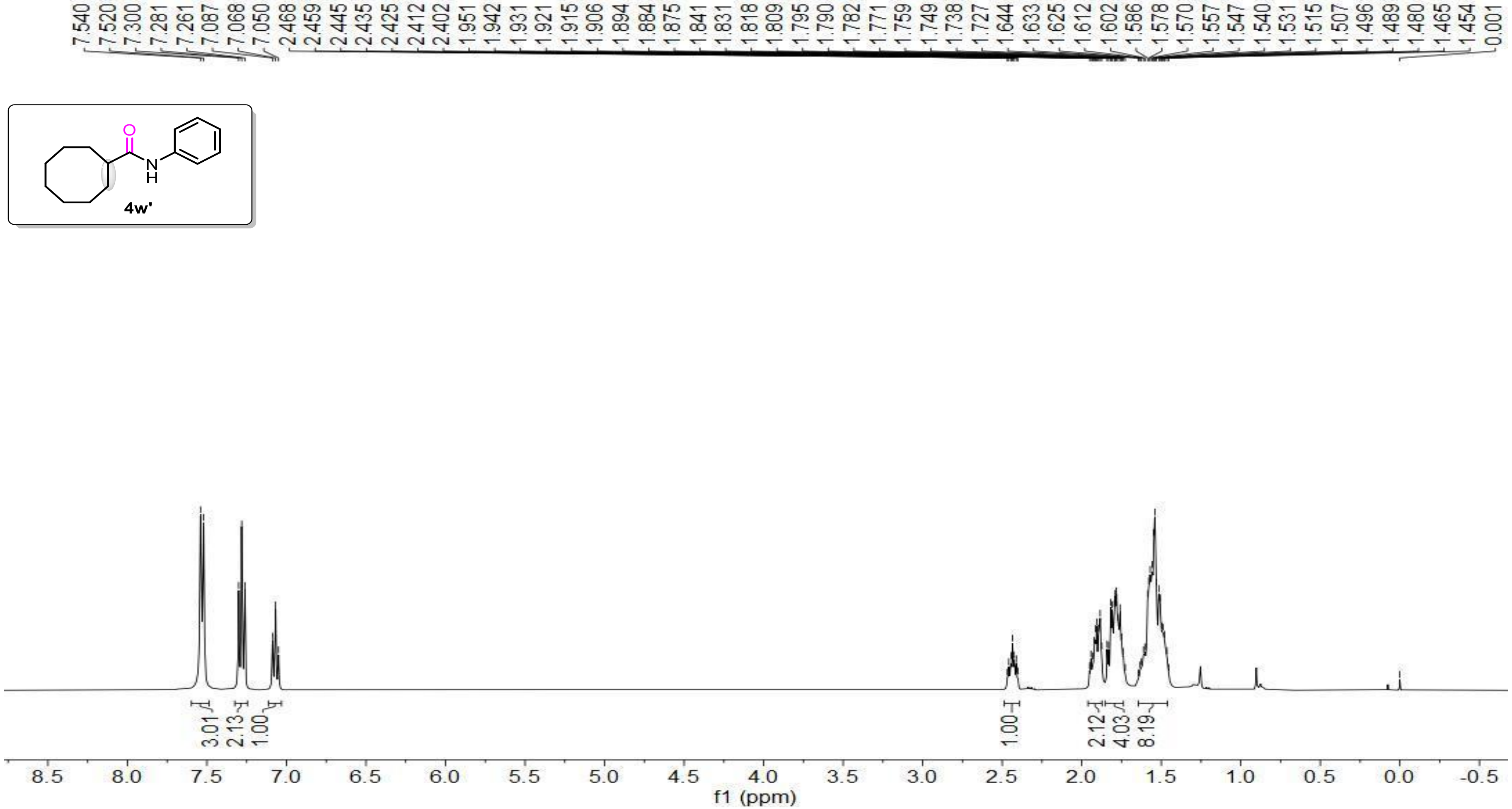

Supplementary Fig. 135. ^{13}C NMR Spectra (101 MHz, $CDCl_3$) of **4w'**

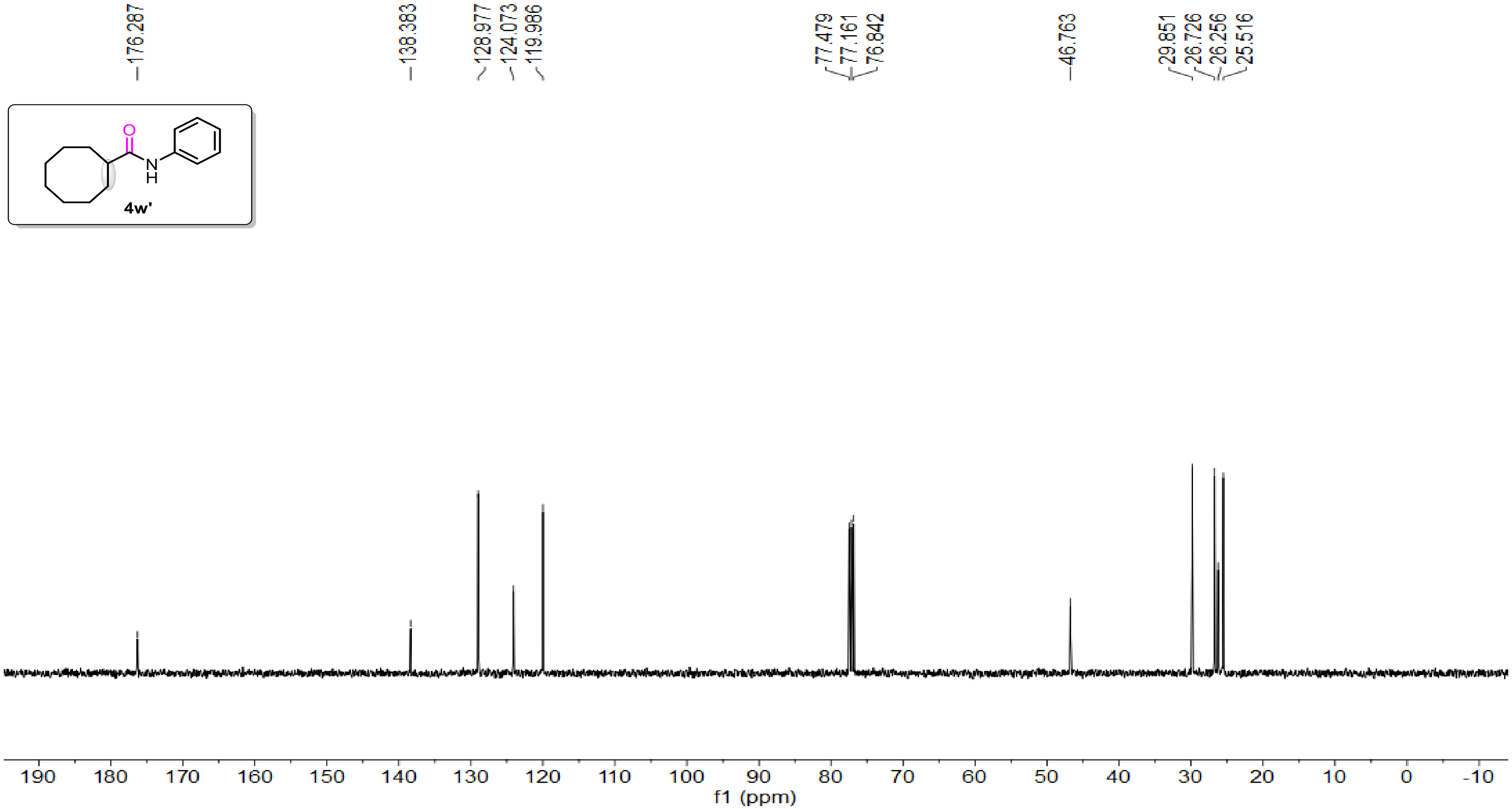

Supplementary Fig. 136. ^{1}H NMR Spectra (400 MHz, $CDCl_3$) of **4x'**

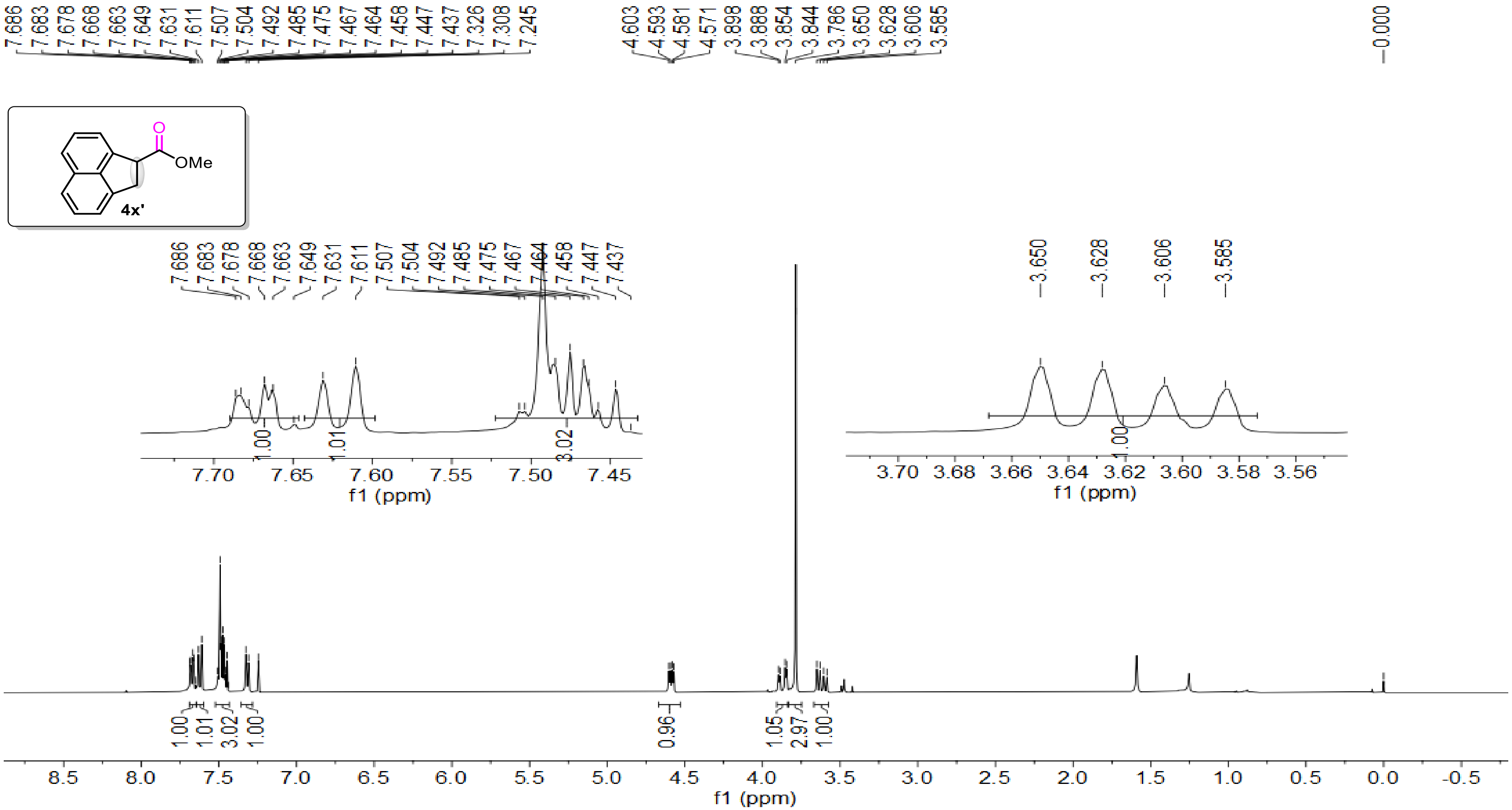

Supplementary Fig. 137. ^{13}C NMR Spectra (101 MHz, $CDCl_3$) of **4x'**

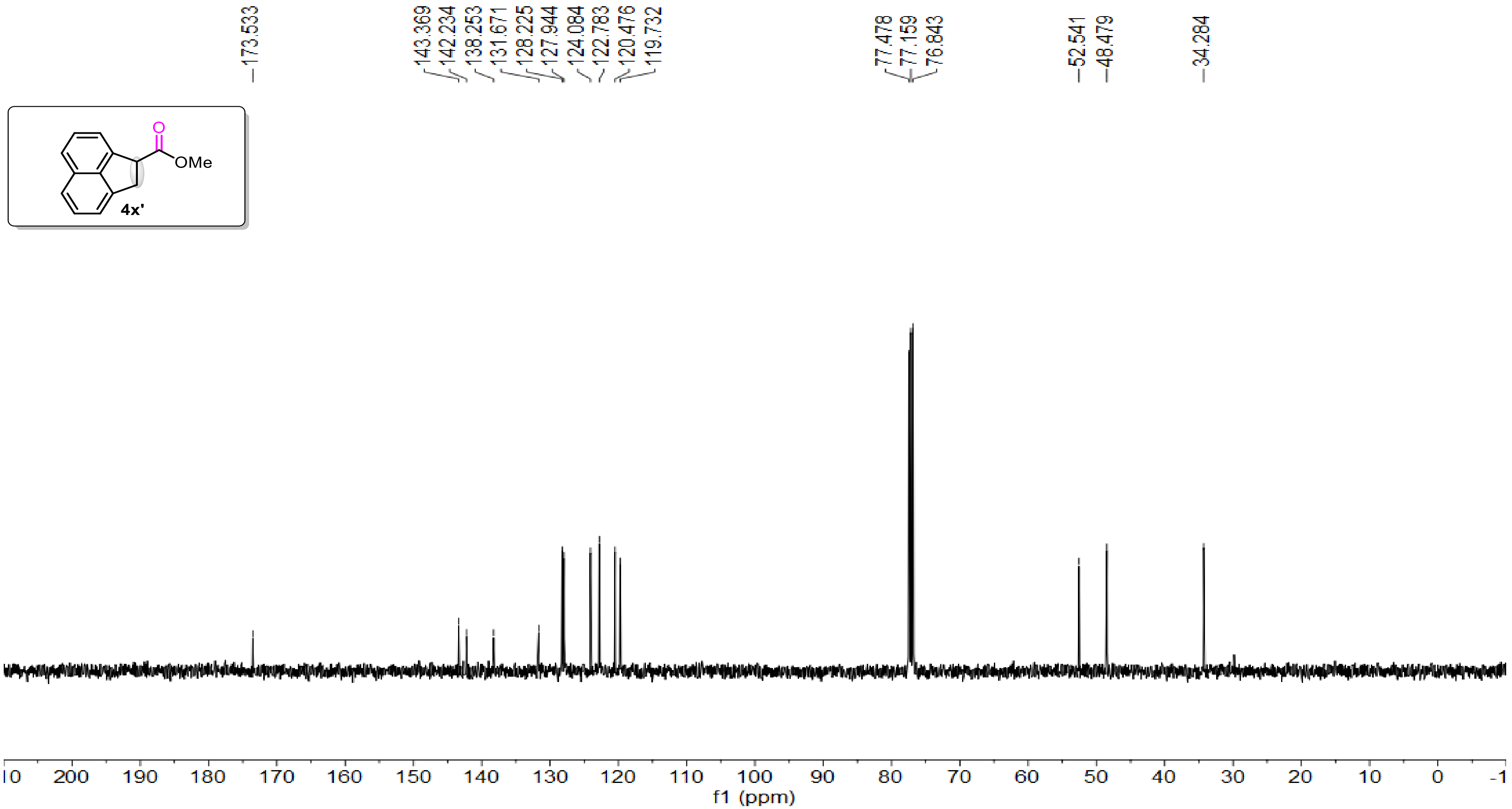

Supplementary Fig. 138. ^{1}H NMR Spectra (400 MHz, $CDCl_3$) of **4y'**

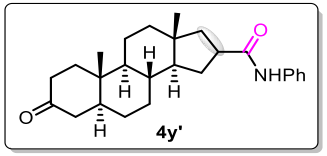

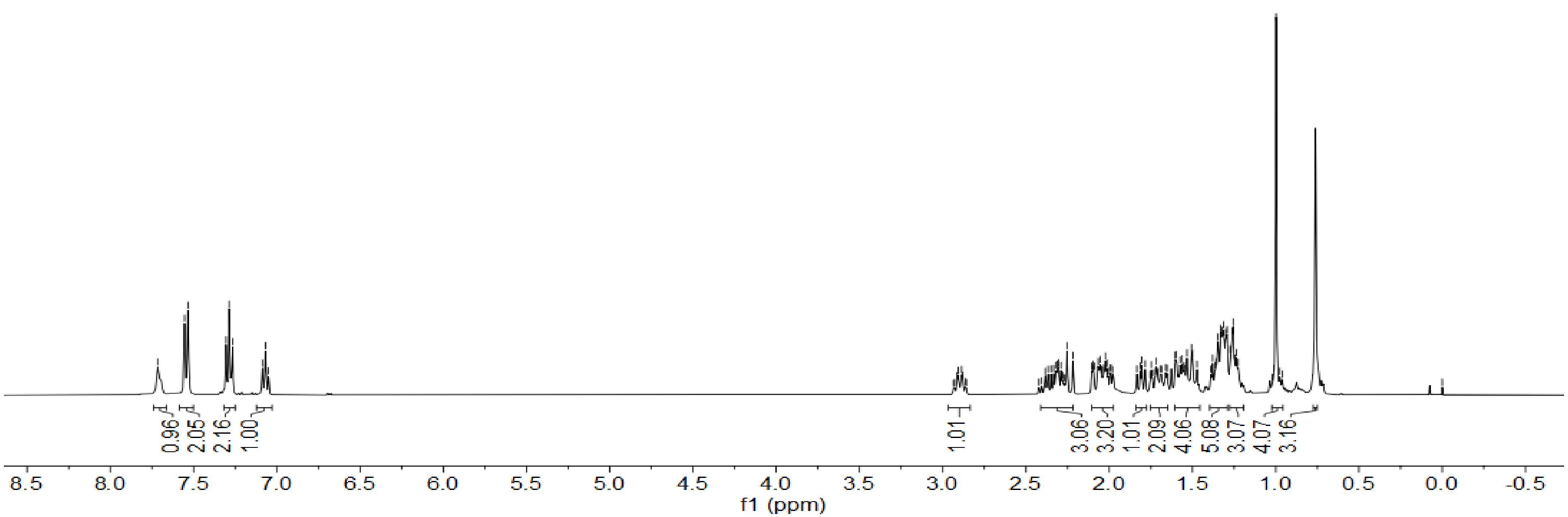

Supplementary Fig. 139. ^{13}C NMR Spectra (101 MHz, $CDCl_3$) of **4y'**

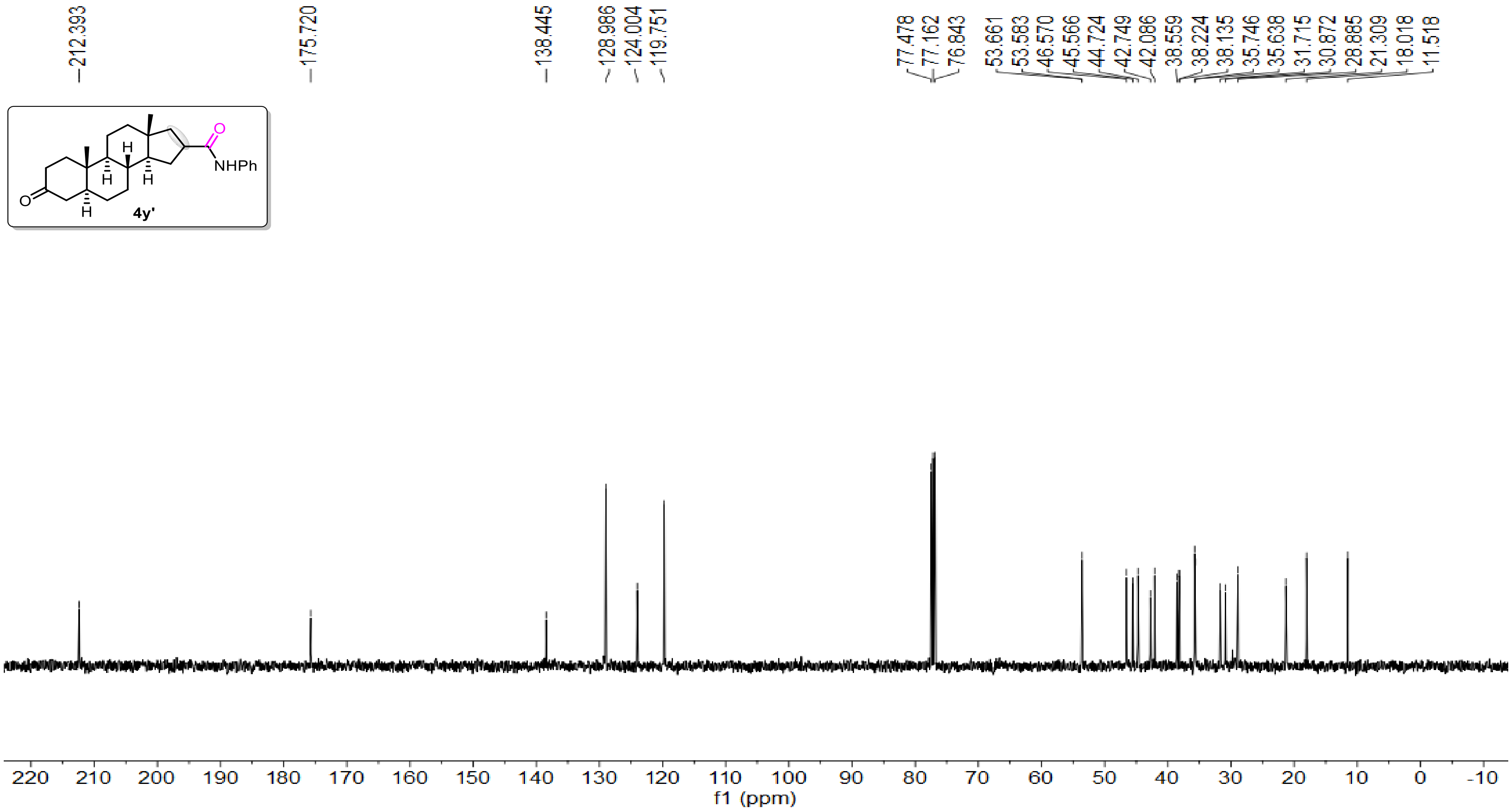

Supplementary Fig. 140. ^{1}H NMR Spectra (400 MHz, $CDCl_3$) of **5a**

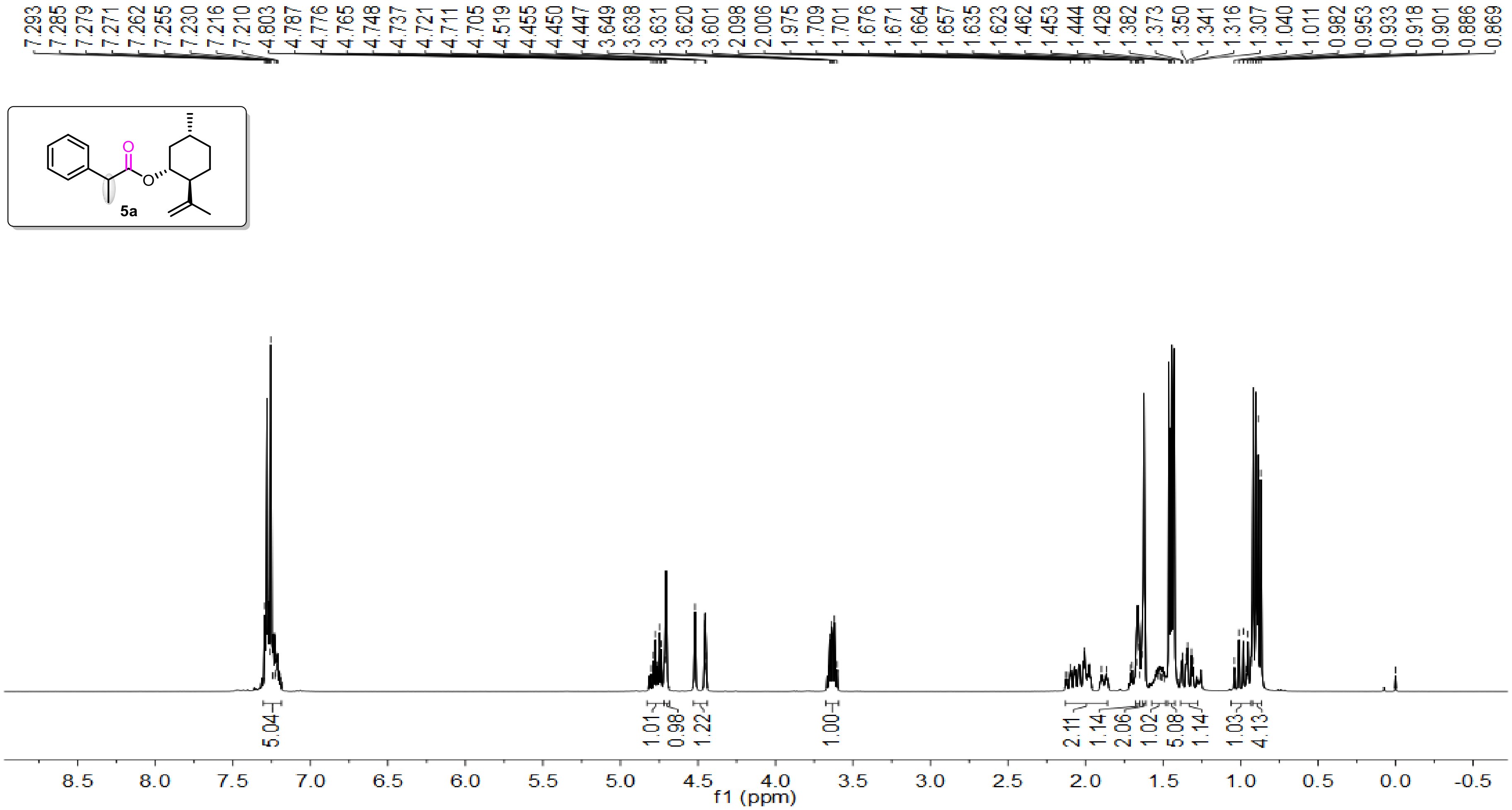

Supplementary Fig. 141. ^{13}C NMR Spectra (101 MHz, $CDCl_3$) of **5a**

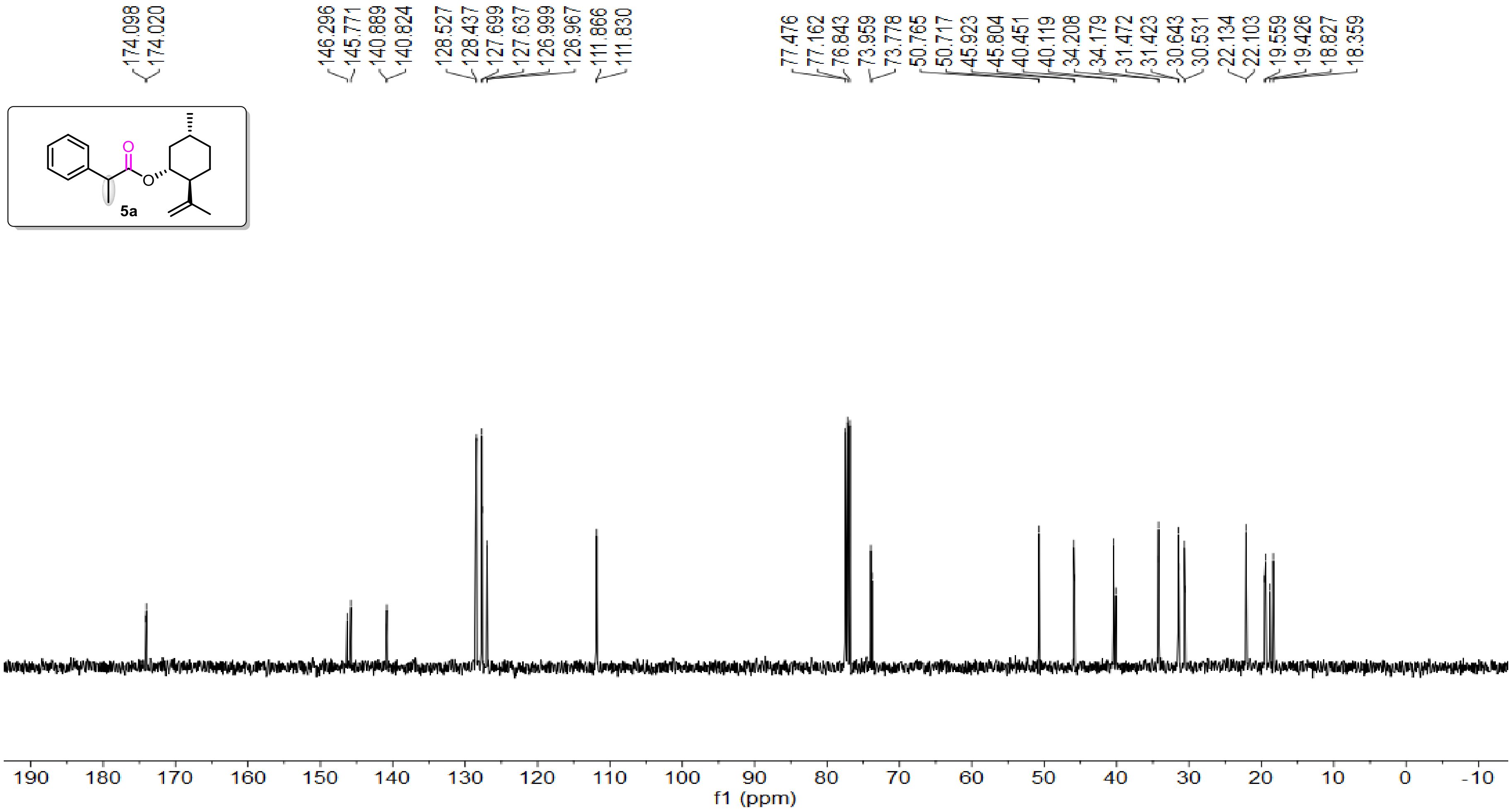

Supplementary Fig. 142. ^{1}H NMR Spectra (400 MHz, $CDCl_3$) of **5b**

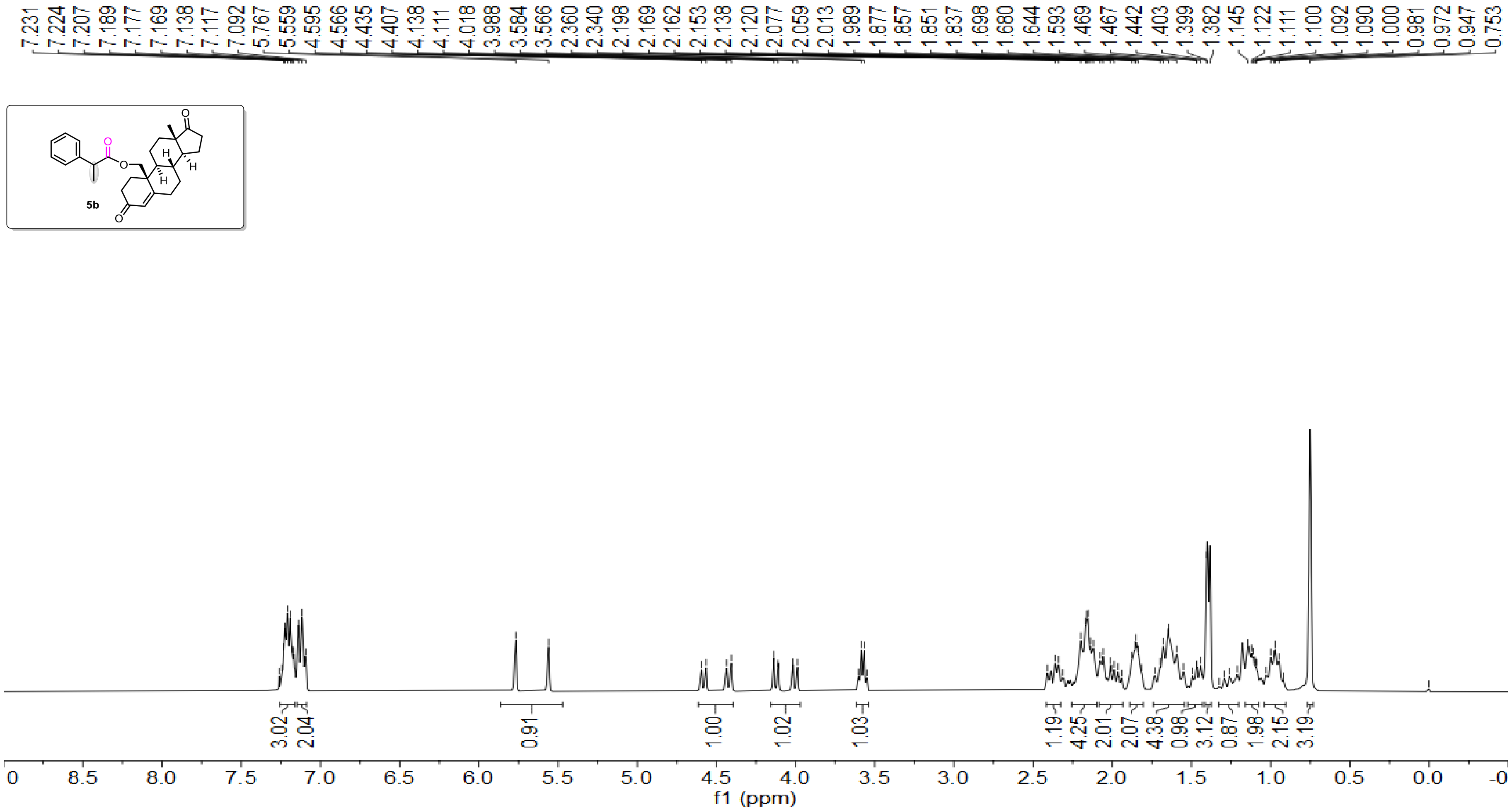

Supplementary Fig. 143. ^{13}C NMR Spectra (101 MHz, $CDCl_3$) of **5b**

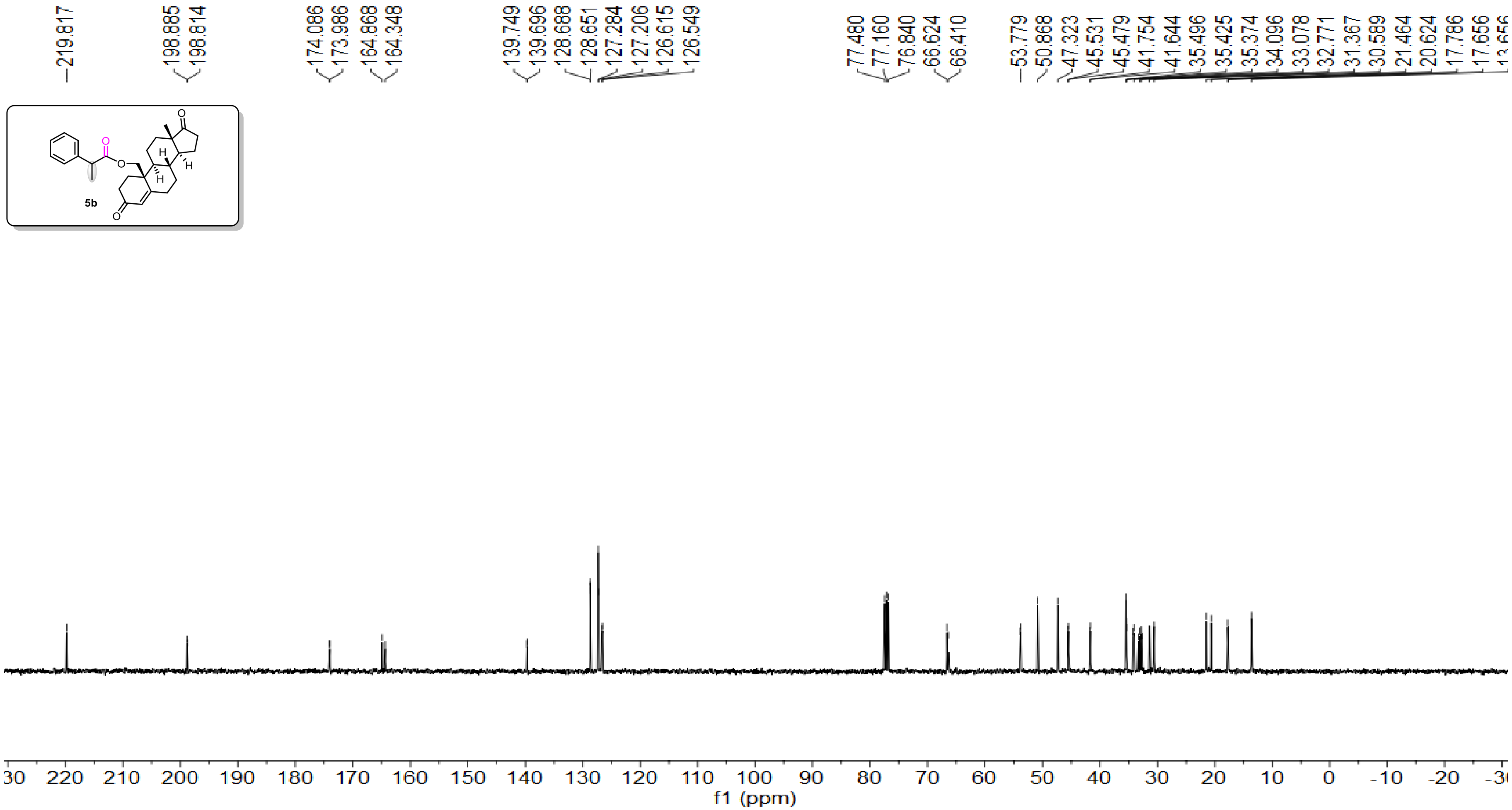

Supplementary Fig. 144. ^{1}H NMR Spectra (400 MHz, $CDCl_3$) of **5c**

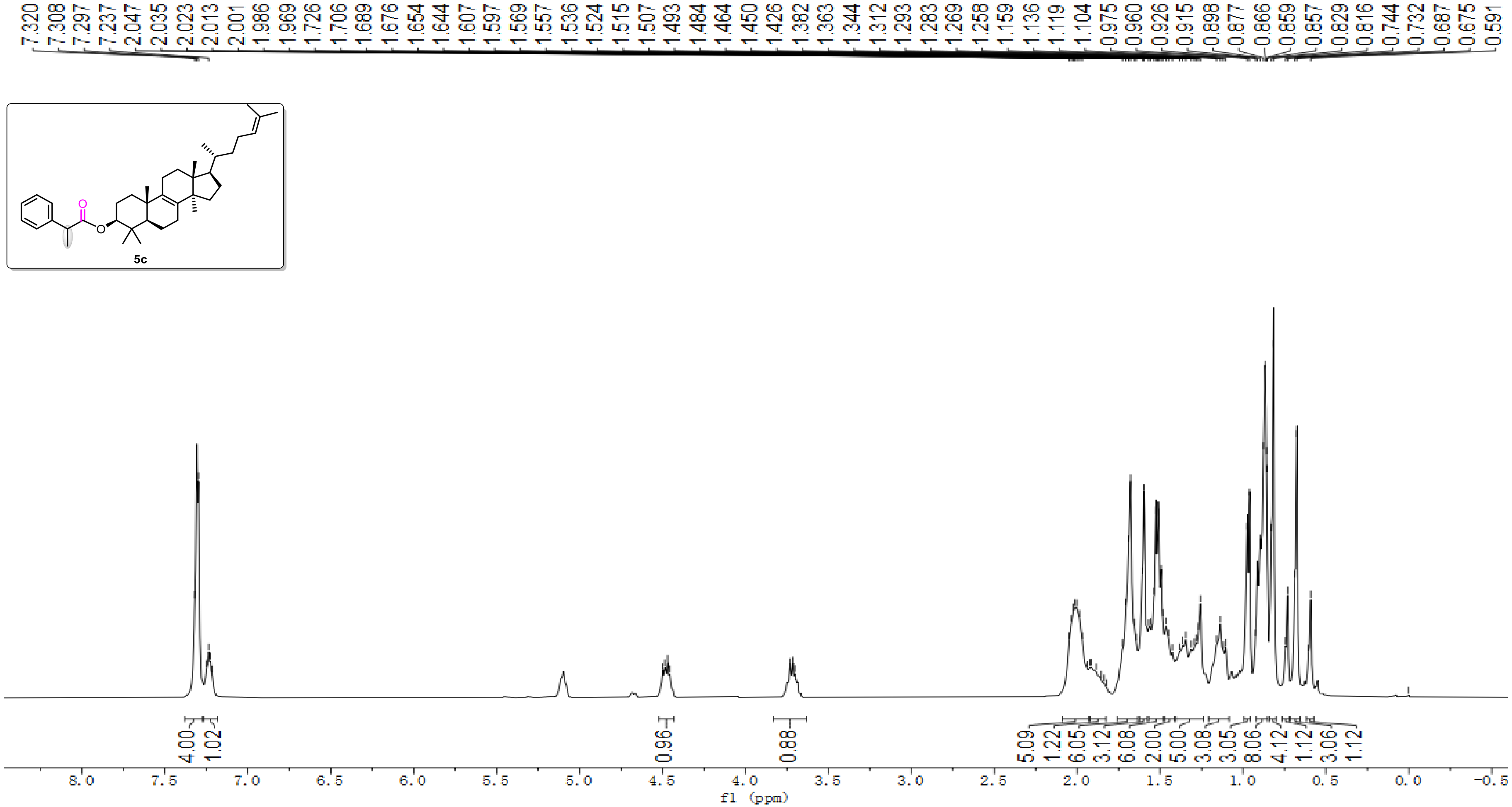

Supplementary Fig. 145. ^{13}C NMR Spectra (101 MHz, CDCl$_3$) of **5c**

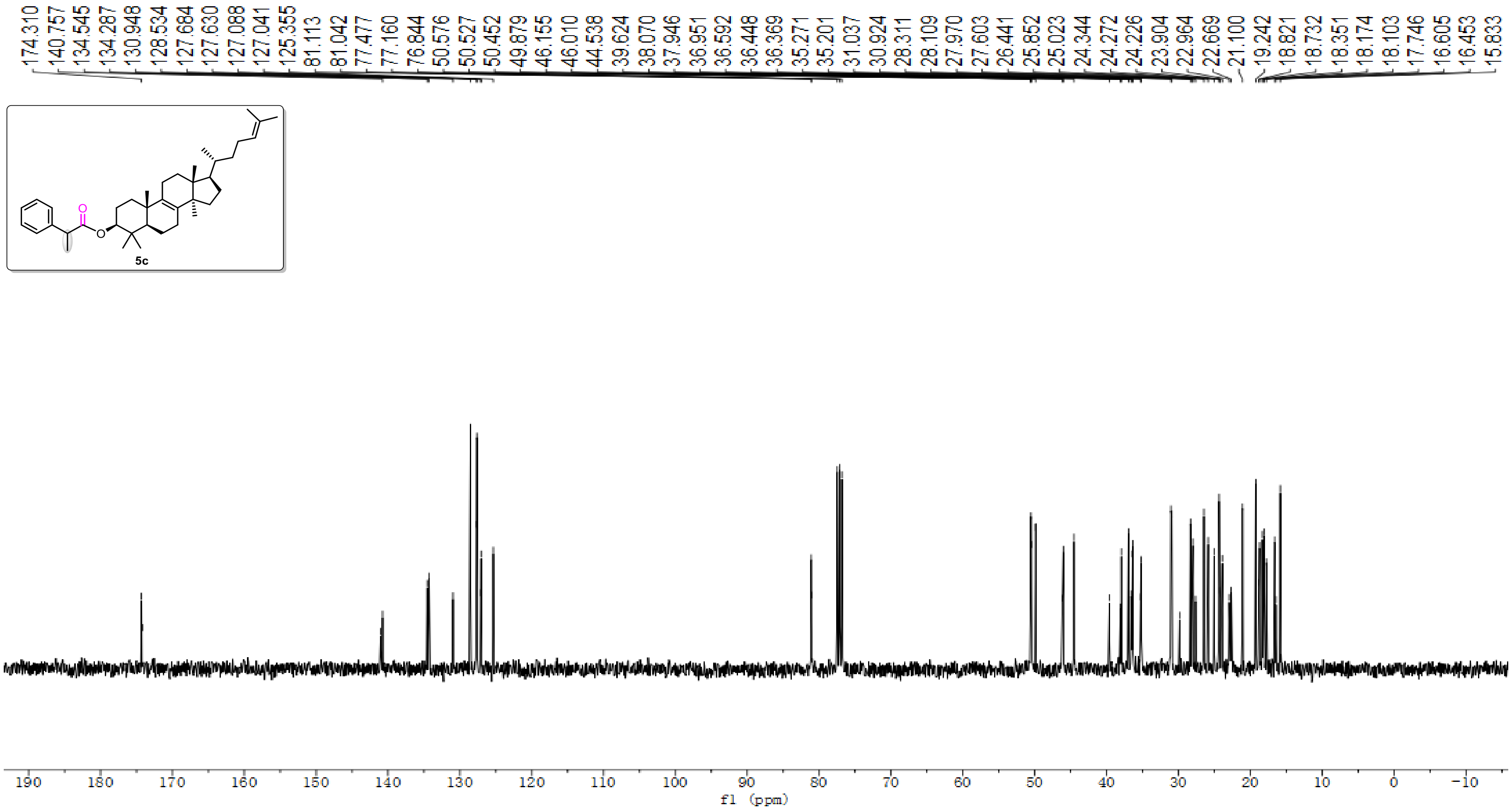

Supplementary Fig. 146. ^{1}H NMR Spectra (400 MHz, $CDCl_3$) of **5d**

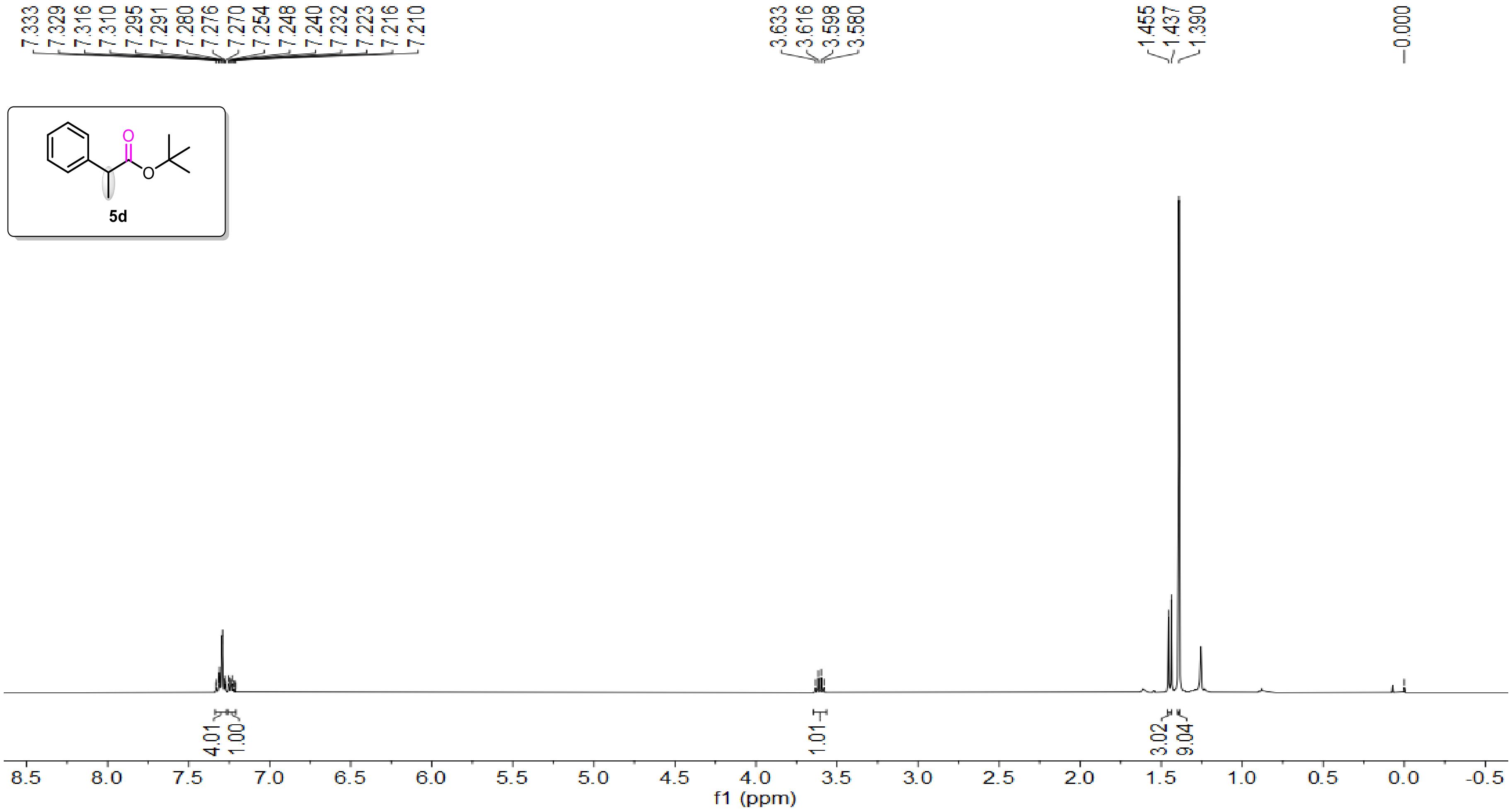

Supplementary Fig. 147. ^{13}C NMR Spectra (101 MHz, $CDCl_3$) of **5d**

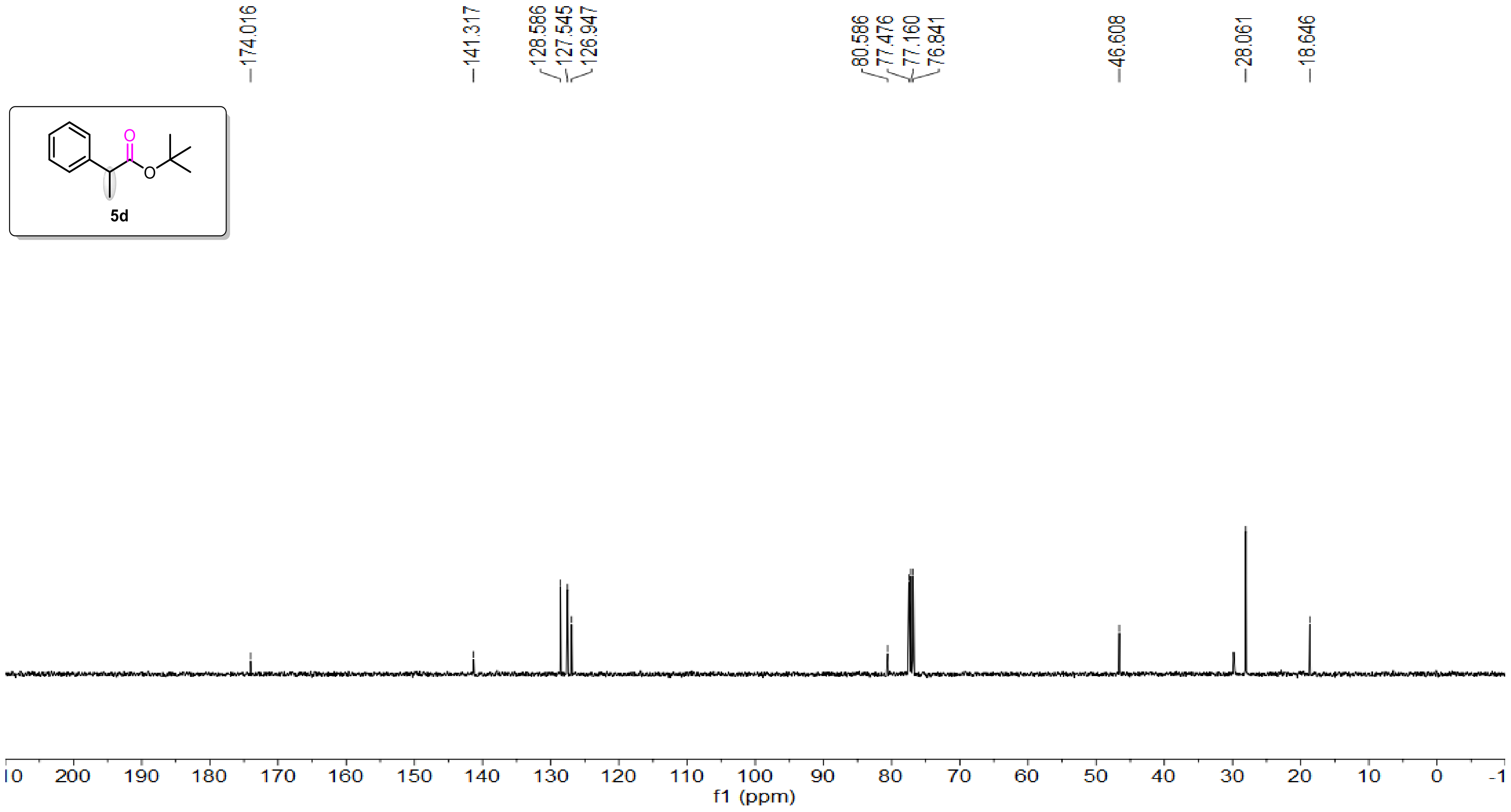

Supplementary Fig. 148. ^1H NMR Spectra (400 MHz, $CDCl_3$) of **5e**

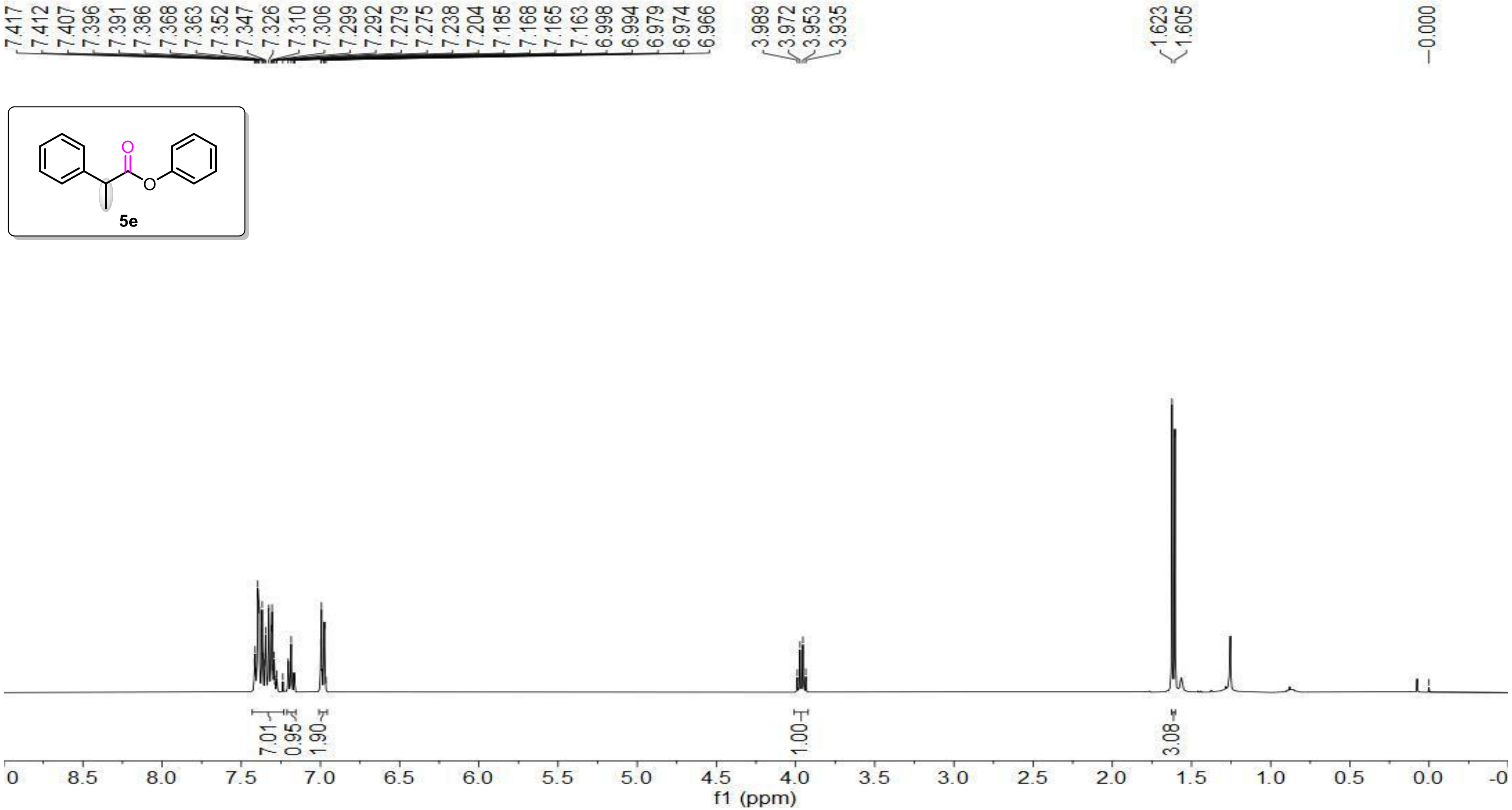

Supplementary Fig. 149. ^{13}C NMR Spectra (101 MHz, $CDCl_3$) of **5e**

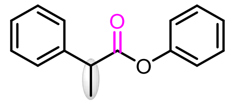

5e

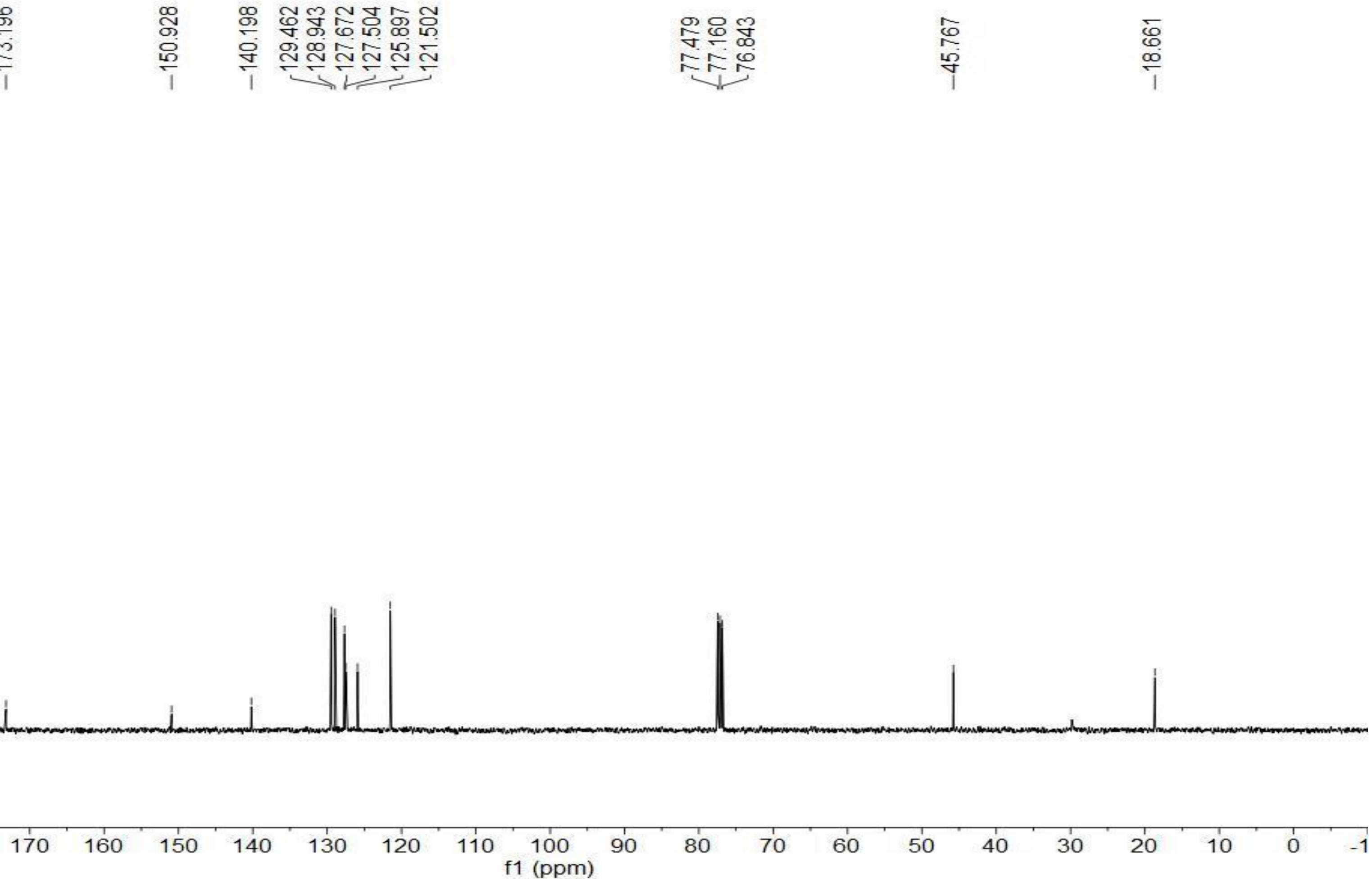

Supplementary Fig. 150. ^{1}H NMR Spectra (400 MHz, $CDCl_3$) of **5f**

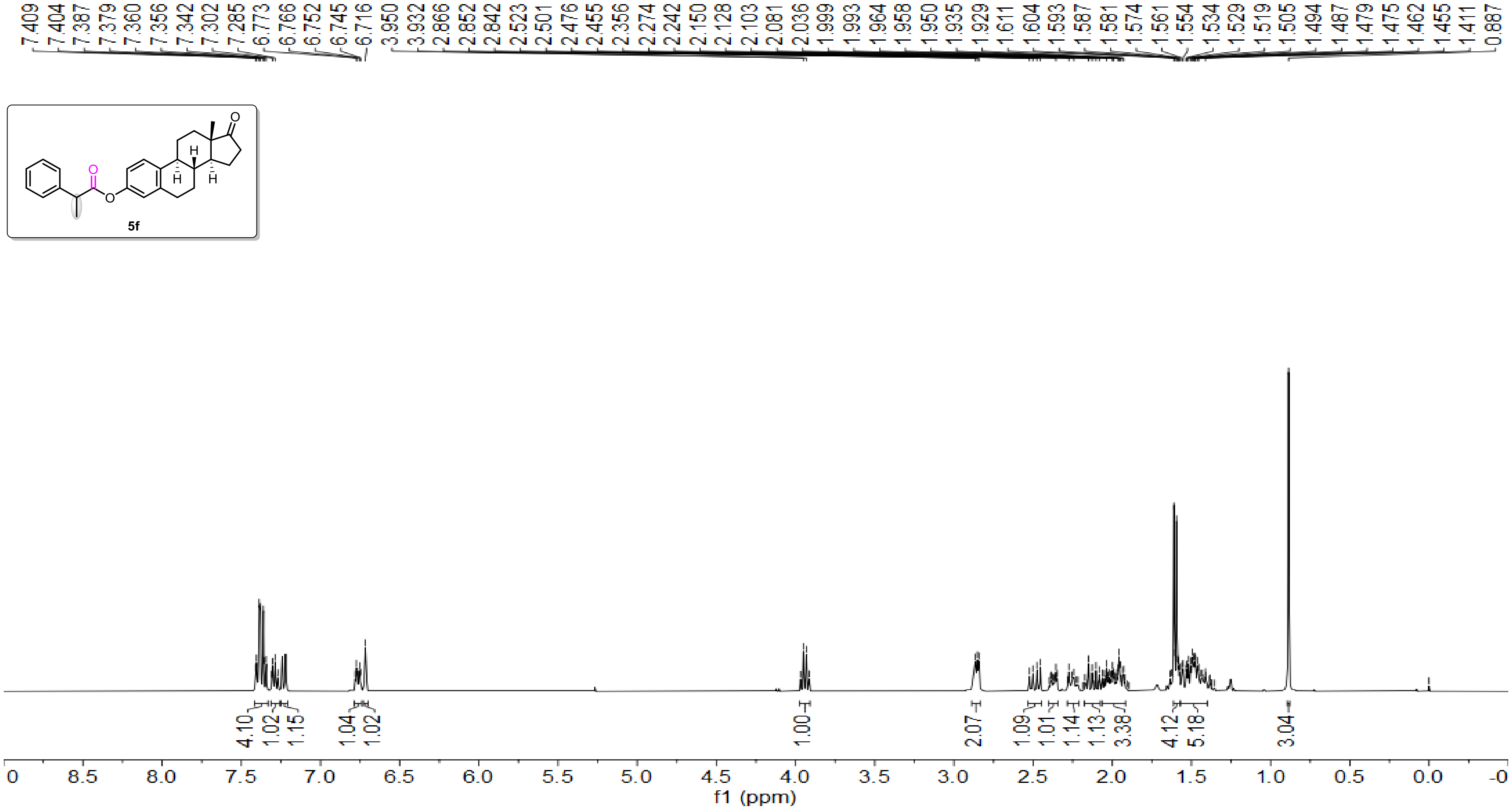

Supplementary Fig. 151. ^{13}C NMR Spectra (101 MHz, $CDCl_3$) of **5f**

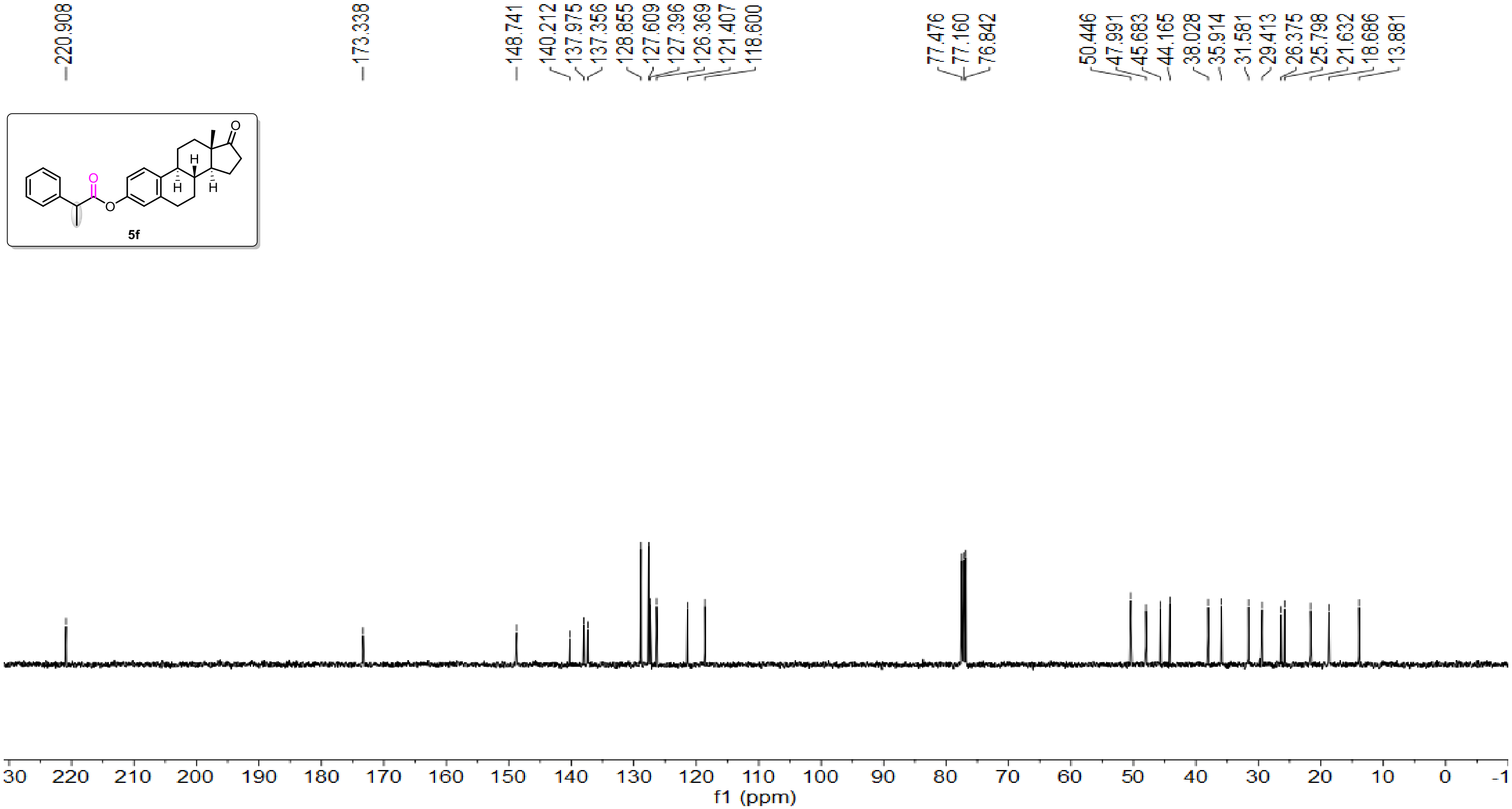

Supplementary Fig. 152. ^{1}H NMR Spectra (400 MHz, $CDCl_3$) of **5g**

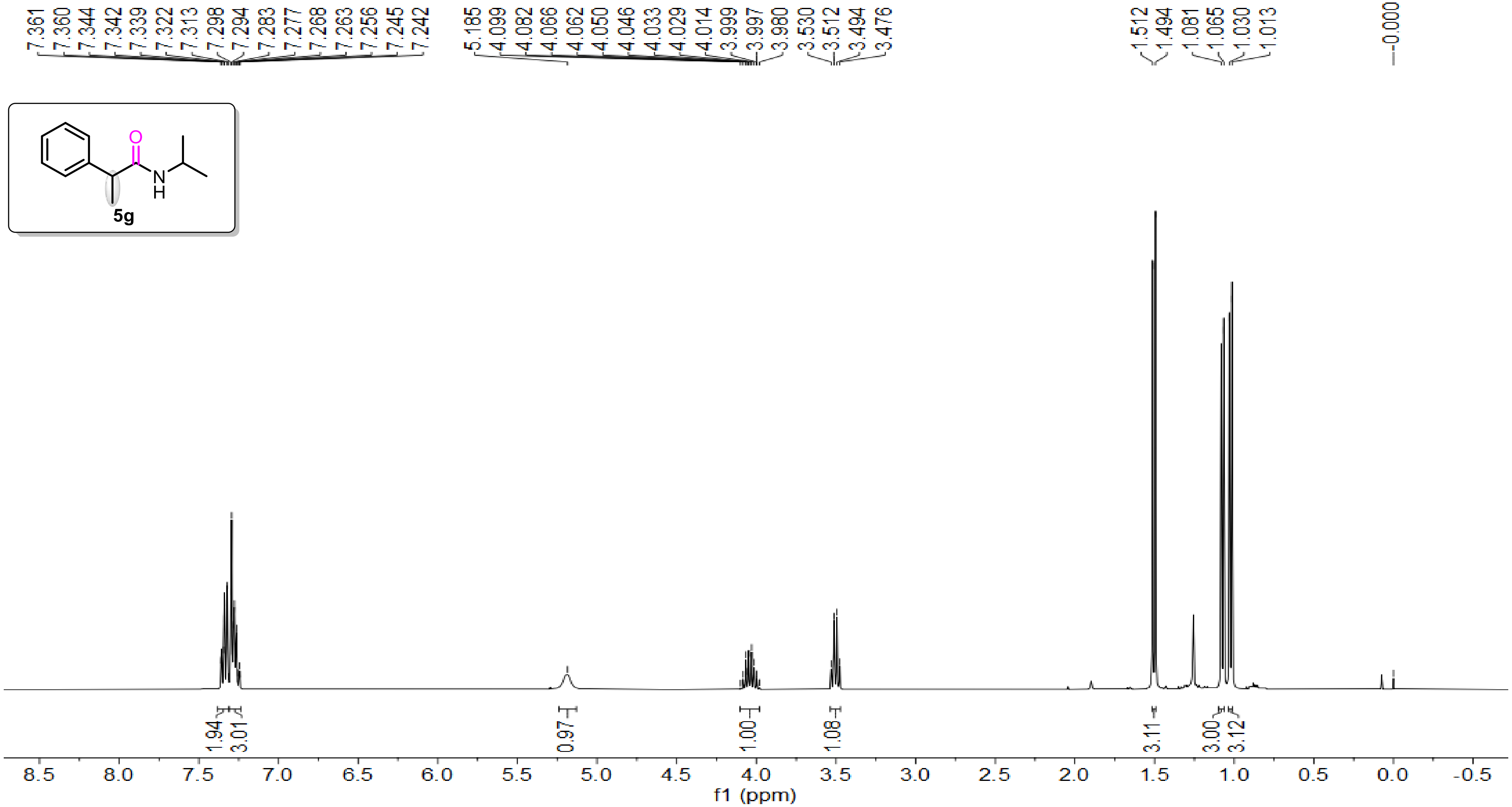

Supplementary Fig. 153. ^{13}C NMR Spectra (101 MHz, $CDCl_3$) of **5g**

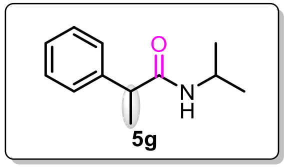

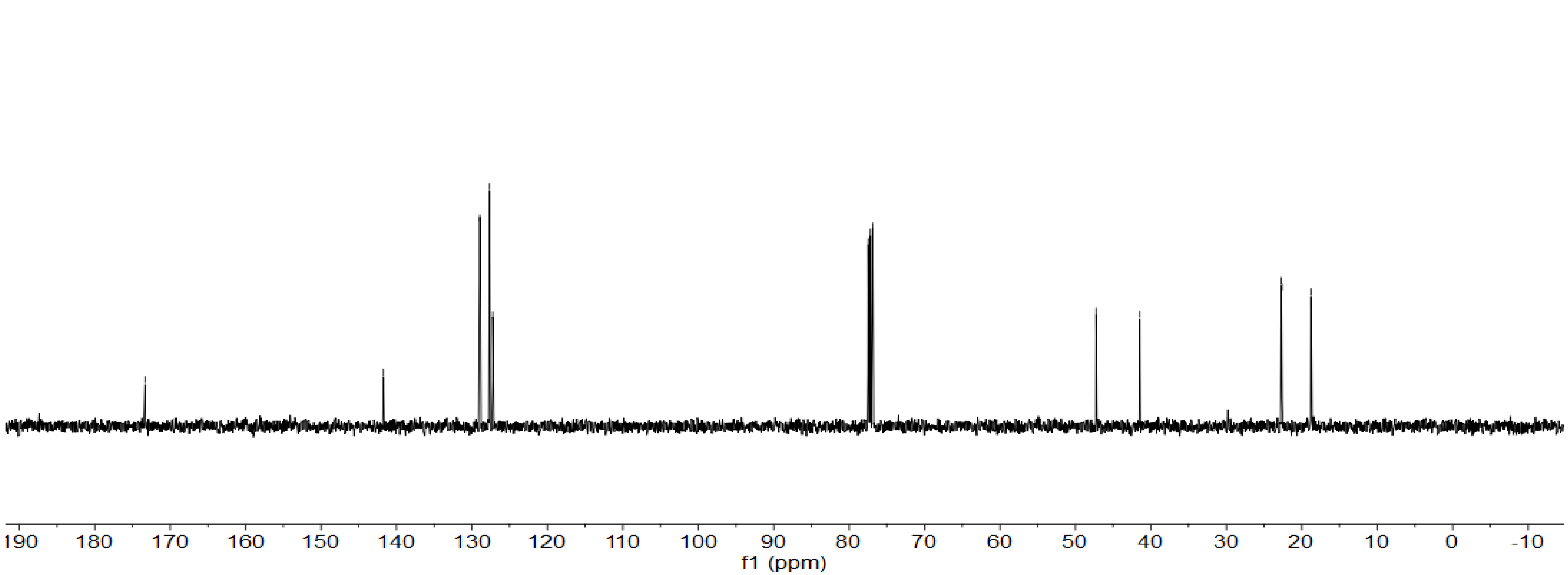

Supplementary Fig. 154. ^{1}H NMR Spectra (400 MHz, $CDCl_3$) of **5h**

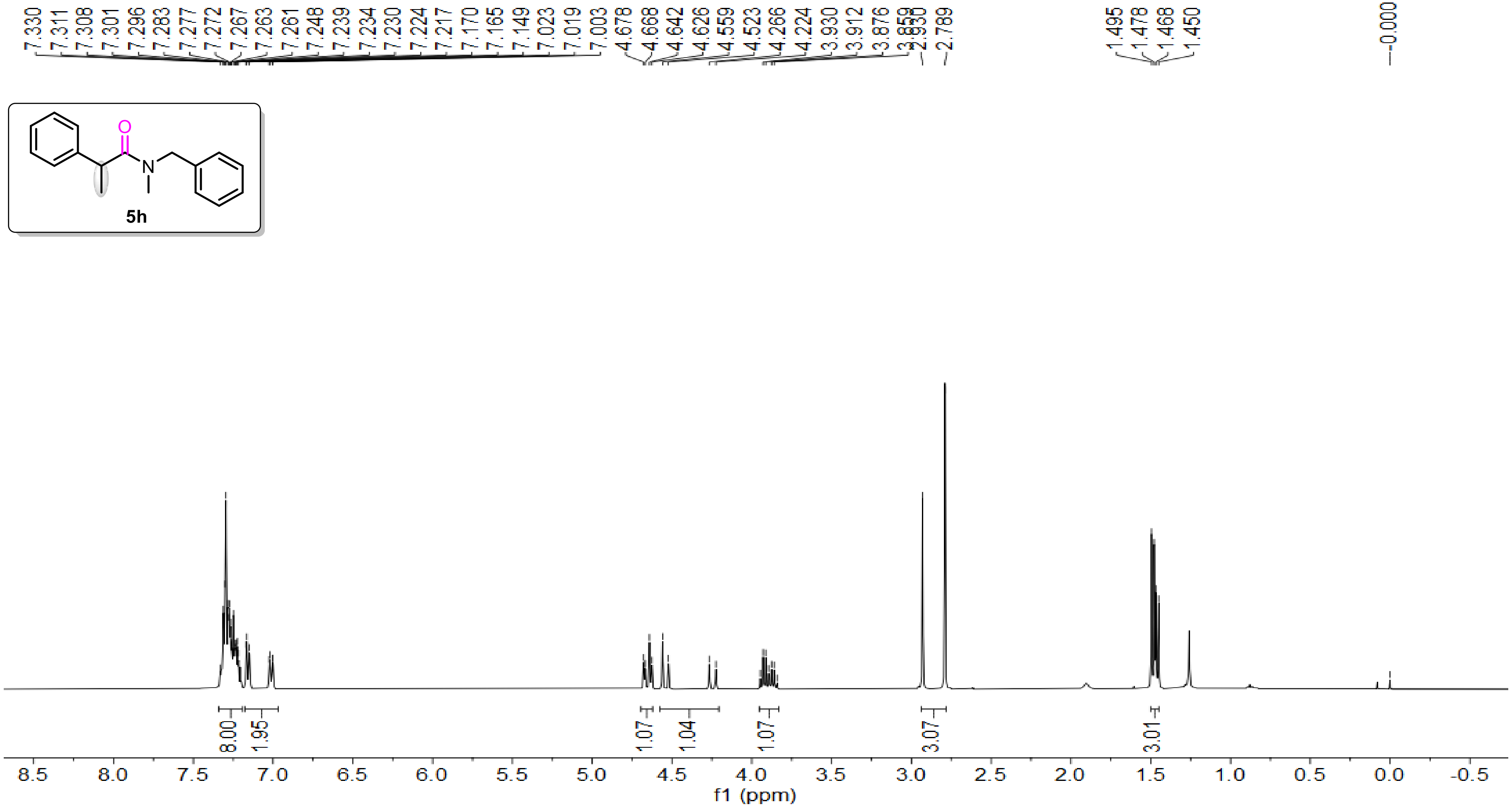

Supplementary Fig. 155. ^{13}C NMR Spectra (101 MHz, $CDCl_3$) of **5h**

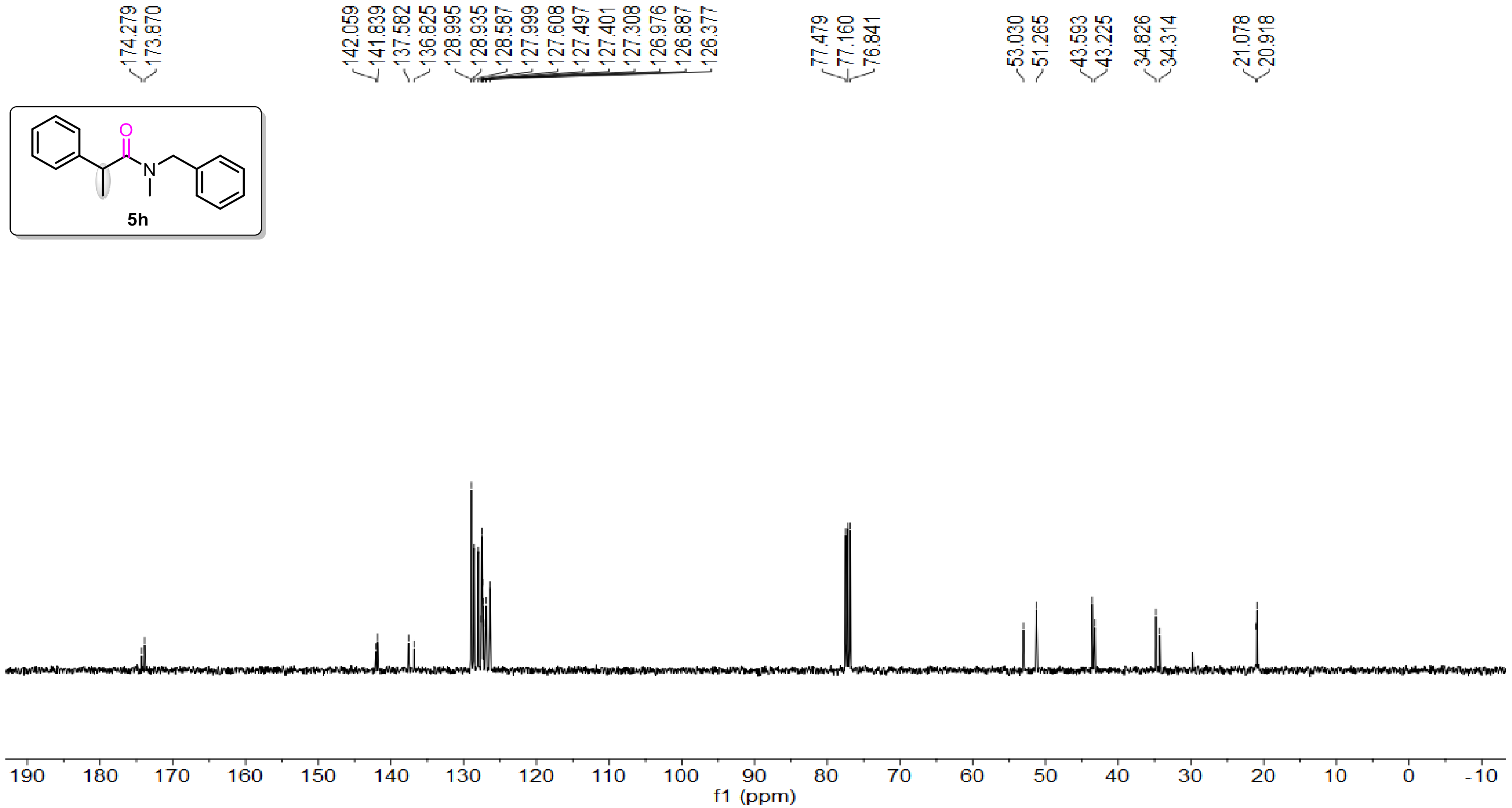

Supplementary Fig. 156. ^{1}H NMR Spectra (400 MHz, $CDCl_3$) of **5i**

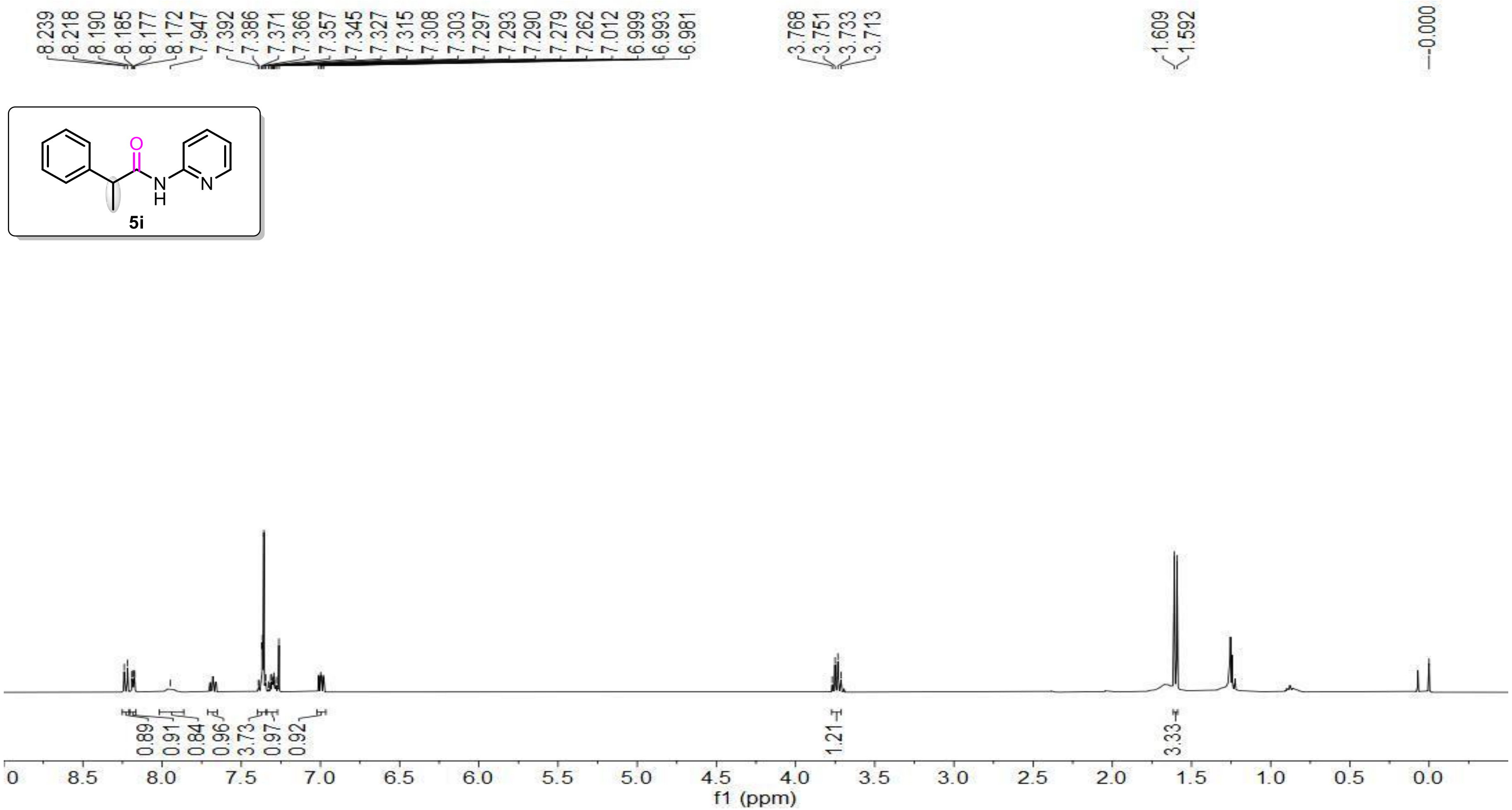

Supplementary Fig. 157. ^{13}C NMR Spectra (101 MHz, $CDCl_3$) of **5i**

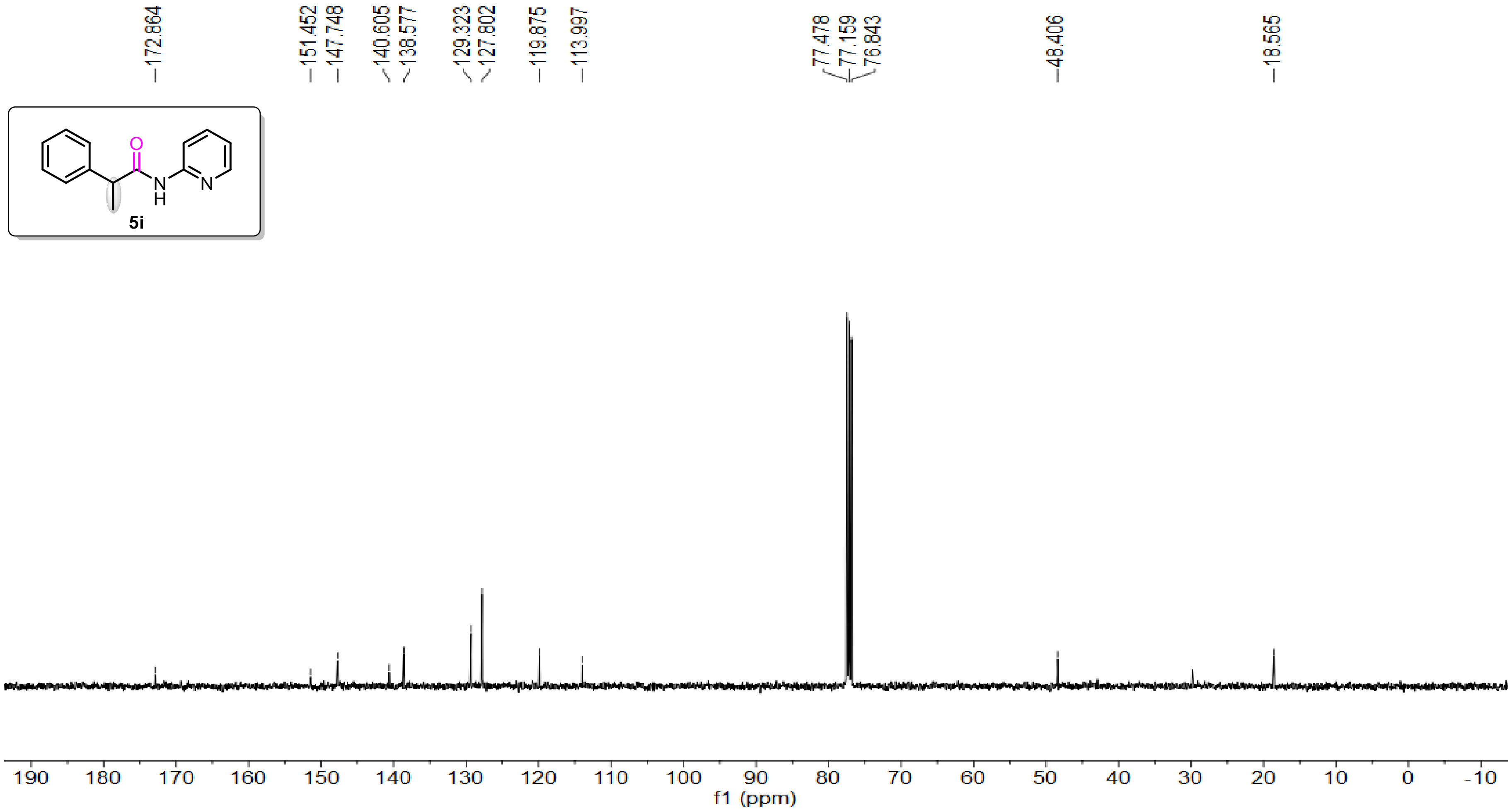

Supplementary Fig. 158. ^{1}H NMR Spectra (400 MHz, $CDCl_3$) of **5j**

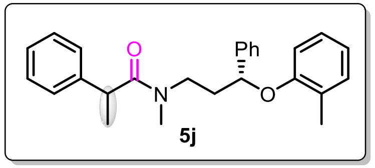

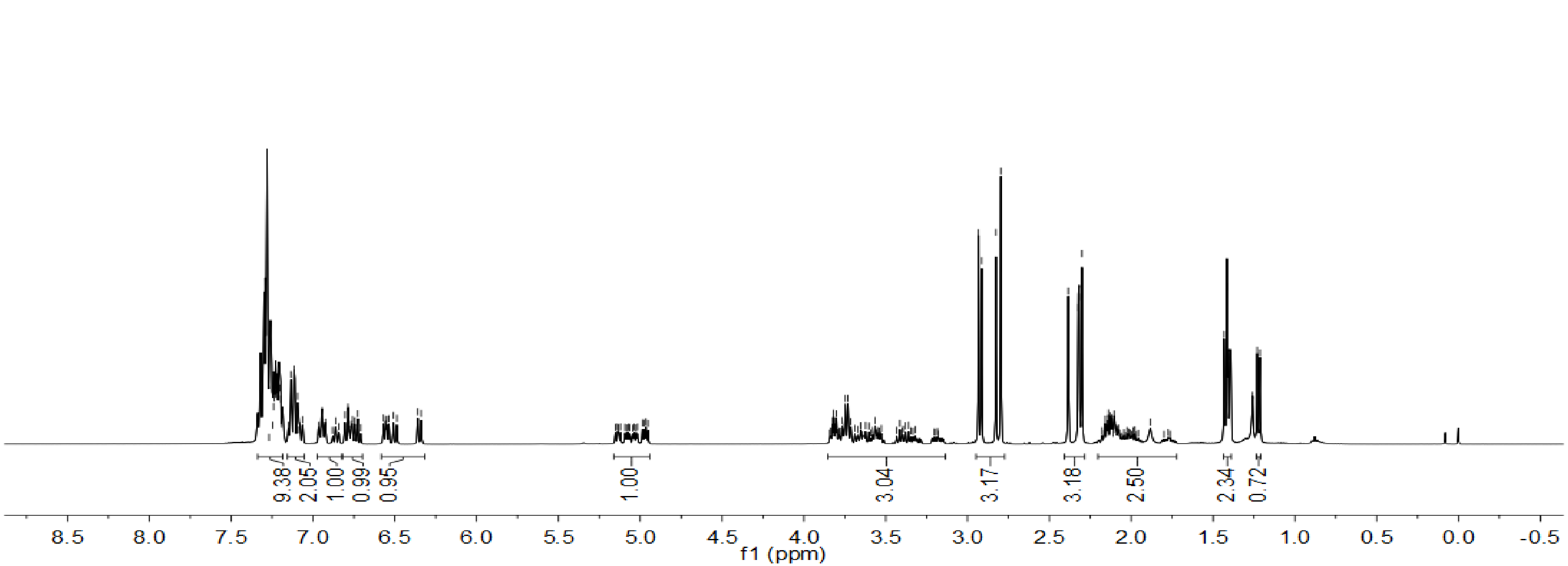

Supplementary Fig. 159. ^{13}C NMR Spectra (101 MHz, $CDCl_3$) of **5j**

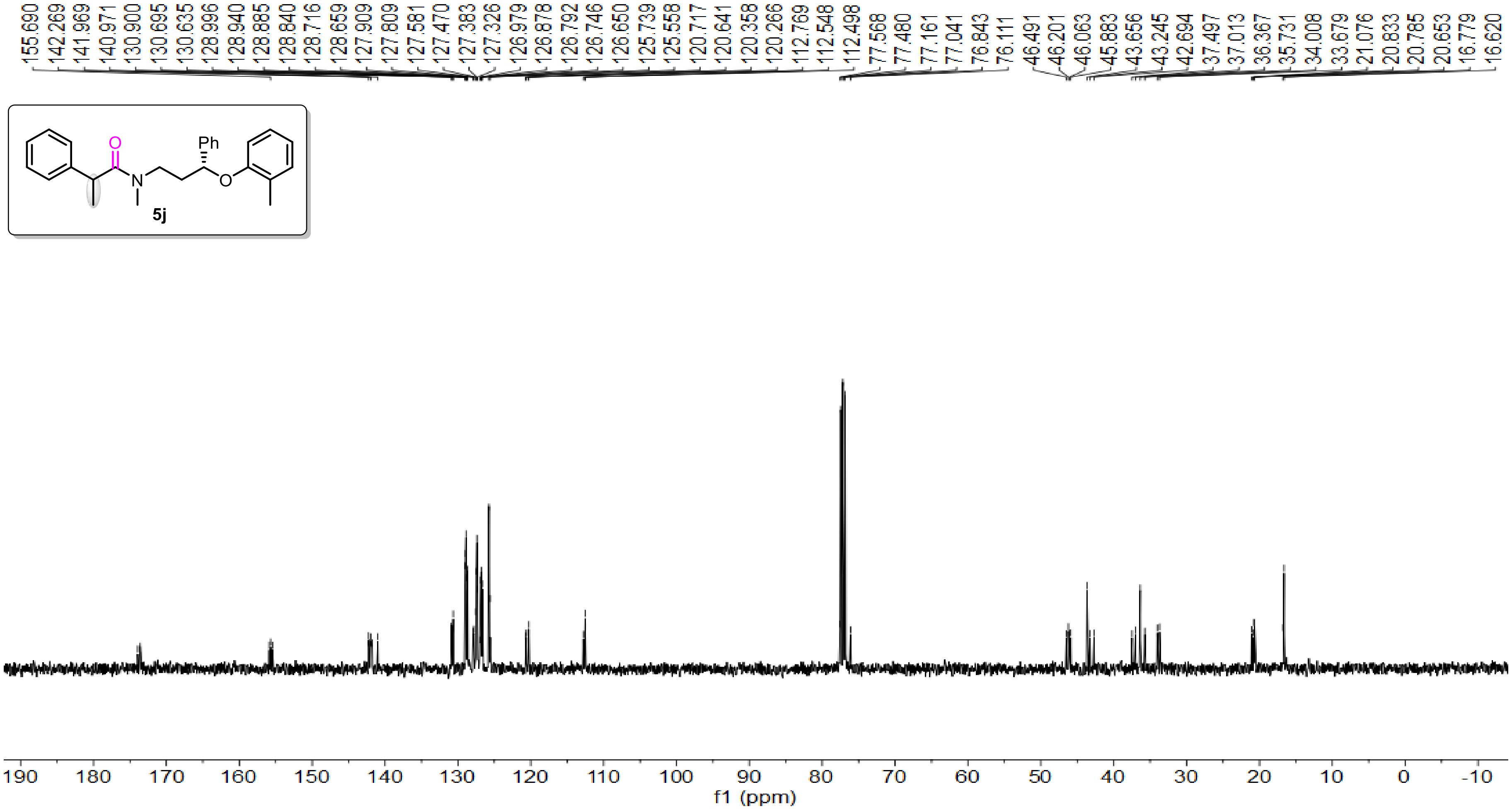

Supplementary Fig. 160. ^{1}H NMR Spectra (400 MHz, $CDCl_3$) of **5k**

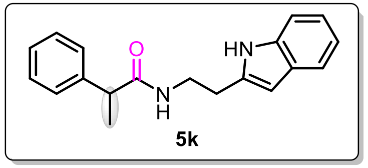

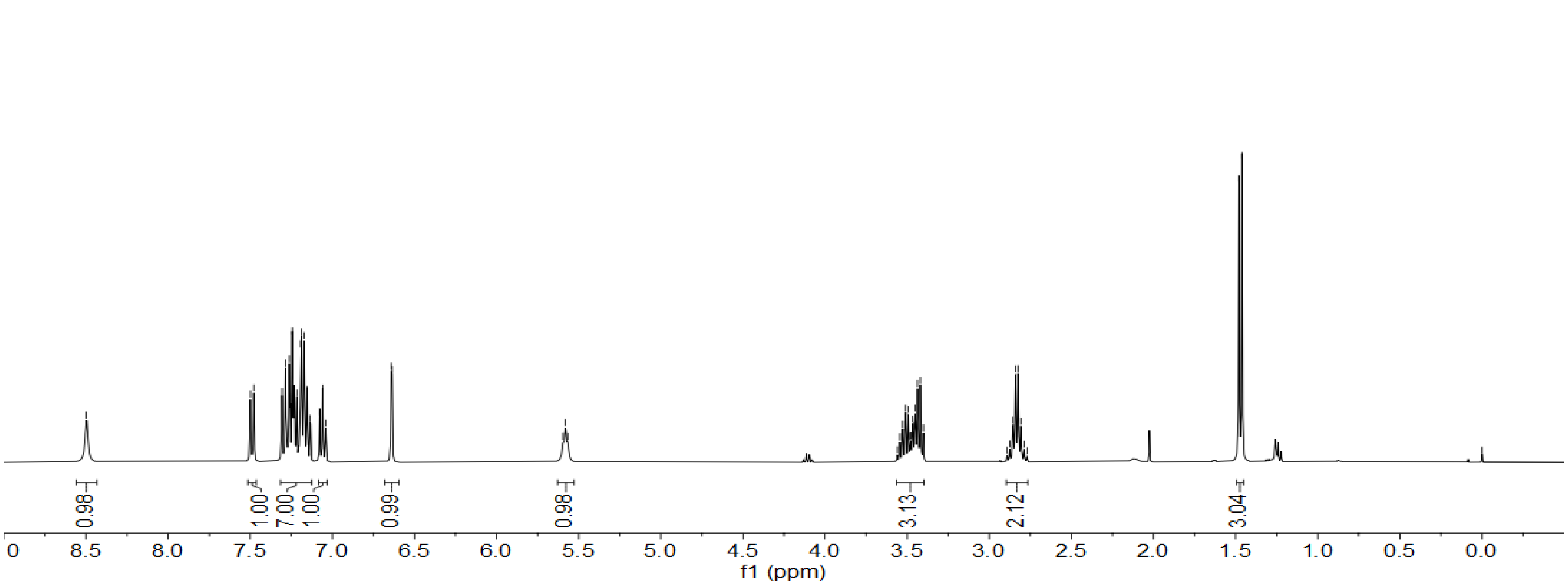

Supplementary Fig. 161. ^{13}C NMR Spectra (101 MHz, $CDCl_3$) of **5k**

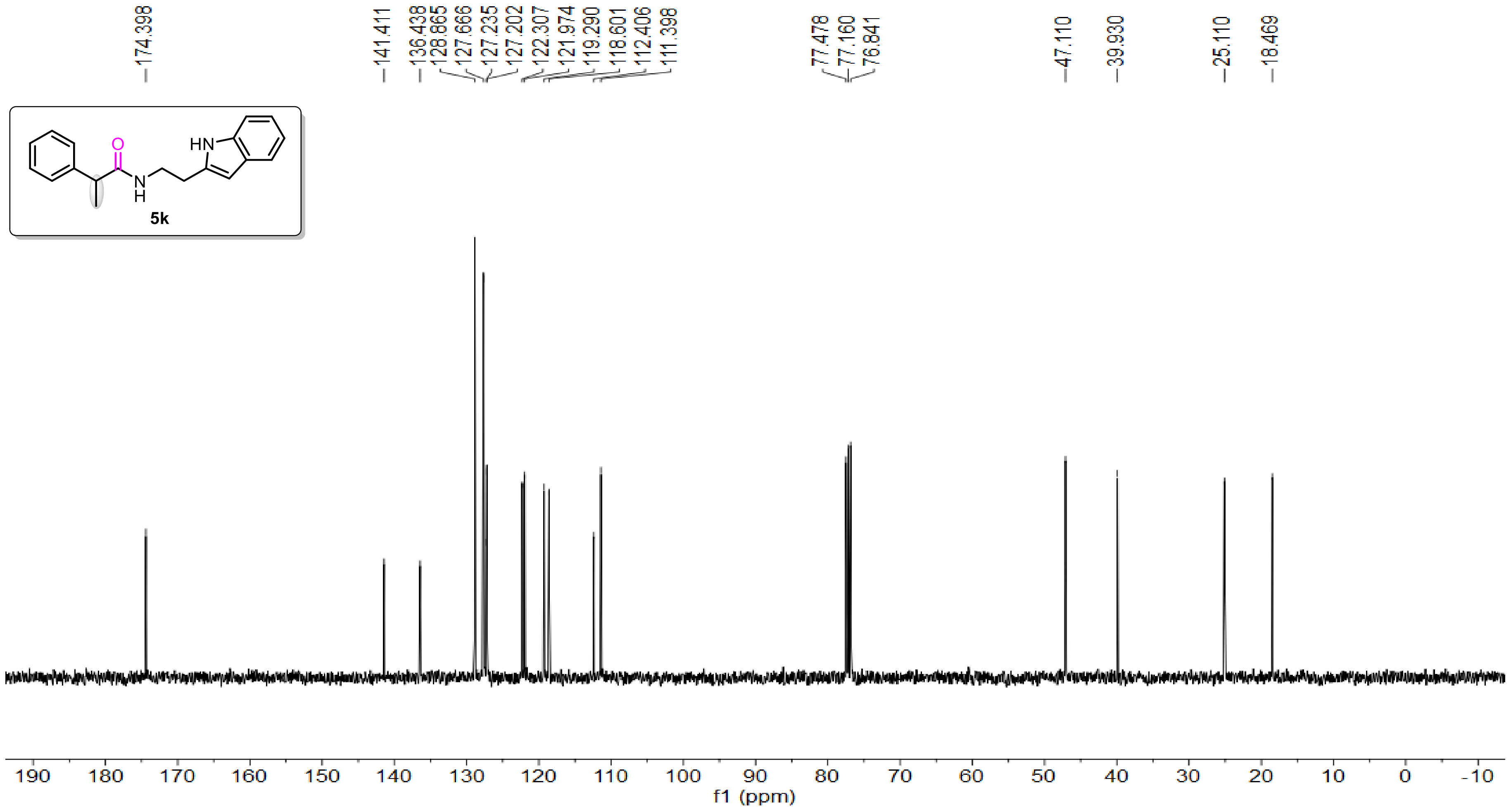

Supplementary Fig. 162. ^{1}H NMR Spectra (400 MHz, $CDCl_3$) of **5l**

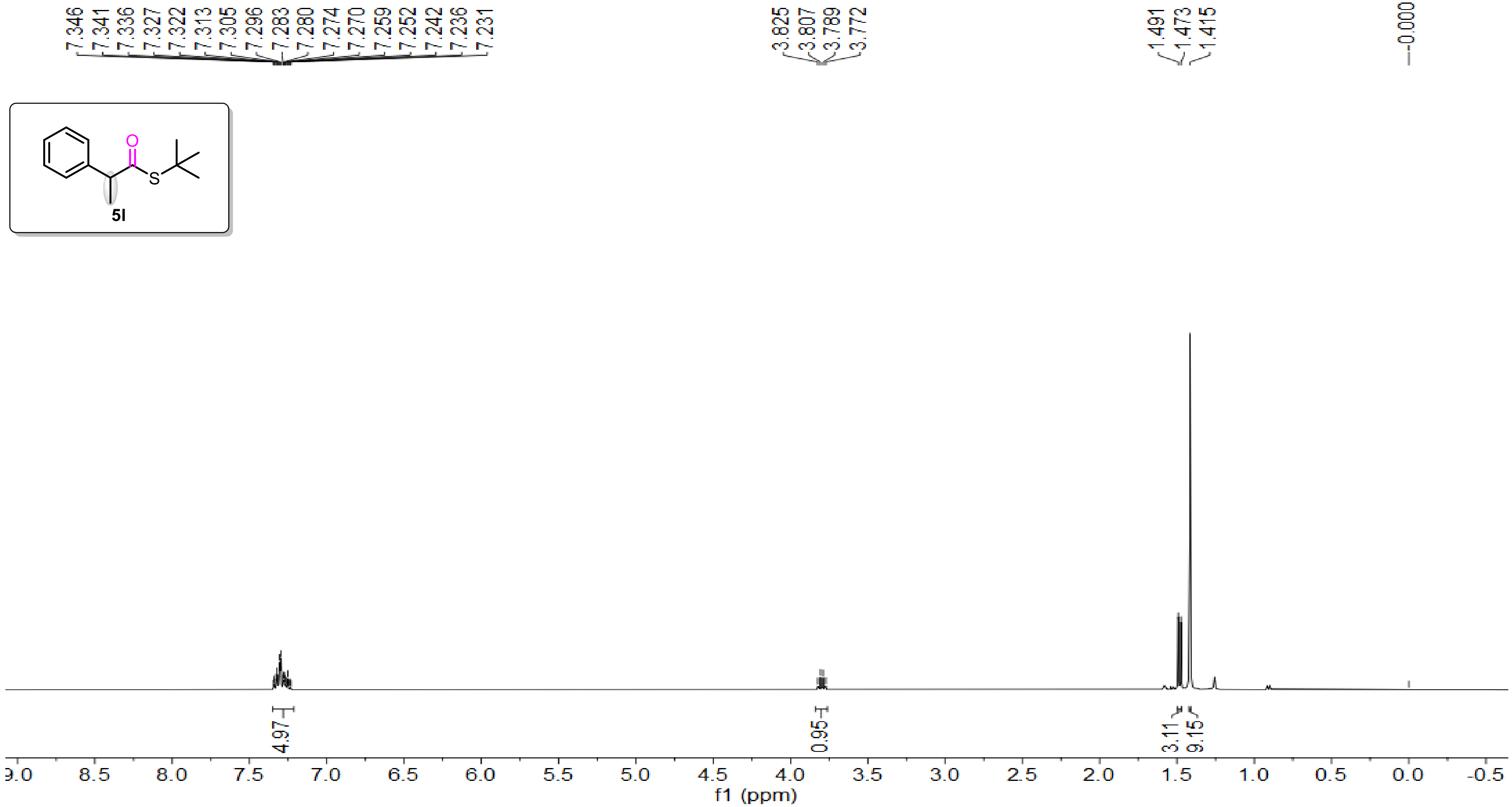

Supplementary Fig. 163. ^{13}C NMR Spectra (101 MHz, $CDCl_3$) of **5l**

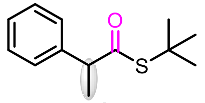

5l

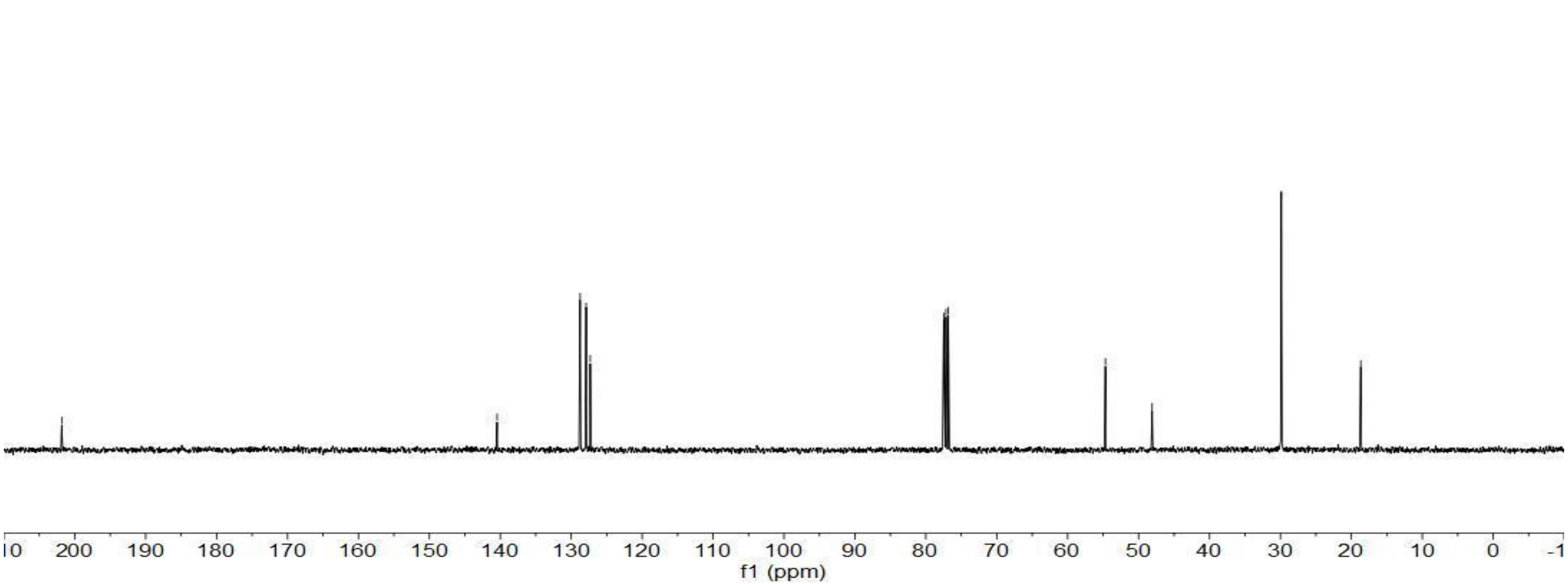

Supplementary Fig. 164. ^{1}H NMR Spectra (400 MHz, $CDCl_3$) of **5m**

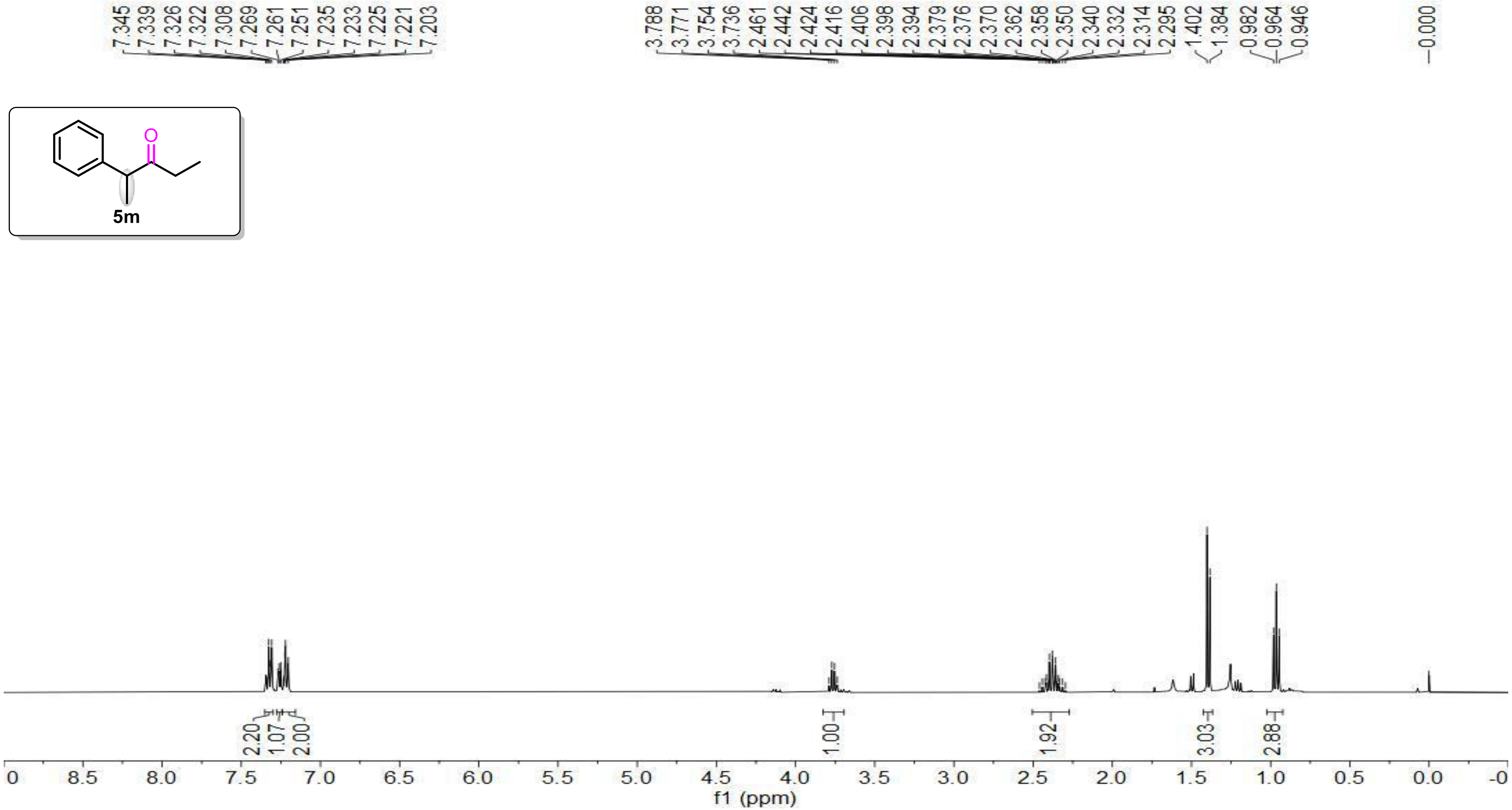

Supplementary Fig. 165. ^{13}C NMR Spectra (101 MHz, $CDCl_3$) of **5m**

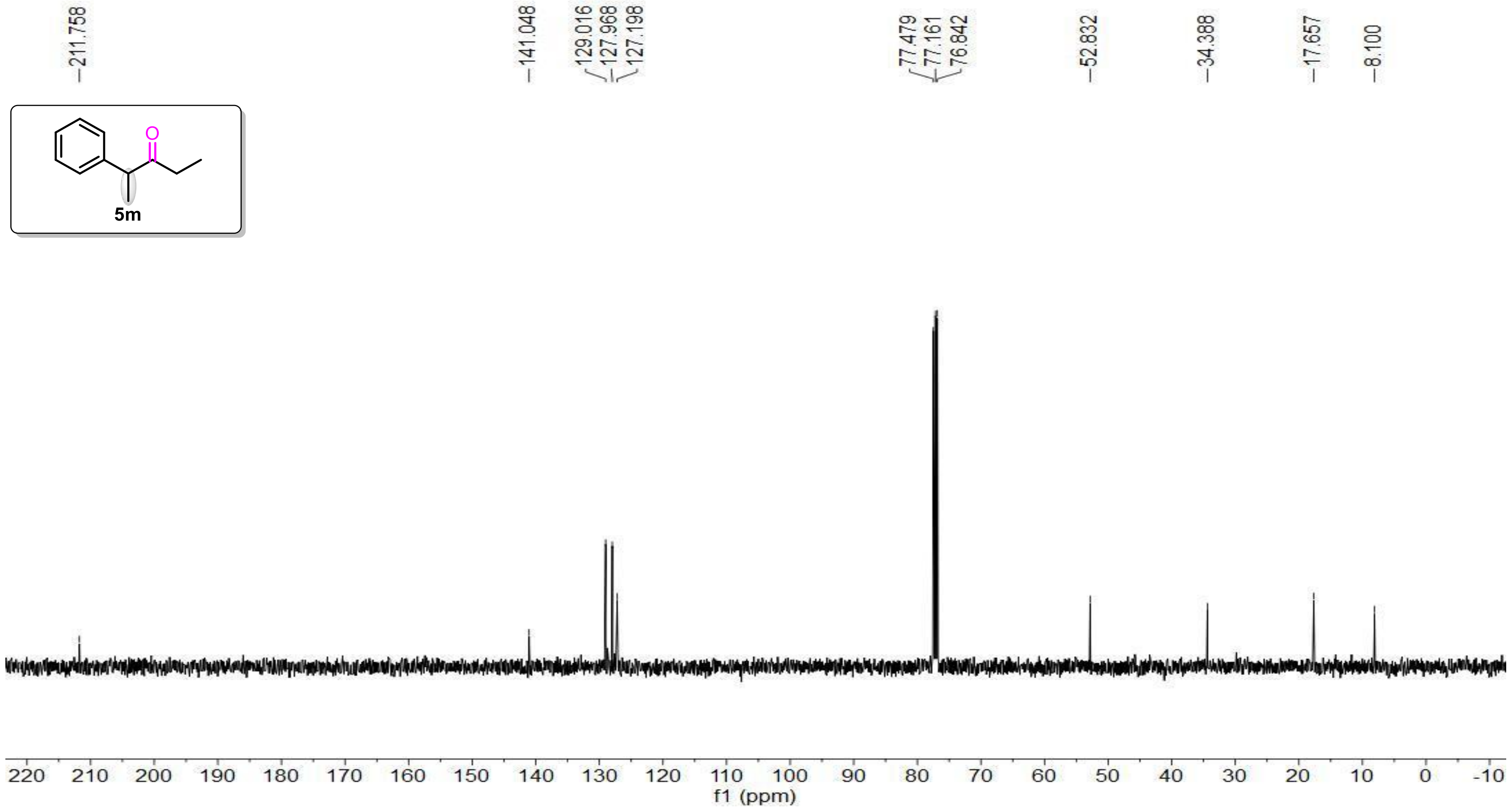

Supplementary Fig. 166. ^{1}H NMR Spectra (400 MHz, $CDCl_3$) of **5n**

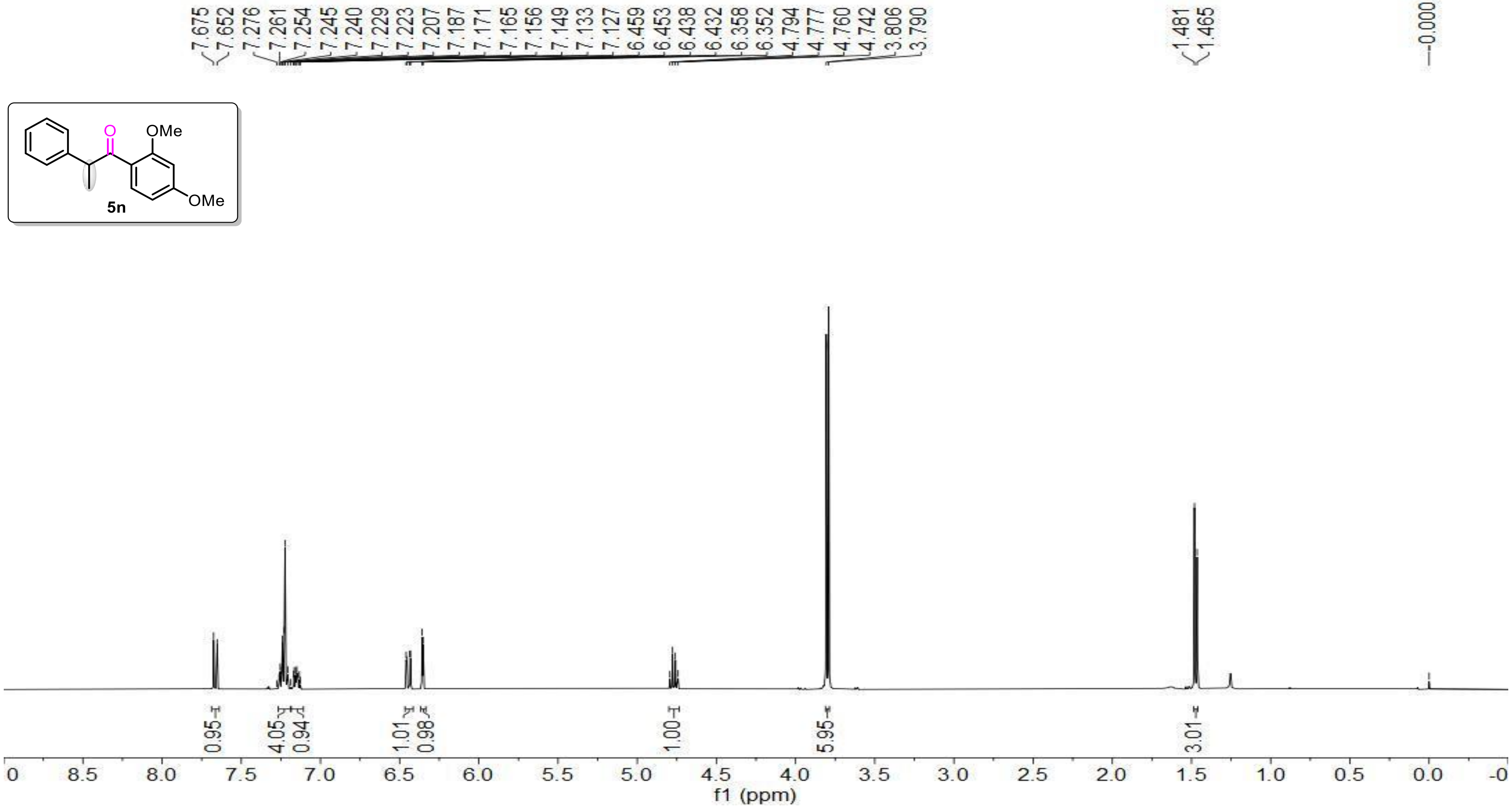

Supplementary Fig. 167. ^{13}C NMR Spectra (101 MHz, $CDCl_3$) of **5n**

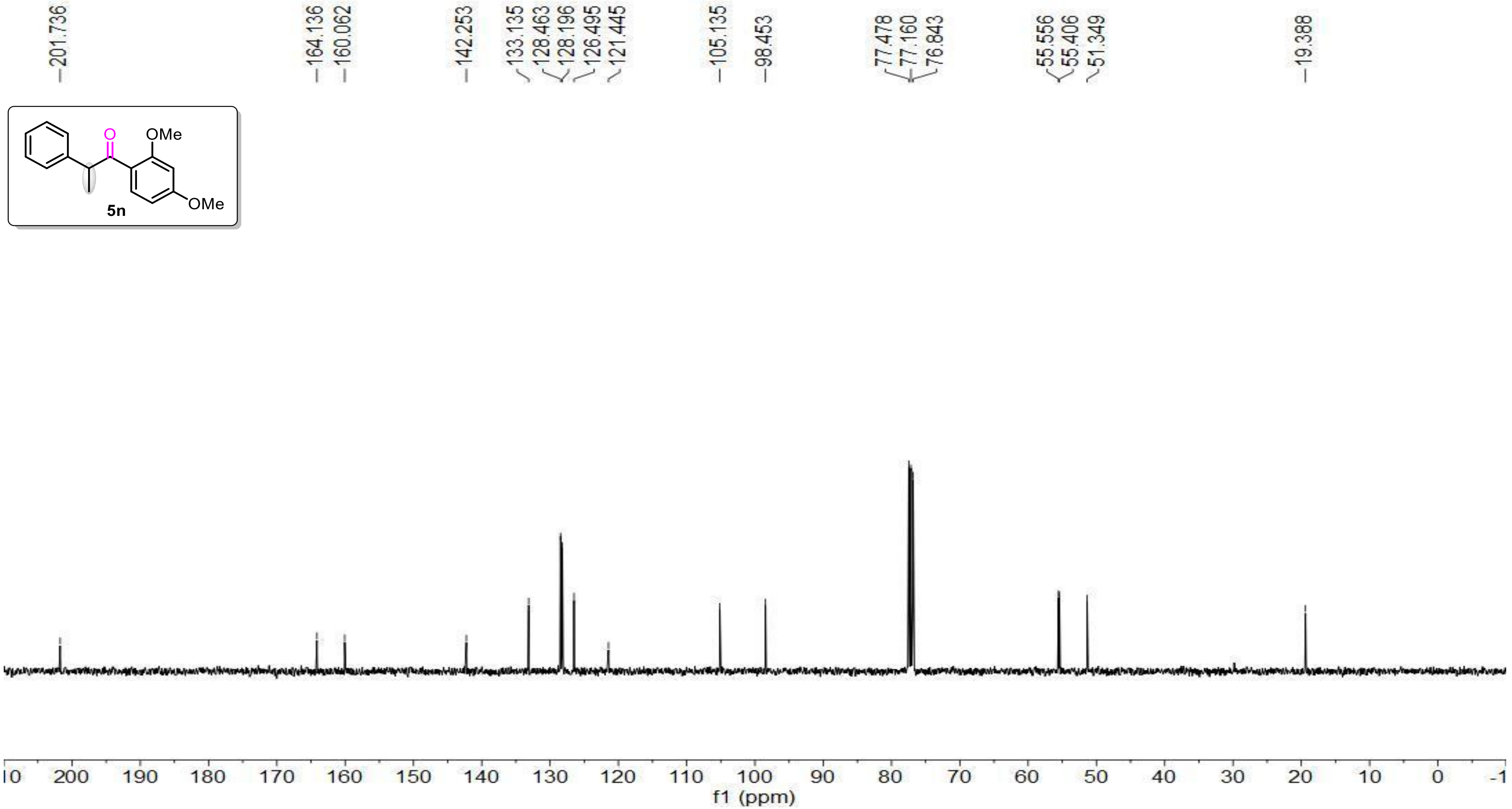

Supplementary Fig. 168. ^{1}H NMR Spectra (400 MHz, $CDCl_3$) of **6a**

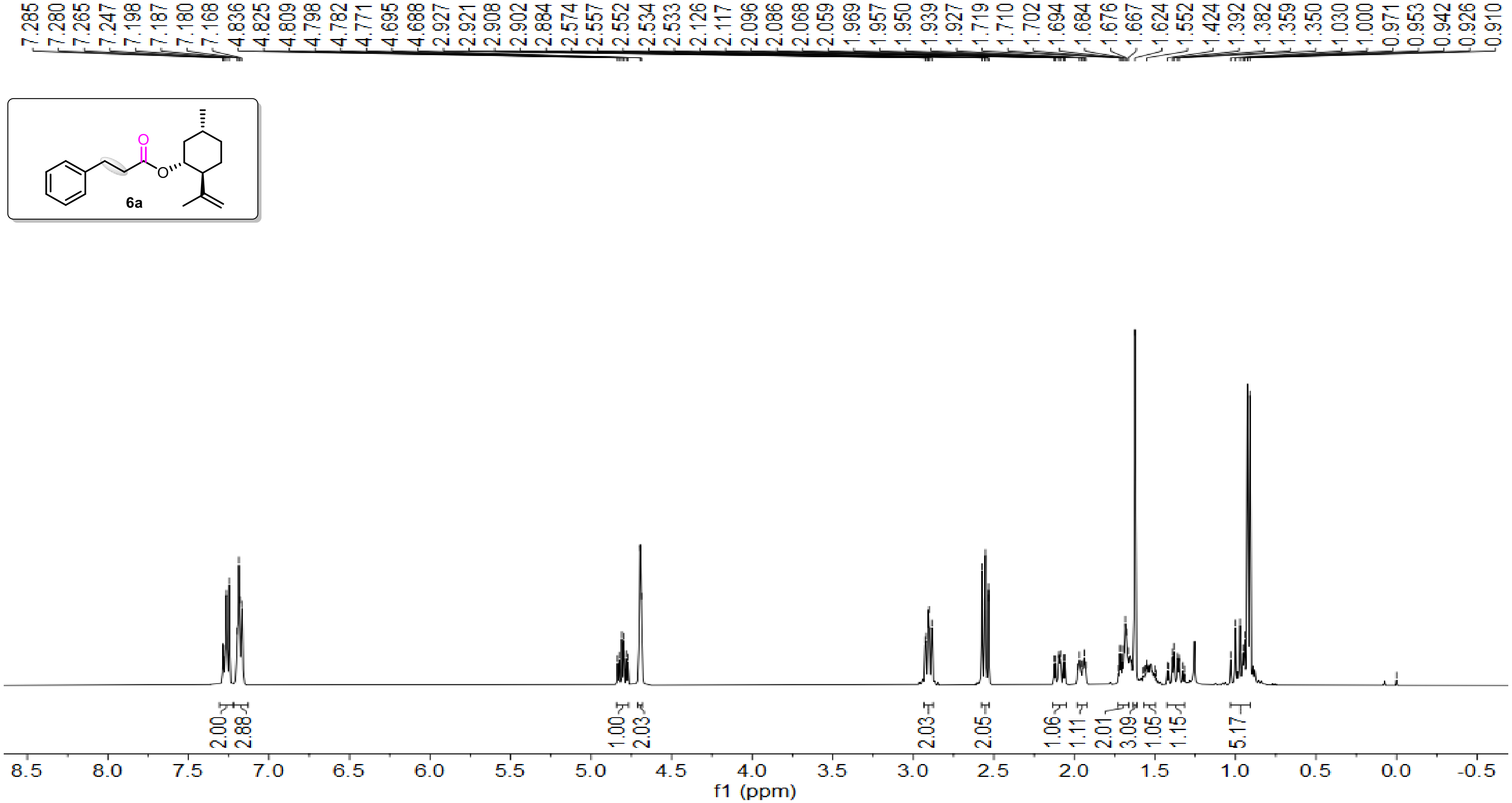

Supplementary Fig. 169. ^{13}C NMR Spectra (101 MHz, $CDCl_3$) of **6a**

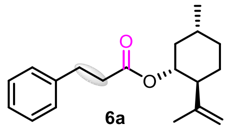

6a

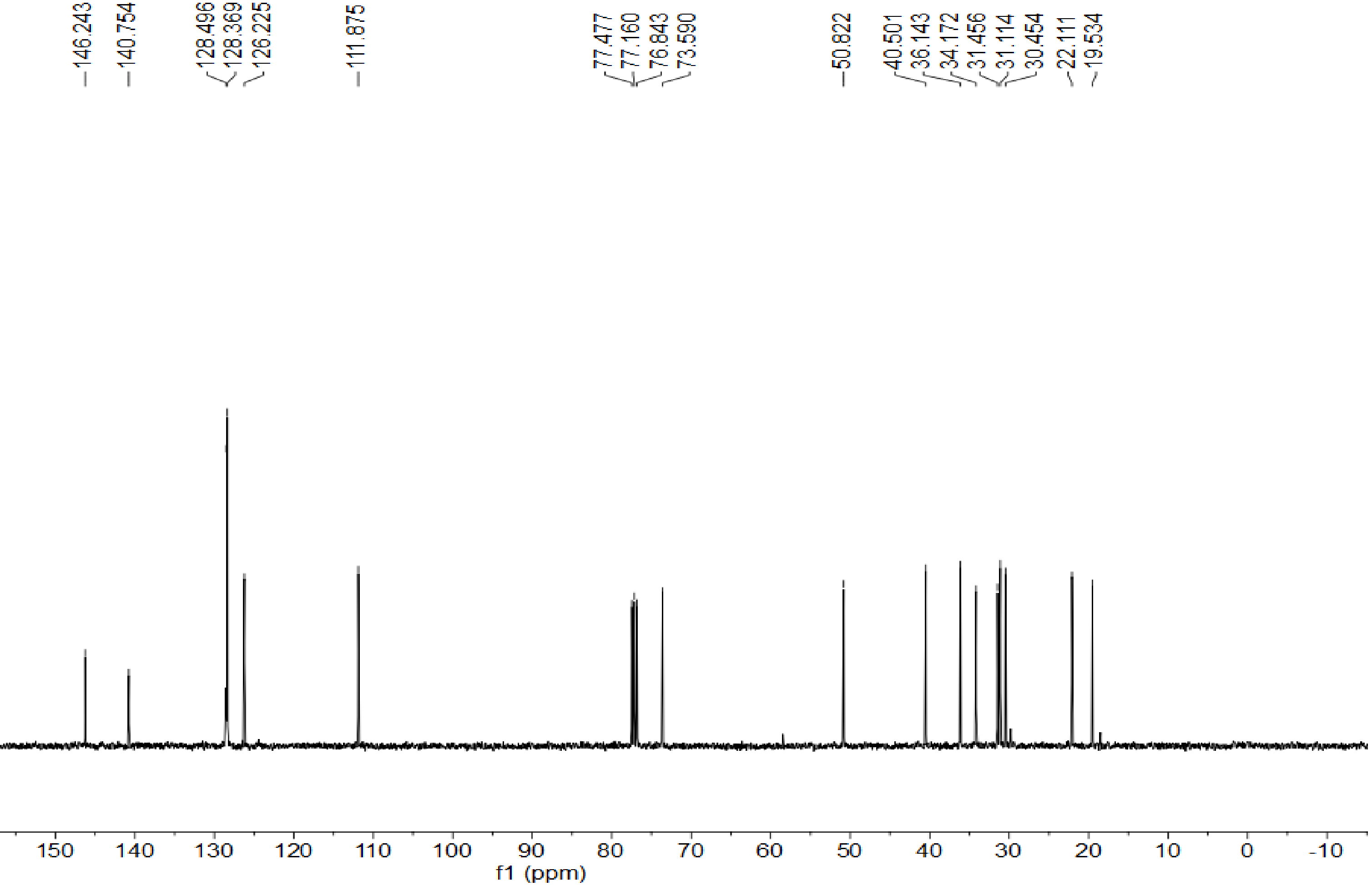

Supplementary Fig. 170. ^{1}H NMR Spectra (400 MHz, $CDCl_3$) of **6b**

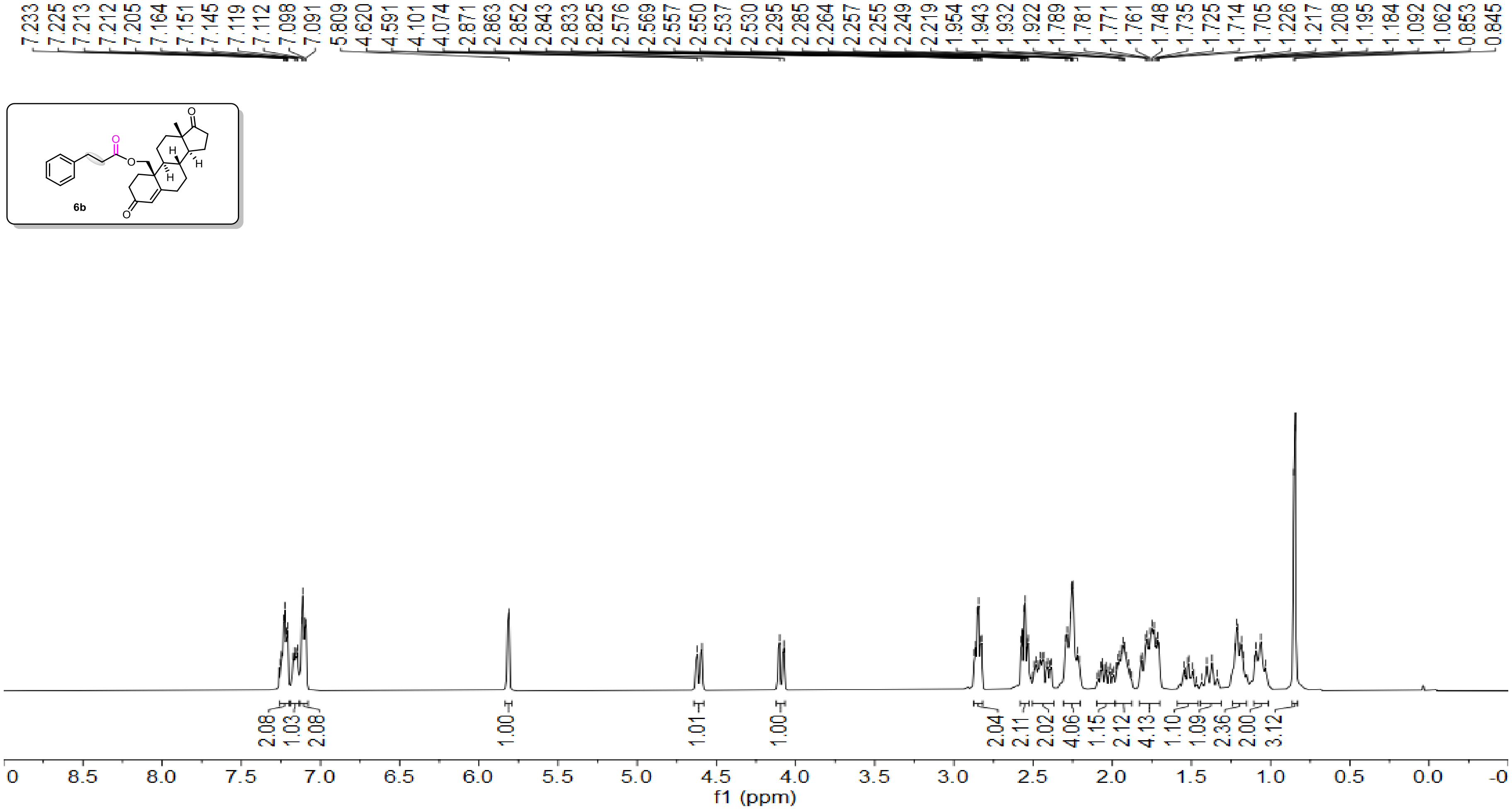

Supplementary Fig. 171. ^{13}C NMR Spectra (101 MHz, $CDCl_3$) of **6b**

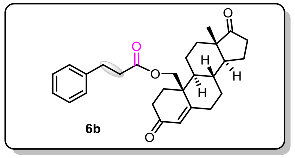

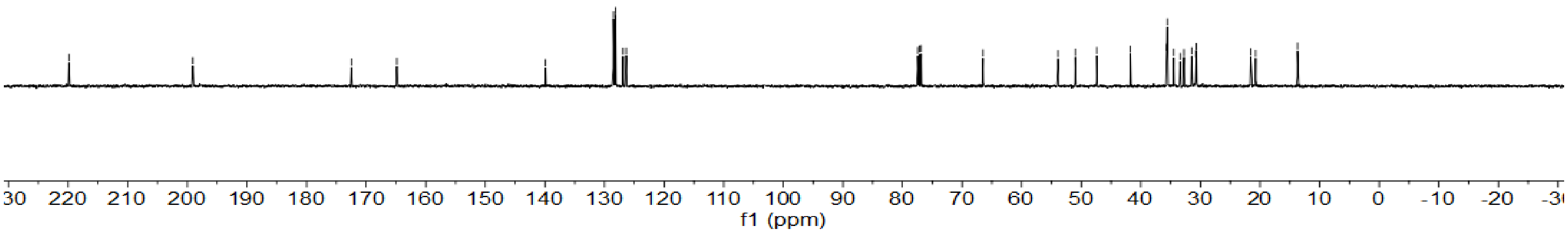

Supplementary Fig. 172. ^{1}H NMR Spectra (400 MHz, $CDCl_3$) of **6c**

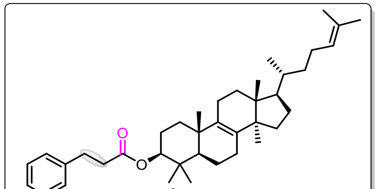

6c

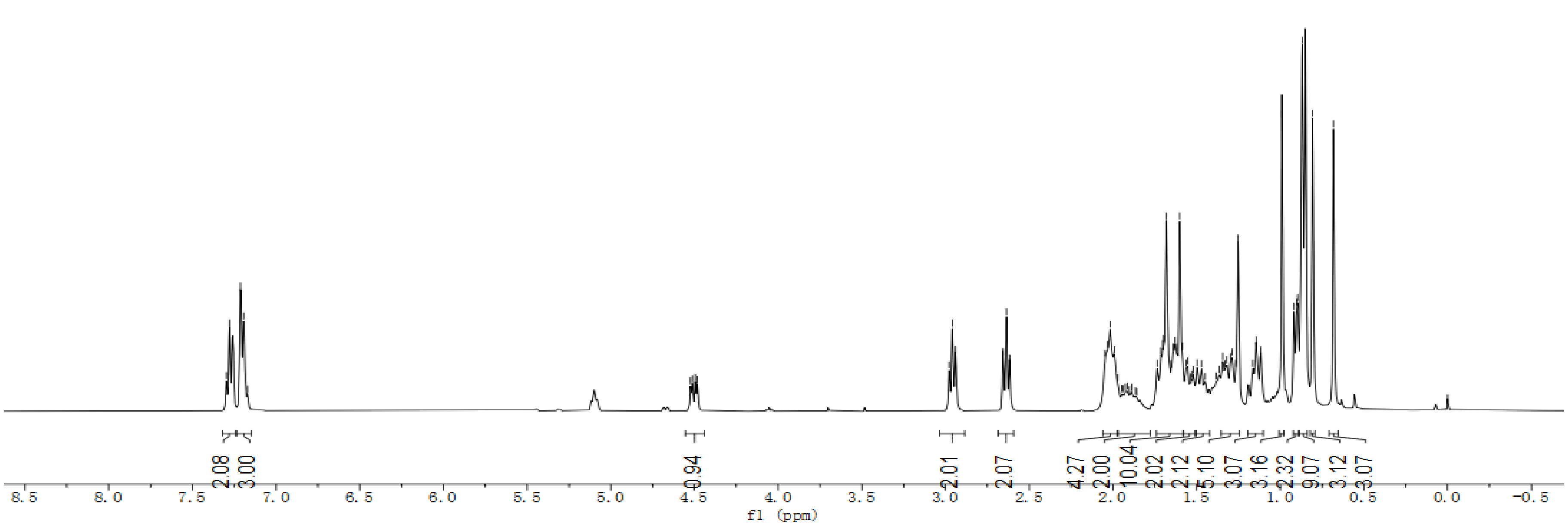

Supplementary Fig. 173. ^{13}C NMR Spectra (101 MHz, $CDCl_3$) of **6c**

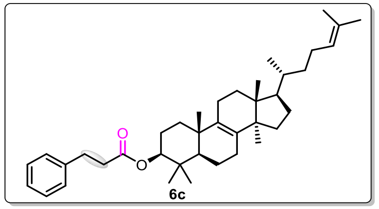

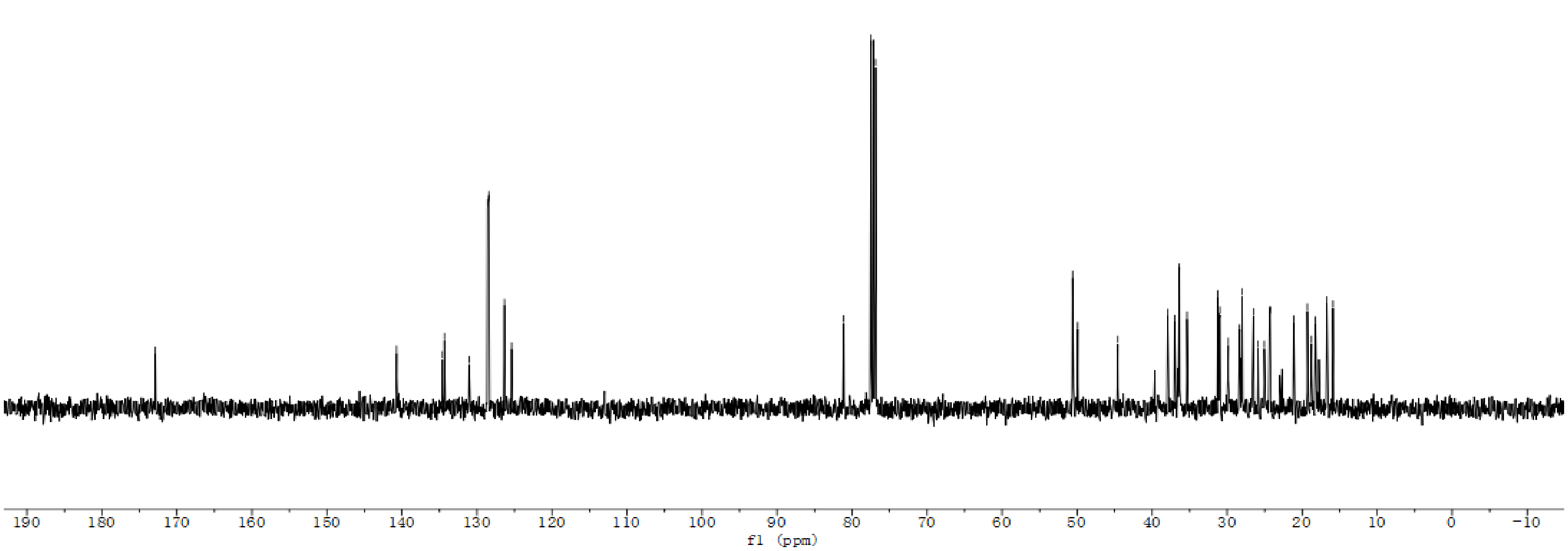

Supplementary Fig. 174. ^{1}H NMR Spectra (400 MHz, $CDCl_3$) of **6d**

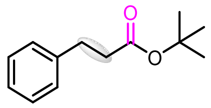
6d

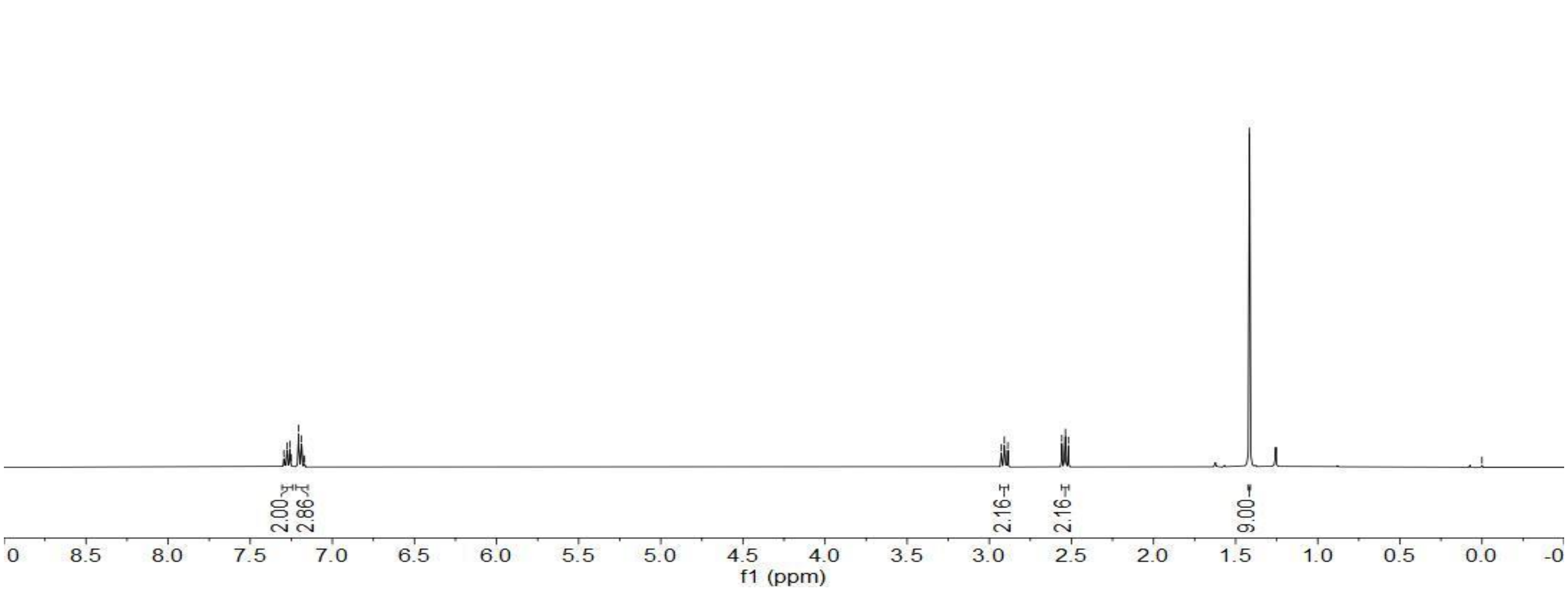

Supplementary Fig. 175. ^{13}C NMR Spectra (101 MHz, $CDCl_3$) of **6d**

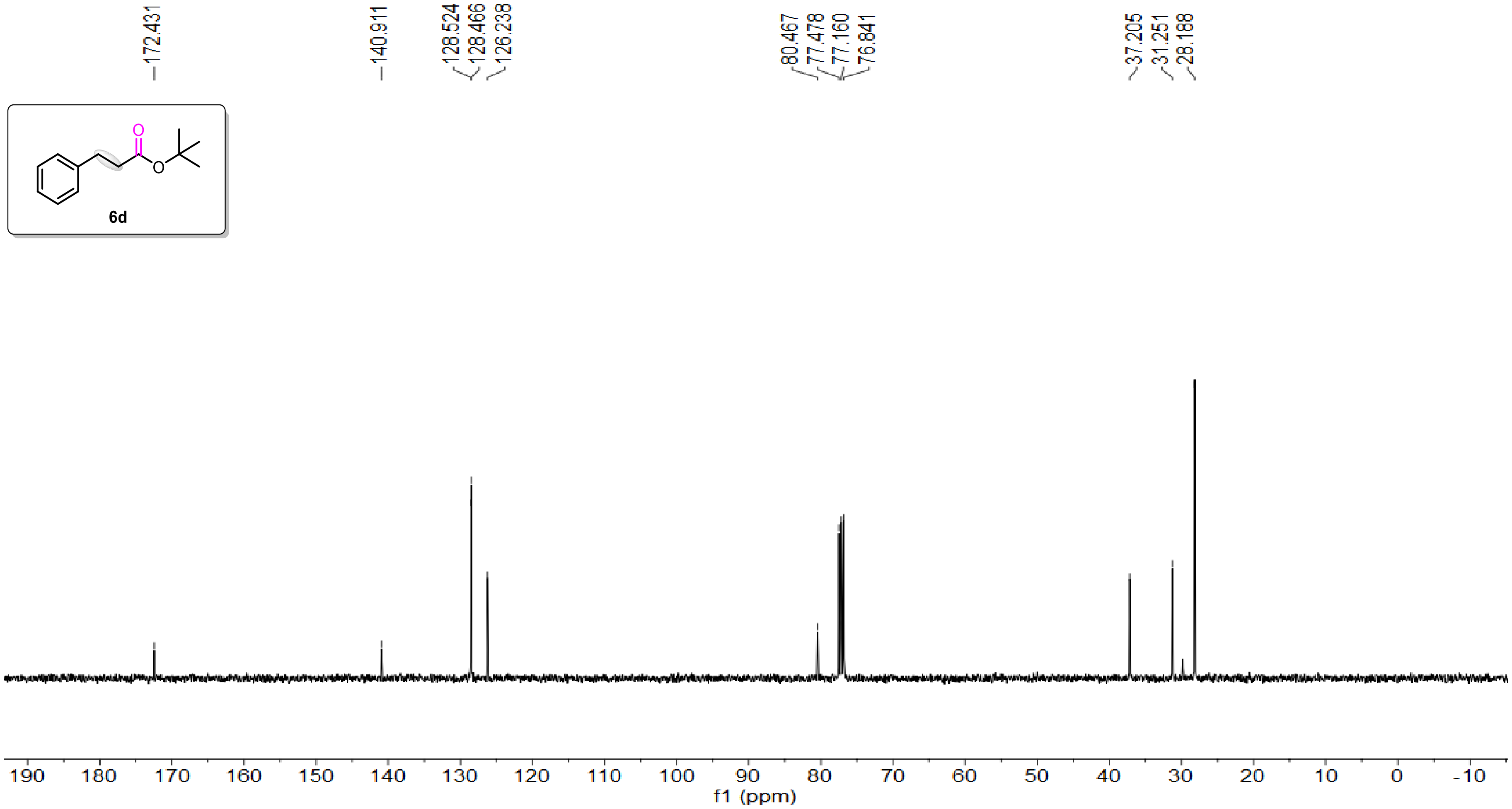

Supplementary Fig. 176. ^{1}H NMR Spectra (400 MHz, $CDCl_3$) of **6e**

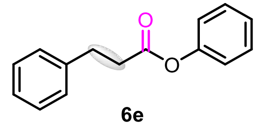

6e

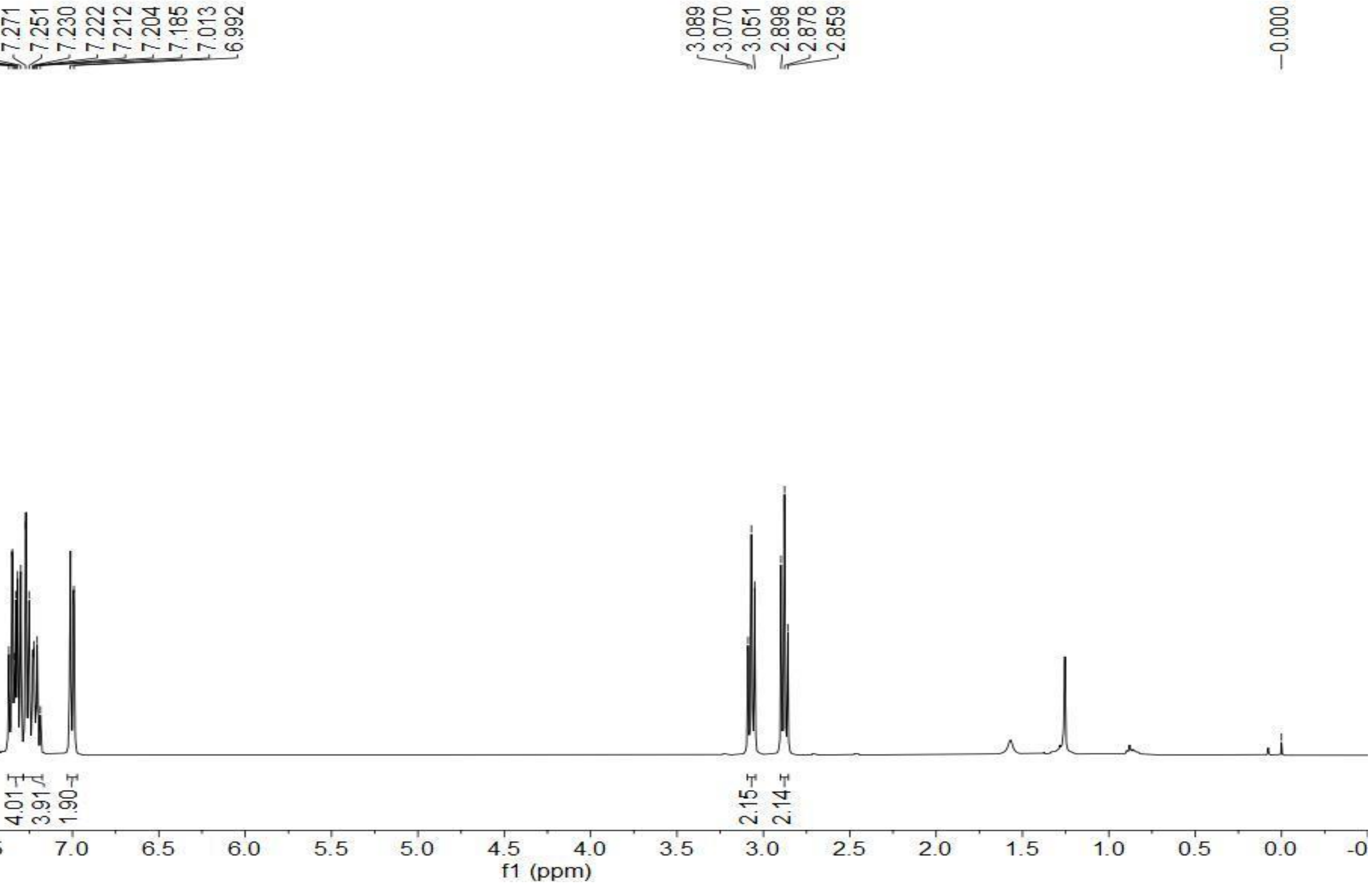

Supplementary Fig. 177. ^{13}C NMR Spectra (101 MHz, $CDCl_3$) of **6e**

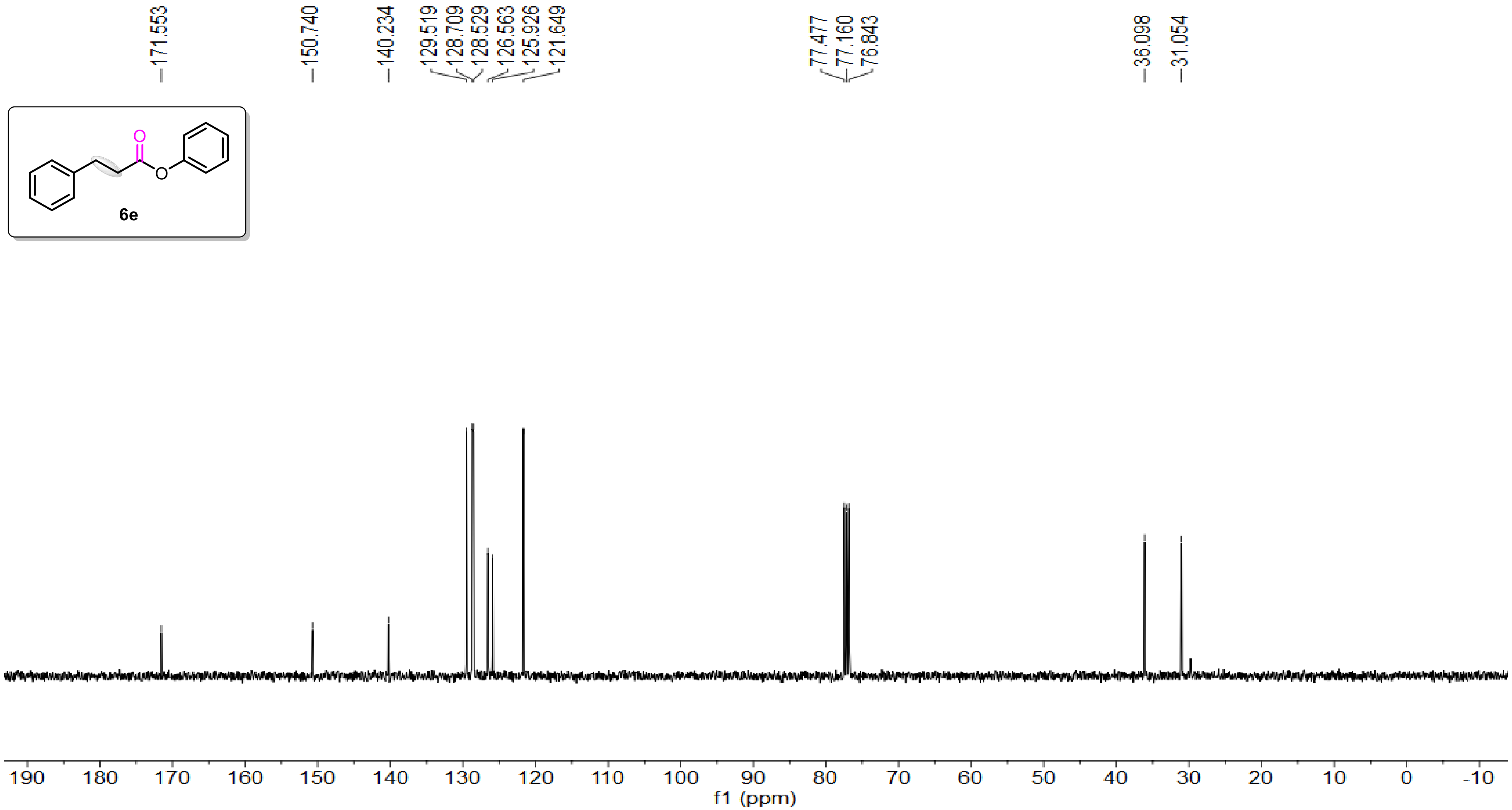

Supplementary Fig. 178. ^{1}H NMR Spectra (400 MHz, $CDCl_3$) of **6f**

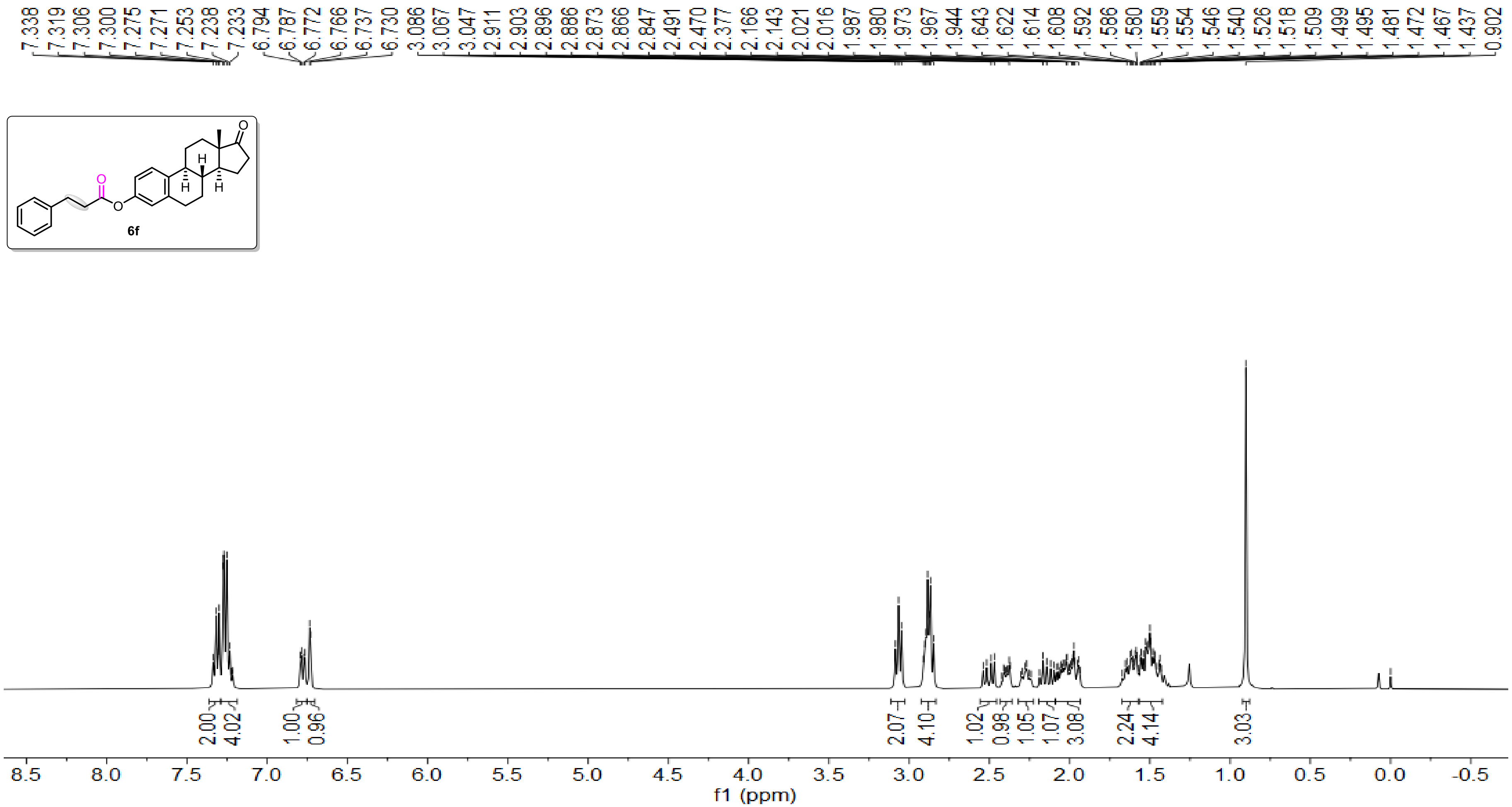

Supplementary Fig. 179. ^{13}C NMR Spectra (101 MHz, $CDCl_3$) of **6f**

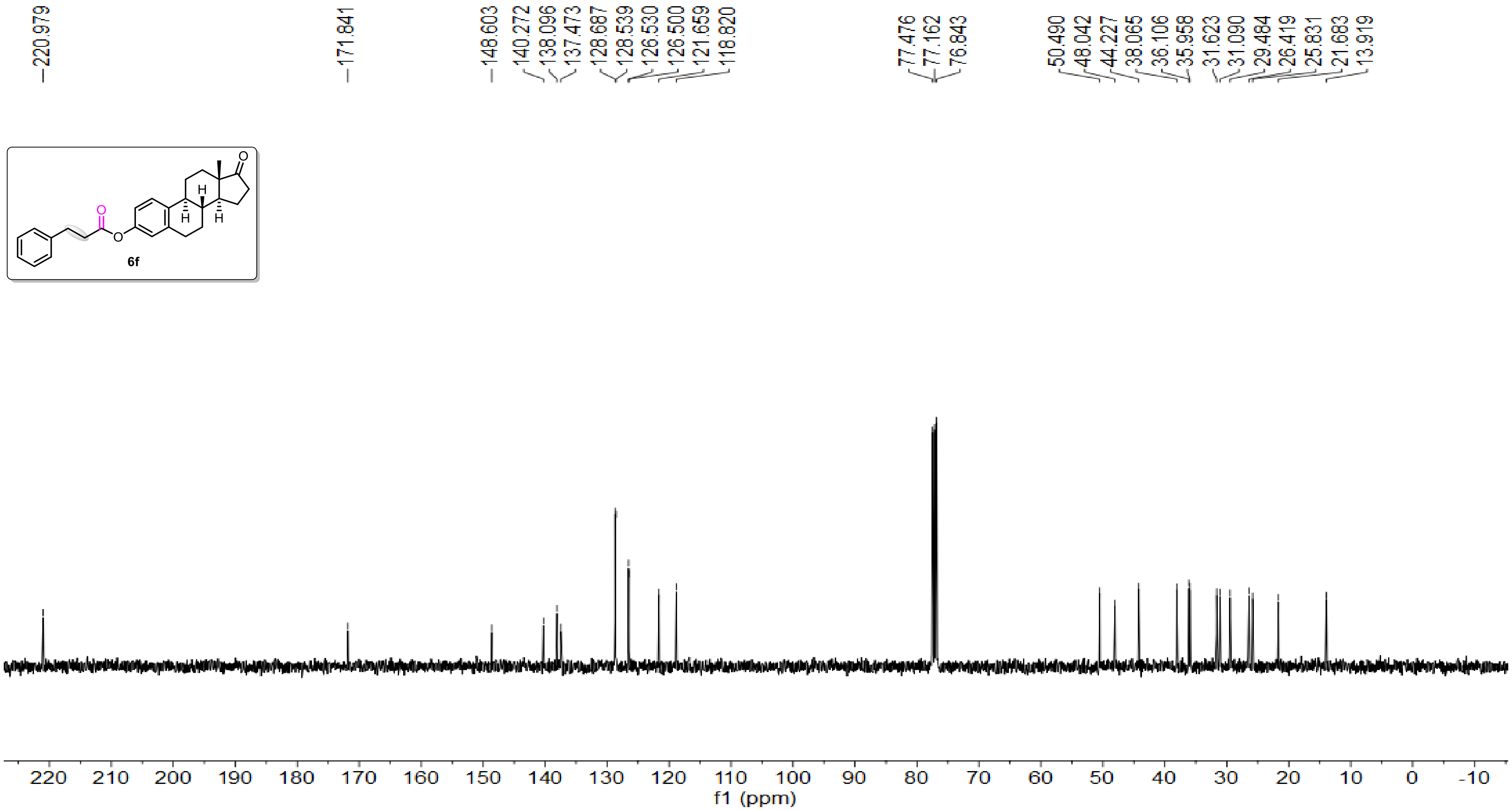

Supplementary Fig. 180. ^{1}H NMR Spectra (400 MHz, $CDCl_3$) of **6g**

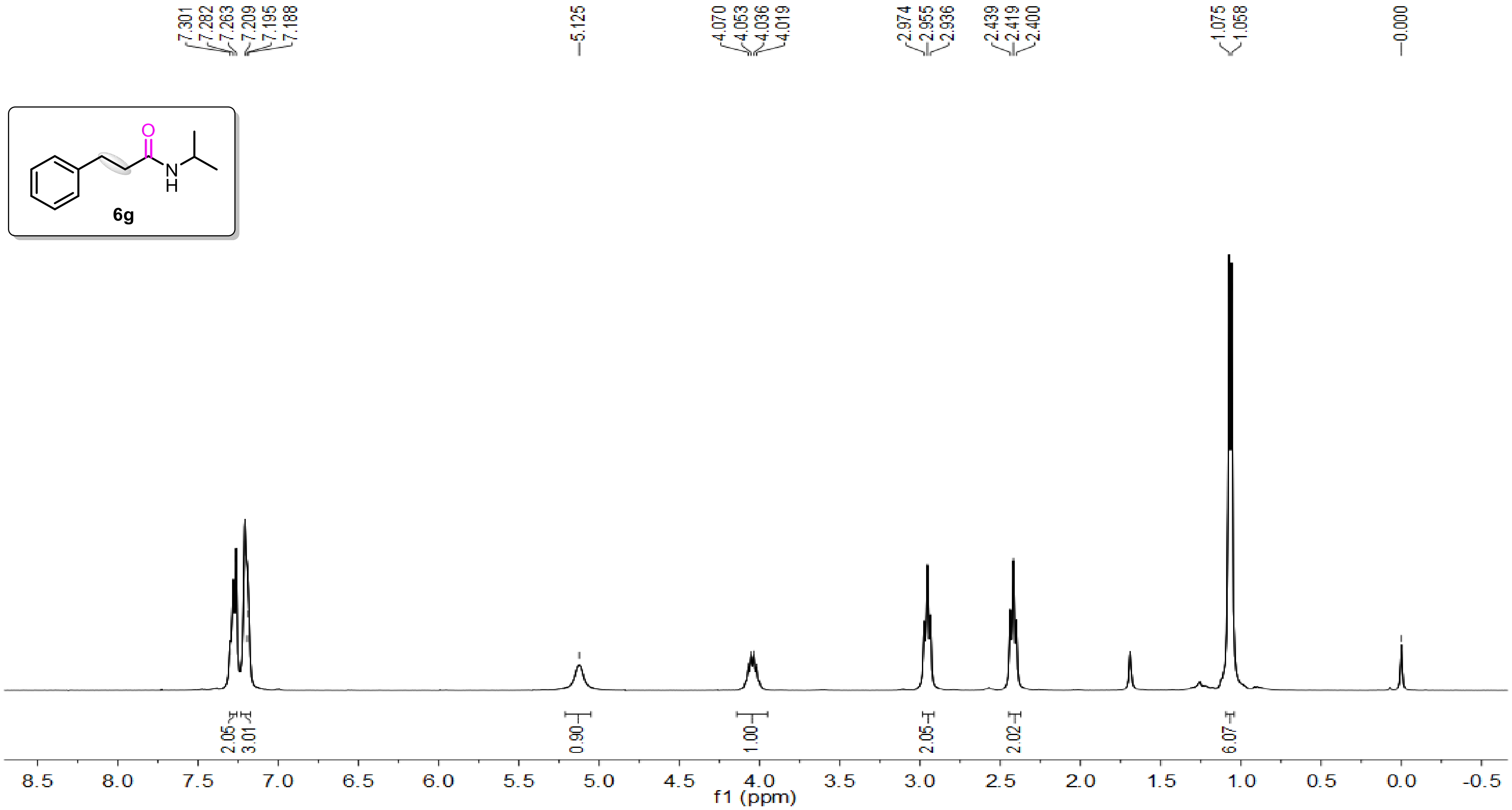

Supplementary Fig. 181. ^{13}C NMR Spectra (101 MHz, $CDCl_3$) of **6g**

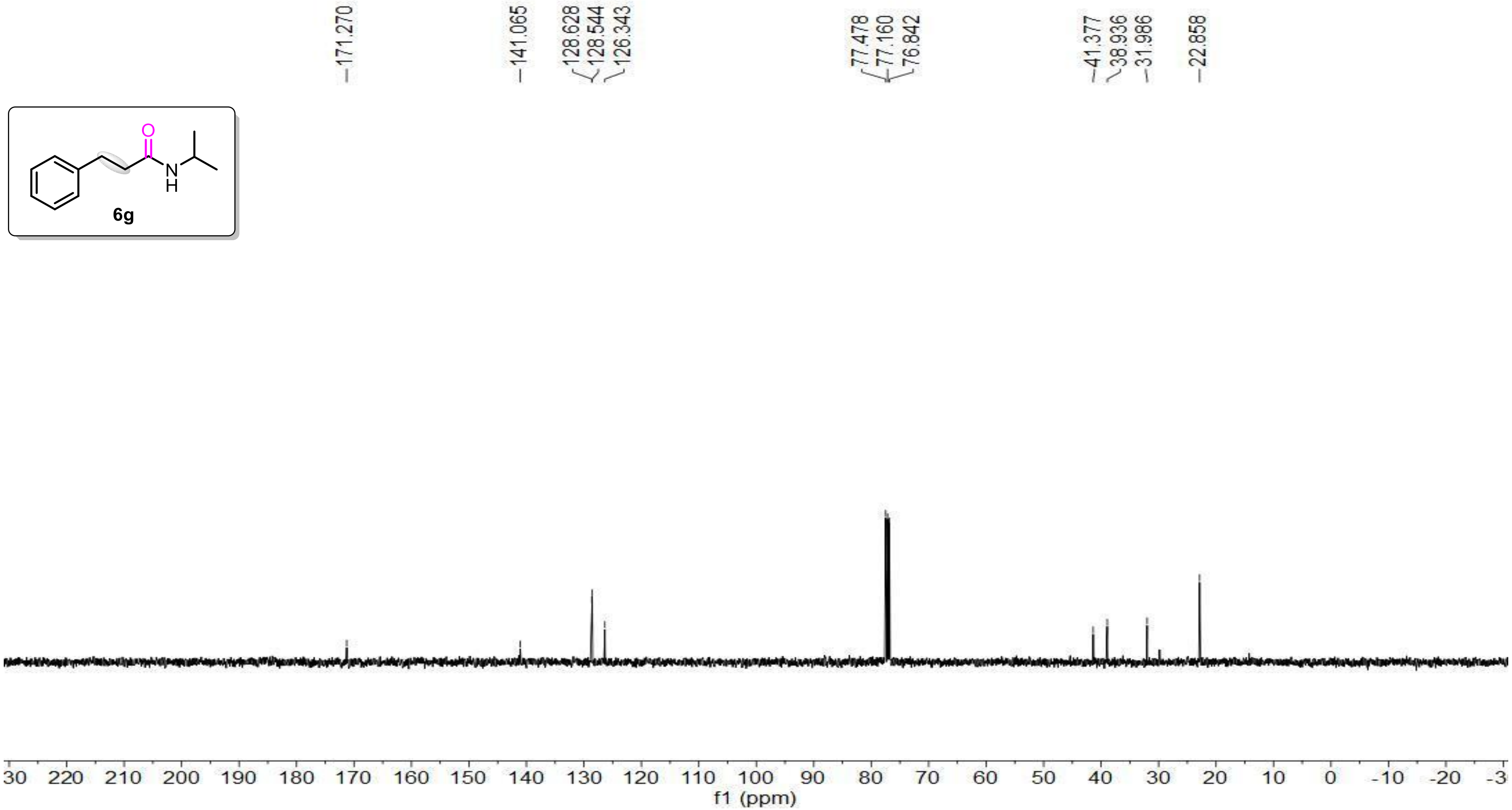

Supplementary Fig. 182. ^{1}H NMR Spectra (400 MHz, $CDCl_3$) of **6h**

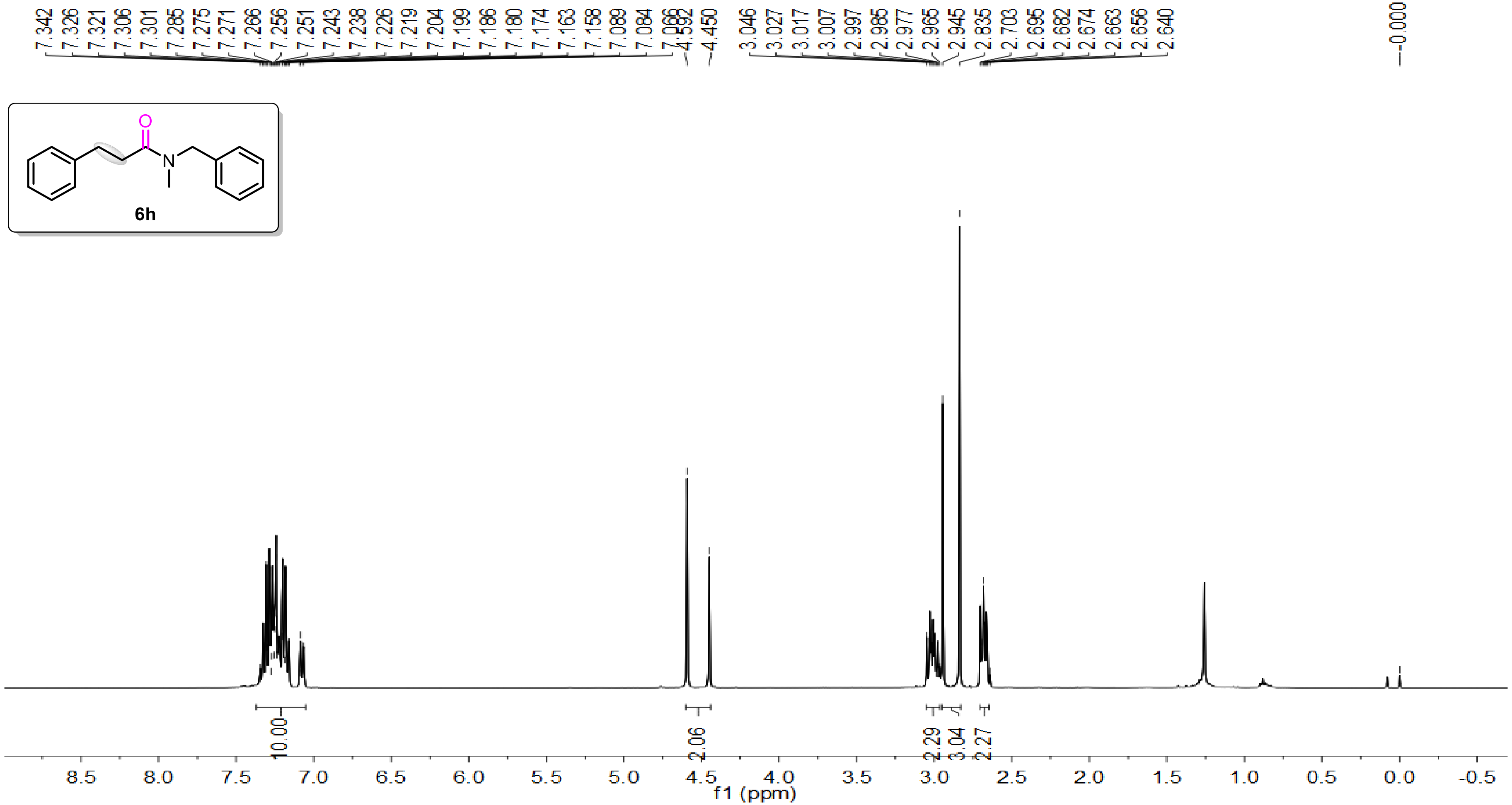

Supplementary Fig. 183. ^{13}C NMR Spectra (101 MHz, $CDCl_3$) of **6h**

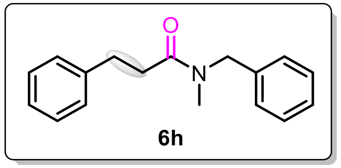

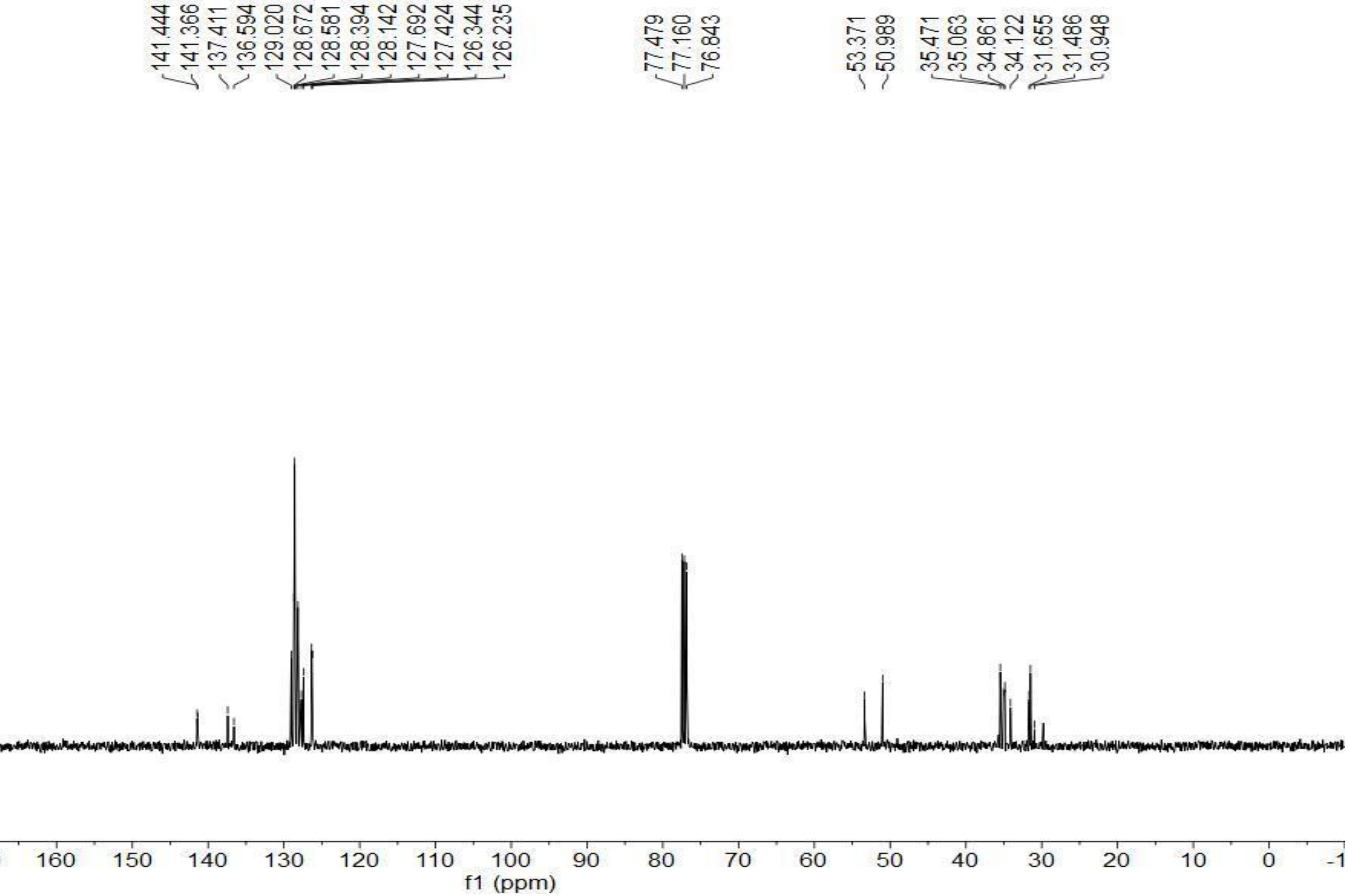

Supplementary Fig. 184. ^{1}H NMR Spectra (400 MHz, $CDCl_3$) of **6i**

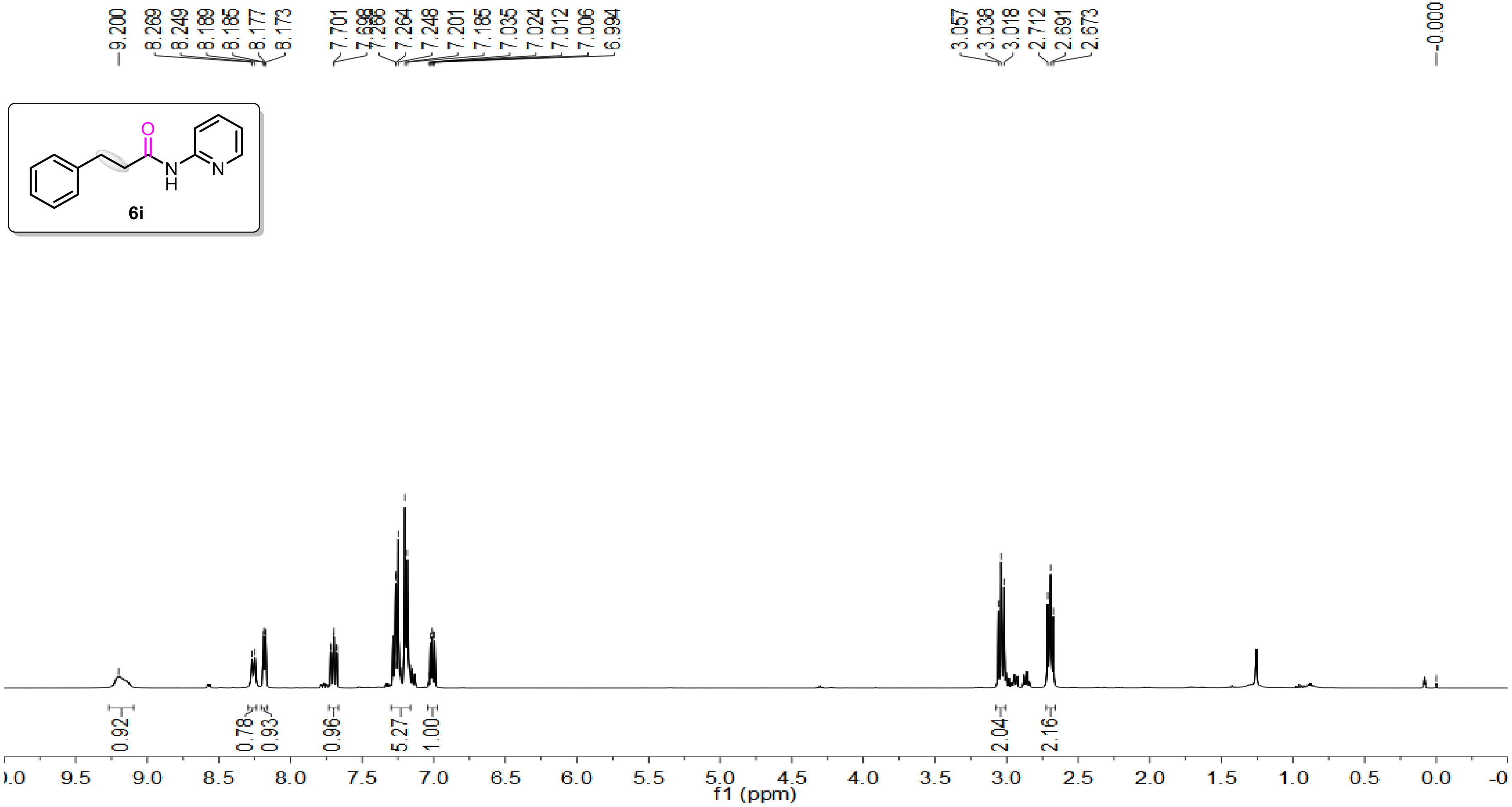

Supplementary Fig. 185. ^{13}C NMR Spectra (101 MHz, $CDCl_3$) of **6i**

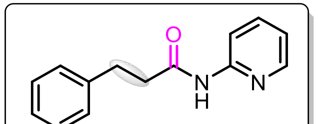

6i

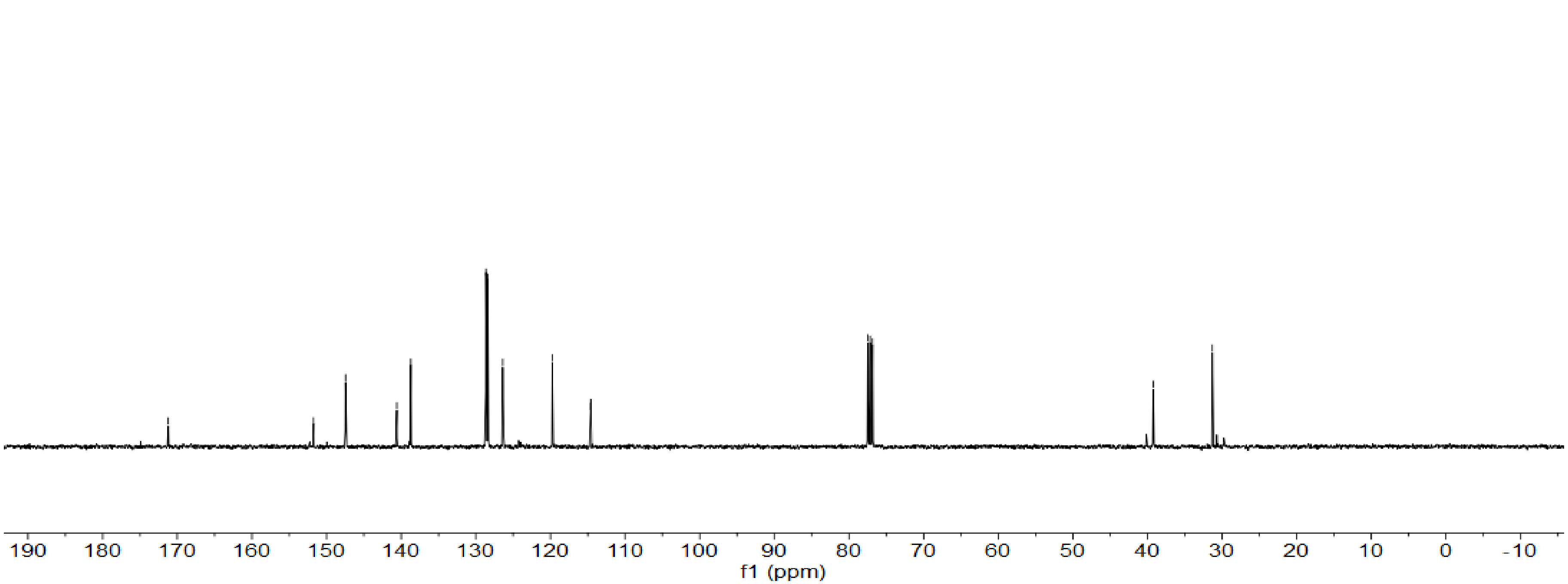

Supplementary Fig. 186. ^{1}H NMR Spectra (400 MHz, $CDCl_3$) of **6j**

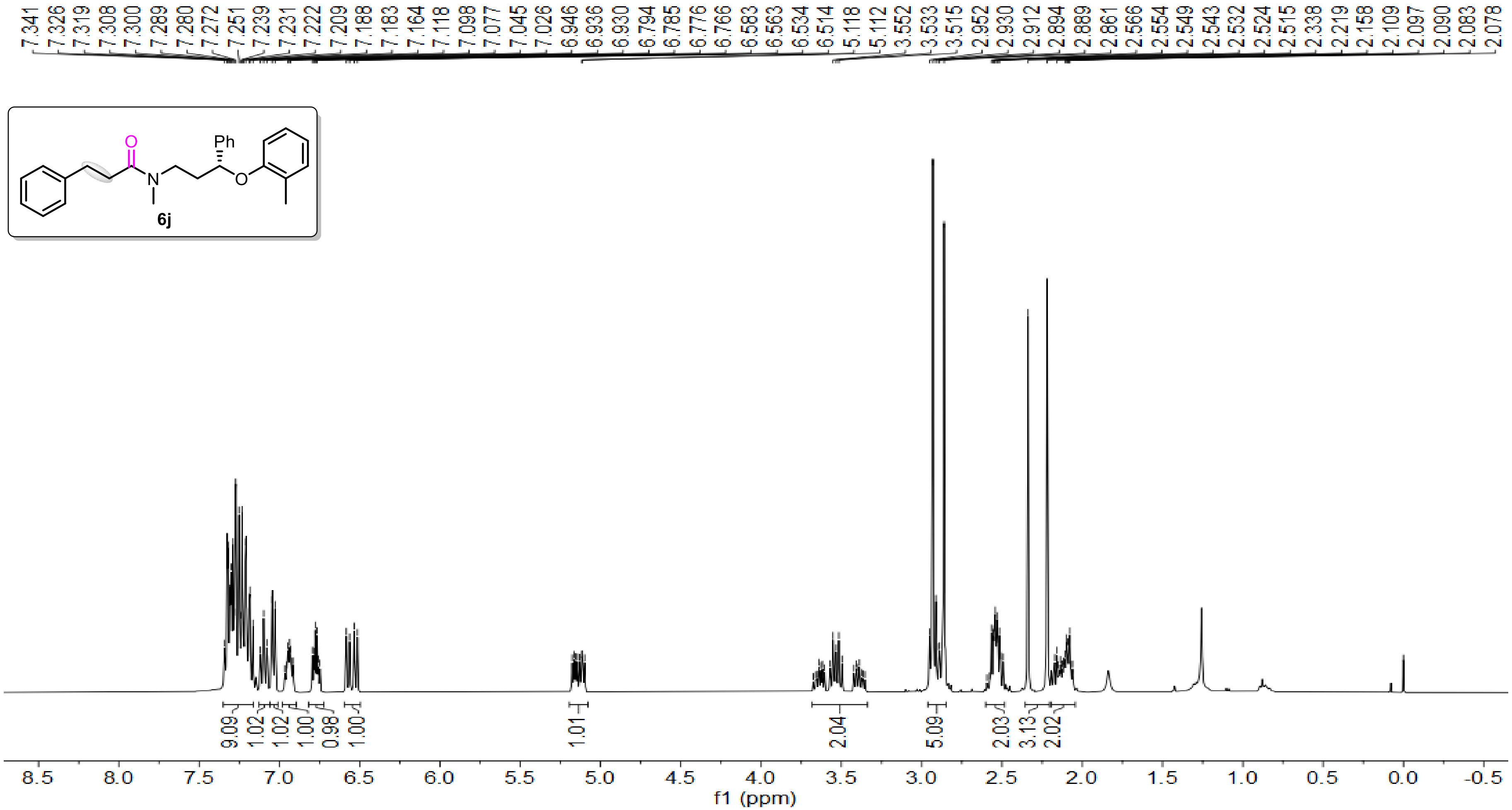

Supplementary Fig. 187. ^{13}C NMR Spectra (101 MHz, $CDCl_3$) of **6j**

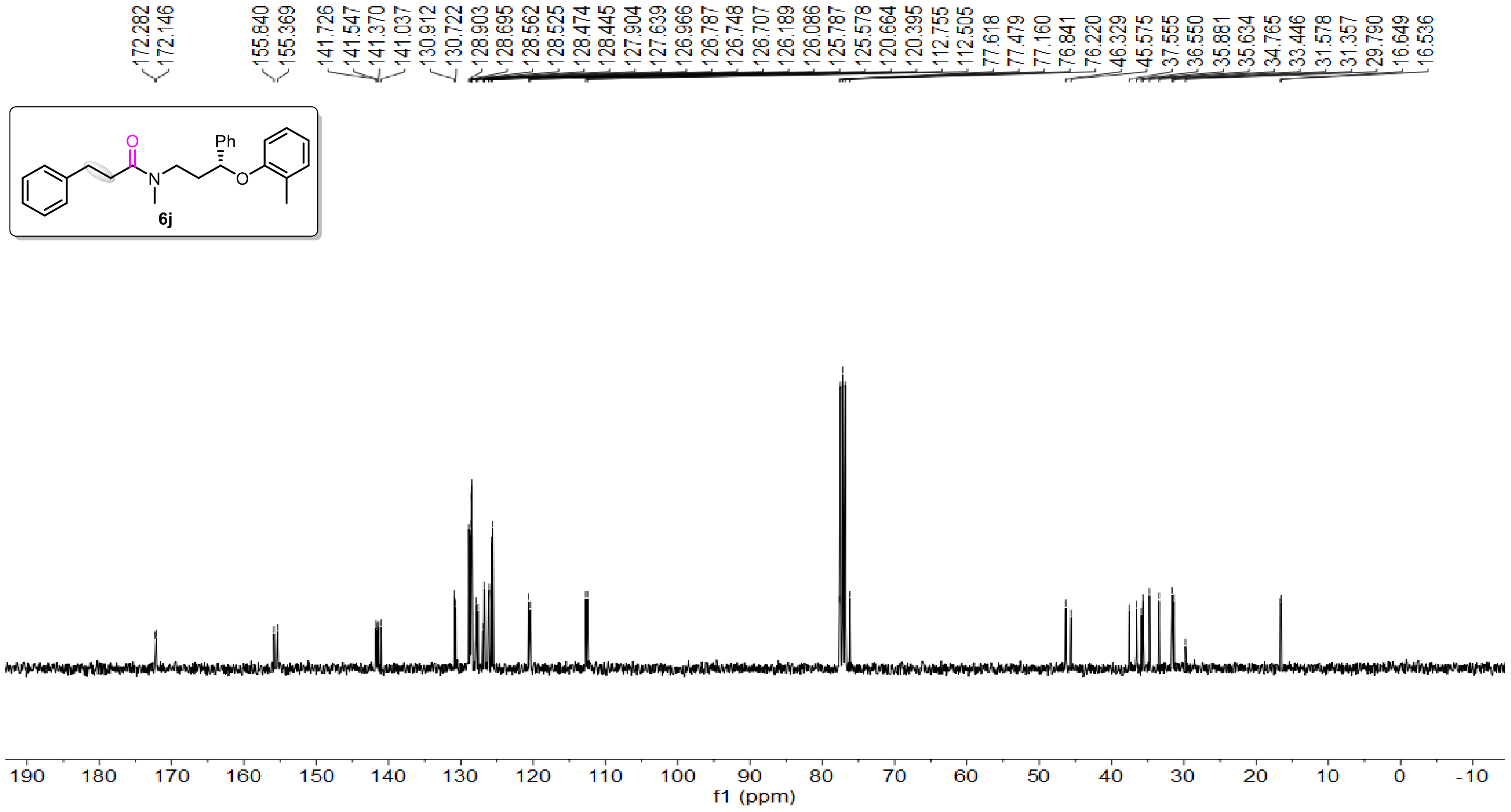

Supplementary Fig. 188. ^{1}H NMR Spectra (400 MHz, $CDCl_3$) of **6k**

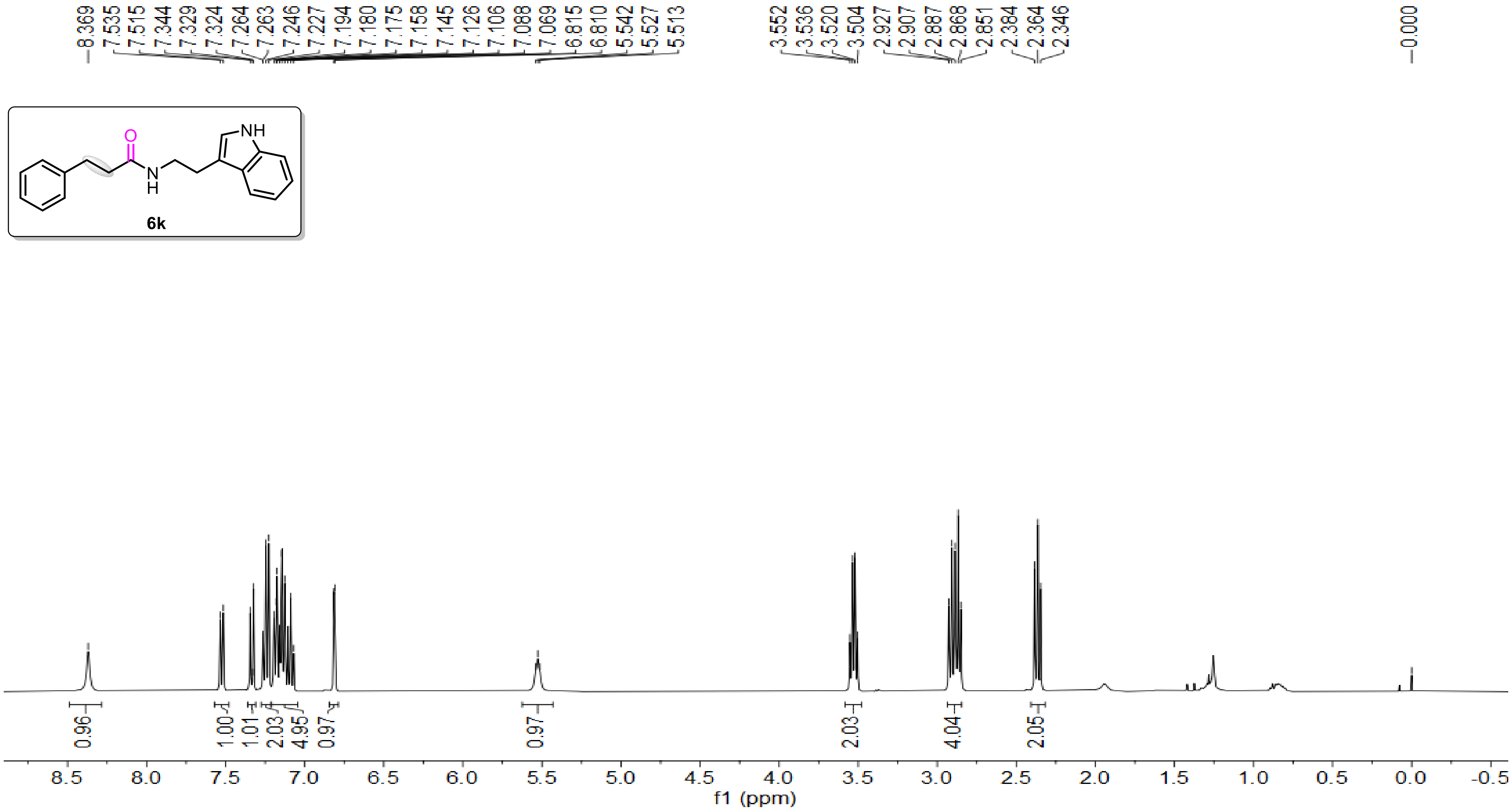

Supplementary Fig. 189. ^{13}C NMR Spectra (101 MHz, $CDCl_3$) of **6k**

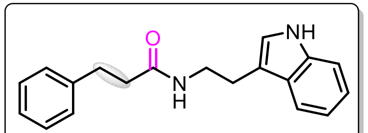

6k

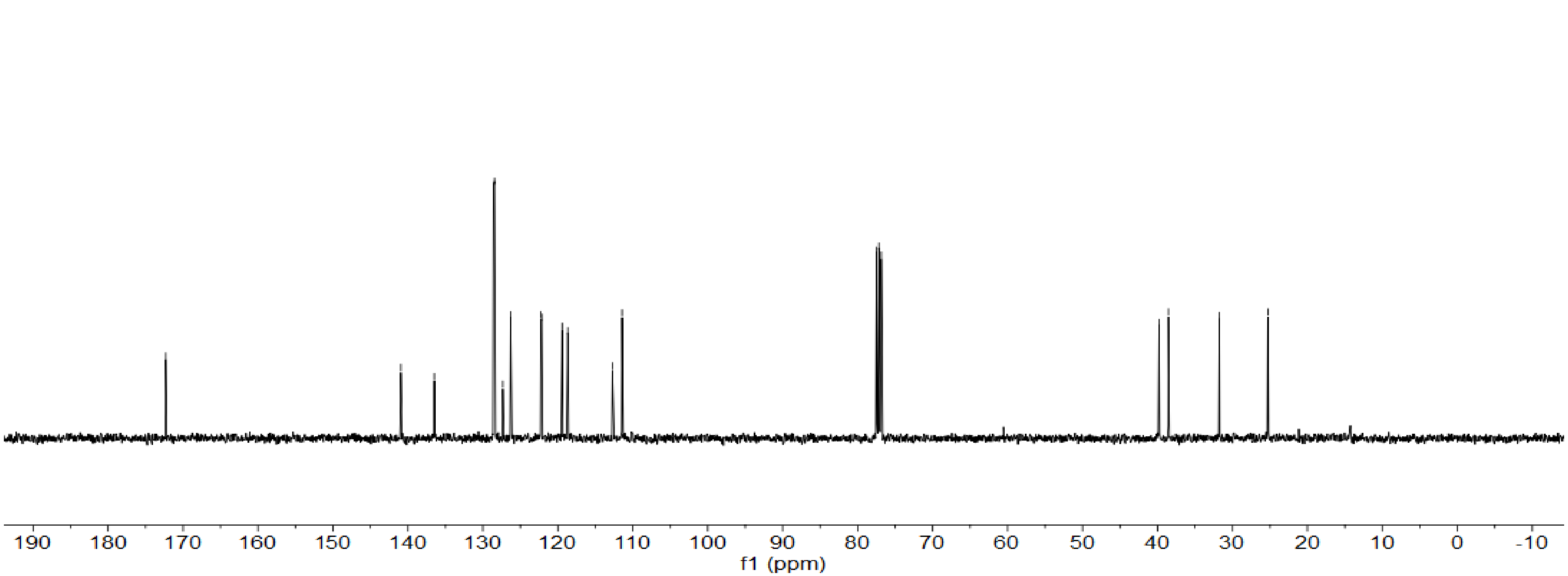

Supplementary Fig. 190. ^{1}H NMR Spectra (400 MHz, $CDCl_3$) of **6l**

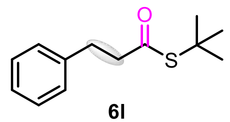

6l

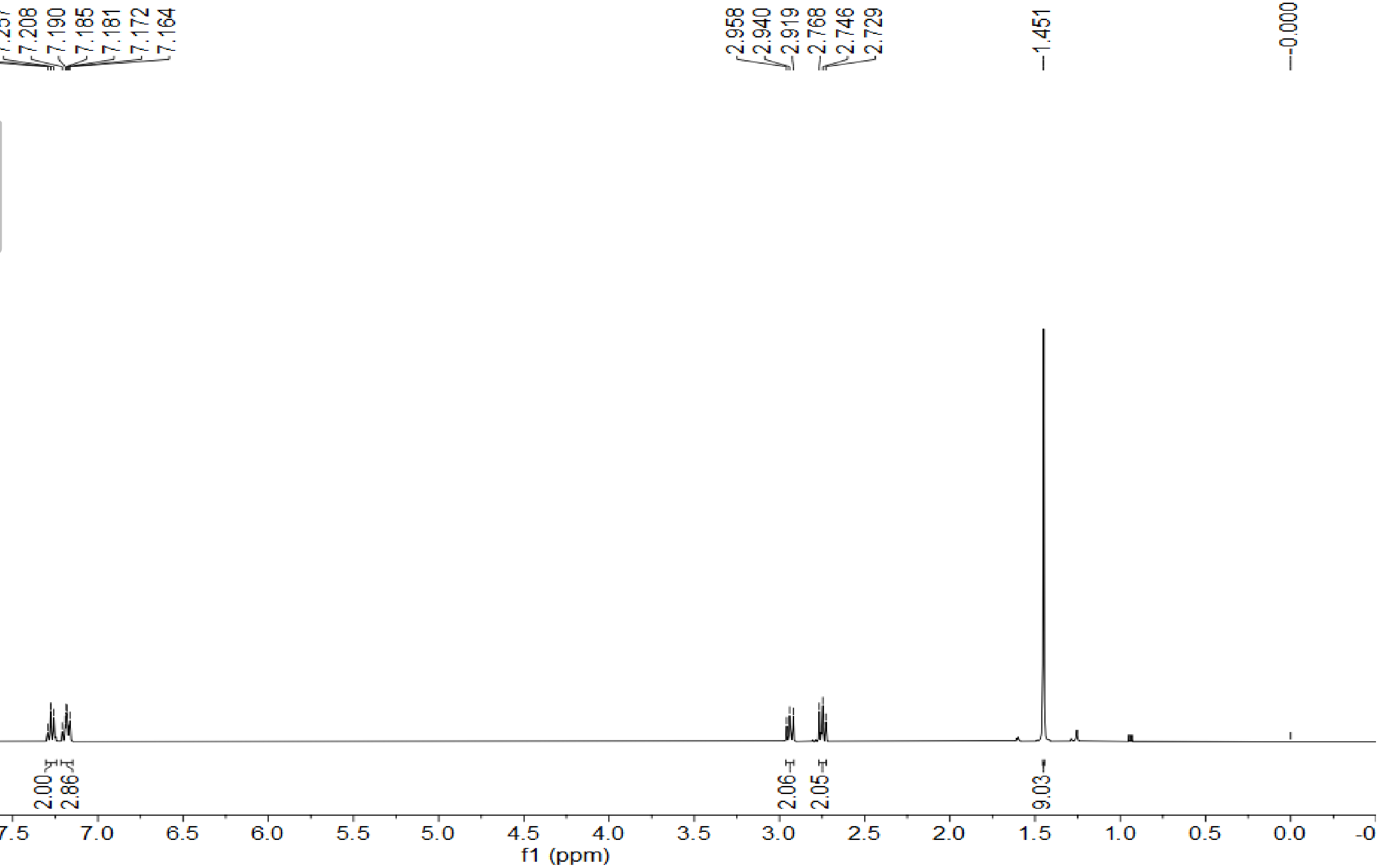

Supplementary Fig. 191. ^{13}C NMR Spectra (101 MHz, $CDCl_3$) of **6l**

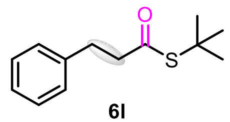

6l

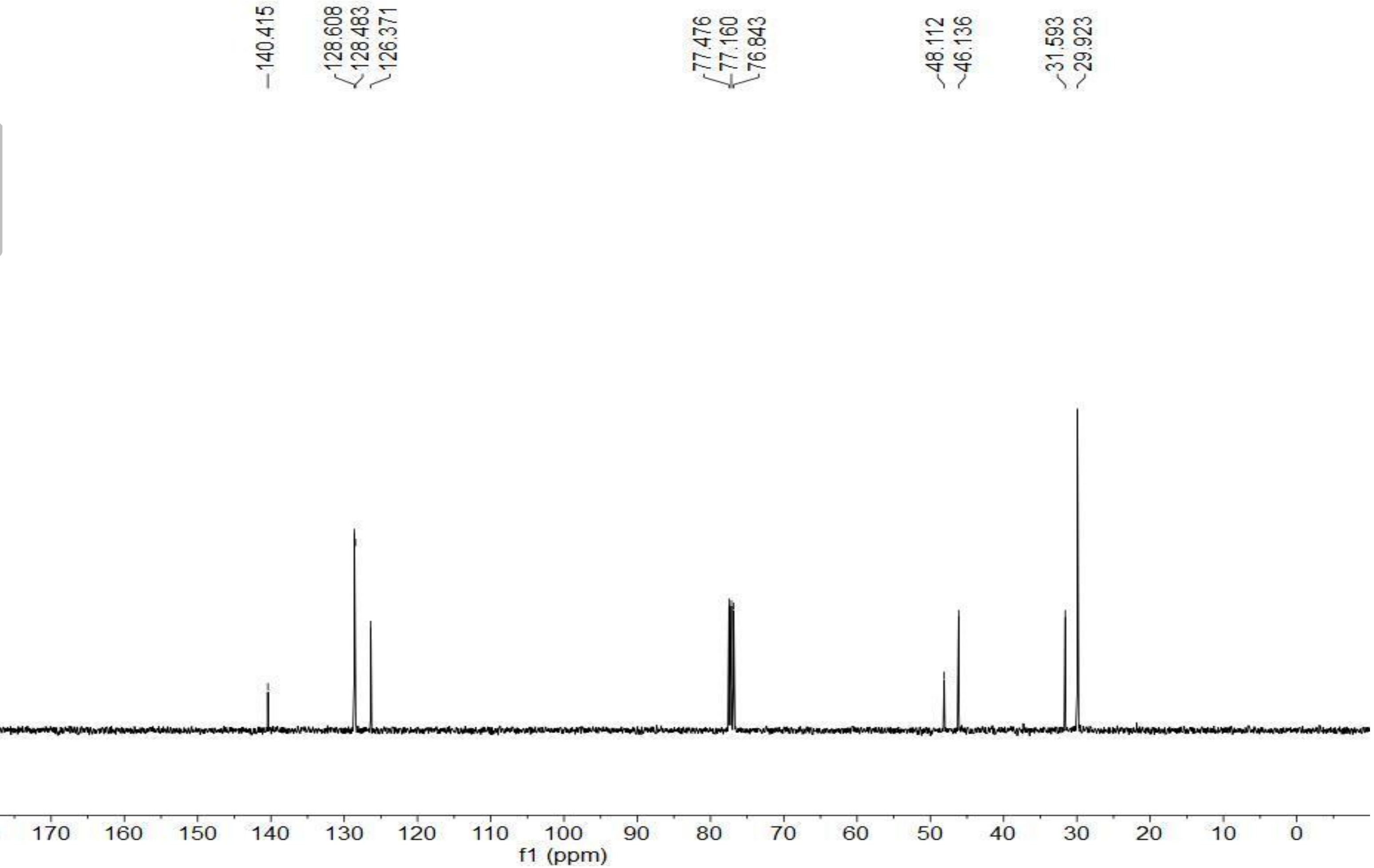

Supplementary Fig. 192. ^{1}H NMR Spectra (400 MHz, $CDCl_3$) of **6m**

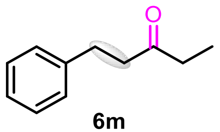

6m

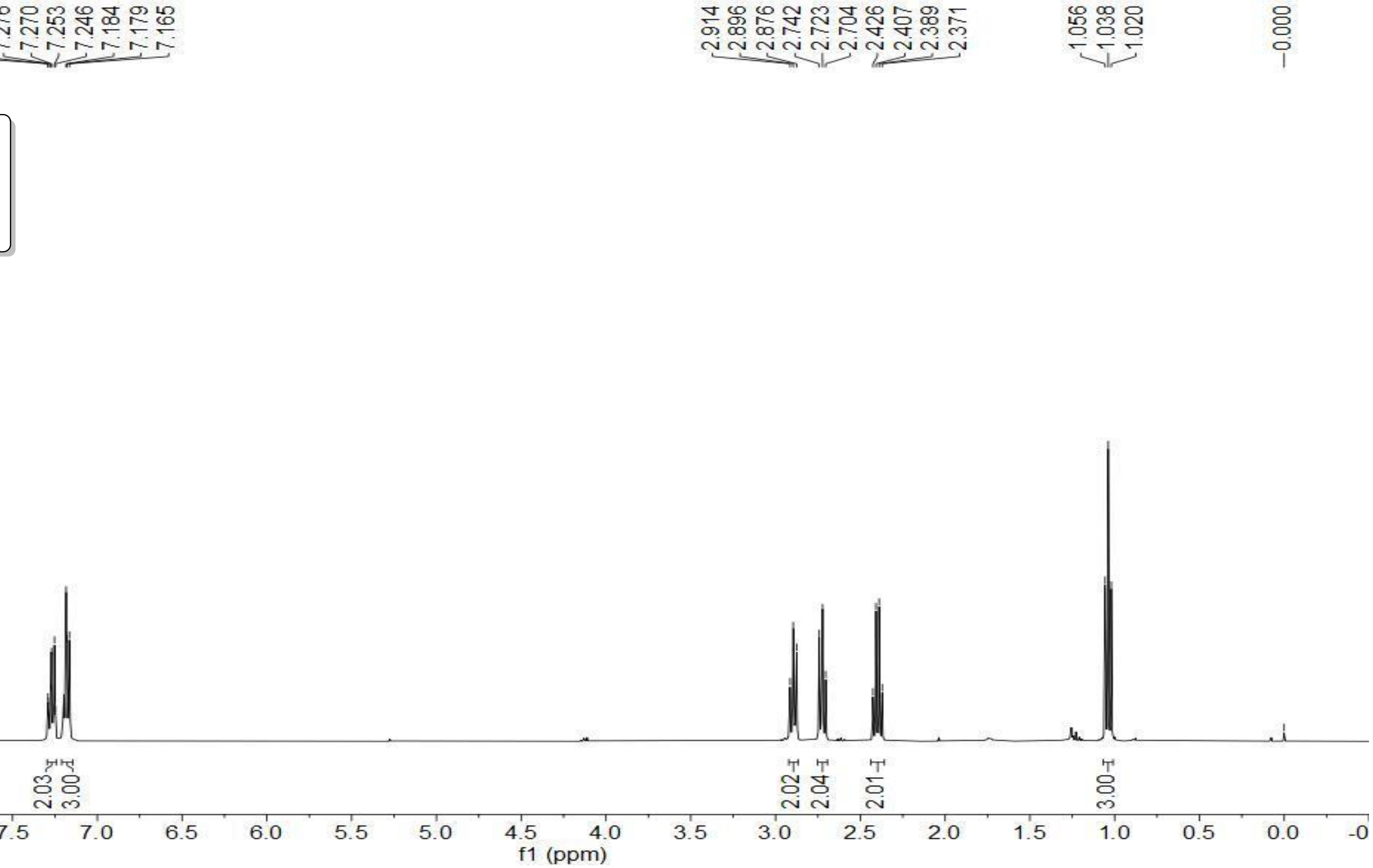

Supplementary Fig. 193. ^{13}C NMR Spectra (101 MHz, $CDCl_3$) of **6m**

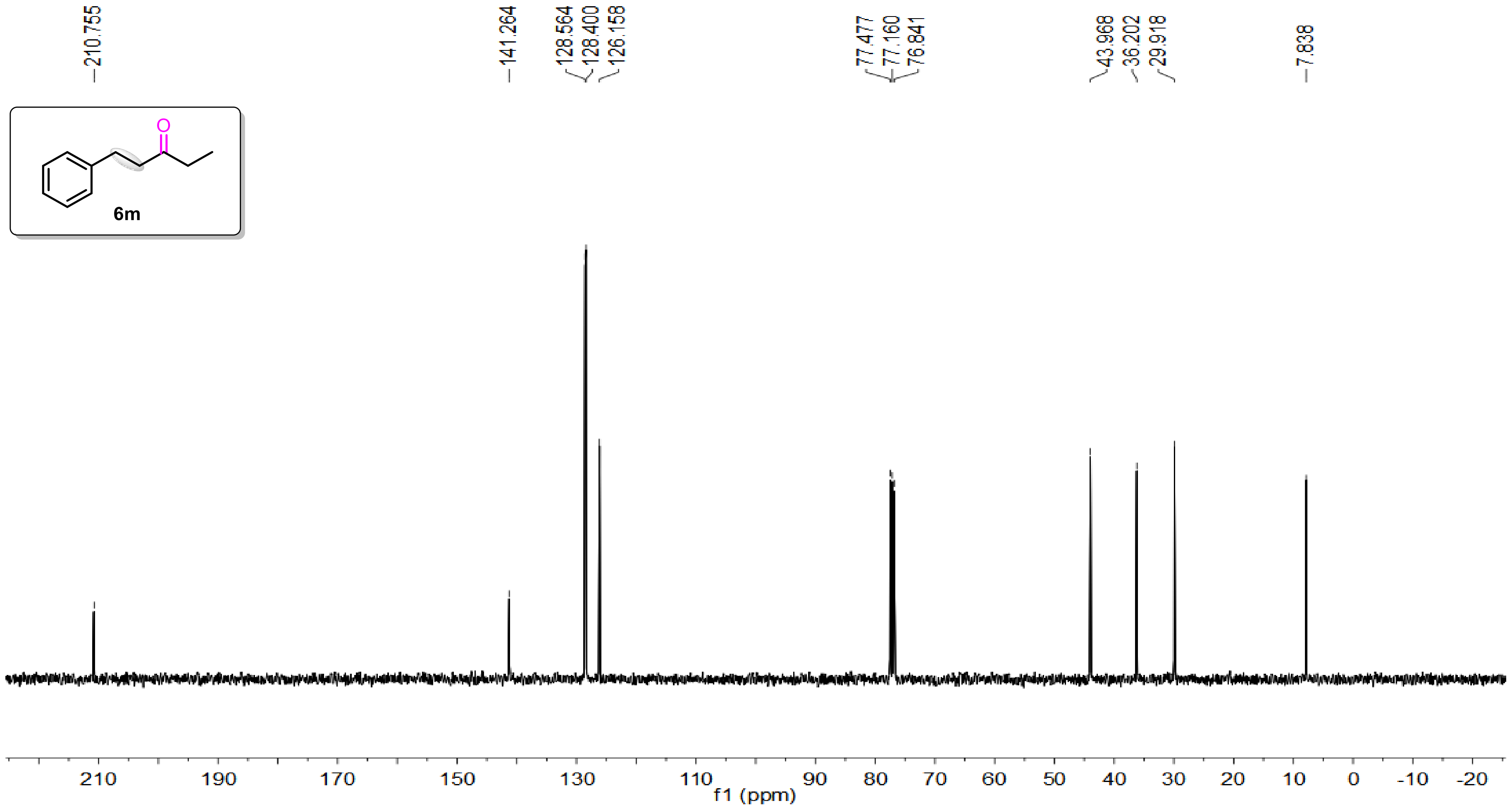

Supplementary Fig. 194. ^{1}H NMR Spectra (400 MHz, $CDCl_3$) of **6n**

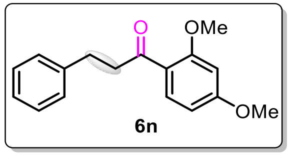

6n

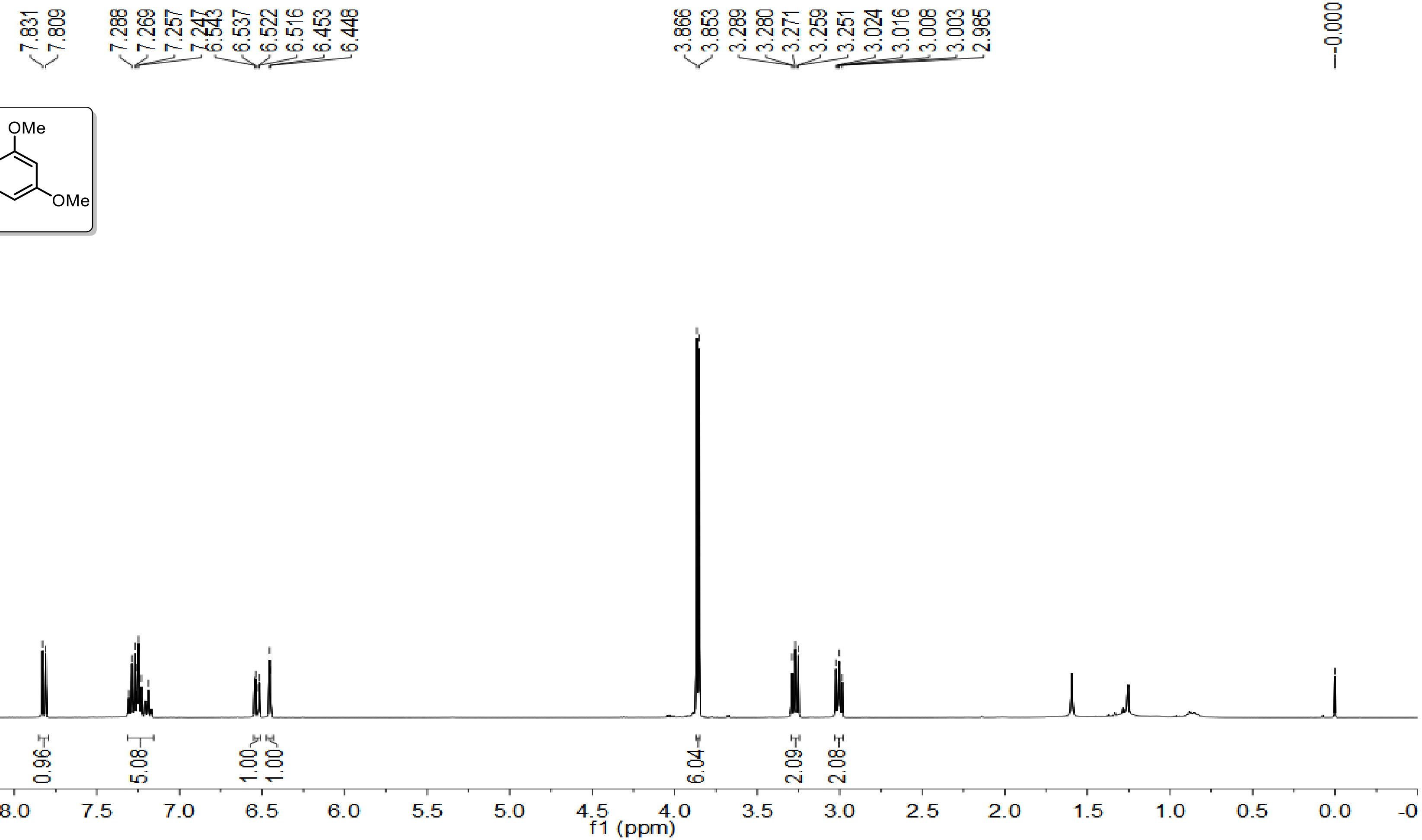

Supplementary Fig. 195. ^{13}C NMR Spectra (101 MHz, $CDCl_3$) of **6n**

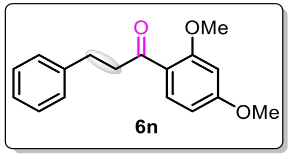

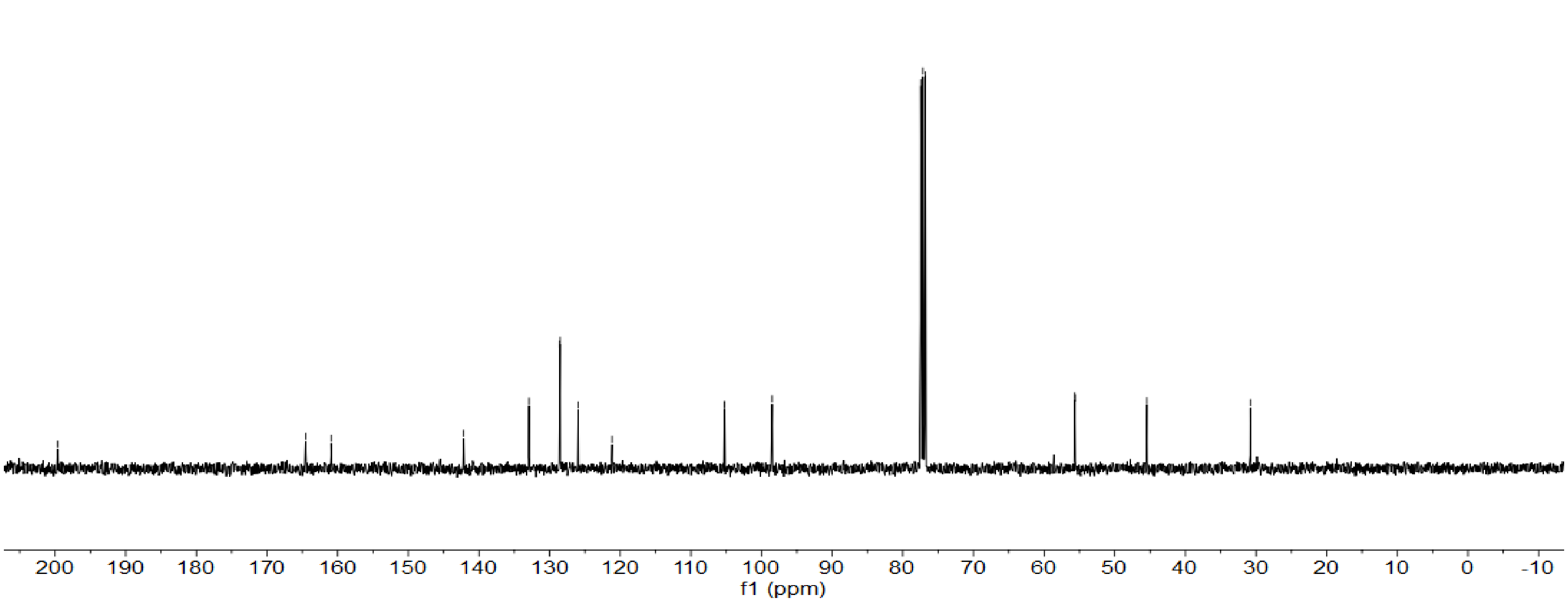

Supplementary Fig. 196. ^{1}H NMR Spectra (400 MHz, $CDCl_3$) of **7a**

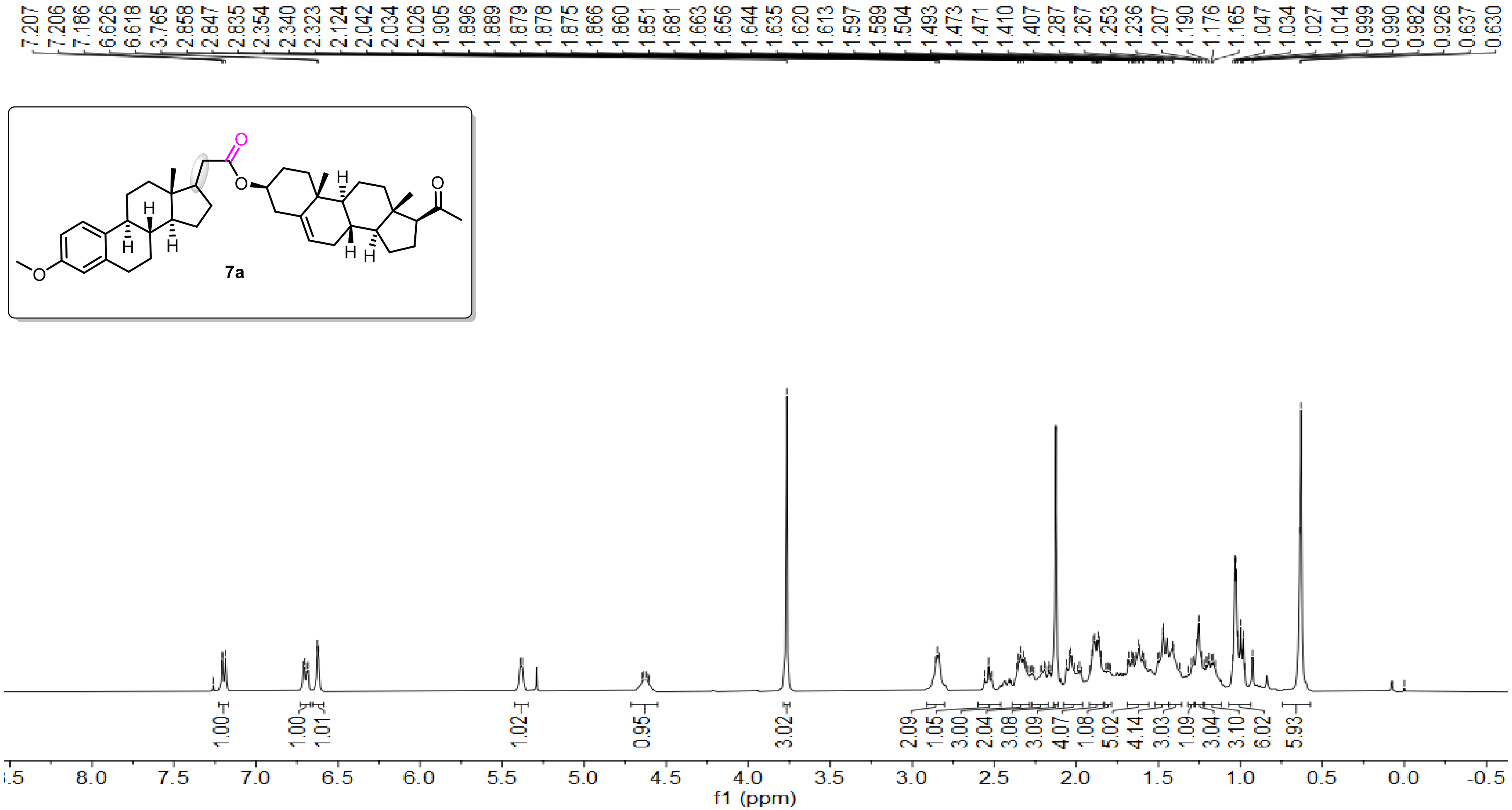

Supplementary Fig. 197. ^{13}C NMR Spectra (101 MHz, $CDCl_3$) of **7a**

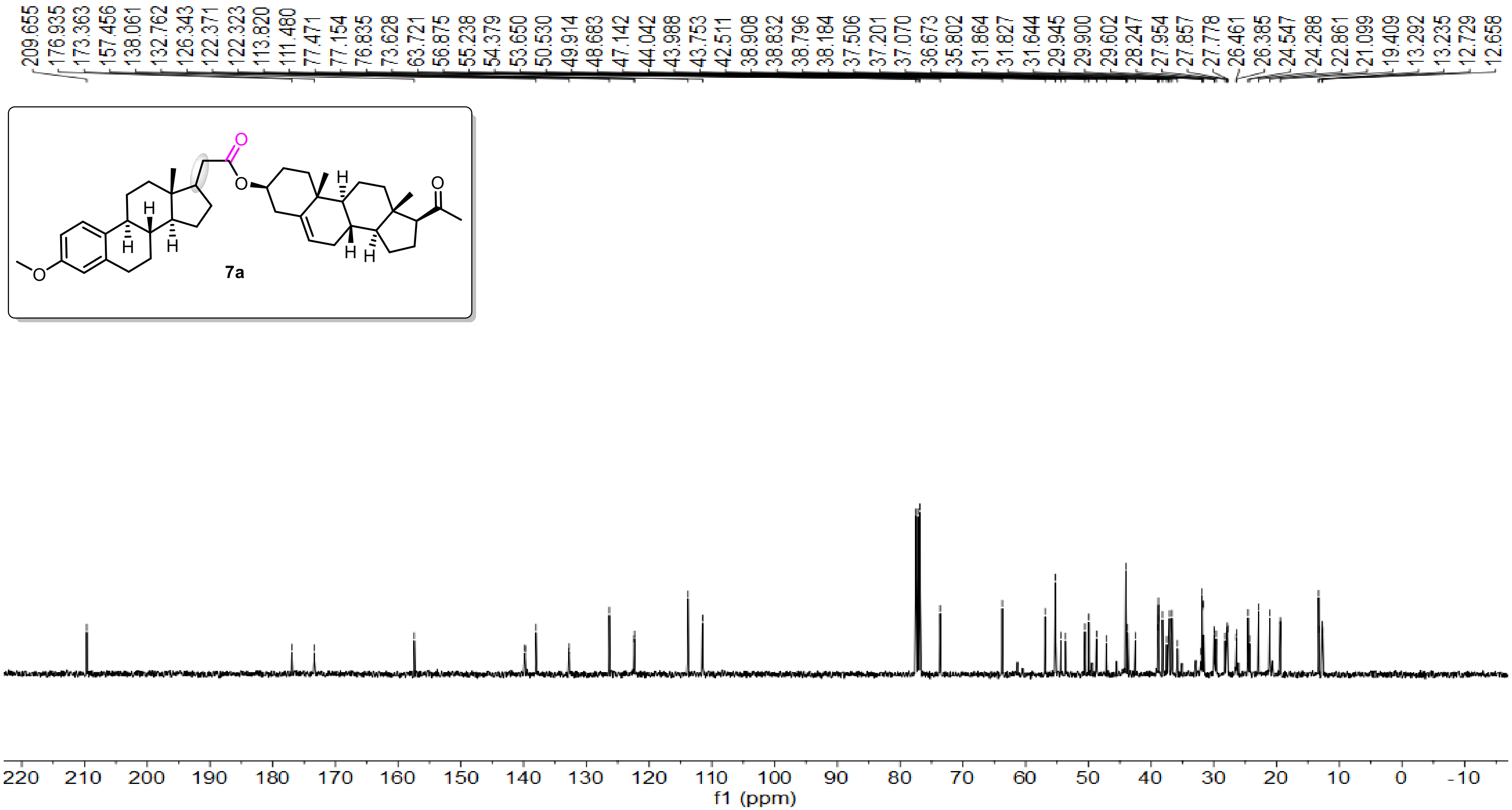

Supplementary Fig. 198. ^{1}H NMR Spectra (400 MHz, $CDCl_3$) of **7b**

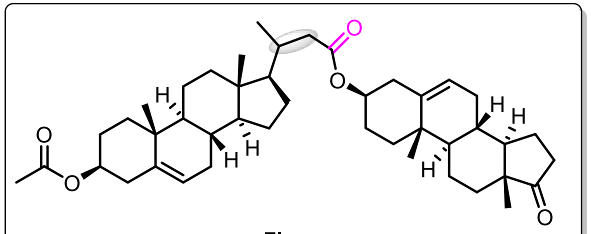

7b

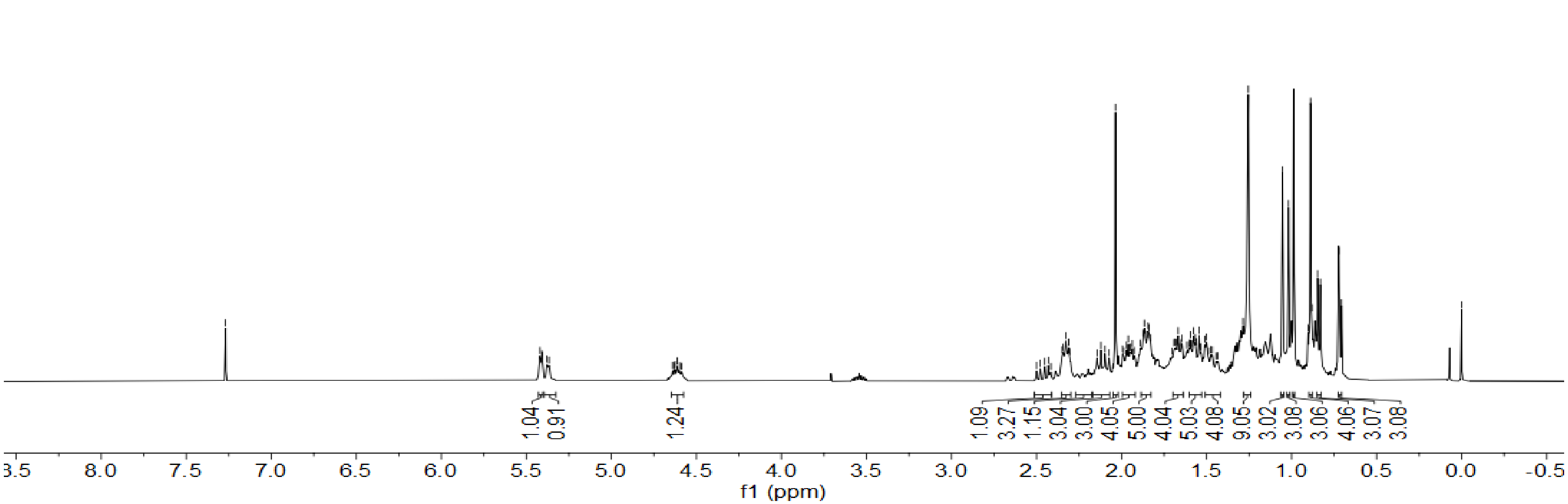

Supplementary Fig. 199. ^{13}C NMR Spectra (101 MHz, $CDCl_3$) of **7b**

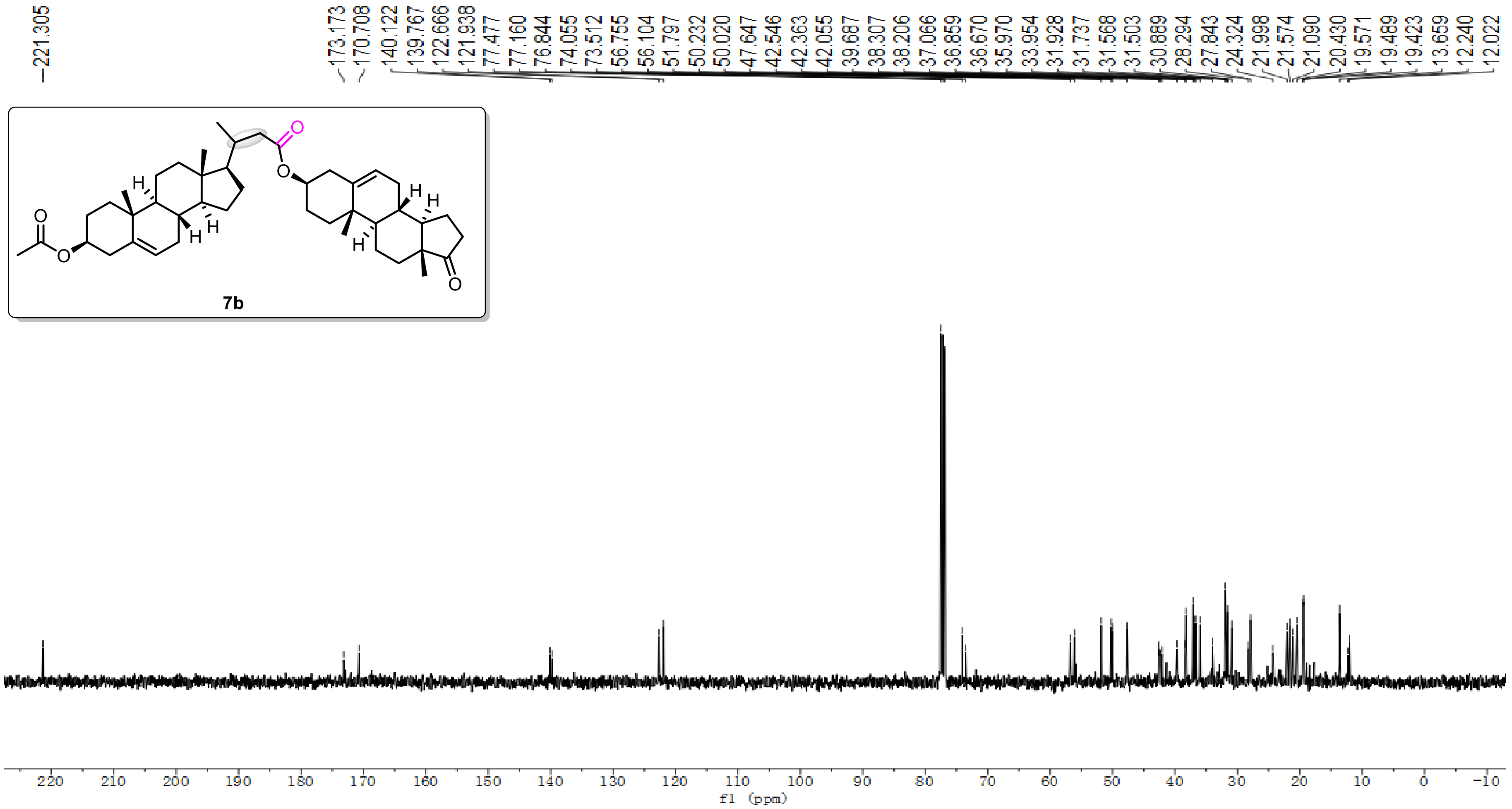

Supplementary Fig. 200. ^{1}H NMR Spectra (400 MHz, $CDCl_3$) of **7c**

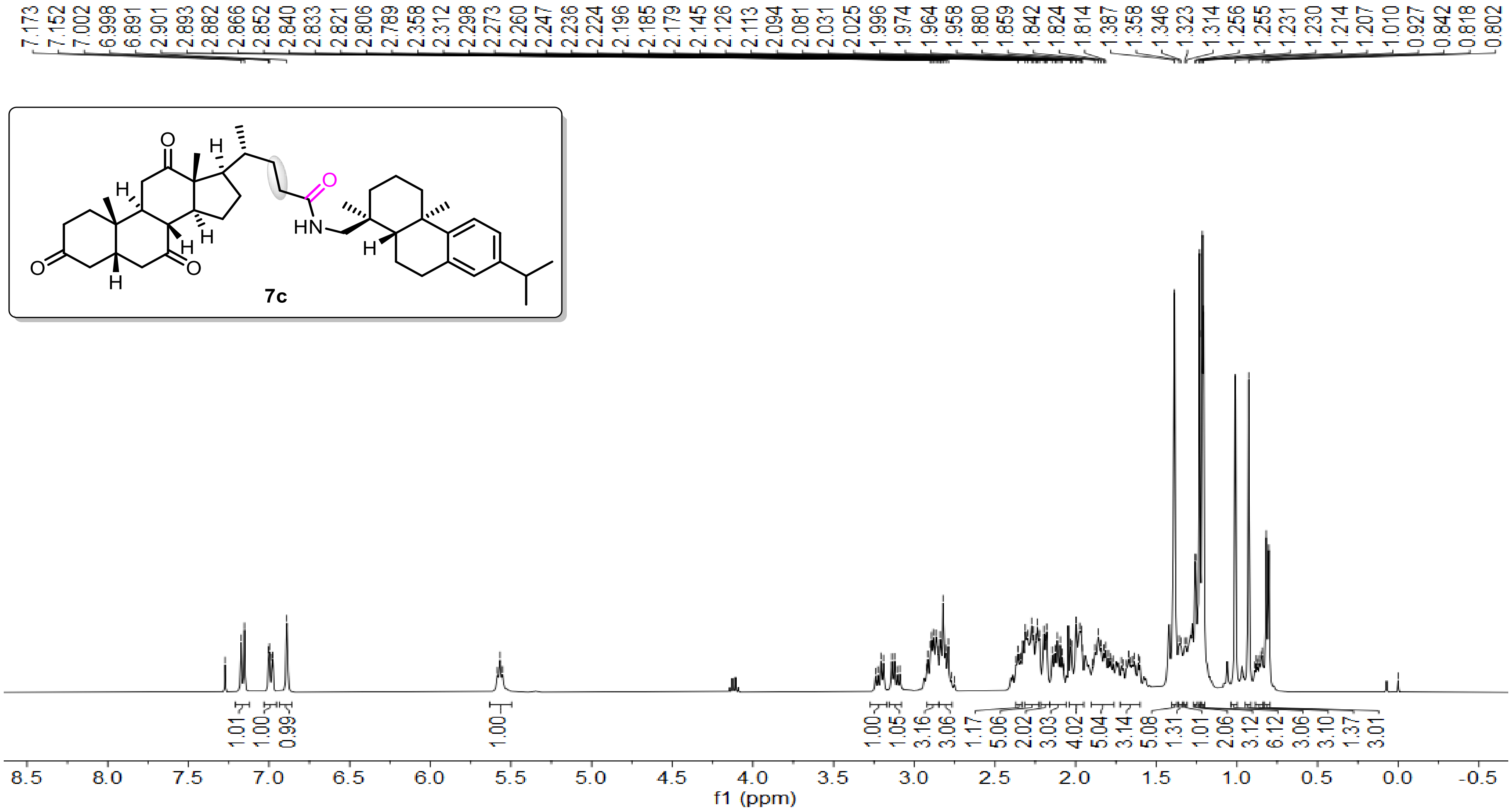

Supplementary Fig. 201. ^{13}C NMR Spectra (101 MHz, $CDCl_3$) of **7c**

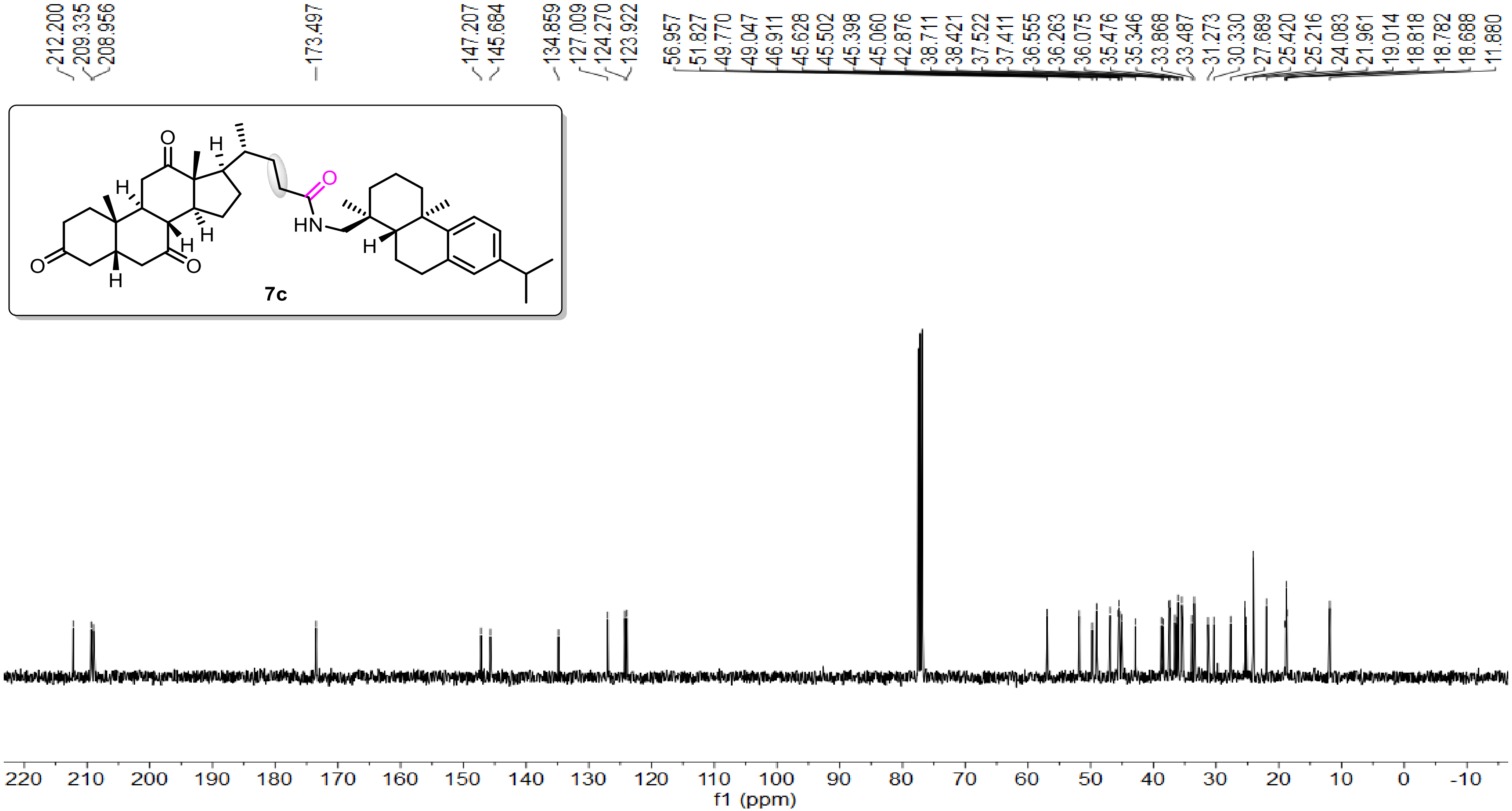

Supplement: Supplementary file 1 — Supplementary Information [file 41467_2023_38748_MOESM1_ESM.pdf]
